# Single-Cell RNA Sequencing Reveals the Role of Epithelial Cell Marker Genes in Predicting the Prognosis of Colorectal Cancer Patients

**DOI:** 10.1155/2022/8347125

**Published:** 2022-08-01

**Authors:** Kai-yu Shen, Bin-yu Chen, Wen-cang Gao

**Affiliations:** ^1^The Second Clinical Medical College of Zhejiang Chinese Medical University, Hangzhou, Zhejiang, China; ^2^Department of Oncology, The Second Affiliated Hospital of Zhejiang Chinese Medical University, Hangzhou, Zhejiang, China

## Abstract

Single-cell RNA sequencing (scRNA-seq) is increasingly used in studies on gastrointestinal cancers. This study investigated the prognostic value of epithelial cell-associated biomarkers in colorectal cancer (CRC) using scRNA-seq data. We downloaded and analysed scRNA-seq data from four CRC samples from the Gene Expression Omnibus (GEO), and we identified marker genes of malignant epithelial cells (MECs) using CRC transcriptome and clinical data downloaded from The Cancer Genome Atlas (TCGA) and GEO as training and validation cohorts, respectively. In the TCGA training cohort, weighted gene correlation network analysis, univariate Cox proportional hazard model (Cox) analysis, and least absolute shrinkage and selection operator regression analysis were performed on the marker genes of MEC subsets to identify a signature of nine prognostic MEC-related genes (MECRGs) and calculate a risk score based on the signature. CRC patients were divided into high- and low-risk groups according to the median risk score. We found that the MECRG risk score was significantly correlated with the clinical features and overall survival of CRC patients, and that CRC patients in the high-risk group showed a significantly shorter survival time. The univariate and multivariate Cox regression analyses showed that the MECRG risk score can serve as an independent prognostic factor for CRC patients. Gene set enrichment analysis revealed that the MECRG signature genes are involved in fatty acid metabolism, p53 signalling, and other pathways. To increase the clinical application value, we constructed a MECRG nomogram by combining the MECRG risk score with other independent prognostic factors. The validity of the nomogram is based on receiver operating characteristics and calibration curves. The MECRG signature and nomogram models were well validated in the GEO dataset. In conclusion, we established an epithelial cell marker gene-based risk assessment model based on scRNA-seq analysis of CRC samples for predicting the prognosis of CRC patients.

## 1. Introduction

Colorectal cancer (CRC) is among the most common malignant tumours of the digestive system worldwide, and its high incidence and mortality are second only to those of lung cancer and breast cancer [1, 2]. Unhealthy lifestyle habits, such as smoking, drinking, and consuming a high-fat diet, have led to an increase in CRC incidence [3], with 1.5% annual increase reported in people aged 30 to 39 years from 2007 to 2016 [4]. At present, tumour node metastasis (TNM) staging, histopathology, and completion of surgical resection are mainly evaluated to determine CRC prognosis, and molecular markers are widely used for CRC diagnosis and treatment. Various medical treatments, including surgery, postoperative adjuvant chemoradiotherapy, and molecular targeted therapy, have been used to treat CRC [5]. CRC symptoms are generally minor in early disease stages, and therefore, patients are often at an advanced stage of the disease by the time they seek medical attention. Furthermore, the optimal treatment timing can easily be missed in cases of advanced, metastatic, and recurrent CRC, and the effects of conventional treatment are usually inconsistent, resulting in variable prognoses.

As a malignant tumour of epithelial origin, the development and progression of CRC are closely related to epithelial tissue [6, 7]. Normal epithelial cells have antitumor activities and are able to eliminate oncogenic transformed cells by regulating cytoskeletal proteins [8]. According to Lv et al., epithelial cells secrete periostin, which inhibits the growth of gastric cancer cells by stabilising P53 and E-cadherin proteins [9]. Epithelial cells exhibit apical-basal polarity and cell–cell adhesion. Correct regulation of polarity is essential to inhibit tumour growth [10]. Royer and Lu suggested that the malignant transformation of epithelial cells in the presence of oncogene activation is generally closely associated with the loss of cell polarity and disorganisation and that the disruption of epithelial cell polarity promotes epithelial-mesenchymal transition (EMT), which is a key step in the invasion of the surrounding stroma by epithelial tumour cells [11]. In addition, under hypoxic conditions, pulmonary epithelial cells can downregulate connexin (CX)26 and CX43 via the P53 signalling pathway to promote lung carcinogenesis [12]. Epithelial cells are both the structural basis of most organ tissues in the body and the source of most human tumours [13]. With the progressive research on epithelial cells, epithelial cell-related biomarkers have become a research hotspot in recent years. Keratins are widely used as epithelial cell biomarkers in the pathological diagnosis of tumours and in predicting survival prognosis. In CRC patients, decreased expression of keratin (K)8 and K20 is closely related to EMT and suggestive of poor survival and high tumour invasion [14]. Increased serum levels of a cleaved K18 fragment produced by apoptotic epithelial cells suggest a high risk of CRC recurrence within 3 years [15]. However, because of the uncontrollable pathological types, stages, and metastases of CRC [16], using conventional biomarkers to predict prognosis does not achieve adequate results. Therefore, to explore more epithelial cell-related biomarkers with clinical application potential, we used high-resolution omics tools to perform a more accurate analysis of CRC epithelial cells.

Single-cell RNA sequencing (scRNA-seq) allows the construction of a gene-regulatory network at the cellular level [17]. Analysis of the genome, transcriptome, or epigenome of single cells individually or simultaneously enables the detection of gene expression profiles and tracking of cell development at the single-cell level [18]. The RNA-seq technology has been widely used to evaluate the tumour immune microenvironment [19, 20]; however, few studies have applied this method to CRC epithelial cells to predict CRC prognosis.

In this study, we identified marker genes of CRC epithelial cell subsets using scRNA-seq analysis, determined the prognostic significance of nine malignant epithelial cell- (MEC-) related genes (MECRGs) using data from The Cancer Genome Atlas (TCGA) and the Gene Expression Omnibus (GEO), and integrated the MECRG-based risk score and clinical traits to construct a nomogram for the prediction of CRC patient prognosis.

## 2. Materials and Methods

### 2.1. Data Availability

ScRNA-seq data of four CRC samples were downloaded from the GEO database (https://www.ncbi.nlm.nih.gov/geo/) (GSE161277). Relevant clinical information related to the CRC samples is provided in Supplementary Table S1. The dataset comprises four CRC samples, four adenoma samples, three normal tissue samples, one paracancer sample, and one blood sample [21]. Tissue samples other than the CRC samples were not considered in this study. Transcriptome data of 473 CRC samples and 41 nontumour tissues were obtained from the TCGA database (https://portal.gdc.cancer.gov/), including 435 CRC samples with matched clinical data. The extracted clinical information included sex, age, and stage. TCGA-CRC samples with complete clinical information were used to construct the training set used to develop a prediction score. A total of 232 CRC samples with complete clinical information obtained from the GEO database (GSE17538) were used as the external validation set.

### 2.2. scRNA-seq Data Processing

The scRNA-seq data of the four CRC samples were processed using the R language (v4.1.0; https://www.r-project.org/) and the “Seurat” package [22] (https://cran.r-project.org/web/packages/Seurat/index.html). The “harmony” package was used to remove batch effects from the scRNA-seq data of the four CRC samples. First, we determined the percentage of mitochondrial genes in each cell using the “PercentageFeatureSet” function with the parameter set to pattern = “^MT-”. Subsequently, using the “subset” function, genes expressed in <10 cells and cells expressing <200 genes were eliminated. Further, we excluded noncells and cell aggregates. Cell samples with a mitochondrial gene proportion < 15% were included in the subsequent analysis and logarithmically normalized. Principal component analysis (PCA) was used for unsupervised clustering, and the “JackStraw” function was used to determine and visualise the number of principal components. We used nonlinear dimension reduction (t-distributed stochastic neighbour embedding; t-SNE) clustering and the “FindAllMarkers” function (parameters: minimum% = 0.3, log function threshold = 0.25) to identify marker genes based on between-cluster differences. The cell clusters were then annotated based on reported cell-specific marker genes [23–29].

### 2.3. Analysis of Chromosome Copy Number Variation (CNV) and Screening of MEC Marker Genes

To distinguish epithelial cell malignancy, we identified chromosome CNV in each sample using the “infercnv” package [30] (https://github.com/broadinstitute/inferCNV; default parameters). Using immune cells as a reference [31], we calculated CNV scores for epithelial cell subsets and defined the epithelial cell subset showing a median CNV score > 1,000 as the MEC subset, which was used for subsequent analysis. Differences in CNV scores between epithelial cell clusters were compared using the Kruskal–Wallis test. We then identified marker genes in the MEC subset based on a |log2(*fold* *change*)| > 0.5 and a false discovery rate (FDR) < 0.01.

### 2.4. Analysis of Differential Gene Enrichment of Epithelial Cell Subsets

The potential biological mechanisms of the MEC marker genes were determined using the “clusterProfiler” (https://bioconductor.org/packages/release/bioc/html/clusterProfiler.html) and “org.Hs.eg.db” (https://www.bioconductor.org/packages/release/data/annotation/html/org.Hs.eg.db.html) packages. Gene Ontology (GO) and Kyoto Encyclopedia of Genes and Genomes (KEGG) enrichment analyses were performed, using a *q* value < 0.05 to determine statistically significant enrichment.

### 2.5. MEC Marker Gene Analysis Using Weighted Gene Correlation Network Analysis (WGCNA)

WGCNA can be used to find modules of correlated genes and identify disease-related biomarkers. We used the WGCNA R package [32] (https://horvath.genetics.ucla.edu/html/CoexpressionNetwork/Rpackages/WGCNA/) to identify MEC marker genes related to CRC. We generated a similarity matrix between MEC marker genes using the Pearson correlation analysis and then calculated an adjacency matrix and constructed a topological overlap matrix. Next, we plotted a tree diagram of modules and clustered closely related MEC marker genes within the modules using hierarchical clustering. The MEC marker genes in the final correlation module (*P* < 0.05) were used for subsequent analysis.

### 2.6. Construction and Validation of the MECRG Signature

The univariate Cox analysis was used to find MECRGs significantly related to overall survival (OS) in the TCGA-CRC cohort, and a forest map was plotted. MECRGs were identified using least absolute shrinkage and selection operator (LASSO) analysis, using 10-fold cross-validation and 1,000 iterations to determine the minimum value of the penalty parameter (*λ*) and construct a MECRG signature. The regression coefficients of the nine MECRGs thus identified were calculated using the “predict” function. The following formula was used to calculate the risk score:

(1) $$\begin{array}{c} \text{MECRG risk score}=\sum X,G*\text{coef }G, \end{array}$$

where “coef G” is the regression coefficient and “*X* *G*” describes the expression levels of the MECRGs. Patients from the TCGA-CRC training cohort were divided into high- and low-risk groups according to the median value of the MECRG risk score. A Kaplan–Meier survival curve was used to explore differences in survival and prognosis between the two groups. We then used a receiver operating characteristic (ROC) curve to evaluate the predictive value of the MECRG signature. Finally, the signature was validated using the GSE17538 GEO dataset.

### 2.7. Analysis of the Prognostic Accuracy of the MECRG Risk Score

Combining the MECRG risk score with clinical features, we used univariate and multivariate Cox analyses to assess whether the risk score could serve as an independent prognostic factor. Using the training and validation sets, we then performed a survival analysis of the MECRG signature for patients of different clinical subgroups. Additionally, we identified the relationships between MECRG risk groups and clinical traits (including sex, age, and TNM stage) and generated a heat map.

### 2.8. Gene Set Enrichment Analysis (GSEA) of the MECRG Signature

Patients in the TCGA-CRC training cohort were grouped according to the median value of the risk score and we used GSEA to evaluate the functions of and signalling pathways associated with MECRGs in the high- and low-risk groups, using *P* < 0.05 as a threshold. The “AUCell” package (https://www.rdocumentation.org/packages/msigdbr/versions/7.4.1) was used to present the results of enrichment analysis according to cell subsets.

### 2.9. Construction of a MECRG Nomogram

Using the training set, we integrated the three independent prognostic factors of age, stage, and MECRG risk group to plot a MECRG nomogram capable of predicting the 1-, 3-, and 5-year OS of CRC patients for clinical application. ROC and calibration curves were used to evaluate the predictive value of the MECRG nomogram, and feasibility was confirmed by external validation using the GSE17538 GEO dataset.

### 2.10. Statistical Analysis

The “survival” (https://cran.r-project.org/web/packages/survival/index.html) and “survminer” (https://cran.r-project.org/web/packages/survminer/index.html) packages were used to construct Kaplan–Meier survival curves. The log-rank test was used to determine significant differences in survival between the high- and low-risk groups according to the training and validation datasets. LASSO regression was performed using the “glmnet” package (https://cran.r-project.org/web/packages/glmnet/index.html), and the “timeROC” package (https://cran.r-project.org/web/packages/timeROC/index.html) was used to generate the ROC curve to evaluate model accuracy. The univariate and multivariate Cox regression analyses were used to determine the independent predictors of OS outcomes in CRC patients. The “rms” package (https://cran.r-project.org/web/packages/rms/) was used to construct the nomogram model. Comparisons between two groups were made using the Wilcoxon rank sum test, and comparisons between multiple groups were made using the Kruskal–Wallis test. Correlation analysis was conducted using the Pearson method. *P* < 0.05 was considered significant.

## 3. Results

### 3.1. CRC scRNA-seq Data Analysis

**Figure 1:**
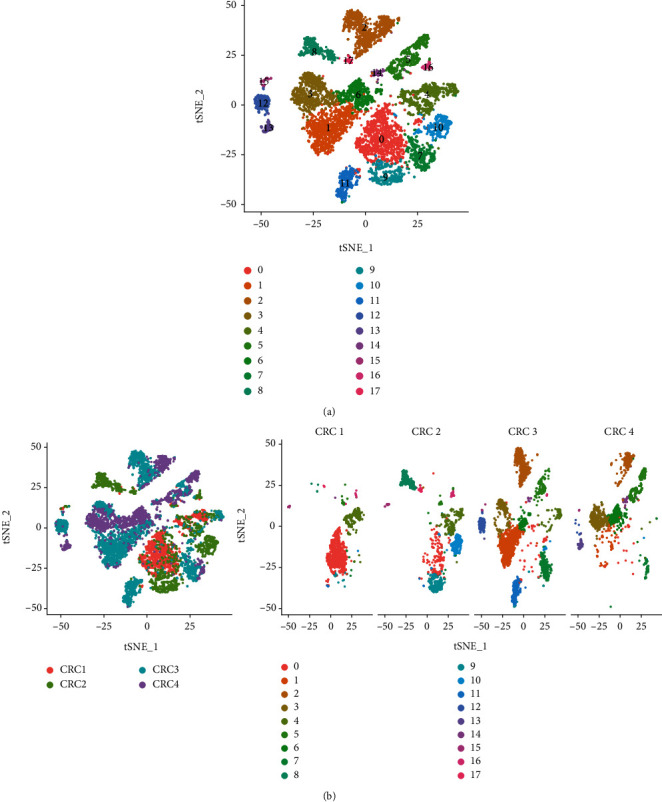
Characterisation of scRNA-seq from 15,465 cells. (a) The cells were classified into 18 subsets using the t-SNE algorithm. (b) Distribution ratios of the cell subsets in the four CRC samples.

**Figure 2:**
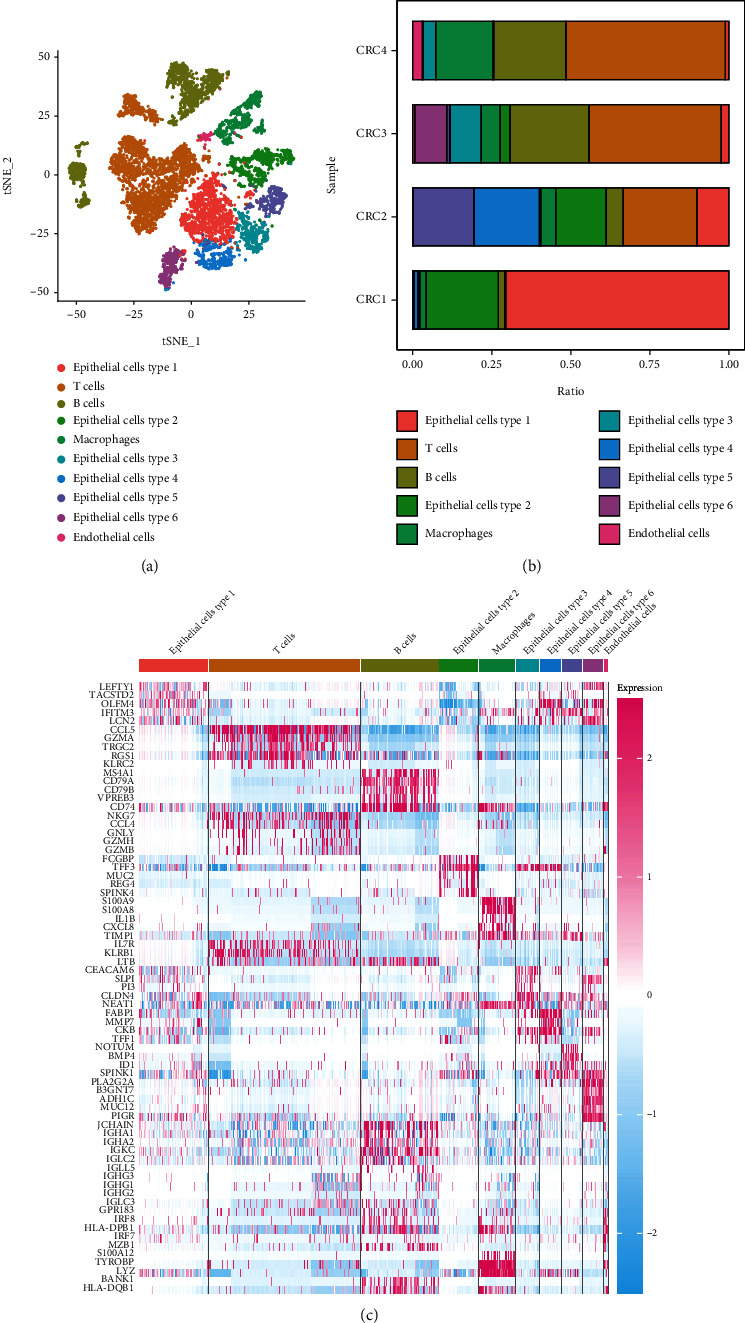
Characteristics of the cell subsets. (a) Annotated cell subsets. (b) Proportions of the various types of cells in the four CRC samples. (c) Heat map of the five most differentially expressed marker genes in each cell subset.

We obtained 15,465 cells from the four CRC samples. After applying the screening criteria, 8,798 high-quality cell samples were obtained. Gene numbers (nFeature_RNA), sequencing depth (nCount_RNA), and mitochondrial gene percentage (percent.mt) are shown in Supplementary Figure S1. The Pearson correlation coefficient between gene count and sequencing depth was 0.92 (Supplementary Figure S2), suggesting a positive correlation. PCA used to classify the high-quality cells identified 40 principal components (Supplementary Figure S3), and t-SNE of the first 11 principal components (*P* < 0.05) (Supplementary Figure S4) allowed visualisation of the high-dimensional CRC scRNA-seq data and the distribution of the cell subsets, as well as classification of the cells into 18 subclasses (Figures 1(a) and 1(b)). Among the 18 subclasses, clusters 0, 4, 7, 9, 10, and 11 were identified as epithelial cell subtypes 1, 2, 3, 4, 5, and 6, respectively, based on the presence of epithelial cell marker genes (*EPCAM*, *KRT19*, and *CDH1*), clusters 2, 12, 13, 15, and 17 were identified as B cell subsets (*CD79A*, *MS4A1*, and *CD19*), clusters 1, 3, 6, and 8 were identified as T cell subsets (*CD3D*, *CD8A*, and *CD3E*), clusters 5 and 16 were identified as macrophage subsets (*CD14*, CD68, and CD163), and cluster 14 was identified as an endothelial cell subset (*IL3RA*, *SERPINF1*, and *CCDC88A*). For the purpose of selecting epithelial cell subsets with high malignancy, we did not merge the epithelial cell subsets. Based on the above annotation effects, we summarised the final cell subpopulation annotations in Figure 2(a). In addition, we calculated the proportions of the five cell types in the four CRC samples, which revealed that the epithelial cell subpopulation accounted for a relatively large fraction (Figure 2(b)). The five most strongly expressed marker genes in each cell subset are shown in Figure 2(c).

### 3.2. Evaluation of Chromosome CNV in the Epithelial Cell Subsets

**Figure 3:**
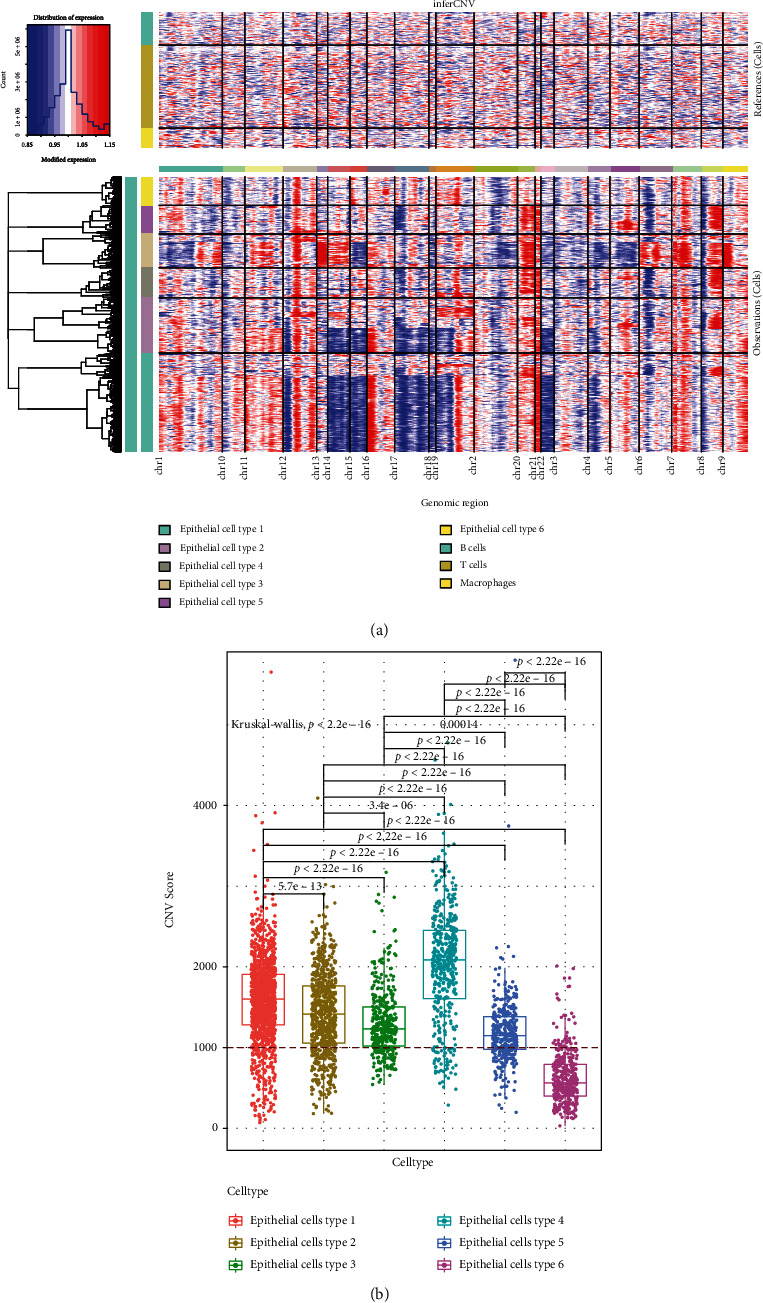
CNV analysis of epithelial cells from CRC patients. (a) Heat map of CNV in epithelial cells from the four CRC samples. (b) CNV score distribution among different epithelial cell subtypes.

We next determined the chromosome CNV in each sample based on the transcriptome data to evaluate the degree of malignancy in the epithelial cell subsets. We observed low CNV in the immune cell subsets (macrophages, B cells, and T cells) in control samples, whereas high CNV was observed in epithelial cells. Chromosome amplification mainly occurred in chromosomes 7, 8, 9, 12, 13, 16, 19, 20, and 21, and deletions were most prevalent in chromosomes 4, 6, 8, 12, 14, 15, 17, 18, 19, and 22 (Figure 3(a)). The MEC subpopulation was screened using a median CNV score > 1,000 as the threshold. Notably, the CNV scores for epithelial cell subtypes 1 through 5 were more significant than epithelial cell subtype 6 (Figure 3(b) and Supplementary Table S2), suggesting a higher degree of malignancy of CRC lesions associated with these cell subsets. Therefore, we defined these as MEC subsets, and, using |log2(*fold* *change*)| > 0.5 and FDR < 0.01 as thresholds, we identified 1,259 marker genes (Supplementary Table S3), and we speculated that 1259 MEC marker genes function differently in CRC than in normal colonic epithelial cells and therefore require further analysis.

### 3.3. GO and KEGG Enrichment Analyses of MEC Subset Marker Genes

**Figure 4:**
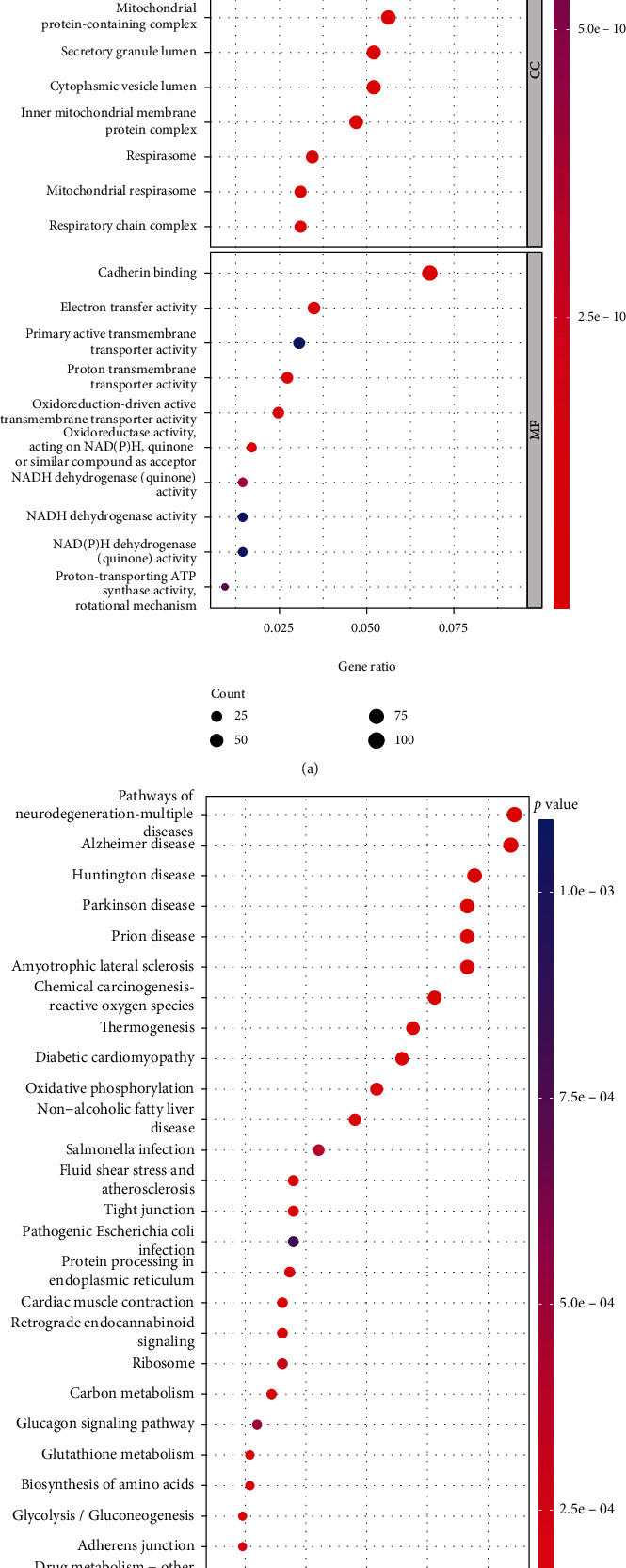
Functional enrichment analysis of marker genes in the MEC subsets. Results of GO function (a) and KEGG pathway (b) enrichment analyses.

We performed GO function (Figure 4(a)) and KEGG pathway (Figure 4(b)) enrichment analyses of the 1,259 marker genes in the five MEC subsets to determine their possible biological functions. We found that the differentially expressed genes in the MEC subsets were mainly involved in processes associated with ATP metabolism, focal adhesion, formation of cell–substrate junctions, cadherin binding, formation of adherens junctions, reactive oxygen species (ROS), and thyroid cancer. Notably, cell–substrate junction formation, calmodulin binding, and adhesion junctions are closely related to the characteristics of epithelial cells themselves, ROS is strongly associated with tumor progression, and as thyroid cancer and CRC are both epithelial-derived malignancies, we hypothesised that common epithelial cell-related genes might be involved in the development of both. These results tentatively suggest that marker genes of the MEC subpopulation are involved in the occurrence and progression of CRC mainly through the above-mentioned biological mechanisms.

### 3.4. WGCNA of MEC Marker Genes

**Figure 5:**
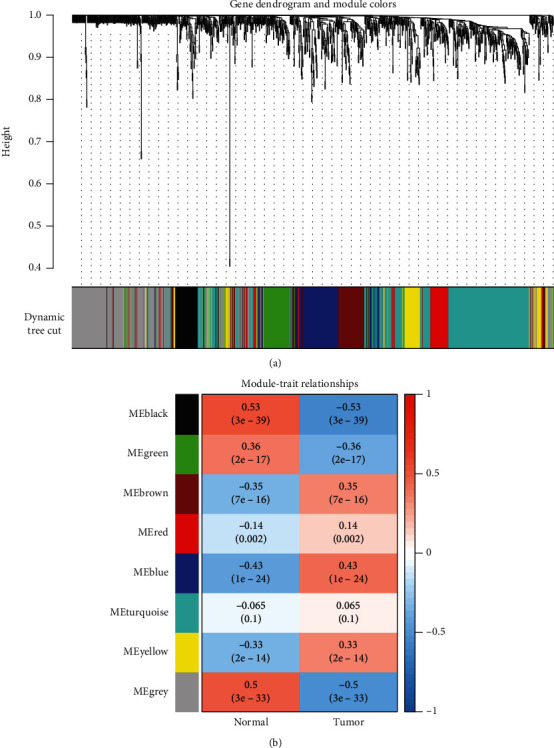
MEC marker genes associated with CRC. (a) Dendrogram of MEC subset marker genes obtained by WGCNA according to colour. (b) Correlations between characteristic genes of different modules and CRC.

Using the TCGA-CRC cohort, we performed WGCNA of the expression profiles of the identified 1259 marker genes. We clustered the MEC genes into modules associated with clinical traits (“tumour” and “normal”) based on a soft threshold of *β* = 7. As shown in Figure 5, to prevent meaningful MEC marker genes from being missed, with the exception of the turquoise module (*P* > 0.05), all genes in the remaining modules were significantly associated with clinical characteristics (“tumour” and “normal”) (*P* < 0.05) and were used in subsequent analyses. To build a clinical prediction model by linking to the clinical characteristics of CRC patients, we initially identified 787 genes significantly associated with clinical characteristics (“tumour” and “normal”) and further identified MEC marker genes associated with survival prognosis.

### 3.5. Establishment and Validation of a MECRG Signature for Predicting CRC Patient Survival

**Figure 6:**
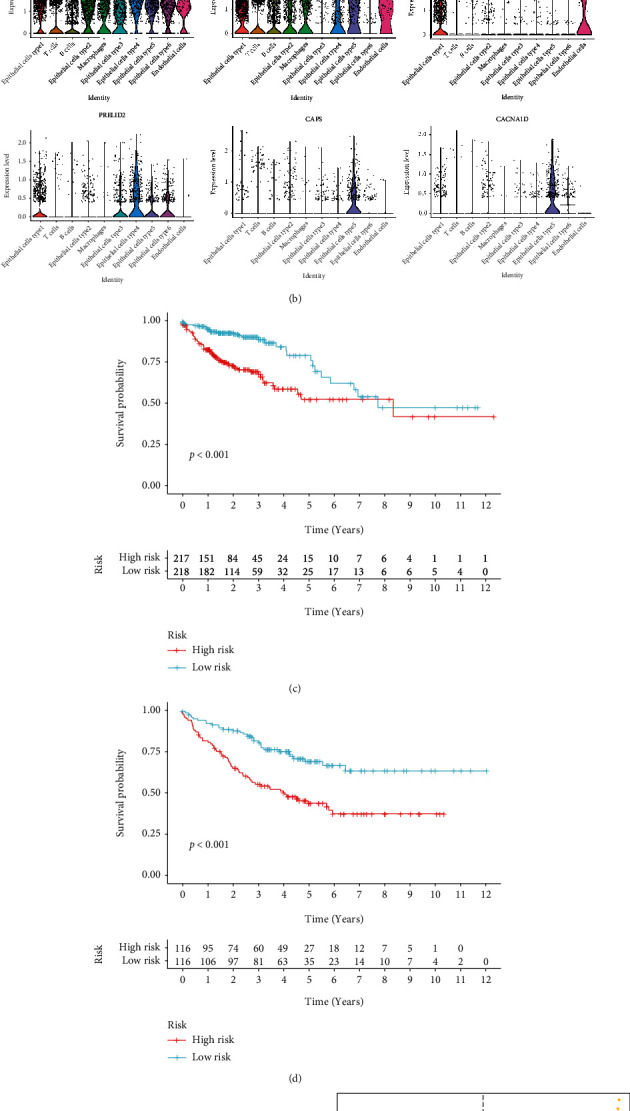
Identification and validation of MECRGs in the TCGA-CRC training and GSE17538 validation cohorts. (a) Univariate Cox analysis was used to screen MECRGs with prognostic significance. (b) Distribution of MECRGs in cell subsets. (c and d) Kaplan–Meier survival curve showing the prognostic value of the MECRG signature in the training cohort (c) and the validation cohort (d). (e and f) Distribution of the MECRG risk scores and survival status of CRC patients in the training cohort (e) and validation cohort (f). ROC curve representing the efficiency of the MECRG signature in predicting 1-, 3-, and 5-year OS in CRC patients in the training cohort (g) and validation cohort (h).

The univariate Cox analysis (Figure 6(a)) and LASSO regression analysis (Supplementary Figure S7) of the TCGA-CRC training cohort identified 47 MEC marker genes capable of predicting OS, the regression coefficients of the nine MECRGs were calculated using the “predict” function, and nine MECRGs were identified based on the minimum *λ* (*λ* = 0.01970): galectin 2 (*LGALS2*), glycerophosphodiester phosphodiesterase 1 (*GDE1*), monocyte chemoattractant protein 1 (*MPC1*), bone marrow stromal cell antigen 2 (*BST2*), tropomyosin 2 (*TPM2*), PRELI domain-containing 2 (*PRELID2*), G protein subunit gamma 5 (*GNG5*), calcyphosin (*CAPS*), and calcium voltage-gated channel subunit alpha 1D (*CACNA1D*). Validation using the GSE17538 data identified *BST2*, *TPM2*, and *CAPS* as risk genes (hazard ratio (HR) > 1) and *LGALS2*, *GDE1*, *MPC1*, *GNG5*, *PRELID2*, and *CACNA1D* as protective genes (HR < 1). The MECRG risk score was defined as follows: (–0.13614∗*expression* of *LGALS*2) + (–0.60881∗*expression* of *GDE*1) + (–0.49609∗*expression* of *MPC*1) + (–0.20530∗*expression* of *GNG*5) + (0.05613∗*expression* of *BST*2) + (0.12894∗*expression* of *TPM*2) + (–0.16624∗*expression* of *PRELID*2) + (0.48635∗*expression* of *CAPS*) + (–0.83941∗*expression* of *CACNA*1*D*). The final risk scores for all CRC samples in the training and validation cohorts are shown in Supplementary Tables S4 and S5. The MECRGs were predominantly distributed in the epithelial cell subsets (Figure 6(b)).

Next, we divided the patients into high- and low-risk groups according to the median MECRG risk score. The Kaplan–Meier survival analysis demonstrated that in the TCGA-CRC training and GSE17538 validation cohorts, the survival time of the high-risk group was shorter than that of the low-risk group (*P* < 0.001) (Figures 6(c) and 6(d)). In both cohorts, an increase in the risk score was accompanied by an increase in patient mortality (Figures 6(e) and 6(f)). The area under the ROC curve (AUC) at 1, 3, and 5 years was 0.746, 0.726, and 0.710, respectively, in the training cohort, and 0.618, 0.668, and 0.658, respectively, in the validation cohort (Figures 6(g) and 6(h)). These data indicated that the MECRG risk score shows good sensitivity and specificity for predicting the prognosis of CRC patients.

### 3.6. Independent Prognostic Value of the MECRG Risk Score

**Figure 7:**
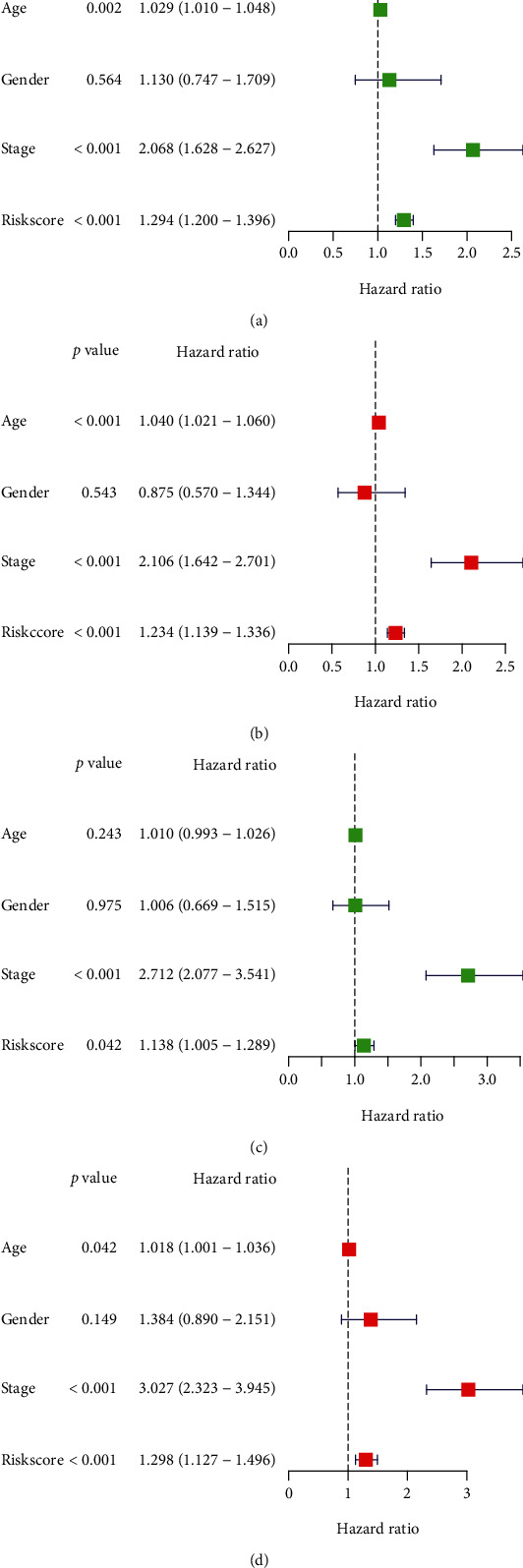
Independent prognostic value of the MECRG signature. Univariate and multivariate Cox analyses of the MECRG risk score in the TCGA-CRC training cohort (a and b) and GSE17538 validation cohort (c and d).

To determine whether the MECRG risk score can predict prognosis independently of traditional clinical features, such as age, sex, and TNM stage, we performed univariate and multivariate Cox regression analyses. In the training cohort, age (HR = 1.029 and 1.040, 95% confidence interval (CI): 1.010–1.048 and 1.021–1.060, respectively; *P* = 0.002 and *P* < 0.001), TNM stage (HR = 2.068 and 2.106, 95% CI: 1.628–2.627 and 1.642–2.701, respectively; *P* < 0.001), and the MECRG risk score (HR = 1.294 and 1.234, 95% CI: 1.200–1.396 and 1.139–1.336, respectively; *P* < 0.001) were independent predictors of OS. Analysis of the GSE17538 validation cohort confirmed that TNM stage (HR = 2.712 and 3.027, 95% CI: 2.077–3.541 and 2.323–3.945, respectively; *P* < 0.001) and the MECRG risk score (HR = 1.138 and 1.298, 95% CI: 1.005–1.289 and 1.127–1.496, respectively; *P* = 0.042 and *P* < 0.001) were independent predictors of OS (Figure 7). The above results indicated that the MECRG risk score was an independent prognostic factor in both the training and validation sets.

### 3.7. Correlation between MECRG Risk Groups and Clinical Features

**Figure 8:**
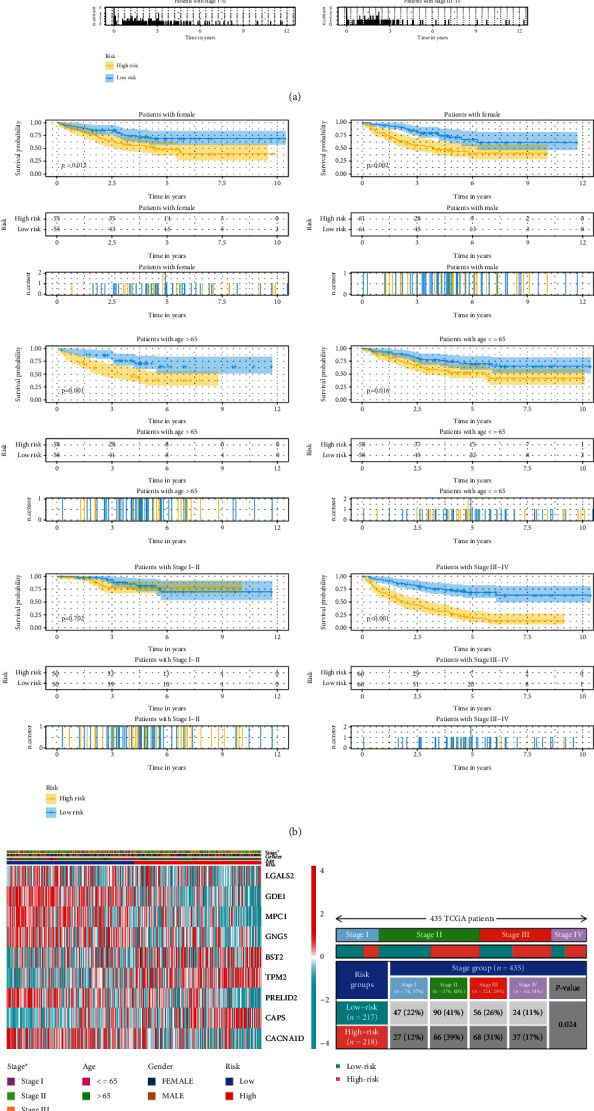
Analysis of the relationships between the MECRG signature and clinical features using the TCGA-CRC training and GSE17538 validation cohorts. (a) Survival analysis of the MECRG signature in clinical features based on the training cohort. (b) Survival analysis of the MECRG signature in clinical features based on the validation cohort. (c and d) Heat maps showing the correlation between MECRG risk grouping and TNM stage in the training cohort (c) and validation cohort (d). ^∗∗∗^*P* < 0.001, ^∗∗^*P* < 0.01, and ^∗^*P* < 0.05.

We evaluated the correlation between clinical traits and risk groups among the 435 patients in the TCGA-CRC training cohort and the 232 CRC patients in the GSE17538 validation cohort (with complete clinical information). The patients were grouped according to sex (male or female), age (≤65 or >65 years), and TNM stage (I–II or III–IV), and analyses were performed using the log-rank test and Kaplan–Meier analysis. We found that among female and male patients (*P* < 0.05), patients aged >65 years and ≤65 years (*P* < 0.05), and patients at TNM stage III–IV (*P* ≤ 0.001), those in the high-risk group had a shorter survival time than those in the low-risk group (Figures 8(a) and 8(b)). A heat map revealed significant differences in tumour staging between the high- and low-risk groups (TCGA-CRC: *P* = 0.024; GSE17538: *P* = 0.02) (Figures 8(c) and 8(d)). These results suggested that the MECRG risk score can significantly affect the survival prognosis of CRC patients across gender, age, and stage.

### 3.8. Functional Enrichment Analysis of the MECRG Signature

**Figure 9:**
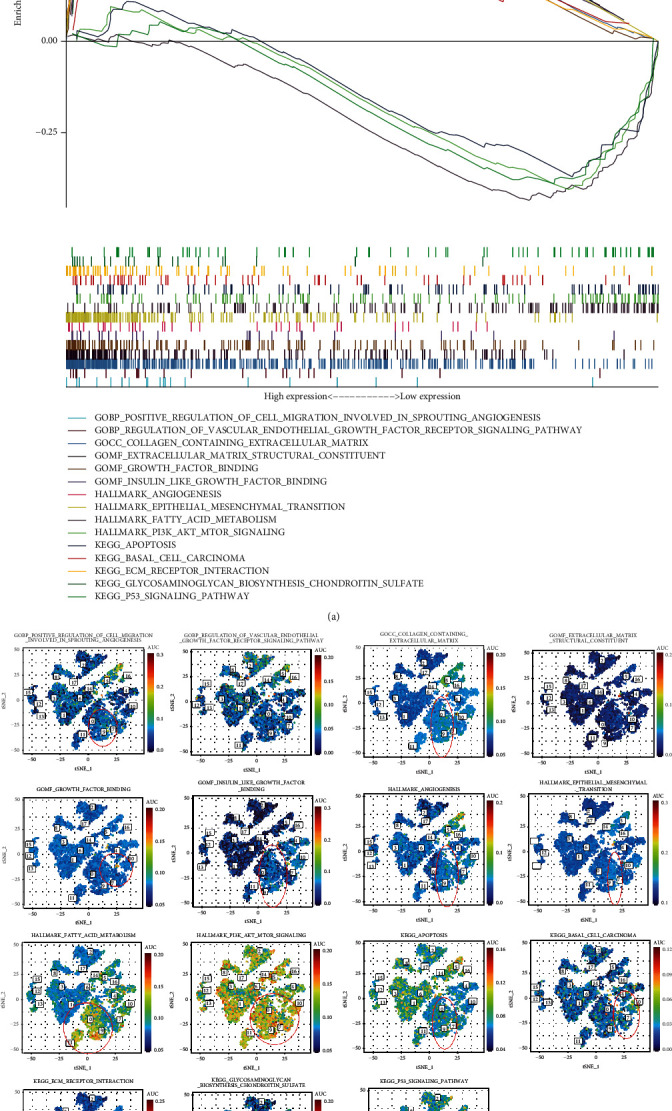
Functional enrichment analysis of the MECRG signature and distribution of enriched pathways in the cell subsets. (a) GSEA of the MECRG high- and low-risk groups. (b) Pathways enriched in the cell subsets of the MECRG risk groups.

We used GSEA to evaluate the functions of and signalling pathways associated with the genes in the high- and low-risk groups (Figure 9(a)). The results showed that the high-risk group was significantly enriched in functions related to the positive regulation of migration involved in sprouting angiogenesis, the phosphoinositide 3-kinase (PI3K)-AKT-mammalian target of rapamycin (mTOR) signalling pathway, basal cell carcinoma, extracellular matrix (ECM) receptor interaction, and angiogenesis, whereas the low-risk group was significantly enriched in apoptosis and the p53 signalling pathway. Additionally, enrichment analysis of the epithelial cell subsets indicated significant enrichment of activities involving collagen-containing ECM, fatty acid metabolism, basal cell carcinoma, PI3K-AKT signalling, and p53 signalling (Figure 9(b)). We found that the above signalling pathways are more likely to mediate the process of tumor progression in CRC epithelial cells than in other cell subpopulations.

### 3.9. Nomogram for Predicting the Prognosis of CRC Patients

**Figure 10:**
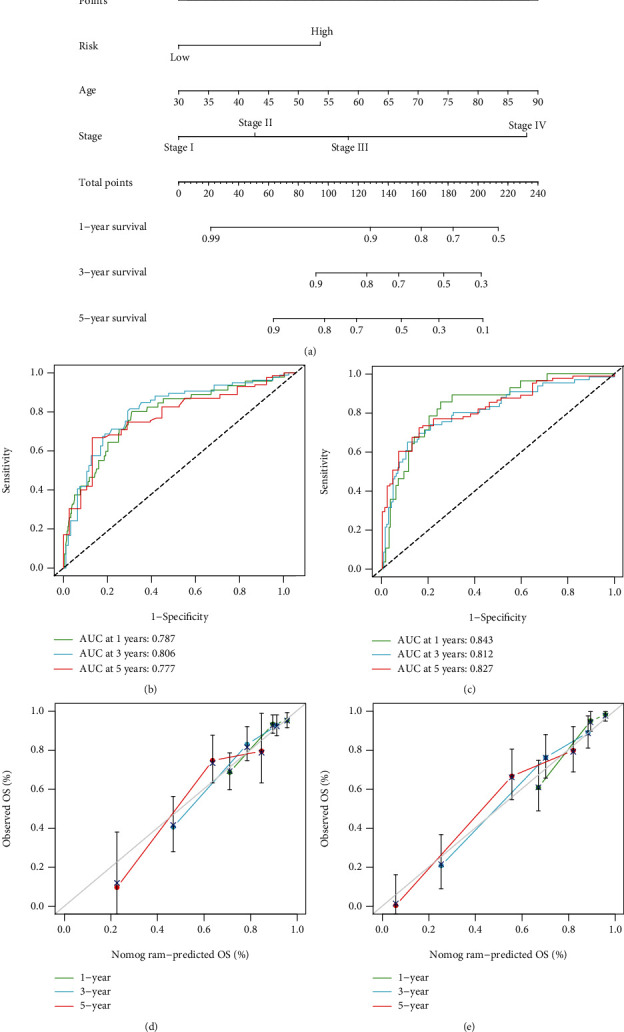
Construction of a nomogram based on the MECRG signature. ROC curve analysis of the nomogram (a) using the TCGA-CRC training cohort (b) and the GSE17538 validation cohort (c). (d and e) Calibration curves for the nomogram using the training cohort (d) and the validation cohort (e).

To establish a practical method for predicting the probability of CRC patient survival, we constructed a MECRG nomogram using the TCGA-CRC training cohort to predict OS at 1, 3, and 5 years (Figure 10(a)). The predictors included three clinical features (age, sex, and TNM stage) and the risk score. ROC curve analysis of nomogram reliability revealed AUC values at 1, 3, and 5 years of 0.787, 0.806, and 0.777, respectively (Figure 10(b)). Analysis of the GSE17538 validation cohort yielded AUC values of 0.843, 0.812, and 0.827, respectively (Figure 10(c)). The calibration curve showed that the predicted survival rate agreed well with the actual survival rate (Figures 10(d) and 10(e)). Our results demonstrated that the MECRG nomogram constructed in combination with the clinical characteristics of CRC can provide good prediction.

## 4. Discussion

Epithelial adenocarcinoma represents the most prevalent type of CRC and arises from benign colon adenoma [33]. Studies have shown that in epithelial malignant tumours, unstable adhesion between epithelial cells strongly correlates with an increased invasiveness of tumour cells. Recently, numerous mechanisms underlying cell–cell junctions regulated by E-cadherin expression in epithelial cells have been discovered [34–36]. Apical-basal polarity is the main characteristic of epithelial cells. Epithelial cell-associated polarity proteins are associated with the origin and poor prognosis of colorectal tumours, hepatocellular carcinoma, and endometrial cancer [37–39]. Therapeutic targets based on epithelial cell apical-basal polarity complexes have been reported. For example, partitioning-defective 6 (Par6) is expected to be a therapeutic target for breast cancer [37], and atypical protein kinase C (aPKC) has been suggested as a possible therapeutic target for gastric cancer [40]. Based on the above findings, we believed that the discovery of CRC epithelial cell-related biomarkers would facilitate the development of new therapeutic and predictive targets for CRC prognosis. ScRNA-seq technology allows the sequencing of RNA from individual cells to systematically track the dynamic changes in individual cells and deepen the understanding of cellular states and gene expression regulation in pathological disease processes [41]. scRNA-seq analysis provides analytical detail on the cellular level [42]. The technology has been recently applied to study epithelial cells in serous epithelial ovarian cancer and endometrial cancer [43, 44]. In nontumour research, scRNA-seq has been used for gene expression profiling of breast epithelial cells during their development [45]. Xu et al. revealed the biological mechanisms implicated in the involvement of lung epithelial cells in idiopathic fibrosis using scRNA-seq [46]. Additionally, scRNA-seq technology has received increasing attention for predicting the prognosis of cancer patients. For example, Wang et al. constructed a model based on 10 biomarkers of pancreatic ductal epithelial cells to predict the prognosis of patients with pancreatic adenocarcinoma [47], Zheng et al. [48] screened nine fibroblast marker genes from scRNA-seq CRC data as potential prognostic markers, and Li et al. [49] identified seven macrophage marker genes from breast cancer scRNA-seq data as promising diagnostic and prognostic biomarkers.

In our study, we constructed a CRC prognosis model by analysing scRNA-seq data from CRC epithelial cells. Specifically, we analysed scRNA-seq data of 15,465 cells from four CRC patients, followed by PCA for unsupervised clustering and the identification of MEC subsets based on chromosome CNV analysis. We then used WGCNA, univariate Cox, and LASSO regression analyses to construct a MECRG signature of nine genes related to CRC prognosis. We validated the MECRG signature using both training and validation cohorts. The Kaplan–Meier analysis confirmed a significantly shortened OS for CRC patients with a high MECRG risk score. Furthermore, the univariate and multivariate Cox regression analyses showed that the MECRG risk score may be an independent predictor of OS. Based on the MECRG risk score coupled with the clinical characteristics of sex, age, and TNM stage (III–IV), low-risk patients showed significantly longer survival times than high-risk patients. It is worth noting that there were significant differences between the MECRG signature-based risk groups at different CRC stages. Furthermore, the MECRG nomogram showed excellent prediction in both the training and validation sets, suggesting that it may efficiently predict CRC patient survival in the clinical setting.

Elevated expression of *LGALS2* reportedly inhibits the development of CRC and lymph node metastasis of gastric cancer [50, 51]. *GDE1* expression is significantly reduced in drug-resistant ovarian cancer samples [52]. Consistent with these findings, our results suggested that high expression of *LGALS2* and *GDE1* in CRC patients implies a good prognosis. However, the relationship between *GDE1* and CRC requires further study. Lysine demethylase 5A (KDM5A) regulates *MPC1* expression, and the KDM5A–MPC1 axis is involved in regulating the mesenchymal characteristics of cancer cells during EMT [53]. Schell et al. [54] found that loss of *MPC1* expression enhances the Warburg effect and promotes the proliferation of CRC cells. Deletion of *MPC1* is related to a poor prognosis in glioblastoma [55]. Furthermore, *BST2* activates the nuclear factor-*κ*B-Snail-Raf kinase inhibitor protein axis to promote tumour invasion and metastasis via EMT [56, 57]. *BST2* overexpression correlates with poor prognosis in CRC, stomach cancer, and oesophageal cancer [58]. *BST2* expression is specifically upregulated in oral squamous cell carcinoma and is responsible for drug resistance [59]. *BST2* is more highly expressed in breast cancer cells derived from patients presenting bone metastasis than in human primary breast cancer cells [60]. Furthermore, Mukai et al. [58] showed that *BST2* knockout *in vitro* inhibited the proliferation of gastric cancer cells. Together, these findings suggest that *MPC1* is a protective gene and *BST2* a risk gene in CRC, which is consistent with the results of the present study. *CAPS* is a calcium-binding protein related to cell proliferation and differentiation signals [61] and associated with poor prognosis in CRC and gliomas and drug resistance in breast cancer [62–64], which is consistent with our results. Abnormal expression of *TPM2* is involved in actin cytoskeleton remodelling during EMT of lens epithelial cells [65]. Shibata et al. [66] showed that downregulation of *TPM2* expression decreased EMT in injured mouse lens epithelium, resulting in delayed lens wound healing. Additionally, *TPM2* is upregulated in ovarian cancer, liver cancer, and breast cancer [67–69]. Zhou et al. [70] found that elevated *TPM2* expression in CRC patients was predictive of poor prognosis, which is in line with the findings of the present study. However, Ma et al. [71] showed that *TPM2* expression was downregulated in CRC; therefore, the precise mechanism of *TPM2* in CRC requires clarification. Patients with glioma exhibiting elevated *GNG5* expression have a shorter survival time [72], and patients with head and neck squamous cell carcinoma and elevated *PRELID2* expression have a poor prognosis [73]. Tan et al. [74] reported that excessive aldosterone secretion from aldosteronoma is related to a *CACNA1D* mutation. We identified *GNG5*, *PRELID2*, and *CACNA1D* as protective genes in CRC patients; however, further basic experimental studies of these three genes in CRC are needed. Additionally, we showed that *MPC1*, *BST2*, and *TPM2* are closely related to EMT, suggesting that these molecules are potentially important EMT-related therapeutic targets.

GSEA suggested that genes related to collagen-containing ECM, fatty acid metabolism, PI3K-AKT signalling, p53 signalling, EMT, and other related pathways were enriched in epithelial cell subsets. Among these, EMT-related pathways were more significantly enriched in epithelial cell clusters 7, 9, and 10. Activation of the PI3K-AKT signalling pathway is a key feature of the EMT programme during tumour progression [75]. The expression of genes involved in fatty acid synthesis is upregulated in CRC epithelial cells, where the accumulation of polyunsaturated fatty acids contributes to the development of CRC [76]. Targeting fatty acid synthesis genes may become a new strategy for the treatment of CRC in the future. ECM remodelling can affect the signalling in the tumour microenvironment [77]. The P53 signalling pathway is a common tumour suppressor pathway [78], and we speculate that epithelial cells may promote CRC progression by interfering with this pathway. Thus, in the CRC microenvironment, epithelial cells may mediate the development of CRC through collagen-containing ECM, fatty acid metabolism, PI3K-AKT signalling, p53 signalling, EMT, and other pathological mechanisms.

This was a retrospective study that used scRNA-seq data and bulk data from public databases to construct a model for predicting survival in CRC patients. However, this study had some limitations. First, the mechanisms of *GDE1*, *PRELID2*, *GNG5*, and *CACNA1D* in CRC have not been clarified; therefore, the data suggesting their prognostic value need to be validated. Second, we did not evaluate tumour size, metastasis, surgery, postoperative chemoradiotherapy, and other prognostic factors in this study. This may have affected the predictive accuracy of the model. In future studies, we will include additional data to increase the accuracy of the model.

## 5. Conclusion

We evaluated epithelial cell marker genes with prognostic significance in CRC using scRNA-seq data and bulk data and generated a MECRG signature and risk score, which was confirmed to show independent prognostic efficacy for CRC patients. A nomogram based on the MECRG signature along with specific clinical features demonstrated accurate prediction of CRC patient survival, suggesting its potential utility for clinical application.

## Data Availability

The datasets generated during and/or analysed in this study are available from the corresponding author upon reasonable request.

## Supplementary Materials

Supplementary 1 Figure S1: quality control of the scRNA-seq data from the four CRC samples. Figure S2: gene numbers in the four CRC samples are positively correlated with sequencing depth. Figure S3: PCA classification of the cells based on the scRNA-seq data. Figure S4: eleven statistically significant principal components as identified by PCA. Figure S5: dot diagram showing the expression of specific marker genes in the cell subsets. Figure S6: distribution of specific marker genes in the four cell subsets. Figure S7: LASSO regression was used to screen MECRGs with prognostic significance.

Supplementary 2 Table S1: clinical characteristics of the four CRC patients. Table S2: CNV scores of epithelial cell subclusters. Table S3: MEC subclusters of marker genes. Table S4: MECRG signature using the TCGA-CRC training cohort. Table S5: MECRG signature using the GSE17538 validation cohort.

## References

[1] Peng Y. N., Huang M. L., Kao C. H. (2019). Prevalence of depression and anxiety in colorectal cancer patients: a literature review. *International Journal Environmental Research and Public Health*.

[2] Sung H., Ferlay J., Siegel R. L. (2021). Global cancer statistics 2020: GLOBOCAN estimates of incidence and mortality worldwide for 36 cancers in 185 countries. *CA: a Cancer Journal for Clinicians*.

[3] Islami F., Goding Sauer A., Miller K. D. (2018). Proportion and number of cancer cases and deaths attributable to potentially modifiable risk factors in the United States. *CA: a Cancer Journal for Clinicians*.

[4] Miller K. D., Fidler-Benaoudia M., Keegan T. H., Hipp H. S., Jemal A., Siegel R. L. (2020). Cancer statistics for adolescents and young adults, 2020. *CA: a Cancer Journal for Clinicians*.

[5] Edge S. B., Compton C. C. (2010). The American Joint Committee on Cancer: the 7th edition of the AJCC cancer staging manual and the future of TNM. *Annals of Surgical Oncology*.

[6] Jemal A., Siegel R., Ward E. (2008). Cancer statistics, 2008. *CA: a Cancer Journal for Clinicians*.

[7] Karantza V. (2011). Keratins in health and cancer: more than mere epithelial cell markers. *Oncogene*.

[8] Tanimura N., Fujita Y. (2020). Epithelial defense against cancer (EDAC). *Seminars in Cancer Biology*.

[9] Lv H., Liu R., Fu J. (2014). Epithelial cell-derived periostin functions as a tumor suppressor in gastric cancer through stabilizing p53 and E-cadherin proteins via the Rb/E2F1/p14ARF/Mdm2 signaling pathway. *Cell Cycle*.

[10] McCaffrey L. M., Macara I. G. (2011). Epithelial organization, cell polarity and tumorigenesis. *Trends in Cell Biology*.

[11] Royer C., Lu X. (2011). Epithelial cell polarity: a major gatekeeper against cancer?. *Cell Death & Differentiation*.

[12] Zeng S. G., Lin X., Liu J.-C., Zhou J. (2019). Hypoxia-induced internalization of connexin 26 and connexin 43 in pulmonary epithelial cells is involved in the occurrence of non-small cell lung cancer via the P53/MDM2 signaling pathway. *International Journal of Oncology*.

[13] Varga J., Greten F. R. (2017). Cell plasticity in epithelial homeostasis and tumorigenesis. *Nature Cell Biology*.

[14] Knösel T., Emde V., Schlüns K., Schlag P. M., Dietel M., Petersen I. (2006). Cytokeratin profiles identify diagnostic signatures in colorectal cancer using multiplex analysis of tissue microarrays. *Cell Oncology*.

[15] Ausch C., Buxhofer-Ausch V., Olszewski U. (2009). Caspase-cleaved cytokeratin 18 fragment (M30) as marker of postoperative residual tumor load in colon cancer patients. *European Journal of Surgical Oncology*.

[16] Cheong C. K., Nistala K. R. Y., Ng C. H. (2020). Neoadjuvant therapy in locally advanced colon cancer: a meta-analysis and systematic review. *Journal of Gastrointestinal Oncology*.

[17] Lambrechts D., Wauters E., Boeckx B. (2018). Phenotype molding of stromal cells in the lung tumor microenvironment. *Nature Medicine*.

[18] Tang F., Barbacioru C., Wang Y. (2009). mRNA-Seq whole-transcriptome analysis of a single cell. *Nature Methods*.

[19] Azizi E., Carr A. J., Plitas G. (2018). Single-cell map of diverse immune phenotypes in the breast tumor microenvironment. *Cell*.

[20] Zhang Q., He Y., Luo N. (2019). Landscape and dynamics of single immune cells in hepatocellular carcinoma. *Cell*.

[21] Zheng X., Song J., Yu C. (2022). Single-cell transcriptomic profiling unravels the adenoma-initiation role of protein tyrosine kinases during colorectal tumorigenesis. *Signal Transduction and Targeted Therapy*.

[22] Mangiola S., Doyle M. A., Papenfuss A. T. (2021). Interfacing Seurat with the R tidy universe. *Bioinformatics*.

[23] Chen Z., Zhang H., Bai Y. (2021). Single cell transcriptomic analysis identifies novel vascular smooth muscle subsets under high hydrostatic pressure. *Science China Life Sciences*.

[24] Pan J. H., Zhou H., Cooper L. (2019). LAYN is a prognostic biomarker and correlated with immune infiltrates in gastric and colon cancers. *Frontiers in Immunology*.

[25] Lombardo G., Gili M., Grange C. (2018). IL-3R-alpha blockade inhibits tumor endothelial cell-derived extracellular vesicle (EV)-mediated vessel formation by targeting the *β*-catenin pathway. *Oncogene*.

[26] Ichimiya H., Maeda K., Enomoto A., Weng L., Takahashi M., Murohara T. (2015). Girdin/GIV regulates transendothelial permeability by controlling VE-cadherin trafficking through the small GTPase, R-Ras. *Biochemical and Biophysical Research Communications*.

[27] Gires O., Pan M., Schinke H., Canis M., Baeuerle P. A. (2020). Expression and function of epithelial cell adhesion molecule EpCAM: where are we after 40 years?. *Cancer and Metastasis Reviews*.

[28] Corso G., Figueiredo J., De Angelis S. P. (2020). E-cadherin deregulation in breast cancer. *Journal of Cellular and Molecular Medicine*.

[29] Fang L., Yu G., Yu W., Chen G., Ye B. (2021). The correlation of WDR76 expression with survival outcomes and immune infiltrates in lung adenocarcinoma. *PeerJ*.

[30] Patel A. P., Tirosh I., Trombetta J. J. (2014). Single-cell RNA-seq highlights intratumoral heterogeneity in primary glioblastoma. *Science*.

[31] Xu Q., Chen S., Hu Y., Huang W. (2021). Single-cell RNA transcriptome reveals the intra-tumoral heterogeneity and regulators underlying tumor progression in metastatic pancreatic ductal adenocarcinoma. *Cell Death Discovery*.

[32] Langfelder P., Horvath S. (2008). WGCNA: an R package for weighted correlation network analysis. *BMC Bioinformatics*.

[33] Takhar A. S., Eremin O., Watson S. A. (2004). The role of gastrin in colorectal carcinogenesis. *The Surgeon*.

[34] Bryant D. M., Stow J. L. (2004). The ins and outs of E-cadherin trafficking. *Trends in Cell Biology*.

[35] Kowalczyk A. P., Reynolds A. B. (2004). Protecting your tail: regulation of cadherin degradation by p120-catenin. *Current Opinion in Cell Biology*.

[36] Swaminathan G., Cartwright C. A. (2012). Rack1 promotes epithelial cell-cell adhesion by regulating E-cadherin endocytosis. *Oncogene*.

[37] Kamei Y., Kito K., Takeuchi T. (2007). Human scribble accumulates in colorectal neoplasia in association with an altered distribution of *β*-catenin. *Human Pathology*.

[38] Ouyang Z., Zhan W., Dan L. (2009). hScrib, a human homolog of Drosophila neoplastic tumor suppressor, is involved in the progress of endometrial cancer. *Oncology Research*.

[39] Wan S., Meyer A.-S., Weiler S. M. E. (2018). Cytoplasmic localization of the cell polarity factor scribble supports liver tumor formation and tumor cell invasiveness. *Hepatology*.

[40] Takagawa R., Akimoto K., Ichikawa Y. (2010). High expression of atypical protein kinase C lambda/iota in gastric cancer as a prognostic factor for recurrence. *Annals of Surgical Oncology*.

[41] Kolodziejczyk A. A., Kim J. K., Svensson V., Marioni J. C., Teichmann S. A. (2015). The technology and biology of single-cell RNA sequencing. *Molecular Cell*.

[42] Ren X., Kang B., Zhang Z. (2018). Understanding tumor ecosystems by single-cell sequencing: promises and limitations. *Genome Biology*.

[43] Shih A. J., Menzin A., Whyte J. (2018). Identification of grade and origin specific cell populations in serous epithelial ovarian cancer by single cell RNA-seq. *PLoS One*.

[44] Guo Y.-E., Li Y., Cai B. (2021). Phenotyping of immune and endometrial epithelial cells in endometrial carcinomas revealed by single-cell RNA sequencing. *Aging*.

[45] Bach K., Pensa S., Grzelak M. (2017). Differentiation dynamics of mammary epithelial cells revealed by single-cell RNA sequencing. *Nature Communications*.

[46] Xu Y., Mizuno T., Sridharan A. (2016). Single-cell RNA sequencing identifies diverse roles of epithelial cells in idiopathic pulmonary fibrosis. *JCI Insight*.

[47] Wang X., Dou X., Ren X. (2021). A ductal-cell-related risk model integrating single-cell and bulk sequencing data predicts the prognosis of patients with pancreatic adenocarcinoma. *Frontiers in Genetics*.

[48] Zheng H., Liu H., Ge Y., Wang X. (2021). Integrated single-cell and bulk RNA sequencing analysis identifies a cancer associated fibroblast-related signature for predicting prognosis and therapeutic responses in colorectal cancer. *Cancer Cell International*.

[49] Li Y., Zhao X., Liu Q., Liu Y. (2021). Bioinformatics reveal macrophages marker genes signature in breast cancer to predict prognosis. *Annals of Medicine*.

[50] Li H., Zhao L., Lau Y. S., Zhang C., Han R. (2021). Genome-wide CRISPR screen identifies LGALS2 as an oxidative stress-responsive gene with an inhibitory function on colon tumor growth. *Oncogene*.

[51] Jung J.-H., Kim H.-J., Yeom J. (2012). Lowered expression of galectin-2 is associated with lymph node metastasis in gastric cancer. *Journal of Gastroenterology*.

[52] Lu J., Li Y., Li Y. A. (2022). In vivo detection of dysregulated choline metabolism in paclitaxel-resistant ovarian cancers with proton magnetic resonance spectroscopy. *Journal of Translational Medicine*.

[53] You J. H., Lee J., Roh J. L. (2021). Mitochondrial pyruvate carrier 1 regulates ferroptosis in drug-tolerant persister head and neck cancer cells via epithelial-mesenchymal transition. *Cancer Letters*.

[54] Schell J. C., Olson K. A., Jiang L. (2014). A role for the mitochondrial pyruvate carrier as a repressor of the Warburg effect and colon cancer cell growth. *Molecular Cell*.

[55] Chai Y., Wang C., Liu W., Fan Y., Zhang Y. (2019). *MPC1* deletion is associated with poor prognosis and temozolomide resistance in glioblastoma. *Journal of Neurooncology*.

[56] Matsuda A., Suzuki Y., Honda G. (2003). Large-scale identification and characterization of human genes that activate NF-*κ*B and MAPK signaling pathways. *Oncogene*.

[57] Wu K., Bonavida B. (2009). The activated NF-*κ*B-Snail-RKIP circuitry in cancer regulates both the metastatic cascade and resistance to apoptosis by cytotoxic drugs. *Critical Reviews of Immunology*.

[58] Mukai S., Oue N., Oshima T. (2017). Overexpression of transmembrane protein BST2 is associated with poor survival of patients with esophageal, gastric, or colorectal cancer. *Annals of Surgical Oncology*.

[59] Jin H., Zhang L., Wang S., Qian L. (2021). BST2 promotes growth and induces gefitinib resistance in oral squamous cell carcinoma via regulating the EGFR pathway. *Archives of Medicinal Science*.

[60] Cai D., Cao J., Li Z. (2009). Up-regulation of bone marrow stromal protein 2 (BST2) in breast cancer with bone metastasis. *BMC Cancer*.

[61] Lecocq R., Lamy F., Erneux C., Dumont J. E. (1995). Rapid purification and identification of calcyphosine, a Ca^2+^-binding protein phosphorylated by protein kinase A. *Biochemistry Journal*.

[62] Shao W., Wang Q., Wang F., Jiang Y., Xu M., Xu J. (2016). Abnormal expression of calcyphosine is associated with poor prognosis and cell biology function in colorectal cancer. *Onco Targets Therapy*.

[63] Zhu Z., Wang J., Tan J. (2021). Calcyphosine promotes the proliferation of glioma cells and serves as a potential therapeutic target. *Journal of Pathology*.

[64] Johansson H. J., Sanchez B. C., Forshed J. (2015). Proteomics profiling identify CAPS as a potential predictive marker of tamoxifen resistance in estrogen receptor positive breast cancer. *Clinical Proteomics*.

[65] Kubo E., Hasanova N., Fatma N., Sasaki H., Singh D. P. (2013). Elevated tropomyosin expression is associated with epithelial-mesenchymal transition of lens epithelial cells. *Journal of Cellular and Molecular Medicine*.

[66] Shibata T., Shibata S., Ishigaki Y. (2018). Tropomyosin 2 heterozygous knockout in mice using CRISPR-cas9 system displays the inhibition of injury-induced epithelial-mesenchymal transition, and lens opacity. *Mechanisms of Ageing and Development*.

[67] Li D. Q., Wang L., Fei F. (2006). Identification of breast cancer metastasis-associated proteins in an isogenic tumor metastasis model using two-dimensional gel electrophoresis and liquid chromatography-ion trap-mass spectrometry. *Proteomics*.

[68] Tian Z., Zhao J., Wang Y. (2022). The prognostic value of TPM1–4 in hepatocellular carcinoma. *Cancer Medicine*.

[69] Tang H.-Y., Beer L. A., Tanyi J. L., Zhang R., Liu Q., Speicher D. W. (2013). Protein isoform-specific validation defines multiple chloride intracellular channel and tropomyosin isoforms as serological biomarkers of ovarian cancer. *Journal of Proteomics*.

[70] Zhou Y., Bian S., Zhou X. (2020). Single-cell multiomics sequencing reveals prevalent genomic alterations in tumor stromal cells of human colorectal cancer. *Cancer Cell*.

[71] Ma Y., Xiao T., Xu Q., Shao X., Wang H. (2016). iTRAQ-based quantitative analysis of cancer-derived secretory proteome reveals TPM2 as a potential diagnostic biomarker of colorectal cancer. *Frontiers in Medicine*.

[72] Zhang W., Liu Z., Liu B. (2021). *GNG5* is a novel oncogene associated with cell migration, proliferation, and poor prognosis in glioma. *Cancer Cell International*.

[73] Liu G., Zeng X., Wu B., Zhao J., Pan Y. (2020). RNA-seq analysis of peripheral blood mononuclear cells reveals unique transcriptional signatures associated with radiotherapy response of nasopharyngeal carcinoma and prognosis of head and neck cancer. *Cancer Biology and Therapy*.

[74] Tan G. C., Negro G., Pinggera A. (2017). Aldosterone-producing adenomas: histopathology-genotype correlation and identification of a novel *CACNA1D* mutation. *Hypertension*.

[75] Larue L., Bellacosa A. (2005). Epithelial-mesenchymal transition in development and cancer: role of phosphatidylinositol 3′ kinase/AKT pathways. *Oncogene*.

[76] Hofmanová J., Slavík J., Ciganek M. (2021). Complex alterations of fatty acid metabolism and phospholipidome uncovered in isolated colon cancer epithelial cells. *International Journal of Molecular Science*.

[77] Mohan V., Das A., Sagi I. (2020). Emerging roles of ECM remodeling processes in cancer. *Seminars in Cancer Biology*.

[78] Hernández Borrero L. J., El-Deiry W. S. (2021). Tumor suppressor p53: biology, signaling pathways, and therapeutic targeting. *Biochimica Biophysica Acta Reviews on Cancer*.

