# Supplementary figures and images for: Single-Cell RNA Sequencing Reveals the Role of Epithelial Cell Marker Genes in Predicting the Prognosis of Colorectal Cancer Patients
Source: Dis Markers. 2022 Aug 1;2022:8347125. doi: 10.1155/2022/8347125 (PMC9372514; doi:10.1155/2022/8347125)

### nFeature\_RNA

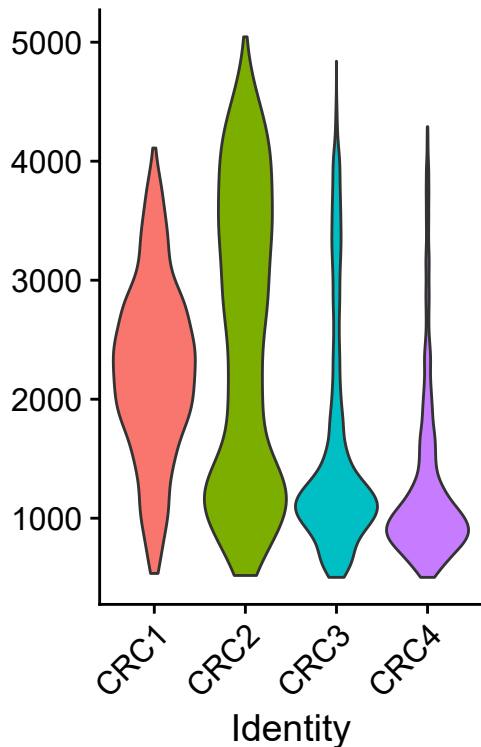

### nCount\_RNA

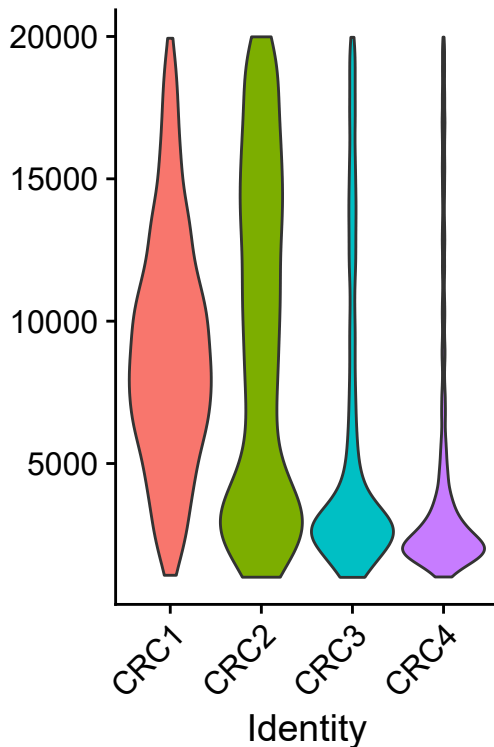

### percent.mt

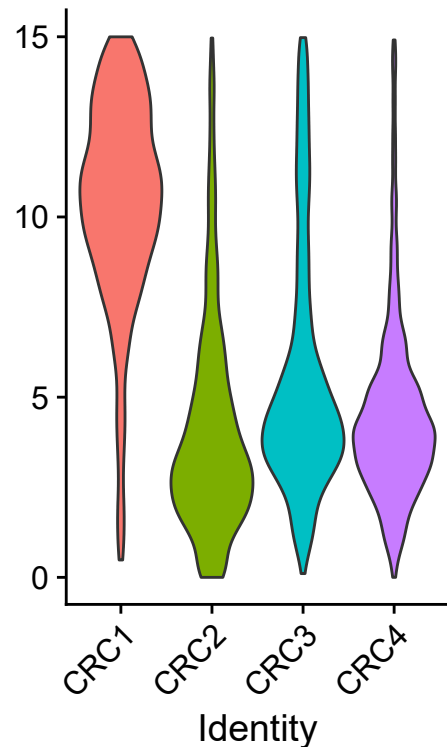

Supplement: Supplementary 1 — Figure S1: quality control of the scRNA-seq data from the four CRC samples. Figure S2: gene numbers in the four CRC samples are positively correlated with sequencing depth. Figure S3: PCA classification of the cells based on the scRNA-seq data. Figure S4: eleven statistically significant principal components as identified by PCA. Figure S5: dot diagram showing the expression of specific marker genes in the cell subsets. Figure S6: distribution of specific marker genes in the four cell subsets. Figure S7: LASSO regression was used to screen MECRGs with prognostic significance. [file 8347125.f1.zip › Figure S1.pdf]

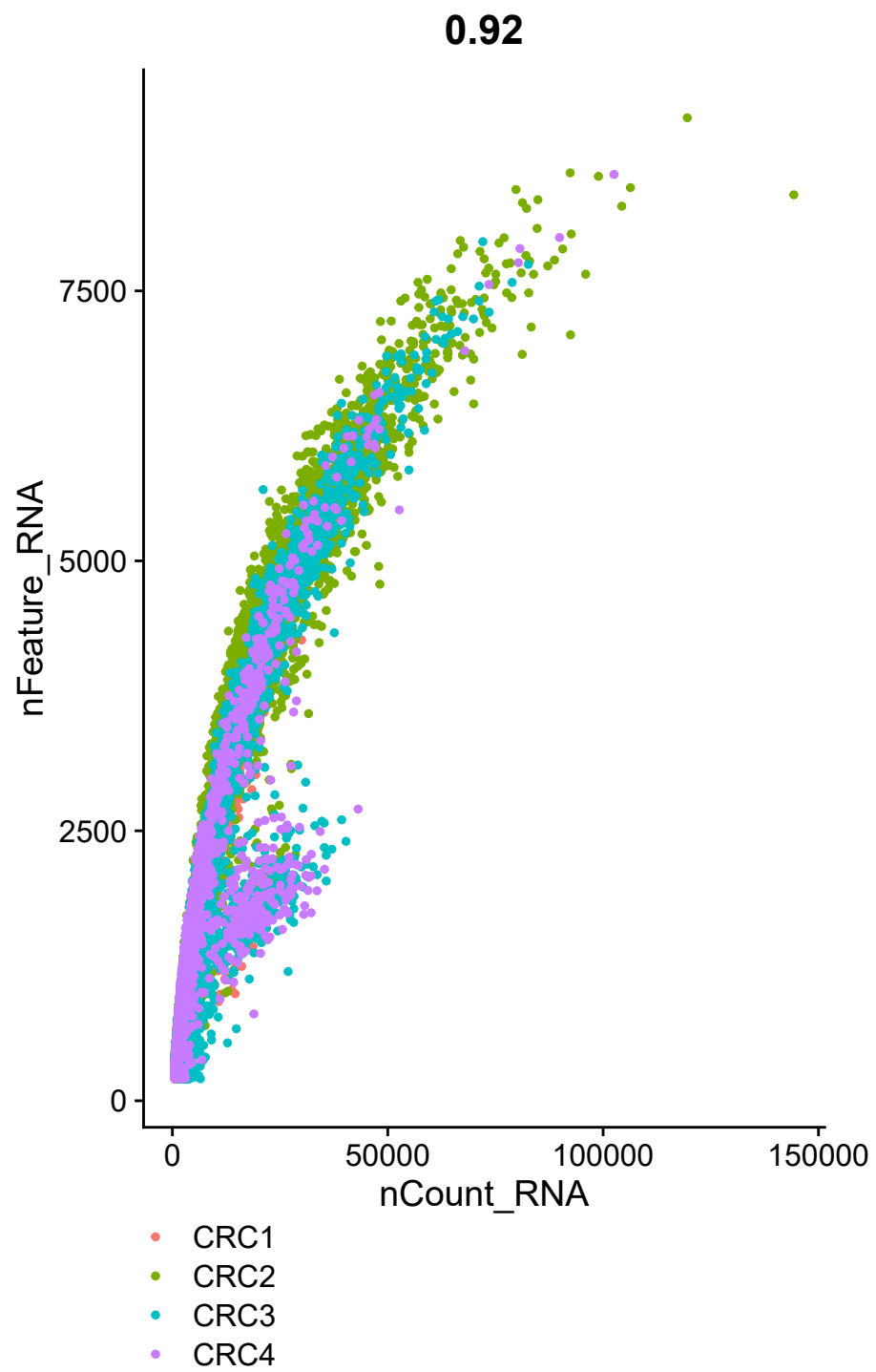

Supplement: Supplementary 1 — Figure S1: quality control of the scRNA-seq data from the four CRC samples. Figure S2: gene numbers in the four CRC samples are positively correlated with sequencing depth. Figure S3: PCA classification of the cells based on the scRNA-seq data. Figure S4: eleven statistically significant principal components as identified by PCA. Figure S5: dot diagram showing the expression of specific marker genes in the cell subsets. Figure S6: distribution of specific marker genes in the four cell subsets. Figure S7: LASSO regression was used to screen MECRGs with prognostic significance. [file 8347125.f1.zip › Figure S2.pdf]

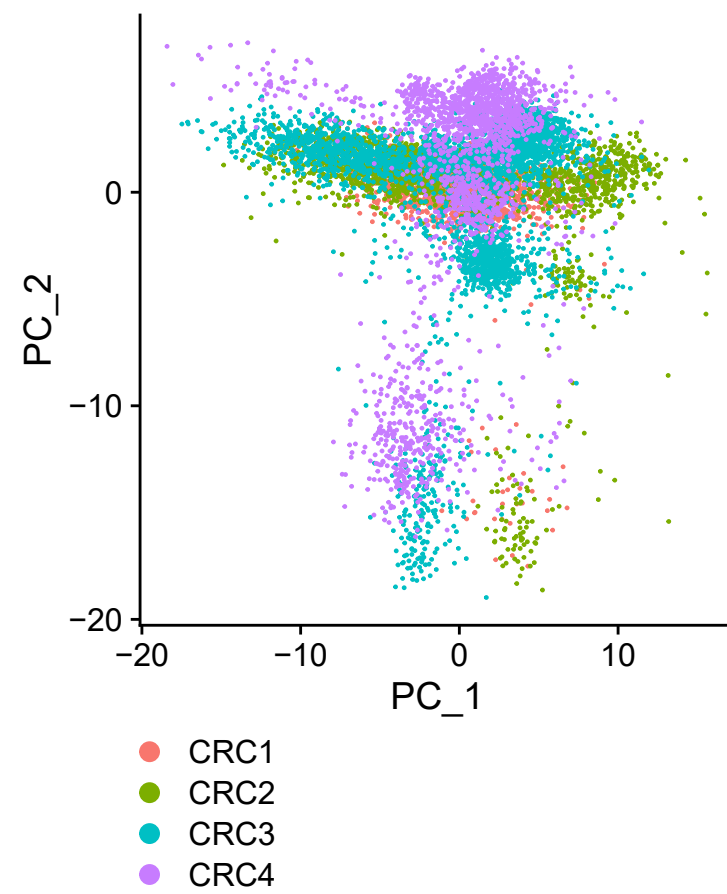

Supplement: Supplementary 1 — Figure S1: quality control of the scRNA-seq data from the four CRC samples. Figure S2: gene numbers in the four CRC samples are positively correlated with sequencing depth. Figure S3: PCA classification of the cells based on the scRNA-seq data. Figure S4: eleven statistically significant principal components as identified by PCA. Figure S5: dot diagram showing the expression of specific marker genes in the cell subsets. Figure S6: distribution of specific marker genes in the four cell subsets. Figure S7: LASSO regression was used to screen MECRGs with prognostic significance. [file 8347125.f1.zip › Figure S3.pdf]

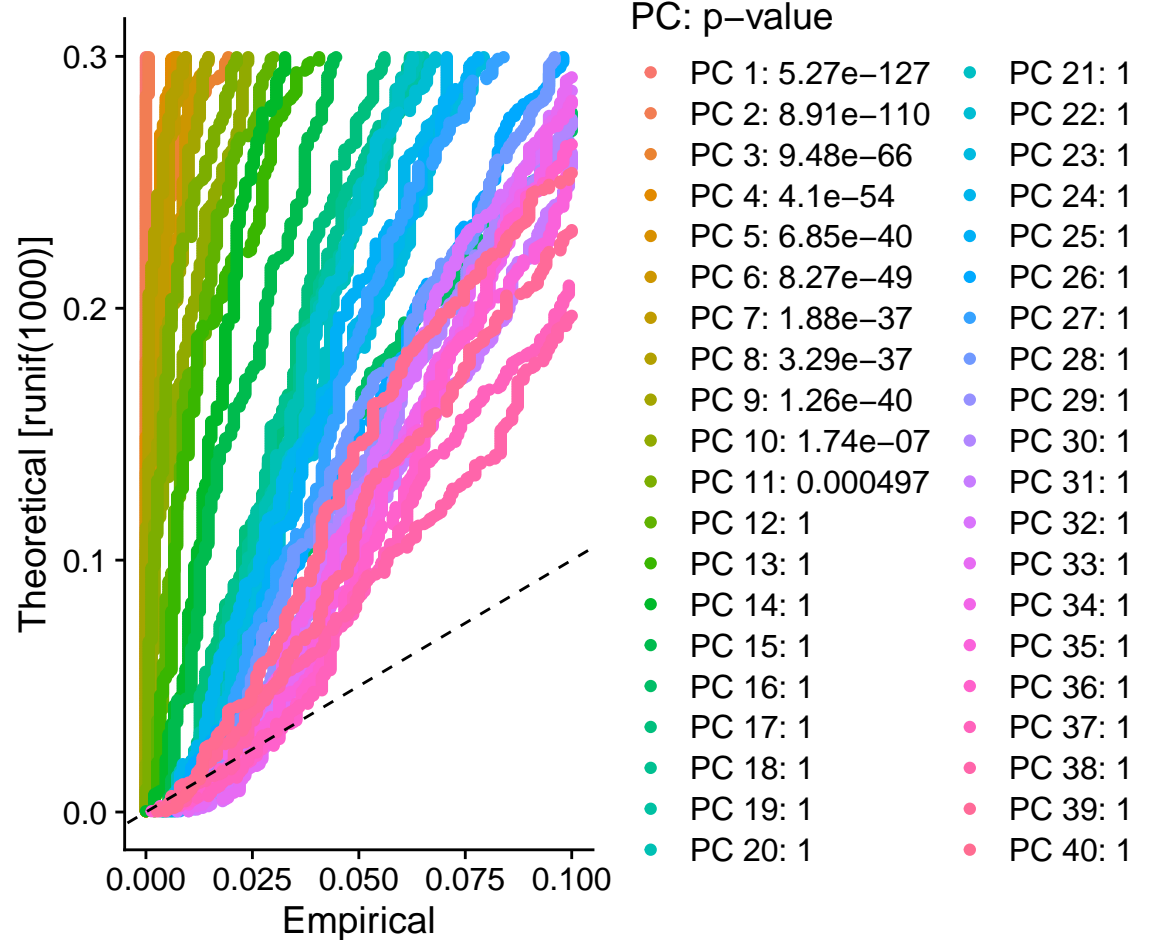

Supplement: Supplementary 1 — Figure S1: quality control of the scRNA-seq data from the four CRC samples. Figure S2: gene numbers in the four CRC samples are positively correlated with sequencing depth. Figure S3: PCA classification of the cells based on the scRNA-seq data. Figure S4: eleven statistically significant principal components as identified by PCA. Figure S5: dot diagram showing the expression of specific marker genes in the cell subsets. Figure S6: distribution of specific marker genes in the four cell subsets. Figure S7: LASSO regression was used to screen MECRGs with prognostic significance. [file 8347125.f1.zip › Figure S4.pdf]

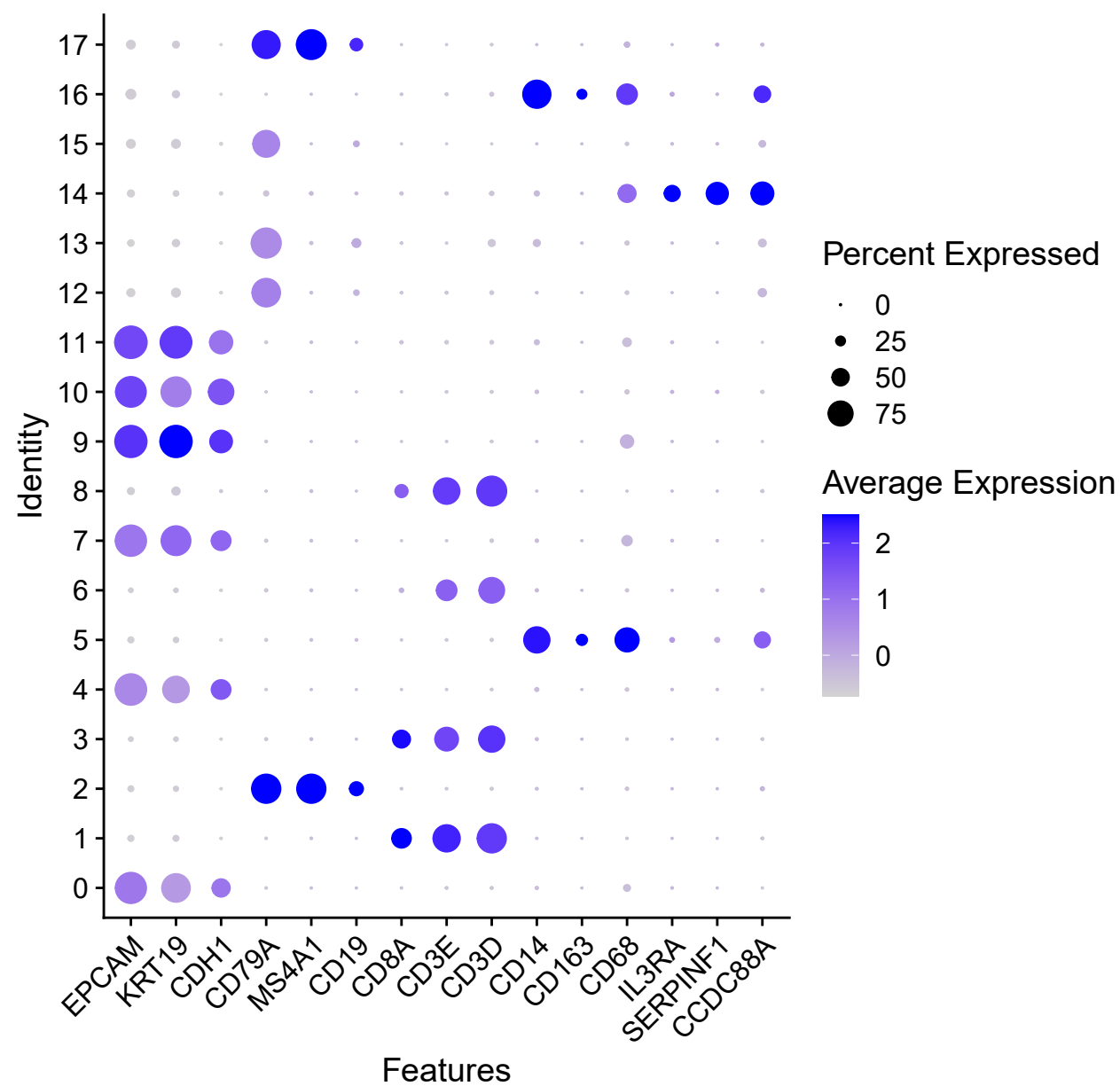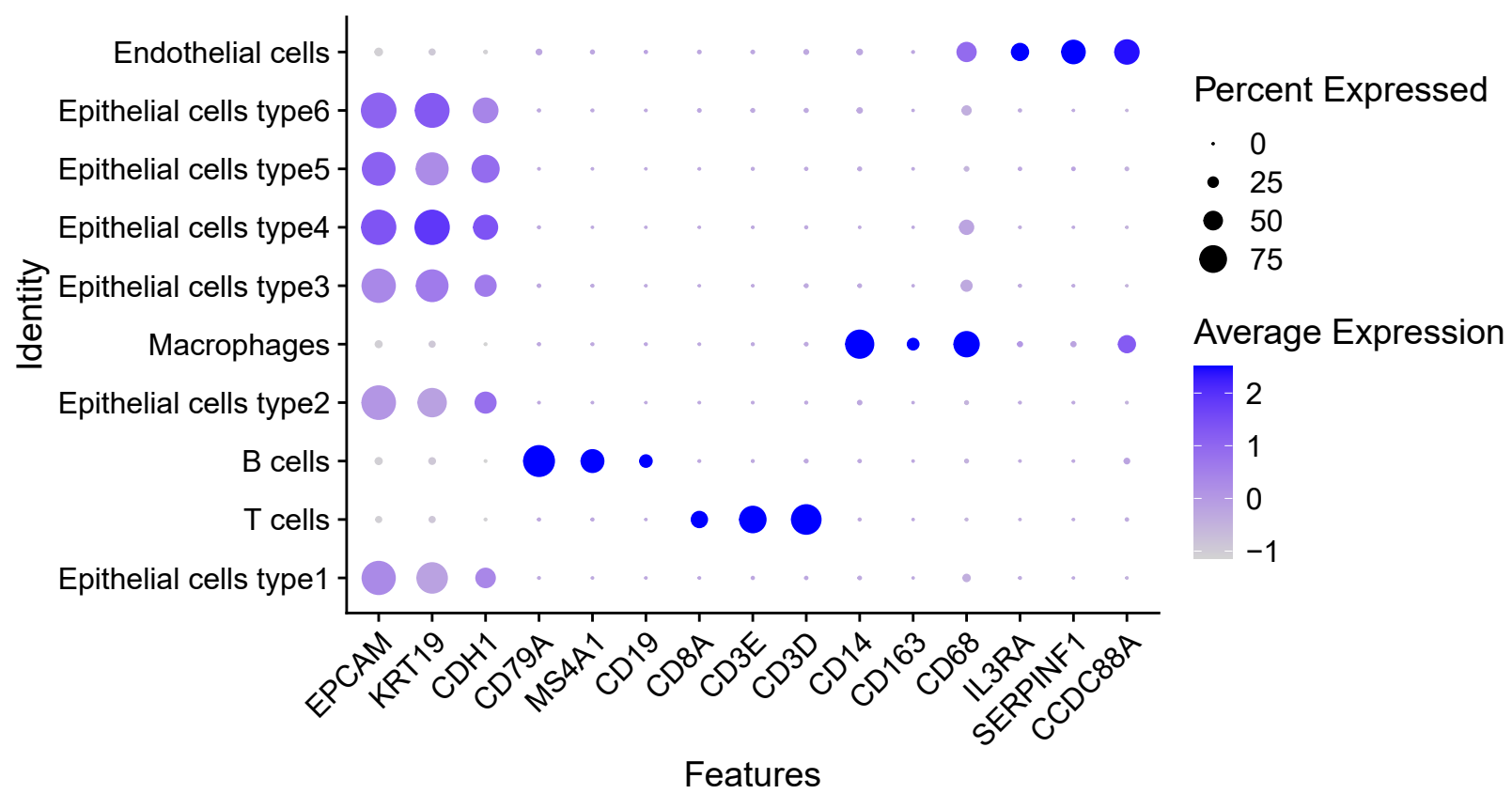

Supplement: Supplementary 1 — Figure S1: quality control of the scRNA-seq data from the four CRC samples. Figure S2: gene numbers in the four CRC samples are positively correlated with sequencing depth. Figure S3: PCA classification of the cells based on the scRNA-seq data. Figure S4: eleven statistically significant principal components as identified by PCA. Figure S5: dot diagram showing the expression of specific marker genes in the cell subsets. Figure S6: distribution of specific marker genes in the four cell subsets. Figure S7: LASSO regression was used to screen MECRGs with prognostic significance. [file 8347125.f1.zip › Figure S5.pdf]

**EPCAM**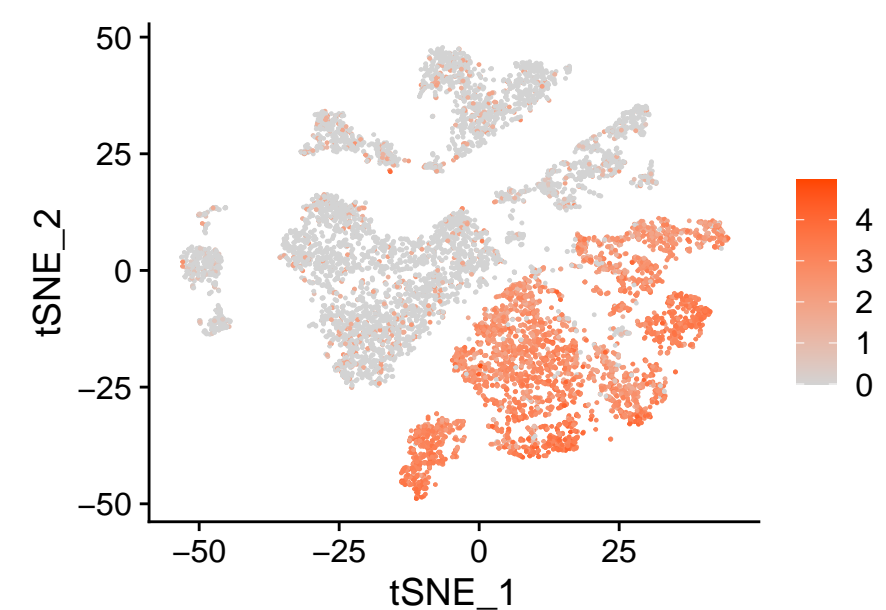**KRT19**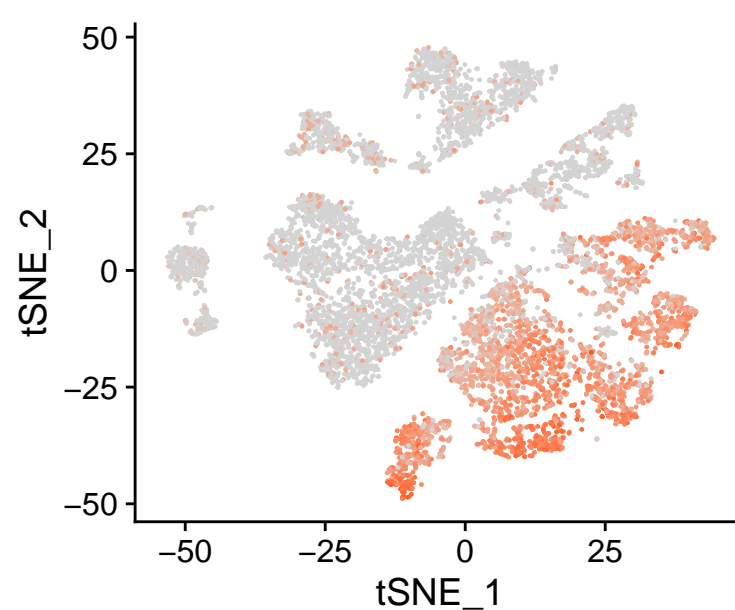**CDH1**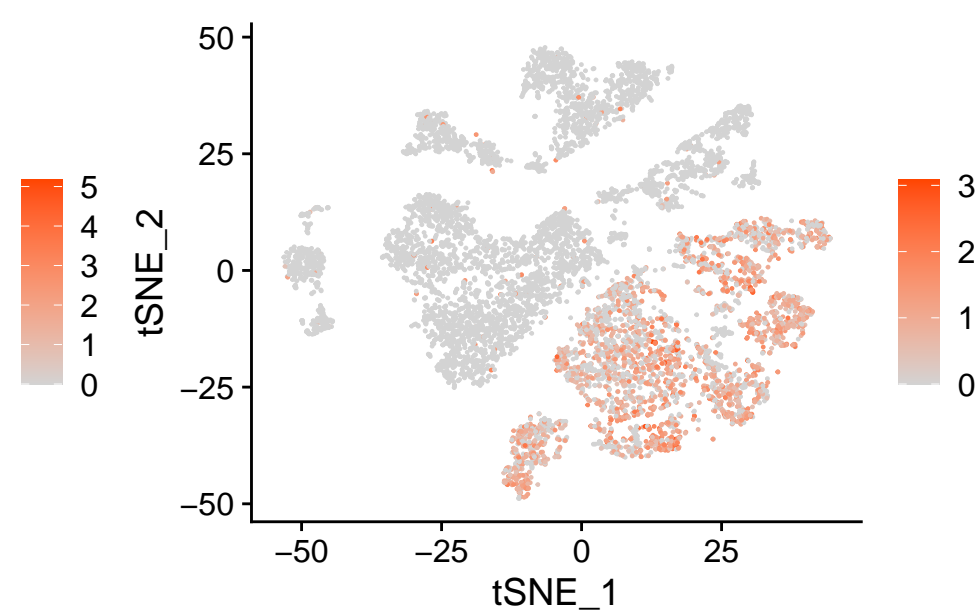**CD79A**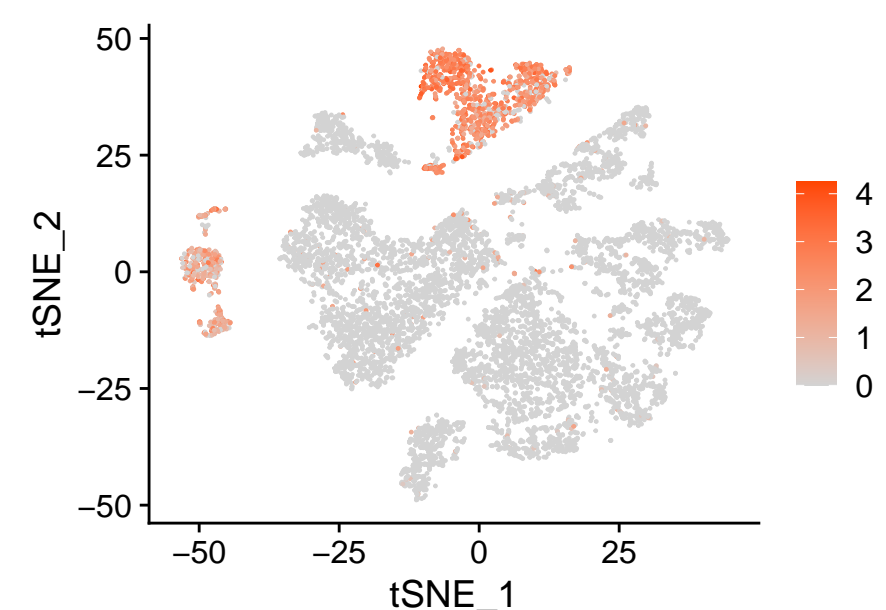**MS4A1**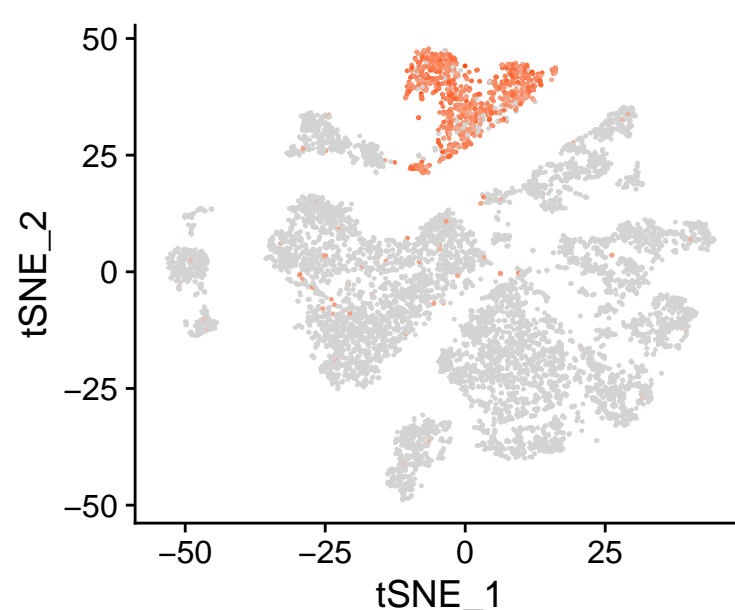**CD19**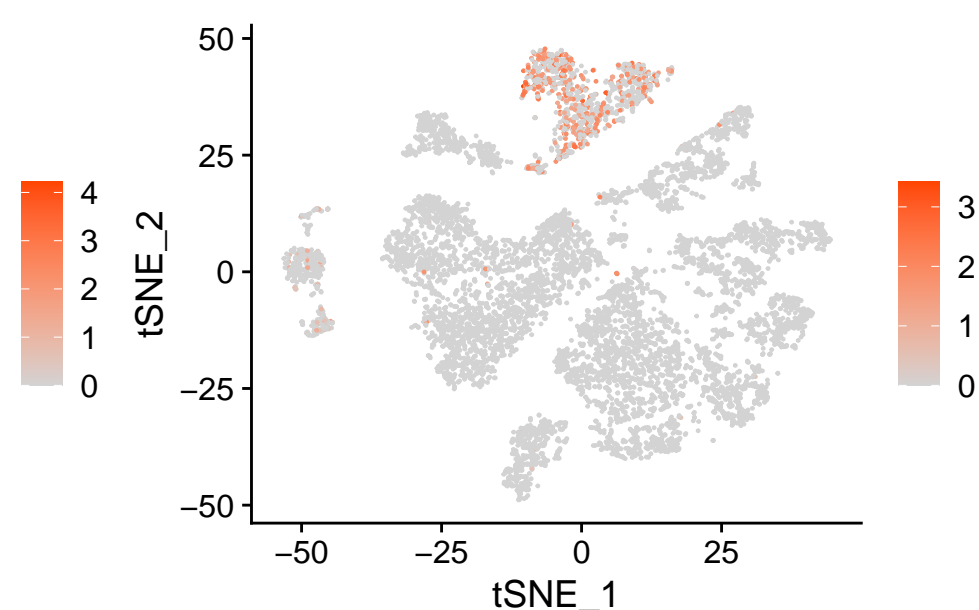**CD8A**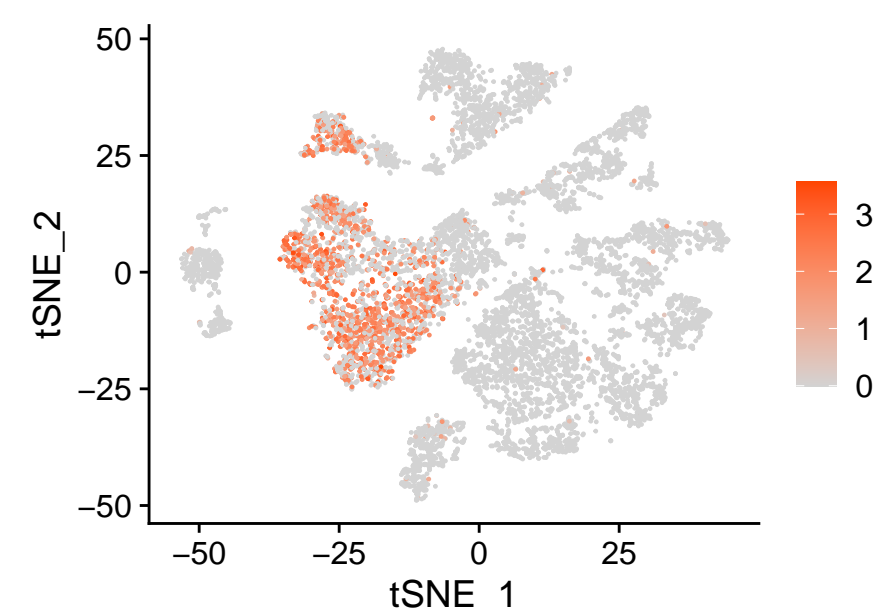**CD3E**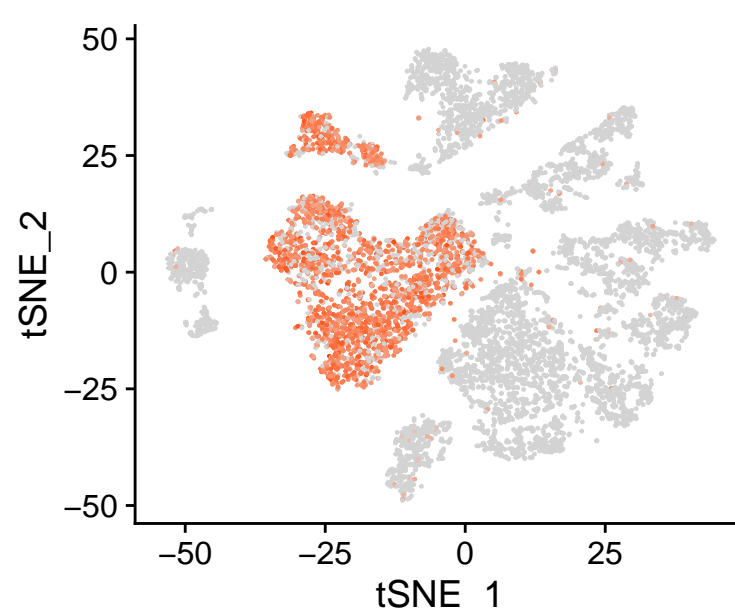**CD3D**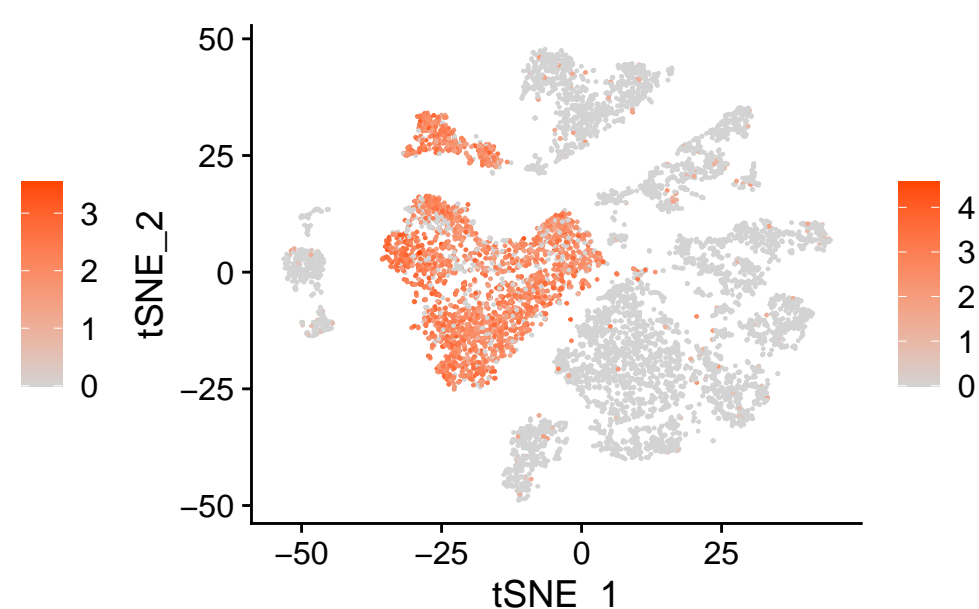**CD14**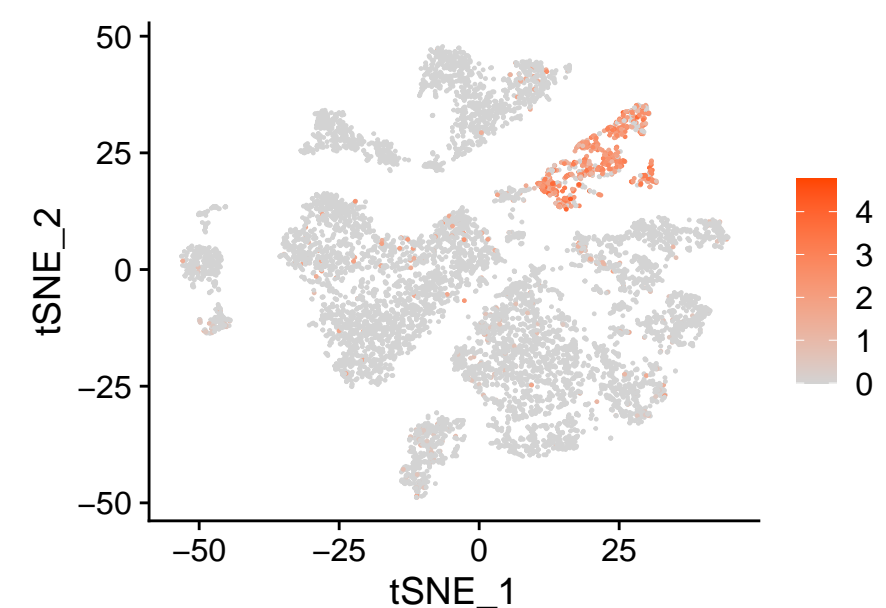**CD163**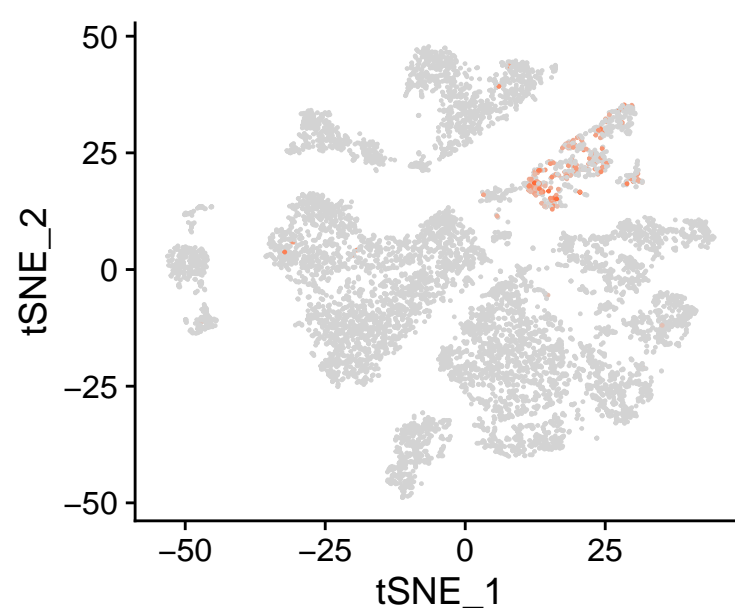**CD68**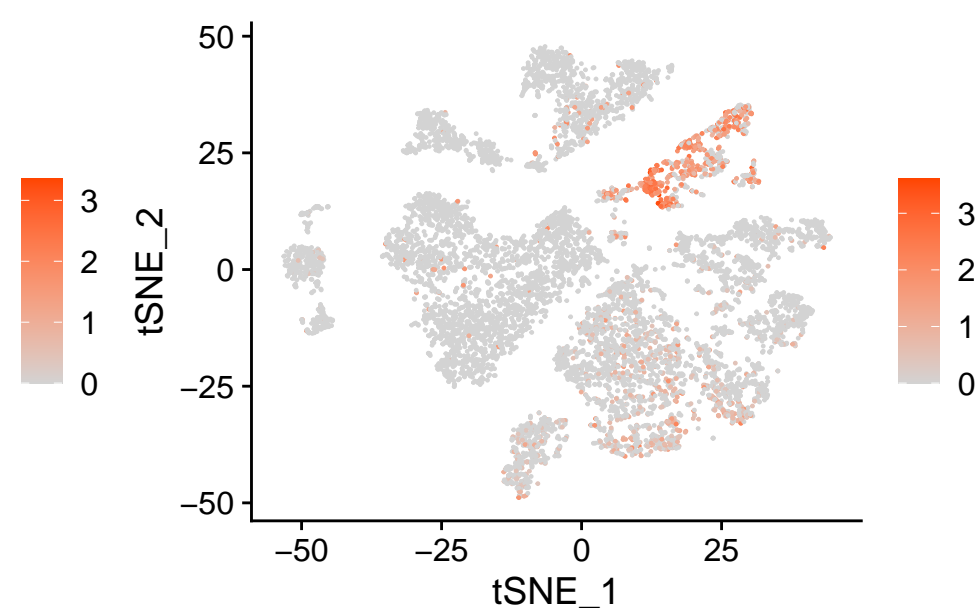**IL3RA**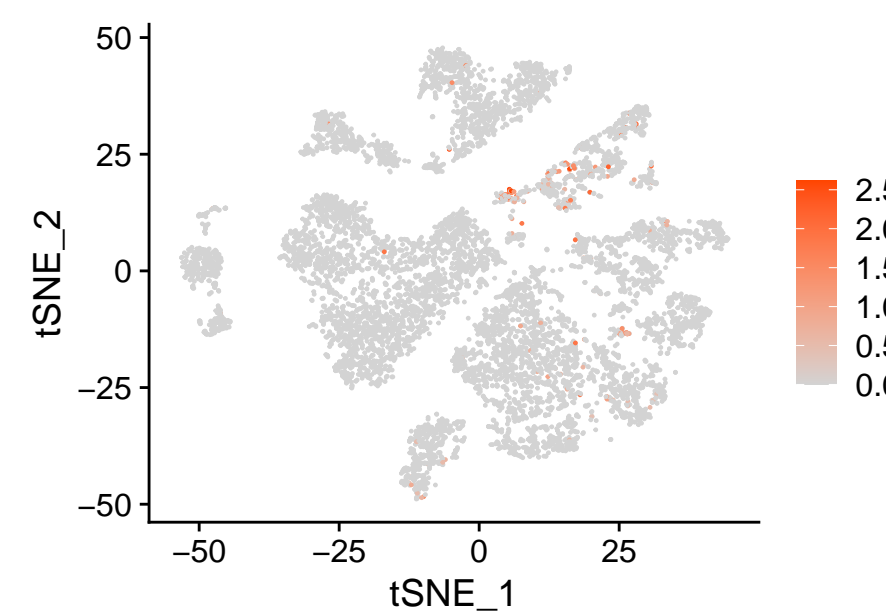**SERPINF1**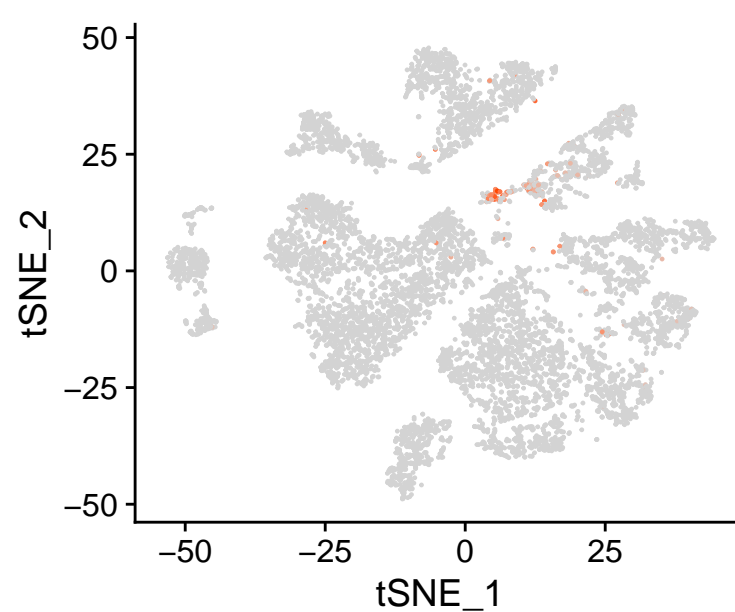**CCDC88A**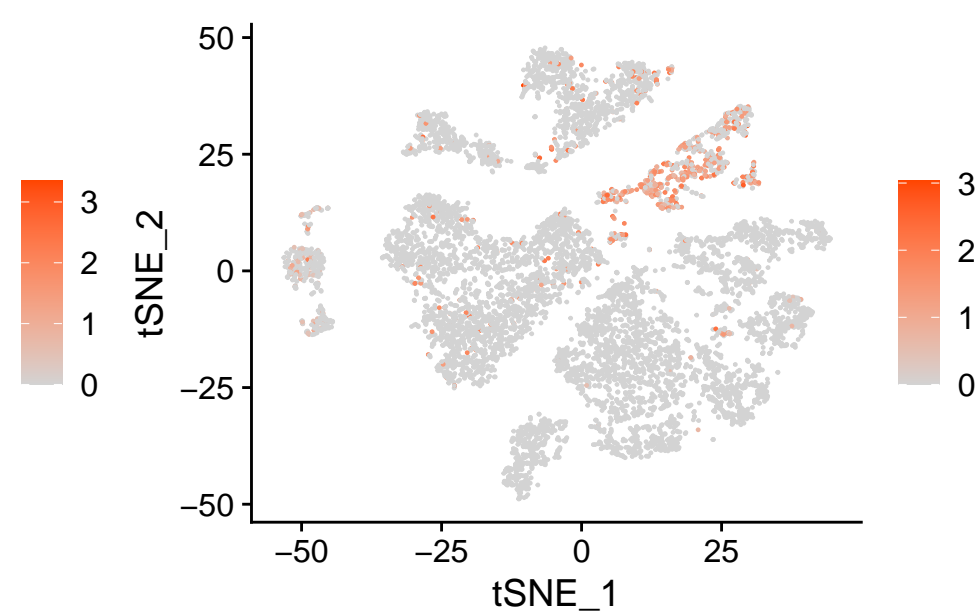

Supplement: Supplementary 1 — Figure S1: quality control of the scRNA-seq data from the four CRC samples. Figure S2: gene numbers in the four CRC samples are positively correlated with sequencing depth. Figure S3: PCA classification of the cells based on the scRNA-seq data. Figure S4: eleven statistically significant principal components as identified by PCA. Figure S5: dot diagram showing the expression of specific marker genes in the cell subsets. Figure S6: distribution of specific marker genes in the four cell subsets. Figure S7: LASSO regression was used to screen MECRGs with prognostic significance. [file 8347125.f1.zip › Figure S6.pdf]

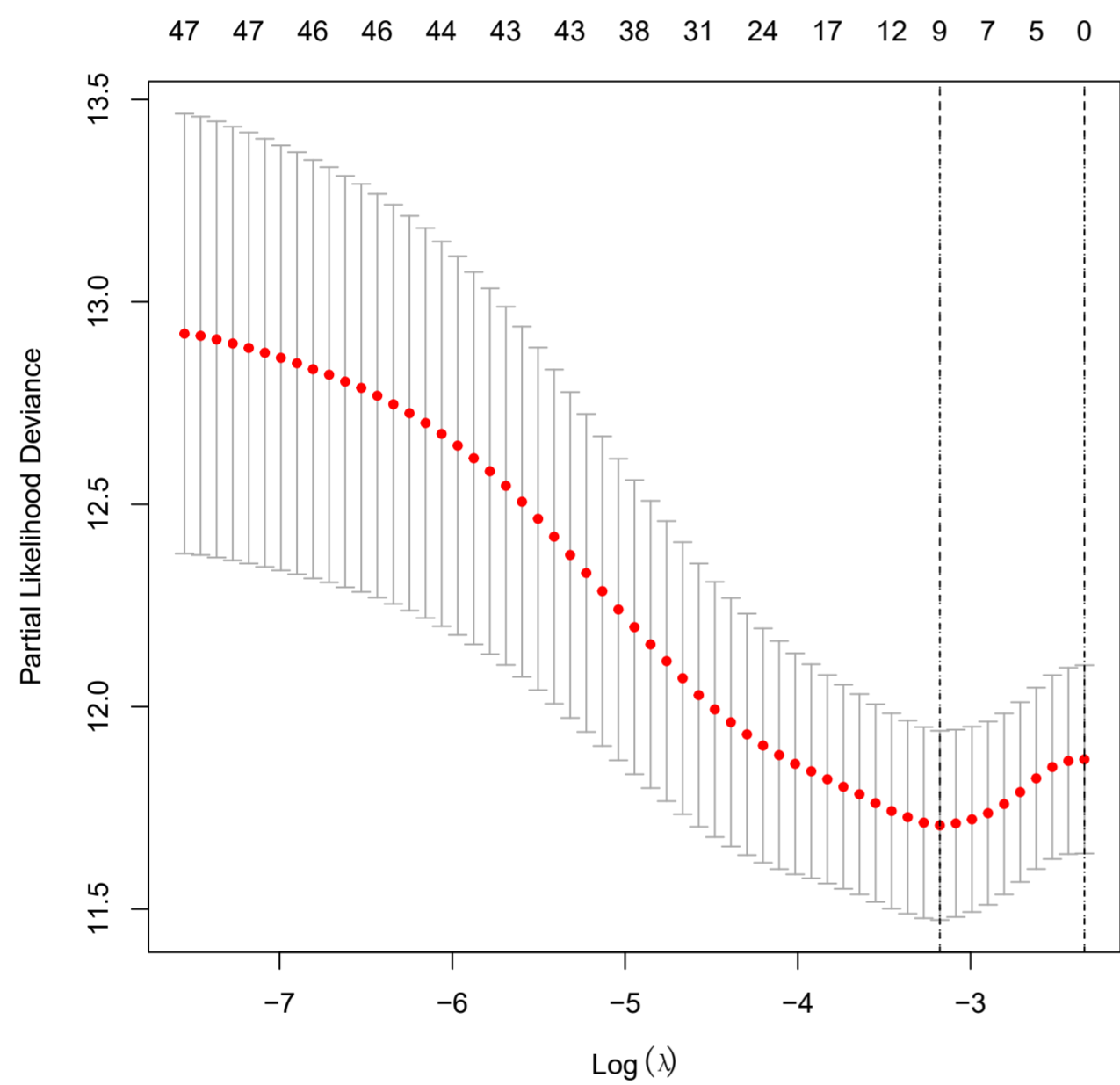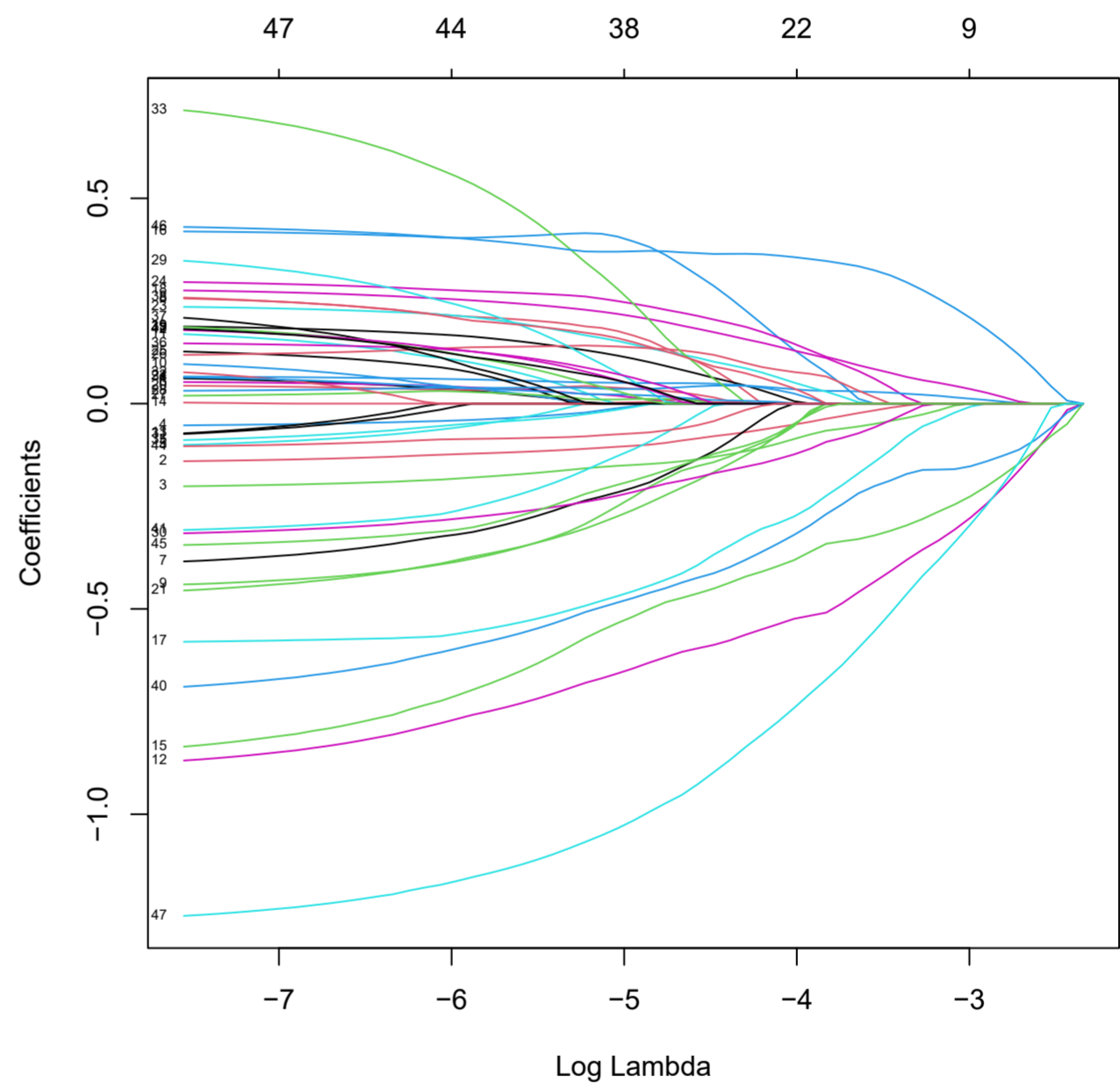

Supplement: Supplementary 1 — Figure S1: quality control of the scRNA-seq data from the four CRC samples. Figure S2: gene numbers in the four CRC samples are positively correlated with sequencing depth. Figure S3: PCA classification of the cells based on the scRNA-seq data. Figure S4: eleven statistically significant principal components as identified by PCA. Figure S5: dot diagram showing the expression of specific marker genes in the cell subsets. Figure S6: distribution of specific marker genes in the four cell subsets. Figure S7: LASSO regression was used to screen MECRGs with prognostic significance. [file 8347125.f1.zip › Figure S7.pdf]
