# Supplementary material for: Single-Cell RNA Sequencing Reveals the Role of Epithelial Cell Marker Genes in Predicting the Prognosis of Colorectal Cancer Patients
Source: Dis Markers. 2022 Aug 1;2022:8347125. doi: 10.1155/2022/8347125 (PMC9372514; doi:10.1155/2022/8347125)
Supplement: Supplementary 2 — Table S1: clinical characteristics of the four CRC patients. Table S2: CNV scores of epithelial cell subclusters. Table S3: MEC subclusters of marker genes. Table S4: MECRG signature using the TCGA-CRC training cohort. Table S5: MECRG signature using the GSE17538 validation cohort. [file 8347125.f2.zip › Table S1.pdf]

TableS1 Clinical characteristics of four CRC patients in this study

**Patient**	CRC1	CRC2	CRC3	CRC4
**Age**	46	61	64	53
**Gender**	Female	Male	Male	Female
**Site of tumor**	Colon	Colon	Rectum	Colon
**pTNM**	pT3pN0	pT3pN1b	pT4apN0	pT3pN0
**Stage**	IIA	IIIB	IIB	IIA
**Grade**	G2-G3	G2-G3	G2	G2-G3
