# Supplementary material for: Single-Cell RNA Sequencing Reveals the Role of Epithelial Cell Marker Genes in Predicting the Prognosis of Colorectal Cancer Patients
Source: Dis Markers. 2022 Aug 1;2022:8347125. doi: 10.1155/2022/8347125 (PMC9372514; doi:10.1155/2022/8347125)
Supplement: Supplementary 2 — Table S1: clinical characteristics of the four CRC patients. Table S2: CNV scores of epithelial cell subclusters. Table S3: MEC subclusters of marker genes. Table S4: MECRG signature using the TCGA-CRC training cohort. Table S5: MECRG signature using the GSE17538 validation cohort. [file 8347125.f2.zip › Table S2.pdf]

Table S2 CNV score of epithelial cell subclusters

Cell	Celltype	CNV Score
AGATTGCGTCTCCCTA-1_CRC0	Epithelial cells type1	1501
TGGACGCGTTACCGAT-1_CRC0	Epithelial cells type1	2056
CACAGGCCATTCACTT-1_CRC0	Epithelial cells type1	1444
CTGAAACTCAGTTAGC-1_CRC0	Epithelial cells type1	1283
ACGCAGCAGGTGCACA-1_CRC0	Epithelial cells type1	1761
CACACCTTCCTAGTGA-1_CRC0	Epithelial cells type1	1374
CACAGGCGTTCAGCGC-1_CRC0	Epithelial cells type1	2360
CTACCCAGTCAATACC-1_CRC0	Epithelial cells type1	2008
GCAGCCACACCATGTA-1_CRC0	Epithelial cells type1	1927
AACTCCCTCCTCTAGC-1_CRC0	Epithelial cells type1	1416
GGAATAAGTAGTACCT-1_CRC0	Epithelial cells type1	1422
TCGTACCAGAATGTTG-1_CRC0	Epithelial cells type1	1906
ATCATCTGTATATGAG-1_CRC0	Epithelial cells type1	2060
TCCCGATAGGTTACCT-1_CRC0	Epithelial cells type1	1587
GGCCGATCACTTCGAA-1_CRC0	Epithelial cells type1	1565
GGGCACTCATCCGGGT-1_CRC0	Epithelial cells type1	1582
AGGCCGTAGTCTTGCA-1_CRC0	Epithelial cells type1	2199
GCATGTATCATGTAGC-1_CRC0	Epithelial cells type1	2081
CGCTATCCATGGGAAC-1_CRC0	Epithelial cells type1	2216
CTGAAGTAGTTGAGAT-1_CRC0	Epithelial cells type1	2323
CATCGAAAGCCAGTTT-1_CRC0	Epithelial cells type1	1779
ACTTTCATCTCTGAGA-1_CRC0	Epithelial cells type1	1850
CCCTCCTGTTTAGCTG-1_CRC0	Epithelial cells type1	1964
CATGGCGAGGAACTGC-1_CRC0	Epithelial cells type1	1383
GACCAATGTGCGAAAC-1_CRC0	Epithelial cells type1	1515
GGTATTGCATCACGAT-1_CRC0	Epithelial cells type1	2052
CTGATAGCACCGTTGG-1_CRC0	Epithelial cells type1	2263
GTCCTCAGTCTAGTCA-1_CRC0	Epithelial cells type1	2115
GTCGTAACAGCTGTAT-1_CRC0	Epithelial cells type1	1716
CCTATTACAATGTTGC-1_CRC0	Epithelial cells type1	2336
CCTTCCCAGAGACGAA-1_CRC0	Epithelial cells type1	1429
GCAATCATCACGAAGG-1_CRC0	Epithelial cells type1	1765
CACCAGGTCCAAGCCG-1_CRC0	Epithelial cells type1	1912
GTCCTCACACCAGGTC-1_CRC0	Epithelial cells type1	1912
TTAGTTCAGTCCCACG-1_CRC0	Epithelial cells type1	1879
TTTCCTCCATCCTAGA-1_CRC0	Epithelial cells type1	2503
CCCATACCAAGCGATG-1_CRC0	Epithelial cells type1	2166
TAGTGGTCAGCTGTAT-1_CRC0	Epithelial cells type1	1847
GTGCATAGTCTAGTCA-1_CRC0	Epithelial cells type1	2163
GTTAAGCAGCCAGTAG-1_CRC0	Epithelial cells type1	2462
AAAGTAGAGCTGTCTA-1_CRC0	Epithelial cells type1	1730
GCACTCTCATTAGGCT-1_CRC0	Epithelial cells type1	1837
GTACTTTTCGGCGCAT-1_CRC0	Epithelial cells type1	1884
TGTGTTTTCACCGTAA-1_CRC0	Epithelial cells type1	2179
TTCTACATCCATGAAC-1_CRC0	Epithelial cells type1	2029
CTGCCTATCCAACCAA-1_CRC0	Epithelial cells type1	2100
GATGCTAGTCGCATCG-1_CRC0	Epithelial cells type1	1981
TCGAGGCTCCGCTGTT-1_CRC0	Epithelial cells type1	2128
AAGGTTCGTCTCCACT-1_CRC0	Epithelial cells type1	2103

CATCAAGGTCCTCTTG-1_CRC0	Epithelial cells type1	1897
AGTCTTTGTCGACTGC-1_CRC0	Epithelial cells type1	1505
GTTACAGAGCGTGTCC-1_CRC0	Epithelial cells type1	1640
AAATGCCGTGACAAAT-1_CRC0	Epithelial cells type1	2251
GCATGATGTCATCGGC-1_CRC0	Epithelial cells type1	1750
CAACTAGCACCAACCG-1_CRC0	Epithelial cells type1	1796
CGCTATCGTAGCACGA-1_CRC0	Epithelial cells type1	1546
GGTGCGTCATCTGGTA-1_CRC0	Epithelial cells type1	1869
AGCAGCCTCATCGGAT-1_CRC0	Epithelial cells type1	2323
GCTGGGTCAAGACGTG-1_CRC0	Epithelial cells type1	1987
ACTTGTTCAGTTTACG-1_CRC0	Epithelial cells type1	1790
CCTAGCTTCAACACCA-1_CRC0	Epithelial cells type1	2528
ATCTGCCTCCACGACG-1_CRC0	Epithelial cells type1	1747
ATTCTACGTATTCGTG-1_CRC0	Epithelial cells type1	2013
CCCTCCTTCACTTATC-1_CRC0	Epithelial cells type1	1982
TTCCCAGTCACAATGC-1_CRC0	Epithelial cells type1	1418
CGTCAGGTCTAACTGG-1_CRC0	Epithelial cells type1	1625
ATCCGAAGTGAGCGAT-1_CRC0	Epithelial cells type1	1007
GGGCACTGTTCGGCAC-1_CRC0	Epithelial cells type1	1703
ATGAGGGTCTGCCCTA-1_CRC0	Epithelial cells type1	1566
CTACATTTCTCGTATT-1_CRC0	Epithelial cells type1	1384
GCAGTTAAGCTGAAAT-1_CRC0	Epithelial cells type1	1589
GCACATATCATGTAGC-1_CRC0	Epithelial cells type1	1512
GGCCGATCACGCCAGT-1_CRC0	Epithelial cells type1	1635
TGAGGGACATGAAGTA-1_CRC0	Epithelial cells type1	1944
ATCCGAACAAGTTCTG-1_CRC0	Epithelial cells type1	1978
TACTTACCAGGTCGTC-1_CRC0	Epithelial cells type1	1985
GCGACCATCCCAAGTA-1_CRC0	Epithelial cells type1	2417
AAGGTTCTCAGAGGTG-1_CRC0	Epithelial cells type1	2436
AGGGATGCAGGTCTCG-1_CRC0	Epithelial cells type1	1509
TCAGGTAGTCATATCG-1_CRC0	Epithelial cells type1	1804
CGCTTCAGTGCACCAC-1_CRC0	Epithelial cells type1	1621
TCAGCAACAAGCTGGA-1_CRC0	Epithelial cells type1	1561
ATAAGAGGTTCGCGAC-1_CRC0	Epithelial cells type1	1901
CAAGATCGTAATTGGA-1_CRC0	Epithelial cells type1	1575
ACCCACTGTTAGTGGG-1_CRC0	Epithelial cells type1	1505
AGGTCCGAGGAGTACC-1_CRC0	Epithelial cells type1	1160
CGGCTAGAGATATACG-1_CRC0	Epithelial cells type1	1264
ACCGTAAGTTGTACAC-1_CRC0	Epithelial cells type1	1666
ACCTTTAAGTCCATAC-1_CRC0	Epithelial cells type1	1525
ACCTTTATCATGTGGT-1_CRC0	Epithelial cells type1	1947
TACTTGTAGATCCGAG-1_CRC0	Epithelial cells type1	1245
CTGCTGTTCAGTTTGG-1_CRC0	Epithelial cells type1	1379
TCTGGAAAGTACGTAA-1_CRC0	Epithelial cells type1	1632
CCCAGTTGTGGTACAG-1_CRC0	Epithelial cells type1	1685
CGGCTAGTCGCCAGCA-1_CRC0	Epithelial cells type1	1801
AGTGGGACATTCTCAT-1_CRC0	Epithelial cells type1	1864
TACTCGCGTAAATGAC-1_CRC0	Epithelial cells type1	1318
AGTAGTCAGCCCAATT-1_CRC0	Epithelial cells type1	1914
AAGGAGCGTGCACTTA-1_CRC0	Epithelial cells type1	1463
TGGGAAGGTACAGTGG-1_CRC0	Epithelial cells type1	1692

ATCTGCCTCTCTTGAT-1_CRC0	Epithelial cells type1	1860
CTAACTTCATTCCTCG-1_CRC0	Epithelial cells type1	1609
CTTTGCGGTGTTTGTG-1_CRC0	Epithelial cells type1	1788
TCTCTAATCGCATGAT-1_CRC0	Epithelial cells type1	1582
TGGCCAGCACGAAGCA-1_CRC0	Epithelial cells type1	1988
CGATCGGAGCCTTGAT-1_CRC0	Epithelial cells type1	2579
ACAGCTAGTAAACCTC-1_CRC0	Epithelial cells type1	2514
CCTCAGTCAGATCGGA-1_CRC0	Epithelial cells type1	1696
TCATTTGCAGGCTCAC-1_CRC0	Epithelial cells type1	2050
ATCTACTAGTCGAGTG-1_CRC0	Epithelial cells type1	2262
CAGAATCTCCAAGTAC-1_CRC0	Epithelial cells type1	1641
ACGCCGATCAGGCAAG-1_CRC0	Epithelial cells type1	2133
CCTTTCTGTTGGGACA-1_CRC0	Epithelial cells type1	1624
TCGTACCTCCTCATTA-1_CRC0	Epithelial cells type1	1208
AGAGCGACAGCGTTCG-1_CRC0	Epithelial cells type1	2044
TAGGCATAGCCTCGTG-1_CRC0	Epithelial cells type1	1562
TGCACCTGTGCTTCTC-1_CRC0	Epithelial cells type1	1858
CAGCCGAAGGCGACAT-1_CRC0	Epithelial cells type1	1804
GTTTCTATCCCATTAT-1_CRC0	Epithelial cells type1	1802
CAACCAATCGTACCGG-1_CRC0	Epithelial cells type1	1703
CGCGGTAAGTGGCACA-1_CRC0	Epithelial cells type1	1782
CGCTGGATCAGAGACG-1_CRC0	Epithelial cells type1	1545
AGCCTAACATTCCTCG-1_CRC0	Epithelial cells type1	1691
GGGCATCAGGGCTTGA-1_CRC0	Epithelial cells type1	1181
TCATTACGTGCCTGCA-1_CRC0	Epithelial cells type1	1782
TGGCCAGTCGCTAGCG-1_CRC0	Epithelial cells type1	1405
CCCATACTCTATCGCC-1_CRC0	Epithelial cells type1	1311
TCATTTGGTTCTGAAC-1_CRC0	Epithelial cells type1	1812
CACACTCCATCAGTAC-1_CRC0	Epithelial cells type1	1333
TGCGGGTGTAAGAGGA-1_CRC0	Epithelial cells type1	1499
CGTTAGAAGACTGTAA-1_CRC0	Epithelial cells type1	1275
TGAGCATTCCAAAGTC-1_CRC0	Epithelial cells type1	1499
GTTCTCGTCATGCATG-1_CRC0	Epithelial cells type1	1503
ACATGGTCAGCCACCA-1_CRC0	Epithelial cells type1	1365
AGAGTGGGTTTGCATG-1_CRC0	Epithelial cells type1	1390
TACGGGCCACTTCGAA-1_CRC0	Epithelial cells type1	1519
TCGTACCAGTGCTGCC-1_CRC0	Epithelial cells type1	1448
ATCTGCCCAAGGCTCC-1_CRC0	Epithelial cells type1	2453
CGAATGTAGAGCTGGT-1_CRC0	Epithelial cells type1	1891
GGACGTCTCCAAGTAC-1_CRC0	Epithelial cells type1	1560
AACTTTCCAGATCCAT-1_CRC0	Epithelial cells type1	1955
CTTAGGAAGTTATCGC-1_CRC0	Epithelial cells type1	1789
CCTTTCTTCGGAAATA-1_CRC0	Epithelial cells type1	1506
CGAGCACGTGTTTGGT-1_CRC0	Epithelial cells type1	1336
CGACCTTGTAGCGTAG-1_CRC0	Epithelial cells type1	2653
ATAAGAGCACGTGAGA-1_CRC0	Epithelial cells type1	1470
GGGCACTAGGATTCGG-1_CRC0	Epithelial cells type1	1603
ACACCGGAGTCGCCGT-1_CRC0	Epithelial cells type1	1598
CCATGTCGTACTTGAC-1_CRC0	Epithelial cells type1	1234
GTGTGCGAGTACGCGA-1_CRC0	Epithelial cells type1	1484
GACTAACAGGGTTTCT-1_CRC0	Epithelial cells type1	1395

GTCTTCGCAATCGGTT-1_CRC0	Epithelial cells type1	985
GCCTCTAAGTGCCATT-1_CRC0	Epithelial cells type1	1574
CCCAGTTTCGCTAGCG-1_CRC0	Epithelial cells type1	1900
TACTTGTCAAGAGTCG-1_CRC0	Epithelial cells type1	1807
ACGAGCCGTCGAGTTT-1_CRC0	Epithelial cells type1	1775
CTAGCCTGTGGTGTAG-1_CRC0	Epithelial cells type1	2108
CGAGAAGAGGTGATAT-1_CRC0	Epithelial cells type1	1828
GACTACATCCCGGATG-1_CRC0	Epithelial cells type1	1562
AGGTCCGTCAGTACGT-1_CRC0	Epithelial cells type1	1500
CCTTCCCCAGCTCGCA-1_CRC0	Epithelial cells type1	1806
CGTGTAAAGGTCATCT-1_CRC0	Epithelial cells type1	1564
TTCTTAGGTAAATGTG-1_CRC0	Epithelial cells type1	1373
ACCTTTAGTCCGTGAC-1_CRC0	Epithelial cells type1	1779
TAGTGGTAGGTGCACA-1_CRC0	Epithelial cells type1	1619
ACGGGTCTCATGTGGT-1_CRC0	Epithelial cells type1	1440
CGACCTTCAGACAAGC-1_CRC0	Epithelial cells type1	1920
GGCTCGAAGAGATGAG-1_CRC0	Epithelial cells type1	1623
AGATCTGGTTCCTCCA-1_CRC0	Epithelial cells type1	1467
CACACAATCAGCACAT-1_CRC0	Epithelial cells type1	1704
TCAGGATAGAGTACAT-1_CRC0	Epithelial cells type1	1658
ATTTCTGTCGAACGGA-1_CRC0	Epithelial cells type1	1930
GGGTTGCGTAGGAGTC-1_CRC0	Epithelial cells type1	1742
CCATGTCGTTGTGGAG-1_CRC0	Epithelial cells type1	1715
GCGCAACGTTATCCGA-1_CRC0	Epithelial cells type1	1749
ACACCCTTCTTACCTA-1_CRC0	Epithelial cells type1	1774
AGAGTGGTCAGTACGT-1_CRC0	Epithelial cells type1	1849
GCTGCGACAAGTCATC-1_CRC0	Epithelial cells type1	2197
AGCATACCAGCTGTAT-1_CRC0	Epithelial cells type1	1830
CTCATTAAGAGACGAA-1_CRC0	Epithelial cells type1	1365
GGCTGGTGTAGAAAGG-1_CRC0	Epithelial cells type1	1785
CTAGCCTCAATAAGCA-1_CRC0	Epithelial cells type1	1917
AGCATACGTCCAGTGC-1_CRC0	Epithelial cells type1	1656
CTCTACGCACTGTTAG-1_CRC0	Epithelial cells type1	1851
GATCTAGGTTGGTTTG-1_CRC0	Epithelial cells type1	1896
CTCATTACAATCACAC-1_CRC0	Epithelial cells type1	1959
CGACTTCGTTGATTCG-1_CRC0	Epithelial cells type1	1770
GAATGAAAGTGGAGTC-1_CRC0	Epithelial cells type1	1615
CAAGAAAAGAATTCCC-1_CRC0	Epithelial cells type1	1957
CATTATCAGGACAGAA-1_CRC0	Epithelial cells type1	1439
TGTATTCAGTACACCT-1_CRC0	Epithelial cells type1	1801
GGAATAACAATCCAAC-1_CRC0	Epithelial cells type1	1470
GGGAATGGTTACCAGT-1_CRC0	Epithelial cells type1	1923
TCTATTGAGCCCAACC-1_CRC0	Epithelial cells type1	1479
ACGGCCATCTGAGGGA-1_CRC0	Epithelial cells type1	1995
CTGAAACCAAGGGTCA-1_CRC0	Epithelial cells type1	1745
AGGGTGACAATGGAGC-1_CRC0	Epithelial cells type1	1685
ATTACTCCAAAGCGGT-1_CRC0	Epithelial cells type1	1431
ACGGAGACACAGACAG-1_CRC0	Epithelial cells type1	1171
CACACCTAGAGTACAT-1_CRC0	Epithelial cells type1	1468
GATCGCGTCCTATGTT-1_CRC0	Epithelial cells type1	1224
GATGCTAAGCGGATCA-1_CRC0	Epithelial cells type1	1350

CTGATAGAGACTAAGT-1_CRC0	Epithelial cells type1	1994
TTTATGCCAGCATACT-1_CRC0	Epithelial cells type1	1704
CGTAGGCTCAGTCCCT-1_CRC0	Epithelial cells type1	1742
ACCTTTATCAAGATCC-1_CRC0	Epithelial cells type1	1129
CACACCTCACGTCTCT-1_CRC0	Epithelial cells type1	1266
TCGTACCTCAACTCTT-1_CRC0	Epithelial cells type1	1942
CCCAATCAGTTCCACA-1_CRC0	Epithelial cells type1	1632
GGTGAAGAGATTACCC-1_CRC0	Epithelial cells type1	1671
GTTCGGGAGTAGGCCA-1_CRC0	Epithelial cells type1	1879
AGTGAGGTCTAACTTC-1_CRC0	Epithelial cells type1	1538
CTCGAAACAAGTCTGT-1_CRC0	Epithelial cells type1	1645
TCGTACCTCACCACCT-1_CRC0	Epithelial cells type1	1469
ACGCCGATCATTTGGG-1_CRC0	Epithelial cells type1	1764
TGCACCTGTCTAAACC-1_CRC0	Epithelial cells type1	1661
CGTCCATTCACCCGAG-1_CRC0	Epithelial cells type1	1872
CTCTAATTCCCGACTT-1_CRC0	Epithelial cells type1	1690
GATCGCGTCTGCTGCT-1_CRC0	Epithelial cells type1	1901
AAAGTAGAGAGTACAT-1_CRC0	Epithelial cells type1	2078
AGCGTATAGTGCCATT-1_CRC0	Epithelial cells type1	1662
ATCGAGTGTTCGAATC-1_CRC0	Epithelial cells type1	1449
TCAACGAGTCAAACTC-1_CRC0	Epithelial cells type1	1309
ACGATACAGACTAGAT-1_CRC0	Epithelial cells type1	1420
ACTGATGTCGAATGCT-1_CRC0	Epithelial cells type1	1403
ACAGCTAGTGTGGCTC-1_CRC0	Epithelial cells type1	1556
CCCTCCTTCTCGCATC-1_CRC0	Epithelial cells type1	1305
GTGCAGCTCATCTGTT-1_CRC0	Epithelial cells type1	1247
AGGCCGTGTCTACCTC-1_CRC0	Epithelial cells type1	947
CTGATCCCACATCTTT-1_CRC0	Epithelial cells type1	1429
GAAGCAGCATTGAGCT-1_CRC0	Epithelial cells type1	1716
ACACTGAAGACTAAGT-1_CRC0	Epithelial cells type1	1769
TTTGGTTTCTAACTTC-1_CRC0	Epithelial cells type1	1601
TGGACGCCAATCCGAT-1_CRC0	Epithelial cells type1	1166
TAGGCATGTTACGTCA-1_CRC0	Epithelial cells type1	1695
GATCTAGGTAATTGGA-1_CRC0	Epithelial cells type1	1692
TGTATTCTCACAACGT-1_CRC0	Epithelial cells type1	1312
CGACCTTTCGACCAGC-1_CRC0	Epithelial cells type1	1262
TAGGCATGTTACGGAG-1_CRC0	Epithelial cells type1	1152
TACCTTAGTCGCGGTT-1_CRC0	Epithelial cells type1	1120
TTTCCTCAGATGAGAG-1_CRC0	Epithelial cells type1	933
CCACTACCATAGAAAC-1_CRC0	Epithelial cells type1	1399
CTTAACTAGCGTGAGT-1_CRC0	Epithelial cells type1	1287
TTCGAAGCACTGTCGG-1_CRC0	Epithelial cells type1	1626
ACTATCTCATTCTTAC-1_CRC0	Epithelial cells type1	1592
AGCTTGATCTCCTATA-1_CRC0	Epithelial cells type1	1237
CCACGGATCAATCACG-1_CRC0	Epithelial cells type1	1441
CATCAGAGTATAATGG-1_CRC0	Epithelial cells type1	1695
CATATTCTCGACGGAA-1_CRC0	Epithelial cells type1	1310
CTCGTACTCTGAGGGA-1_CRC0	Epithelial cells type1	762
CTCTAATGTAGCGCAA-1_CRC0	Epithelial cells type1	1184
CCAATCCCATGACGGA-1_CRC0	Epithelial cells type1	1112
CCTACCAAGGATCGCA-1_CRC0	Epithelial cells type1	1171

CATGCCTTCGGGAGTA-1_CRC0	Epithelial cells type1	1179
CCCATACAGGCTATCT-1_CRC0	Epithelial cells type1	1411
ACGCCAGAGAGCTTCT-1_CRC0	Epithelial cells type1	1033
ATTTCTGTCACCGGGT-1_CRC0	Epithelial cells type1	719
CAACTAGCATCCCATC-1_CRC0	Epithelial cells type1	768
CTACACCGTTCAACCA-1_CRC0	Epithelial cells type1	1228
GTGCGGTCAACACCTA-1_CRC0	Epithelial cells type1	1144
TCGCGTTGTTACTGAC-1_CRC0	Epithelial cells type1	1406
AAACGGGCATTACCTT-1_CRC0	Epithelial cells type1	1056
GTGTTAGTCTTACCTA-1_CRC0	Epithelial cells type1	1039
CGATCGGTCTTTCCTC-1_CRC0	Epithelial cells type1	1384
CCGGTAGAGCCCGAAA-1_CRC0	Epithelial cells type1	1277
AGACGTTAGCGTCAAG-1_CRC0	Epithelial cells type1	1150
CTCGGGATCACATAGC-1_CRC0	Epithelial cells type1	1143
ACTTTCAAGATGTAAC-1_CRC0	Epithelial cells type1	806
ATAACGCTCGTTACGA-1_CRC0	Epithelial cells type1	933
ACTGATGCAAACCTAC-1_CRC0	Epithelial cells type1	1407
AAGGCAGGTATCAGTC-1_CRC0	Epithelial cells type1	1605
TCTATTGAGACGCAAC-1_CRC0	Epithelial cells type1	1296
CTGCGGATCGTTACGA-1_CRC0	Epithelial cells type1	1824
TTTGTCATCACAATGC-1_CRC0	Epithelial cells type1	1729
GGGATGATCCTACAGA-1_CRC0	Epithelial cells type1	1891
GTGCTTCTCGAGAACG-1_CRC0	Epithelial cells type1	1667
GGCTGGTTCTGCTGTC-1_CRC0	Epithelial cells type1	2118
CAGAGAGGTCCGTCAG-1_CRC0	Epithelial cells type1	1867
TAAGAGAAGACCCACC-1_CRC0	Epithelial cells type1	1480
CGTCAGGGTCTCGTTC-1_CRC0	Epithelial cells type1	1781
CTGGTCTGTCAGATAA-1_CRC0	Epithelial cells type1	1263
AACTCAGAGACCTTTG-1_CRC0	Epithelial cells type1	1423
CTTAACTGTCAGATAA-1_CRC0	Epithelial cells type1	1385
CTGTGCTCAGTGACAG-1_CRC0	Epithelial cells type1	2058
ACCAGTAGTCATATCG-1_CRC0	Epithelial cells type1	1603
CGAGAAGCACTTACGA-1_CRC0	Epithelial cells type1	1093
CTACATTGTCAGCTAT-1_CRC0	Epithelial cells type1	1311
CTAGAGTGTGTGCGTC-1_CRC0	Epithelial cells type1	1039
AGTCTTTTCTTGTACT-1_CRC0	Epithelial cells type1	911
TACAGTGCATCACGAT-1_CRC0	Epithelial cells type1	1416
GTAACGTTCCGGGTGT-1_CRC0	Epithelial cells type1	1175
TGGCTGGCACTGAAGG-1_CRC0	Epithelial cells type1	1460
TGACTAGGTCCAGTGC-1_CRC0	Epithelial cells type1	804
TTAGGCAGTCAATACC-1_CRC0	Epithelial cells type1	1752
GACGTGCAGAGGGATA-1_CRC0	Epithelial cells type1	2065
AAATGCCGTCGGCACT-1_CRC0	Epithelial cells type1	1501
TCCACACTCGTCGTTC-1_CRC0	Epithelial cells type1	1342
GATCGCGAGTACGCCC-1_CRC0	Epithelial cells type1	864
GCGAGAATCAAGATCC-1_CRC0	Epithelial cells type1	1264
GTTCATTTCTTCCTTC-1_CRC0	Epithelial cells type1	1117
GCCTCTACAAAGGCGT-1_CRC0	Epithelial cells type1	1136
TGCCAAACAGACTCGC-1_CRC0	Epithelial cells type1	1264
ACGCAGCGTACTTGAC-1_CRC0	Epithelial cells type1	1640
CGAGCCAGTAAGCACG-1_CRC0	Epithelial cells type1	1443

GAATAAGTCATAGCAC-1_CRC0	Epithelial cells type1	2018
GTCAAGTGTTCGTTGA-1_CRC0	Epithelial cells type1	1140
CTCTAATGTCTAGTGT-1_CRC0	Epithelial cells type1	1264
AGTCTTTAGAGGTTAT-1_CRC0	Epithelial cells type1	952
ATGGGAGCATCACGTA-1_CRC0	Epithelial cells type1	1610
ACTGAACAGTGTGGCA-1_CRC0	Epithelial cells type1	1178
TCAGGATGTTATCCGA-1_CRC0	Epithelial cells type1	1209
TGAAAGATCAGCTGGC-1_CRC0	Epithelial cells type1	1624
GTGCAGCAGATTACCC-1_CRC0	Epithelial cells type1	1620
AACCATGAGTGTGAAT-1_CRC0	Epithelial cells type1	1401
CCTCTGAAGATCCCAT-1_CRC0	Epithelial cells type1	1124
GAAACTCCAGCCACCA-1_CRC0	Epithelial cells type1	2051
ACTGAACCATTGGTAC-1_CRC0	Epithelial cells type1	1353
GTACGTACACATGTGT-1_CRC0	Epithelial cells type1	1444
TACAGTGGTTTCGCTC-1_CRC0	Epithelial cells type1	1013
CTACGTCCATGTCGAT-1_CRC0	Epithelial cells type1	1424
CACACTCGTAGGCTGA-1_CRC0	Epithelial cells type1	1154
CTAGAGTAGACAGAGA-1_CRC0	Epithelial cells type1	1391
AGTTGGTGTCAATACC-1_CRC0	Epithelial cells type1	1981
TGGGAAGAGGCTCATT-1_CRC0	Epithelial cells type1	1099
CCTACACTCTGTCAAG-1_CRC0	Epithelial cells type1	1086
GTTCATTGTAAGCACG-1_CRC0	Epithelial cells type1	1166
AAATGCCTCTATGTGG-1_CRC0	Epithelial cells type1	1598
AATCGGTAGTGTGAAT-1_CRC0	Epithelial cells type1	1438
CAGATCAAGCCGTCGT-1_CRC0	Epithelial cells type1	1595
CCTATTATCAGGCGAA-1_CRC0	Epithelial cells type1	1994
CTACACCCATGTCGAT-1_CRC0	Epithelial cells type1	1605
CTGTTTACAGTACACT-1_CRC0	Epithelial cells type1	1590
CGGCTAGTCACCAGGC-1_CRC0	Epithelial cells type1	828
TATCTCAAGAAGATTC-1_CRC0	Epithelial cells type1	1059
CGACTTCAGCGAGAAA-1_CRC0	Epithelial cells type1	1113
CAGAATCCACACCGAC-1_CRC0	Epithelial cells type1	1480
GGCGACTAGACGCAAC-1_CRC0	Epithelial cells type1	1119
TGGTTCCAGACTACAA-1_CRC0	Epithelial cells type1	1331
GGAAAGCGTACCGTAT-1_CRC0	Epithelial cells type1	1815
GTACTCCCAGCGTCCA-1_CRC0	Epithelial cells type1	2232
CACAGGCTCATTTGGG-1_CRC0	Epithelial cells type1	1706
TTCTTAGTCGCGGATC-1_CRC0	Epithelial cells type1	1600
GCAATCATCAGATAAG-1_CRC0	Epithelial cells type1	1600
TGCACCTCAGCTGTGC-1_CRC0	Epithelial cells type1	1555
CAGCTGGGTCTTGCGG-1_CRC0	Epithelial cells type1	1335
TGACAACCACATTAGC-1_CRC0	Epithelial cells type1	1229
AACTCCCGTCTACCTC-1_CRC0	Epithelial cells type1	1623
ATAAGAGAGCGATATA-1_CRC0	Epithelial cells type1	1160
CCTCTGATCTGCTGCT-1_CRC0	Epithelial cells type1	1135
GTAACTGCACTCAGGC-1_CRC0	Epithelial cells type1	1909
ACTGCTCAGAATCTCC-1_CRC0	Epithelial cells type1	1648
CAAGTTGTCAACTCTT-1_CRC0	Epithelial cells type1	1813
GCCTCTATCAGTACGT-1_CRC0	Epithelial cells type1	1398
TGAAAGATCGGAGCAA-1_CRC0	Epithelial cells type1	1262
CTACATTGTTCTGTTT-1_CRC0	Epithelial cells type1	1470

GAATGAATCAGGTTCA-1_CRC0	Epithelial cells type1	1529
GTTTCTACACGGCCAT-1_CRC0	Epithelial cells type1	2717
CTGAAGTCATTTGCTT-1_CRC0	Epithelial cells type1	1459
TGACGGCTCACATACG-1_CRC0	Epithelial cells type1	1783
TTCGAAGTCTGCGACG-1_CRC0	Epithelial cells type1	1839
AAGTCTGAGGACAGAA-1_CRC0	Epithelial cells type1	2440
GCAATCACAAGCCGCT-1_CRC0	Epithelial cells type1	1717
CCGTACTGTGCAGGTA-1_CRC0	Epithelial cells type1	1568
GGGTCTGCACCGAATT-1_CRC0	Epithelial cells type1	1656
GATTCAGAGGCTCTTA-1_CRC0	Epithelial cells type1	1501
TTGACTTGTGCAACGA-1_CRC0	Epithelial cells type1	1497
TCAGCTCGTAGGGTAC-1_CRC0	Epithelial cells type1	2457
TCTCTAAGTCTTGCGG-1_CRC0	Epithelial cells type1	1587
AGTGAGGCATCTCCCA-1_CRC0	Epithelial cells type1	1560
GCTGCAGTCGCATGAT-1_CRC0	Epithelial cells type1	1643
CTAGCCTGTCGTGGCT-1_CRC0	Epithelial cells type1	1797
TACCTATCAGTAACGG-1_CRC0	Epithelial cells type1	1476
ACTTTCAAGTAAGTAC-1_CRC0	Epithelial cells type1	1370
CATGCCTCATCGGGTC-1_CRC0	Epithelial cells type1	1444
GATCTAGGTAAAGTCA-1_CRC0	Epithelial cells type1	1572
CTTTGCGAGAGTAAGG-1_CRC0	Epithelial cells type1	1556
TCACGAACAGCTCCGA-1_CRC0	Epithelial cells type1	2196
CCTTACGTCCGCTGTT-1_CRC0	Epithelial cells type1	2057
TCACAAGCAATAACGA-1_CRC0	Epithelial cells type1	1894
CCGTGGAGTAAGTGGC-1_CRC0	Epithelial cells type1	1510
GACGGCTAGGCAGGTT-1_CRC0	Epithelial cells type1	1552
TCCCGATGTGAAATCA-1_CRC0	Epithelial cells type1	2247
ACACCGGCAATGCCAT-1_CRC0	Epithelial cells type1	1697
CCAATCCTCTGCTGTC-1_CRC0	Epithelial cells type1	1319
GATCGTATCTTTAGGG-1_CRC0	Epithelial cells type1	1611
GTCATTTAGACATAAC-1_CRC0	Epithelial cells type1	2289
ACGGGCTCATACGCCG-1_CRC0	Epithelial cells type1	1680
CAGCGACCAGCCAATT-1_CRC0	Epithelial cells type1	1914
ATCACGATCCGTCAAA-1_CRC0	Epithelial cells type1	2357
CGGACACGTCTCATCC-1_CRC0	Epithelial cells type1	2143
GTACGTATCACTCCTG-1_CRC0	Epithelial cells type1	2300
TAGTTGGAGCAATATG-1_CRC0	Epithelial cells type1	2864
CAGCGACCAGGAACGT-1_CRC0	Epithelial cells type1	2172
CTGAAGTAGCATCATC-1_CRC0	Epithelial cells type1	2046
CCGTACTAGAGGACGG-1_CRC0	Epithelial cells type1	1910
AGCCTAATCTAACTCT-1_CRC0	Epithelial cells type1	2530
TGCCAAAGTCTTGTCC-1_CRC0	Epithelial cells type1	2593
CTCGTCACATTAACCG-1_CRC0	Epithelial cells type1	2060
TGCTGCTTCTTACCGC-1_CRC0	Epithelial cells type1	2031
CGACCTTTCCAAAGTC-1_CRC0	Epithelial cells type1	2557
GTACTTTAGCTAGTCT-1_CRC0	Epithelial cells type1	2568
ACACCGGCATGGATGG-1_CRC0	Epithelial cells type1	1770
GTTCATTAGTTAGCGG-1_CRC0	Epithelial cells type1	1333
CTTTGCGGTTTCGCTC-1_CRC0	Epithelial cells type1	1613
GGGACCTCAACGCACC-1_CRC0	Epithelial cells type1	1465
TTAGGCAGTTAGAACA-1_CRC0	Epithelial cells type1	1438

CCGTTCAGTCTACCTC-1_CRC0	Epithelial cells type1	1638
GAGTCCGTCTCGAGTA-1_CRC0	Epithelial cells type1	1857
GTGCATATCCACGAAT-1_CRC0	Epithelial cells type1	2078
TACTTGTCATGCATGT-1_CRC0	Epithelial cells type1	2043
CGTCCATCATTACCTT-1_CRC0	Epithelial cells type1	1582
CACCAGGCAGCCTATA-1_CRC0	Epithelial cells type1	1745
TCGTAGAGTCAGATAA-1_CRC0	Epithelial cells type1	1992
CGCTGGACAGCTGTGC-1_CRC0	Epithelial cells type1	1300
TGGTTAGCAATACGCT-1_CRC0	Epithelial cells type1	2194
CGTTAGAAGAGCCCAA-1_CRC0	Epithelial cells type1	1920
TGGTTAGCAAGTCTAC-1_CRC0	Epithelial cells type1	1548
CCTAGCTTCAAAGACA-1_CRC0	Epithelial cells type1	2167
GATCGATAGAGTCTGG-1_CRC0	Epithelial cells type1	1771
TGCCCTAAGTGATCGG-1_CRC0	Epithelial cells type1	2118
CGACCTTGTCTGATCA-1_CRC0	Epithelial cells type1	1405
CTTCTCTCAACGATGG-1_CRC0	Epithelial cells type1	1654
CGTCAGGTCTCGAGTA-1_CRC0	Epithelial cells type1	2062
TCGGGACCATGGATGG-1_CRC0	Epithelial cells type1	2172
TACGGGCGTCATCCCT-1_CRC0	Epithelial cells type1	2329
GGGATGAAGGGAACGG-1_CRC0	Epithelial cells type1	1975
ATCCGAAAGGAGTTGC-1_CRC0	Epithelial cells type1	2305
GGGTTGCTCCTATGTT-1_CRC0	Epithelial cells type1	2223
ATAGACCGTTAAAGTG-1_CRC0	Epithelial cells type1	1858
CACACCTCATGGTTGT-1_CRC0	Epithelial cells type1	2161
CAACTAGGTGGGTCAA-1_CRC0	Epithelial cells type1	1527
TGGACGCAGACCACGA-1_CRC0	Epithelial cells type1	1789
ATCTACTTCGCATGAT-1_CRC0	Epithelial cells type1	1880
CGTGTCTCACCCAGTG-1_CRC0	Epithelial cells type1	1280
TTCTCAAGTAACGCGA-1_CRC0	Epithelial cells type1	1767
CTAACTTTCTGCAGTA-1_CRC0	Epithelial cells type1	1857
TCATTACAGAATGTTG-1_CRC0	Epithelial cells type1	1726
AAATGCCCATAGTAAG-1_CRC0	Epithelial cells type1	1916
ACTGAGTGTCGCATCG-1_CRC0	Epithelial cells type1	1774
TACAGTGAGATGTAAC-1_CRC0	Epithelial cells type1	1676
TGGCGCATCCACTCCA-1_CRC0	Epithelial cells type1	1712
TTAGTTCTCCGTAGGC-1_CRC0	Epithelial cells type1	1952
CTCAGAACATCAGTAC-1_CRC0	Epithelial cells type1	1675
TATCAGGTCAACGGGA-1_CRC0	Epithelial cells type1	1684
ACCTTTAAGGGTGTTG-1_CRC0	Epithelial cells type1	1949
GCATACAGTTCAGGCC-1_CRC0	Epithelial cells type1	1574
CGTCCATAGAAGATTC-1_CRC0	Epithelial cells type1	1602
ACGGCCAAGTTGCAGG-1_CRC0	Epithelial cells type1	2082
GCATACAGTTCCATGA-1_CRC0	Epithelial cells type1	1512
GATGAAACAACTGCTA-1_CRC0	Epithelial cells type1	1487
CAGTAACCACGGCCAT-1_CRC0	Epithelial cells type1	1180
TACACGACAGCATACT-1_CRC0	Epithelial cells type1	1572
CACACCTGTGCCTGTG-1_CRC0	Epithelial cells type1	2127
CACATAGCACAACGCC-1_CRC0	Epithelial cells type1	2141
CATGACAGTAGAGTGC-1_CRC0	Epithelial cells type1	1829
GTTTCTATCCCGGATG-1_CRC0	Epithelial cells type1	1457
ACCAGTACAGTCTTCC-1_CRC0	Epithelial cells type1	1654

CTCGAAAGTTTGTTGG-1_CRC0	Epithelial cells type1	1334
GTTAAGCGTGAGCGAT-1_CRC0	Epithelial cells type1	2136
TGGGCGTCAGCGTTCG-1_CRC0	Epithelial cells type1	1886
GCTTCCAGTCAAAGCG-1_CRC0	Epithelial cells type1	1716
ACATACGTCTGATACG-1_CRC0	Epithelial cells type1	1690
ACTGATGAGCGTAGTG-1_CRC0	Epithelial cells type1	1425
AGCGTATTCGACGGAA-1_CRC0	Epithelial cells type1	1363
ACTGATGGTCTTGTCC-1_CRC0	Epithelial cells type1	1546
CAGCTGGTCCTCTAGC-1_CRC0	Epithelial cells type1	1610
ACACCCTTCGGACAAG-1_CRC0	Epithelial cells type1	1145
CGCGGTAAGCTATGCT-1_CRC0	Epithelial cells type1	1906
AACTCCCGTCGAGATG-1_CRC0	Epithelial cells type1	1527
GACGGCTGTTGGGACA-1_CRC0	Epithelial cells type1	1343
GCTCTGTGTCCAACTA-1_CRC0	Epithelial cells type1	1555
CCTACCAGTGTTGGGA-1_CRC0	Epithelial cells type1	1404
GGACAGAAGAGCCCAA-1_CRC0	Epithelial cells type1	1691
TTGGCAAAGGCCCGTT-1_CRC0	Epithelial cells type1	1713
CAGCTGGTCTTGAGAC-1_CRC0	Epithelial cells type1	1769
TCACAAGCAAGTAGTA-1_CRC0	Epithelial cells type1	1576
TGACGGCTCTGGCGAC-1_CRC0	Epithelial cells type1	1545
CGGTTAACACGAAATA-1_CRC0	Epithelial cells type1	1577
CTCGGGAAGGCATGTG-1_CRC0	Epithelial cells type1	1738
AGTTGGTGTAGCAAAT-1_CRC0	Epithelial cells type1	2071
CATCCACGTTGGAGGT-1_CRC0	Epithelial cells type1	1965
CCAATCCAGTGCGATG-1_CRC0	Epithelial cells type1	1523
CTCTGGTGTGGCGAAT-1_CRC0	Epithelial cells type1	1622
GTCACAAAGTGCTGCC-1_CRC0	Epithelial cells type1	1975
CTCGAAATCTTTAGGG-1_CRC0	Epithelial cells type1	1465
TTTACTGCACGAAGCA-1_CRC0	Epithelial cells type1	1271
GCCTCTAAGCCTCGTG-1_CRC0	Epithelial cells type1	2458
GTTCATTTCGTAGGTT-1_CRC0	Epithelial cells type1	2640
CGTGTAATCCGGCACA-1_CRC0	Epithelial cells type1	2488
AAAGCAAGTTATCACG-1_CRC0	Epithelial cells type1	1953
GAACCTACACGGTTTA-1_CRC0	Epithelial cells type1	2146
TCCCGATGTTATGCGT-1_CRC0	Epithelial cells type1	1943
TGGTTCCAGTATTGGA-1_CRC0	Epithelial cells type1	1923
GTCTTCGAGAAGGGTA-1_CRC0	Epithelial cells type1	1654
AGCCTAAAGCGTTGCC-1_CRC0	Epithelial cells type1	1768
CCTAAAGGTCTAGTCA-1_CRC0	Epithelial cells type1	2034
TTGGAACAGGCATTGG-1_CRC0	Epithelial cells type1	1978
ACTGCTCCACATTTCT-1_CRC0	Epithelial cells type1	1862
CTGATCCTCAGCGACC-1_CRC0	Epithelial cells type1	1952
TTCGGTCGTGTTTGGT-1_CRC0	Epithelial cells type1	1844
ATCATCTAGACGCACA-1_CRC0	Epithelial cells type1	1687
GTAACGTAGTGTACCT-1_CRC0	Epithelial cells type1	1901
ATCATCTAGGATCGCA-1_CRC0	Epithelial cells type1	1764
CGGACACCACAGGCCT-1_CRC0	Epithelial cells type1	1857
CGAGCACGTCGAGTTT-1_CRC0	Epithelial cells type1	1401
TAAGCGTAGCGTGAAC-1_CRC0	Epithelial cells type1	2289
CCGTACTGTCGAAAGC-1_CRC0	Epithelial cells type1	1995
ACTGAGTAGAAACGCC-1_CRC0	Epithelial cells type1	2199

GCTCCTAGTGACTACT-1_CRC0	Epithelial cells type1	2212
CGAACATTCTACCTGC-1_CRC0	Epithelial cells type1	1939
TCTGAGATCGTGTAGT-1_CRC0	Epithelial cells type1	1980
CACACCTGTGCACTTA-1_CRC0	Epithelial cells type1	2461
TGGCTGGCATCGATTG-1_CRC0	Epithelial cells type1	2648
TTAACTCTCGAACTGT-1_CRC0	Epithelial cells type1	1994
GCATGCGGTTTGCATG-1_CRC0	Epithelial cells type1	2003
GAGCAGACAAACGCGA-1_CRC0	Epithelial cells type1	1513
TGGCGCACATTATCTC-1_CRC0	Epithelial cells type1	1910
AAAGCAATCGCCAAAT-1_CRC0	Epithelial cells type1	2492
GCAATCATCATGGTCA-1_CRC0	Epithelial cells type1	1898
TGACGGCCACCGTTGG-1_CRC0	Epithelial cells type1	2271
CGCTGGAGTGACGCCT-1_CRC0	Epithelial cells type1	2158
GGACAGAAGCGTTCCG-1_CRC0	Epithelial cells type1	1785
CCCAATCCATCCTAGA-1_CRC0	Epithelial cells type1	1987
ACACCGGCAGCCTTTC-1_CRC0	Epithelial cells type1	1733
CGAATGTCATGCAACT-1_CRC0	Epithelial cells type1	2078
CCGTTCACACATTAGC-1_CRC0	Epithelial cells type1	1595
GCGACCAGTTCAACCA-1_CRC0	Epithelial cells type1	1884
TGGCCAGCAGAGTGTG-1_CRC0	Epithelial cells type1	1807
CGATCGGTCCTACAGA-1_CRC0	Epithelial cells type1	1571
TTTCCTCAGTGGGATC-1_CRC0	Epithelial cells type1	1727
CGAGCCAAGTCGAGTG-1_CRC0	Epithelial cells type1	1739
TGCCCATCACGGTAAG-1_CRC0	Epithelial cells type1	2544
CTACATTCAATGGATA-1_CRC0	Epithelial cells type1	2241
ACGGAGAGTACACCGC-1_CRC0	Epithelial cells type1	1897
ACTTTCAAGCCCAACC-1_CRC0	Epithelial cells type1	1683
ATAGACCAGCGACGTA-1_CRC0	Epithelial cells type1	1621
CAGGTGCTCTGCTGCT-1_CRC0	Epithelial cells type1	1697
CATGACAGTTGCGCAC-1_CRC0	Epithelial cells type1	1877
AAACCTGAGGTGATAT-1_CRC0	Epithelial cells type1	1672
CACTCCAGTCGCTTTC-1_CRC0	Epithelial cells type1	1709
CCTACCAAGAACAATC-1_CRC0	Epithelial cells type1	2502
TAGGCATTCTCATTCA-1_CRC0	Epithelial cells type1	2240
GTGCAGCGTTCCACTC-1_CRC0	Epithelial cells type1	1903
TGACTTTCAAAGTCAA-1_CRC0	Epithelial cells type1	1846
TAAGTGCGTCCGTCAG-1_CRC0	Epithelial cells type1	2299
AGGTCCGCAGCGATCC-1_CRC0	Epithelial cells type1	1918
CGACTTCGTTTGTTTC-1_CRC0	Epithelial cells type1	1912
ATCACGAAGAAGATTC-1_CRC0	Epithelial cells type1	1914
TGTGGTACAGTTCCCT-1_CRC0	Epithelial cells type1	1977
ATCCACCAGACTACAA-1_CRC0	Epithelial cells type1	1704
CGCGGTACACTTCGAA-1_CRC0	Epithelial cells type1	1984
TATCTCAGTCATATCG-1_CRC0	Epithelial cells type1	1692
TCAGCAAGTAGAGTGC-1_CRC0	Epithelial cells type1	1633
TGAGAGGGTAGGAGTC-1_CRC0	Epithelial cells type1	1890
CAGGTGCAGTAATCCC-1_CRC0	Epithelial cells type1	1234
TCAGGTAAGCTACCGC-1_CRC0	Epithelial cells type1	1987
CCATGTCAGCCTCGTG-1_CRC0	Epithelial cells type1	1787
ACGCAGCAGGTCATCT-1_CRC0	Epithelial cells type1	1967
CGTGTAATCCTTTACA-1_CRC0	Epithelial cells type1	1999

GATCGCGTCGCATGGC-1_CRC0	Epithelial cells type1	2143
GTTAAGCAGGGCTTGA-1_CRC0	Epithelial cells type1	1649
GTCCTCACATTAGCCA-1_CRC0	Epithelial cells type1	1566
AACTCAGAGCTAAACA-1_CRC0	Epithelial cells type1	1851
TGAGAGGAGCTAACAA-1_CRC0	Epithelial cells type1	1660
CGGAGCTAGACGACGT-1_CRC0	Epithelial cells type1	2319
GTTACAGCACTTGGAT-1_CRC0	Epithelial cells type1	1780
ACATCAGCATGTCCTC-1_CRC0	Epithelial cells type1	1255
AGGCCGTGTCGCATAT-1_CRC0	Epithelial cells type1	1500
CATATGGGTCCAGTAT-1_CRC0	Epithelial cells type1	1859
AGCCTAAAGGCATGGT-1_CRC0	Epithelial cells type1	2183
CCCTCCTAGCCACGCT-1_CRC0	Epithelial cells type1	2475
GCCAAATAGGCTCATT-1_CRC0	Epithelial cells type1	2160
TACCTATCATGCCCGA-1_CRC0	Epithelial cells type1	2102
CTTAGGACATAGTAAG-1_CRC0	Epithelial cells type1	2538
TACGGATGTGATAAGT-1_CRC0	Epithelial cells type1	1336
GTTTCTAGTTAAGACA-1_CRC0	Epithelial cells type1	2548
TGAGCATGTTCAGACT-1_CRC0	Epithelial cells type1	2300
AGCTCTCAGTTGTCGT-1_CRC0	Epithelial cells type1	1632
TGGTTAGCAACTGGCC-1_CRC0	Epithelial cells type1	2159
TAGAGCTAGGACAGCT-1_CRC0	Epithelial cells type1	1833
ACTTTCATCTTGAGAC-1_CRC0	Epithelial cells type1	1757
ATTCTACCACCGATAT-1_CRC0	Epithelial cells type1	1863
CTAAGACCAAAGGCGT-1_CRC0	Epithelial cells type1	2138
GAGGTGAGTTTGTTGG-1_CRC0	Epithelial cells type1	1918
CCTTTCTCACATTTCT-1_CRC0	Epithelial cells type1	1916
CGCGTTTTCCCAAGTA-1_CRC0	Epithelial cells type1	1904
TTGAACGCAGTCGTGC-1_CRC0	Epithelial cells type1	2353
GGCGACTTCGTTACAG-1_CRC0	Epithelial cells type1	1711
TCTGGAACATTTCAGG-1_CRC0	Epithelial cells type1	1668
TGGGAAGCAATAGAGT-1_CRC0	Epithelial cells type1	1539
TTCTTAGAGCCTTGAT-1_CRC0	Epithelial cells type1	1873
AACCATGGTCTAGTCA-1_CRC0	Epithelial cells type1	1678
TGTATTCTCGAATGGG-1_CRC0	Epithelial cells type1	1561
AACTCCCTCATCGCTC-1_CRC0	Epithelial cells type1	1901
TGCCCATTCACTATTC-1_CRC0	Epithelial cells type1	2438
TACCTTATCCCTAATT-1_CRC0	Epithelial cells type1	2393
CCACGGATCCCGACTT-1_CRC0	Epithelial cells type1	1667
GTCACGGCATGTTCCC-1_CRC0	Epithelial cells type1	2260
TGCACCTGTCTGGTCG-1_CRC0	Epithelial cells type1	2097
TGGCTGGGTCATATGC-1_CRC0	Epithelial cells type1	1493
CTAGCCTTCTCTAGGA-1_CRC0	Epithelial cells type1	2061
TCGTACCCACGAGAGT-1_CRC0	Epithelial cells type1	1924
ACACCAAGTTCGGGCT-1_CRC0	Epithelial cells type1	2184
AGCTCCTGTCTCATCC-1_CRC0	Epithelial cells type1	1822
CACCTTGCATCACAAC-1_CRC0	Epithelial cells type1	2393
GACGTGCGTCAAACTC-1_CRC0	Epithelial cells type1	1846
TGAAAGAAGAGTGAGA-1_CRC0	Epithelial cells type1	1924
GCTTCCACAAGTCATC-1_CRC0	Epithelial cells type1	2725
CTCGAGGAGGTACTCT-1_CRC0	Epithelial cells type1	2107
TCTATTGTCTACCAGA-1_CRC0	Epithelial cells type1	2260

TTATGCTCATCCGTGG-1_CRC0	Epithelial cells type1	2341
CTGATAGGTCAGCTAT-1_CRC0	Epithelial cells type1	1774
AAACCTGGTGCAACTT-1_CRC0	Epithelial cells type1	1815
AGCTCCTTCGGATGGA-1_CRC0	Epithelial cells type1	2018
ACGCAGCCAACACCCG-1_CRC0	Epithelial cells type1	2175
TTGACTTAGCGATTCT-1_CRC0	Epithelial cells type1	1783
TTATGCTCAACACCTA-1_CRC0	Epithelial cells type1	2235
CAGTAACAGGATATAC-1_CRC0	Epithelial cells type1	2235
TATCTCATCTGTCAAG-1_CRC0	Epithelial cells type1	2258
CGGACACTCACGGTTA-1_CRC0	Epithelial cells type1	2321
CGGCTAGGTGCTGTAT-1_CRC0	Epithelial cells type1	1991
CTCTACGGTGTTAAGA-1_CRC0	Epithelial cells type1	1721
GACAGAGCATTCTCAT-1_CRC0	Epithelial cells type1	2217
GGAGCAAAGTTAAGTG-1_CRC0	Epithelial cells type1	2482
CCCATACGTATGGTTC-1_CRC0	Epithelial cells type1	2280
TCTATTGAGAGTACAT-1_CRC0	Epithelial cells type1	2109
AGCGTCGAGTGGTAAT-1_CRC0	Epithelial cells type1	1865
GTCACGGAGCCAGTTT-1_CRC0	Epithelial cells type1	1570
TGCCAAAGTAGCTAAA-1_CRC0	Epithelial cells type1	1800
ACTGTCCCATTAACCG-1_CRC0	Epithelial cells type1	1757
GCTCCTATCACGACTA-1_CRC0	Epithelial cells type1	1579
TCCACACTCCTGTAGA-1_CRC0	Epithelial cells type1	1424
GTCACAAGTGGTAACG-1_CRC0	Epithelial cells type1	1570
GACAGAGCACATGGGA-1_CRC0	Epithelial cells type1	1485
TGTATTCGTCGAAAGC-1_CRC0	Epithelial cells type1	1302
CTACCCATCTTGAGGT-1_CRC0	Epithelial cells type1	1018
TACTCGCTCCCGACTT-1_CRC0	Epithelial cells type1	1237
GTCGGGTGTGCTTCTC-1_CRC0	Epithelial cells type1	1407
CCGGGATGTTCCTCCA-1_CRC0	Epithelial cells type1	1201
TCTTTCCGTAGCTAAA-1_CRC0	Epithelial cells type1	1915
CGTCACTGTTGAACTC-1_CRC0	Epithelial cells type1	1360
GGGAATGAGCCAGAAC-1_CRC0	Epithelial cells type1	1441
GTGCATATCAACGAAA-1_CRC0	Epithelial cells type1	1639
AGTGAGGAGTATTGGA-1_CRC0	Epithelial cells type1	1421
CCTTCCCCACTTCTGC-1_CRC0	Epithelial cells type1	1012
ACGATGTAGCGTTTAC-1_CRC0	Epithelial cells type1	1562
TGAGCATAGGCCCTTG-1_CRC0	Epithelial cells type1	1451
CGACCTTTCTTGTATC-1_CRC0	Epithelial cells type1	1057
TTAGTTCCAAGCTGAG-1_CRC0	Epithelial cells type1	1427
GAACATCCACGGTTTA-1_CRC0	Epithelial cells type1	1306
ACGGCCAAGACCCACC-1_CRC0	Epithelial cells type1	1402
GTGCGGTCATCCCATC-1_CRC0	Epithelial cells type1	1007
TCAGGATAGCCACTAT-1_CRC0	Epithelial cells type1	1472
TTTCCTCTCACTTCAT-1_CRC0	Epithelial cells type1	1250
AAAGTAGTCCTCAATT-1_CRC0	Epithelial cells type1	1249
TTTACTGTCACTTCAT-1_CRC0	Epithelial cells type1	1501
CAGAATCCAGCGTTCG-1_CRC0	Epithelial cells type1	413
CTGAAGTAGCCAACAG-1_CRC0	Epithelial cells type1	1347
TCAGGATGTGGTAACG-1_CRC0	Epithelial cells type1	763
CATGCCTCATATACGC-1_CRC0	Epithelial cells type1	1698
CTCGTACAGTTGCAGG-1_CRC0	Epithelial cells type1	1901

CATCGAACAATCAGAA-1_CRC0	Epithelial cells type1	1633
CGAGAAGCATCGACGC-1_CRC0	Epithelial cells type1	1983
TTAGTTCGTGGTTTCA-1_CRC0	Epithelial cells type1	1382
TGCGGGTCAAACTGTC-1_CRC0	Epithelial cells type1	1377
GATTCAGTCTTACCTA-1_CRC0	Epithelial cells type1	1862
TCAGGTAAGCCACGCT-1_CRC0	Epithelial cells type1	1321
GAGCAGACAGTACACT-1_CRC0	Epithelial cells type1	1707
TGCGCAGCAAGAGTCG-1_CRC0	Epithelial cells type1	1503
CTCGGGATCGGCGGTT-1_CRC0	Epithelial cells type1	1617
AATCGGTAGTGACATA-1_CRC0	Epithelial cells type1	1814
GTCAAGTAGTATCGAA-1_CRC0	Epithelial cells type1	1535
CATCAGAAGCCACGTC-1_CRC0	Epithelial cells type1	1857
CCTTCCCGTCGAGTTT-1_CRC0	Epithelial cells type1	1645
ACGTCAAGTGGTACAG-1_CRC0	Epithelial cells type1	1923
CGTGAGCGTGAGGGTT-1_CRC0	Epithelial cells type1	2575
ATTGGACCAACACCCG-1_CRC0	Epithelial cells type1	2847
CCAATCCGTTCGGGCT-1_CRC0	Epithelial cells type1	1527
CACACTCAGCGGATCA-1_CRC0	Epithelial cells type1	1520
TGACGGCGTCCGAATT-1_CRC0	Epithelial cells type1	1432
AGAATAGGTCTTGTCC-1_CRC0	Epithelial cells type1	2298
CAGTAACAGTTACGGG-1_CRC0	Epithelial cells type1	2482
CCTTACGCACTTCGAA-1_CRC0	Epithelial cells type1	1477
GTTTCTACAAGTTCTG-1_CRC0	Epithelial cells type1	1332
TTTATGCGTTCCTCCA-1_CRC0	Epithelial cells type1	1514
GTTCTCGGTGATGTCT-1_CRC0	Epithelial cells type1	1248
TACGGGCTCGTATCAG-1_CRC0	Epithelial cells type1	1173
AGCTCCTAGAAACCGC-1_CRC0	Epithelial cells type1	1822
AGACGTTAGATGTAAC-1_CRC0	Epithelial cells type1	1720
CTCACACAGAAGCCCA-1_CRC0	Epithelial cells type1	2119
TGACAACCAATGGAAT-1_CRC0	Epithelial cells type1	1729
CTGCGGACAGATCGGA-1_CRC0	Epithelial cells type1	1455
CAAGTTGTCCGCAAGC-1_CRC0	Epithelial cells type1	1220
ACGTCAAGTAAAGGAG-1_CRC0	Epithelial cells type1	1678
CAGGTGCGTATCTGCA-1_CRC0	Epithelial cells type1	1606
ATCTACTAGCGACGTA-1_CRC0	Epithelial cells type1	2074
TACGGATGTAAATGTG-1_CRC0	Epithelial cells type1	1795
ACTTACTGTCACCTAA-1_CRC0	Epithelial cells type1	1357
GTGCGGTCAAACCTAC-1_CRC0	Epithelial cells type1	1330
TCGCGAGGTAAATACG-1_CRC0	Epithelial cells type1	1128
GCTCCTACATGGGAAC-1_CRC0	Epithelial cells type1	1005
TTTATGCAGTCCGTAT-1_CRC0	Epithelial cells type1	864
ATCCACCTCGTTACGA-1_CRC0	Epithelial cells type1	1532
GTCAAGTTCCAACCAA-1_CRC0	Epithelial cells type1	1460
AGTCTTTAGACTAGAT-1_CRC0	Epithelial cells type1	1446
TTGTAGGGTGGCCCTA-1_CRC0	Epithelial cells type1	1407
AGACGTTAGACGACGT-1_CRC0	Epithelial cells type1	1384
GCAGTTAGTTGAACTC-1_CRC0	Epithelial cells type1	1186
GATTCAGTCTCGCTTG-1_CRC0	Epithelial cells type1	2411
GGTGCGTTCCTGCCAT-1_CRC0	Epithelial cells type1	2325
TAAGTGCAGTACGATA-1_CRC0	Epithelial cells type1	1485
TGGCGCAAGAATTGTG-1_CRC0	Epithelial cells type1	2381

TTTGCGCAGACGACGT-1_CRC0	Epithelial cells type1	2631
ACGGAGATCACAATGC-1_CRC0	Epithelial cells type1	1537
GTTCTCGAGAAACGCC-1_CRC0	Epithelial cells type1	1895
CCAATCCCAGACACTT-1_CRC0	Epithelial cells type1	1120
GTGCTTCCAGACGCCT-1_CRC0	Epithelial cells type1	1026
GATGCTACATAAGACA-1_CRC0	Epithelial cells type1	1461
TAAGTGCAGGAGCGAG-1_CRC0	Epithelial cells type1	1832
AGGGAGTCAACGATCT-1_CRC0	Epithelial cells type1	2009
CCTTCCCAGCTAGGCA-1_CRC0	Epithelial cells type1	1982
CCTACCAGTGCAGTAG-1_CRC0	Epithelial cells type1	2047
CTGCCTATCCGCAAGC-1_CRC0	Epithelial cells type1	1899
GGGTCTGTCTTAGCCC-1_CRC0	Epithelial cells type1	1576
AAATGCCCACCTATCC-1_CRC0	Epithelial cells type1	1857
GCTGCGAAGCATGGCA-1_CRC0	Epithelial cells type1	1265
AAGTCTGCACTAAGTC-1_CRC0	Epithelial cells type1	1389
CTGTTTATCTGAGGGA-1_CRC0	Epithelial cells type1	1647
GTATTCTCAGTGACAG-1_CRC0	Epithelial cells type1	1679
GATGAGGAGCGATATA-1_CRC0	Epithelial cells type1	1109
GGACAAGAGCTCCTCT-1_CRC0	Epithelial cells type1	1384
AACACGTGTTCTGGTA-1_CRC0	Epithelial cells type1	1175
GCGAGAACAGATCCAT-1_CRC0	Epithelial cells type1	1538
TCTGAGATCATGCTCC-1_CRC0	Epithelial cells type1	1914
GACTGCGCATTAGGCT-1_CRC0	Epithelial cells type1	1590
AAGTCTGAGGTAGCCA-1_CRC0	Epithelial cells type1	1129
GTCGGGTAGGGAACGG-1_CRC0	Epithelial cells type1	1107
GTCAAGTTCTGAGGGA-1_CRC0	Epithelial cells type1	1499
ATCATGGAGGAGTTGC-1_CRC0	Epithelial cells type1	1097
CGAGCCACAGAAGCAC-1_CRC0	Epithelial cells type1	1210
GTACGTACAGCTGGCT-1_CRC0	Epithelial cells type1	1441
GAATAAGGTGTGCGTC-1_CRC0	Epithelial cells type1	1232
CTCACACTCTGGTATG-1_CRC0	Epithelial cells type1	1814
CTTACCGAGGTACTCT-1_CRC0	Epithelial cells type1	1623
TAAGCGTAGCCAACAG-1_CRC0	Epithelial cells type1	1530
GCGAGAAGTATGCTTG-1_CRC0	Epithelial cells type1	1184
TCTTCGGTCAGCTGGC-1_CRC0	Epithelial cells type1	1340
GCTGCGAAGAGTACCG-1_CRC0	Epithelial cells type1	1152
AACACGTCATGGTTGT-1_CRC0	Epithelial cells type1	868
TCGCGAGGTTGTCGCG-1_CRC0	Epithelial cells type1	1306
CACACAACAAGGCTCC-1_CRC0	Epithelial cells type1	1558
TTTCCTCCAGCTCGCA-1_CRC0	Epithelial cells type1	1642
CCTTACGTCGGAGCAA-1_CRC0	Epithelial cells type1	1118
TCCACACTCCTCCTAG-1_CRC0	Epithelial cells type1	693
TGCTGCTTCTGTTGAG-1_CRC0	Epithelial cells type1	1289
GTCATTTAGTTAGGTA-1_CRC0	Epithelial cells type1	878
CAGGTGCAGGTGCTTT-1_CRC0	Epithelial cells type1	1108
TAGTTGGTCCTTGCCA-1_CRC0	Epithelial cells type1	1074
AAGACCTTCAGCCTAA-1_CRC0	Epithelial cells type1	1584
ATCATGGGTACTCTCC-1_CRC0	Epithelial cells type1	1650
GGGATGAGTCAGAATA-1_CRC0	Epithelial cells type1	1327
CCCAATCAGAGATGAG-1_CRC0	Epithelial cells type1	1409
ACTGTCCCACGTAAGG-1_CRC0	Epithelial cells type1	1096

CTTACCGAGCTCCCAG-1_CRC0	Epithelial cells type1	1142
GTTTCTAAGTCCCACG-1_CRC0	Epithelial cells type1	1140
GTCCTCACATGGGAAC-1_CRC0	Epithelial cells type1	1359
CGCGGTAGTAAGTTCC-1_CRC0	Epithelial cells type1	1129
GGGAATGCATAAGACA-1_CRC0	Epithelial cells type1	1442
CCTAGCTGTTGTCGCG-1_CRC0	Epithelial cells type1	2369
TTCTCCTGTCTGATCA-1_CRC0	Epithelial cells type1	2692
AAGTCTGTCCTTTCTC-1_CRC0	Epithelial cells type1	2434
GATTCAGGTAGGGTAC-1_CRC0	Epithelial cells type1	2144
CTACGTCGTGTCGCTG-1_CRC0	Epithelial cells type1	3000
CTGCCTAAGTCATCCA-1_CRC0	Epithelial cells type1	1725
CCACCTACACGCCAGT-1_CRC0	Epithelial cells type1	2086
CTGATAGGTGTAACGG-1_CRC0	Epithelial cells type1	1358
TACTCGCAGTACACCT-1_CRC0	Epithelial cells type1	1771
CGCTATCTCAAACGGG-1_CRC0	Epithelial cells type1	2182
TGTTCCGCAAACGTGG-1_CRC0	Epithelial cells type1	2183
TTCTCAAAGGTACTCT-1_CRC0	Epithelial cells type1	1260
GATTCAGCACGAAGCA-1_CRC0	Epithelial cells type1	1341
CGTTAGATCTCCTATA-1_CRC0	Epithelial cells type1	1558
GCGCCAATCATGCTCC-1_CRC0	Epithelial cells type1	1919
GTGGGTCAGGCCGAAT-1_CRC0	Epithelial cells type1	1555
CAGCAGCAGTCACGCC-1_CRC0	Epithelial cells type1	1540
CTCTAATGTACCGTAT-1_CRC0	Epithelial cells type1	1337
ACACCGGGTTACGCGC-1_CRC0	Epithelial cells type1	1573
CATCAAGCAATTCCTT-1_CRC0	Epithelial cells type1	1529
CATCGGGCAGCCTATA-1_CRC0	Epithelial cells type1	1892
CGATGGCGTGGTGTAG-1_CRC0	Epithelial cells type1	1294
CGTGTAAGTCTTGCGG-1_CRC0	Epithelial cells type1	1522
CCGTTCACATTACGAC-1_CRC0	Epithelial cells type1	2239
ACCCACTTCTGATTCT-1_CRC0	Epithelial cells type1	2035
GACTAACAGGCTCATT-1_CRC0	Epithelial cells type1	2356
AAACGGGGTTGGTAAA-1_CRC0	Epithelial cells type1	2629
GGAATAATCAGCGACC-1_CRC0	Epithelial cells type1	2014
AGGCCGTTCCAAATGC-1_CRC0	Epithelial cells type1	1920
CCTTACGTCTCGTATT-1_CRC0	Epithelial cells type1	2340
GCGCGATTCGTAGATC-1_CRC0	Epithelial cells type1	1631
TACTCATTCGCCTGTT-1_CRC0	Epithelial cells type1	1852
CCGTGGAAGGTGCAAC-1_CRC0	Epithelial cells type1	1479
CTCGTACTCAGCTTAG-1_CRC0	Epithelial cells type1	1838
TATCTCATCTTGCAAG-1_CRC0	Epithelial cells type1	2406
GACAGAGTCAGAGCTT-1_CRC0	Epithelial cells type1	1702
TTAGGACTCTCAAACG-1_CRC0	Epithelial cells type1	1908
ACACCCTTCAAAGTAG-1_CRC0	Epithelial cells type1	2280
TAGTTGGGTTCACGGC-1_CRC0	Epithelial cells type1	2348
GTACGTACAACTTGAC-1_CRC0	Epithelial cells type1	1726
GTTCGGGTCACATACG-1_CRC0	Epithelial cells type1	1828
AGCGTATCAACGATCT-1_CRC0	Epithelial cells type1	1384
TAGAGCTCATCCTAGA-1_CRC0	Epithelial cells type1	1782
GGAATAACACTTACGA-1_CRC0	Epithelial cells type1	2242
CTAAGACTCTCCCTGA-1_CRC0	Epithelial cells type1	1817
TACTTACCACAGATTC-1_CRC0	Epithelial cells type1	1778

ATGTGTGCACACCGAC-1_CRC0	Epithelial cells type1	1927
GTAACGTCATCGTCGG-1_CRC0	Epithelial cells type1	2255
CCTTCCCGTAAATGTG-1_CRC0	Epithelial cells type1	2275
ACAGCTAGTTTGCATG-1_CRC0	Epithelial cells type1	1964
GCGACCAAGATCCGAG-1_CRC0	Epithelial cells type1	2038
GTGGGTCCACATCCGG-1_CRC0	Epithelial cells type1	2572
CCTACACAGAACTGTA-1_CRC0	Epithelial cells type1	2588
GTAGGCCCAAATTGCC-1_CRC0	Epithelial cells type1	2157
GAACGGATCATGTCCC-1_CRC0	Epithelial cells type1	1578
TGGTTCCGTTTACTCT-1_CRC0	Epithelial cells type1	2030
CTACGTCTCCGAATGT-1_CRC0	Epithelial cells type1	1316
CTCGTACCAATGGAAT-1_CRC0	Epithelial cells type1	1528
ACTGAACAGACTGGGT-1_CRC0	Epithelial cells type1	1544
TAAGCGTCATGGTTGT-1_CRC0	Epithelial cells type1	1315
CAAGGCCAGAGAACAG-1_CRC0	Epithelial cells type1	1208
TATCAGGGTCAGAAGC-1_CRC0	Epithelial cells type1	1505
GATGAGGGTTCATGGT-1_CRC0	Epithelial cells type1	1450
ATCCGAATCAGTTAGC-1_CRC0	Epithelial cells type1	1943
GGGAATGAGGTTCCTA-1_CRC0	Epithelial cells type1	1525
CAGTAACGTCACAAGG-1_CRC0	Epithelial cells type1	1751
CTACATTCATGACGGA-1_CRC0	Epithelial cells type1	2018
GGATTACAGGACATTA-1_CRC0	Epithelial cells type1	2266
TCAATCTTCCACTCCA-1_CRC0	Epithelial cells type1	1809
AGCGTATCACGGCTAC-1_CRC0	Epithelial cells type1	1775
AGTCTTTGTCTCTTTA-1_CRC0	Epithelial cells type1	2086
CTAAGACCAAGCCGCT-1_CRC0	Epithelial cells type1	1621
GATCAGTGTCGCATCG-1_CRC0	Epithelial cells type1	1617
TATGCCCGTTCCTCCA-1_CRC0	Epithelial cells type1	1373
ACTGAACTCAACACTG-1_CRC0	Epithelial cells type1	1814
TACACGAGTTATCACG-1_CRC0	Epithelial cells type1	1548
AATCCAGAGTTCGCAT-1_CRC0	Epithelial cells type1	1581
TCTTCGGTCCATGAGT-1_CRC0	Epithelial cells type1	1311
CGGAGCTCATCACGTA-1_CRC0	Epithelial cells type1	1720
GACCTGGCATGAGCGA-1_CRC0	Epithelial cells type1	1389
TGGGAAGAGTCTCCTC-1_CRC0	Epithelial cells type1	1857
ATGCGATGTACAGTGG-1_CRC0	Epithelial cells type1	1517
TAGCCGGAGCTGTCTA-1_CRC0	Epithelial cells type1	1804
AAACCTGAGTACACCT-1_CRC0	Epithelial cells type1	2095
GGTGCGTTCAGCGACC-1_CRC0	Epithelial cells type1	2190
GGACAGATCGAATGCT-1_CRC0	Epithelial cells type1	2212
TAAGAGAGTACCGAGA-1_CRC0	Epithelial cells type1	2008
GTTCGGGCATGGTCAT-1_CRC0	Epithelial cells type1	1667
TGCGTGGGTAGGGTAC-1_CRC0	Epithelial cells type1	1692
TTTGCGCTCTTGACGA-1_CRC0	Epithelial cells type1	1823
CATGACACATGACGGA-1_CRC0	Epithelial cells type1	1888
TGTGGTAAGAAACGAG-1_CRC0	Epithelial cells type1	1996
CGCTTCACAGACAAGC-1_CRC0	Epithelial cells type1	1972
CGGTTAAAGCTAGGCA-1_CRC0	Epithelial cells type1	1744
ACACCCTAGCGCCTCA-1_CRC0	Epithelial cells type1	1782
TCATTTGTCTTACCGC-1_CRC0	Epithelial cells type1	1607
CTCAGAATCAACCATG-1_CRC0	Epithelial cells type1	2081

GTCCTCAGTCATGCCG-1_CRC0	Epithelial cells type1	1557
ACGGCCACATCTGGTA-1_CRC0	Epithelial cells type1	1361
ACAGCTAAGTGATCGG-1_CRC0	Epithelial cells type1	1280
TGCGCAGGTAAGTGGC-1_CRC0	Epithelial cells type1	1437
AGTCTTTGTCTTGATG-1_CRC0	Epithelial cells type1	1183
GTCAAGTGTACGAAAT-1_CRC0	Epithelial cells type1	914
CATTCGCAGTGAAGAG-1_CRC0	Epithelial cells type1	1119
GCTCCTATCTGCTGCT-1_CRC0	Epithelial cells type1	1585
AAACCTGCAGCTGGCT-1_CRC0	Epithelial cells type1	1485
GATGAGGGTGTTCTTT-1_CRC0	Epithelial cells type1	1582
GTGGGTCCAGCTTCGG-1_CRC0	Epithelial cells type1	1715
GTGCATAAGGCATTGG-1_CRC0	Epithelial cells type1	1234
TCTTCGGTCGTTACGA-1_CRC0	Epithelial cells type1	1221
GAACGGATCAACACGT-1_CRC0	Epithelial cells type1	1382
GCGCGATGTTCCACGG-1_CRC0	Epithelial cells type1	1712
AACTCCCCAGACAGGT-1_CRC0	Epithelial cells type1	1504
TCATTTGTCCGCATCT-1_CRC0	Epithelial cells type1	1596
TGGGCGTAGTGGGATC-1_CRC0	Epithelial cells type1	1268
GGCGACTAGCGGATCA-1_CRC0	Epithelial cells type1	1133
AAGCCGCAGCTAACTC-1_CRC0	Epithelial cells type1	1007
TACGGTACATTGAGCT-1_CRC0	Epithelial cells type1	993
TTCTCCTCATATACGC-1_CRC0	Epithelial cells type1	1575
GCTTGAAAGTTGCAGG-1_CRC0	Epithelial cells type1	1258
GTTCGGGGTGTTGGGA-1_CRC0	Epithelial cells type1	1595
AGTGTCATCAAGATCC-1_CRC0	Epithelial cells type1	1288
GATCAGTGTAAAGGAG-1_CRC0	Epithelial cells type1	1138
ATTTCTGTCTTGTACT-1_CRC0	Epithelial cells type1	1213
TGACTTTCAGACGTAG-1_CRC0	Epithelial cells type1	1090
GACTACACAACACCCG-1_CRC0	Epithelial cells type1	1053
CACACTCTCCATTCTA-1_CRC0	Epithelial cells type1	1193
CTCAGAATCAGCTCTC-1_CRC0	Epithelial cells type1	1079
AAGGAGCAGTGACTCT-1_CRC0	Epithelial cells type1	1766
GGACATTAGTCATCCA-1_CRC0	Epithelial cells type1	1485
TGAGCCGTCGCCCTTA-1_CRC0	Epithelial cells type1	1330
CGGTTAAGTGAAGGCT-1_CRC0	Epithelial cells type1	2097
CATCGAATCCTGCAGG-1_CRC0	Epithelial cells type1	1087
GTAGGCCAGGAACTGC-1_CRC0	Epithelial cells type1	1388
AGAGCGATCGCTAGCG-1_CRC0	Epithelial cells type1	1221
TACTCGCGTACGCACC-1_CRC0	Epithelial cells type1	1470
AACACGTGTCTGCAAT-1_CRC0	Epithelial cells type1	1676
TTGAACGGTTCCACAA-1_CRC0	Epithelial cells type1	1862
AACTCAGTCTTAACCT-1_CRC0	Epithelial cells type1	1475
CTCGGGAAGAGCTGGT-1_CRC0	Epithelial cells type1	1180
TCGTAGACAGTGGAGT-1_CRC0	Epithelial cells type1	1660
ATTACTCGTCAGATAA-1_CRC0	Epithelial cells type1	1738
GCGCGATAGCAATATG-1_CRC0	Epithelial cells type1	1878
GTGCATAGTAATCACC-1_CRC0	Epithelial cells type1	1816
CCTATTAAGATAGGAG-1_CRC0	Epithelial cells type1	1700
ATAAGAGTCAAACCAC-1_CRC0	Epithelial cells type1	1556
GCTGCTTTCGTTTAGG-1_CRC0	Epithelial cells type1	1668
GGACAGAAGTCTCCTC-1_CRC0	Epithelial cells type1	2244

GTCTTCGTCTGTCTAT-1_CRC0	Epithelial cells type1	1609
CTGGTCTAGTGGCACA-1_CRC0	Epithelial cells type1	2041
AGCGGTCCATCCCACT-1_CRC0	Epithelial cells type1	2446
GCTTGAATCCATTCTA-1_CRC0	Epithelial cells type1	1378
CCCATACGTACTTAGC-1_CRC0	Epithelial cells type1	2140
ACAGCTACAGGGTTAG-1_CRC0	Epithelial cells type1	1314
AGATCTGGTGAAGGCT-1_CRC0	Epithelial cells type1	1794
GAGCAGATCCTGCAGG-1_CRC0	Epithelial cells type1	1601
GCTCCTACAGCTGCTG-1_CRC0	Epithelial cells type1	1612
GCATACAGTTTGACAC-1_CRC0	Epithelial cells type1	3873
TCGAGGCCACCGAATT-1_CRC0	Epithelial cells type1	1855
GCGCCAAGTATTACCG-1_CRC0	Epithelial cells type1	2128
AACACGTAGCGGATCA-1_CRC0	Epithelial cells type1	1900
CGAGAAGGTGGCAAAC-1_CRC0	Epithelial cells type1	1815
TTGCCGTCACTTCGAA-1_CRC0	Epithelial cells type1	1510
ACATACGGTGCGGTAA-1_CRC0	Epithelial cells type1	1357
CGCGGTAGTTCTGGTA-1_CRC0	Epithelial cells type1	1383
GATGAGGAGCGTGTCC-1_CRC0	Epithelial cells type1	1720
TCACAAGGTAGAGCTG-1_CRC0	Epithelial cells type1	1346
GAATAAGCAGGATCGA-1_CRC0	Epithelial cells type1	1768
TCGAGGCCAGTATAAG-1_CRC0	Epithelial cells type1	2152
ATGCGATGTGGTTTCA-1_CRC0	Epithelial cells type1	2275
GTCGGGTGTTCCCTTG-1_CRC0	Epithelial cells type1	1978
AGCATACCAGTTTACG-1_CRC0	Epithelial cells type1	1821
GACAGAGCAGTCAGCC-1_CRC0	Epithelial cells type1	1548
CACATAGCACCAACCG-1_CRC0	Epithelial cells type1	2008
CTTGGCTCAATAACGA-1_CRC0	Epithelial cells type1	1450
AACGTTGGTGAGGCTA-1_CRC0	Epithelial cells type1	2145
CATTATCTCAGTTAGC-1_CRC0	Epithelial cells type1	1840
CGATTGATCACAAACC-1_CRC0	Epithelial cells type1	1752
AGAGCGAAGGCTAGCA-1_CRC0	Epithelial cells type1	2038
GCGAGAAGTAAGGGAA-1_CRC0	Epithelial cells type1	1940
GCGCCAATCGAGAACG-1_CRC0	Epithelial cells type1	1341
ACACTGATCTGAGGGA-1_CRC0	Epithelial cells type1	2248
CCATTCGCACGGACAA-1_CRC0	Epithelial cells type1	1706
CATTCGCGTGTTGGGA-1_CRC0	Epithelial cells type1	2446
TATGCCCCAGTGACAG-1_CRC0	Epithelial cells type1	2398
AGCCTAATCTCGCTTG-1_CRC0	Epithelial cells type1	1997
GACGGCTAGATGTAAC-1_CRC0	Epithelial cells type1	1887
CGAACATGTCTGCGGT-1_CRC0	Epithelial cells type1	1825
CGTCAGGCAGCGTTCG-1_CRC0	Epithelial cells type1	1482
CTAAGACGTAGAAGGA-1_CRC0	Epithelial cells type1	1496
CCTACCAGTGGCAAAC-1_CRC0	Epithelial cells type1	1242
TTCTTAGGTTGGTGGA-1_CRC0	Epithelial cells type1	1691
AGAGTGGTCCCATTAT-1_CRC0	Epithelial cells type1	1651
GGACAGAGTACCCAAT-1_CRC0	Epithelial cells type1	1768
CGCTGGACAATAGCAA-1_CRC0	Epithelial cells type1	1629
CAAGAAACACCCATTC-1_CRC0	Epithelial cells type1	1835
GTGCAGCAGCGTAATA-1_CRC0	Epithelial cells type1	1784
GGGCATCTCCCTCAGT-1_CRC0	Epithelial cells type1	1622
TGGACGCAGCACGCCT-1_CRC0	Epithelial cells type1	1626

AGTCTTTGTCGTCTTC-1_CRC0	Epithelial cells type1	1502
CCGTACTGTTCCGGCA-1_CRC0	Epithelial cells type1	1368
TTTGTCACAGCCTATA-1_CRC0	Epithelial cells type1	1535
ACTGAACGTACCGTAT-1_CRC0	Epithelial cells type1	1499
ATTGGACAGCAAATCA-1_CRC0	Epithelial cells type1	1569
GTTCGGGTCCAGTATG-1_CRC0	Epithelial cells type1	1792
TACTTGTGTACAAGTA-1_CRC0	Epithelial cells type1	1973
ATAACGCCAACAACCT-1_CRC0	Epithelial cells type1	2154
CCTTTCTCATCGATGT-1_CRC0	Epithelial cells type1	1964
CTGCTGTGTACAAGTA-1_CRC0	Epithelial cells type1	1674
TGCCCATCAAGTCTGT-1_CRC0	Epithelial cells type1	1616
CGAGAAGAGGCCCTCA-1_CRC0	Epithelial cells type1	1896
CTCGAGGCAGGGAGAG-1_CRC0	Epithelial cells type1	1890
GGGCACTCACCAGTTA-1_CRC0	Epithelial cells type1	5652
ATGCGATTCCAGATCA-1_CRC1	Epithelial cells type1	6111
GCTGCGACAGCCAATT-1_CRC0	Epithelial cells type1	2464
ATCCACCGTTCGTTGA-1_CRC0	Epithelial cells type1	2533
CGTGTAAGTTCTGTTT-1_CRC0	Epithelial cells type1	2460
AGGGATGTCGATGAGG-1_CRC0	Epithelial cells type1	2319
TGTCCCACAAGGGTCA-1_CRC0	Epithelial cells type1	1952
GATCGCGTCGTCCAGG-1_CRC0	Epithelial cells type1	2458
TACTTACAGCGGATCA-1_CRC0	Epithelial cells type1	2164
CAGCCGATCAAGATCC-1_CRC0	Epithelial cells type1	1945
TGTATTCTCTTCCTTC-1_CRC0	Epithelial cells type1	2388
CTGAAACAGATCCTGT-1_CRC0	Epithelial cells type1	2458
AATCCAGGTCGTCTTC-1_CRC0	Epithelial cells type1	1577
GTCACGGCAGTTAACC-1_CRC0	Epithelial cells type1	2042
ACTGTCCCACCCAGTG-1_CRC0	Epithelial cells type1	1414
ACGCCGATCGTACGGC-1_CRC0	Epithelial cells type1	1380
GCGAGAACAATTCCTT-1_CRC0	Epithelial cells type1	1772
TTGTAGGAGAAGCCCA-1_CRC0	Epithelial cells type1	2161
CAGCATAGTGTAATGA-1_CRC0	Epithelial cells type1	2347
TACCTTAAGTACGTTC-1_CRC0	Epithelial cells type1	1888
CATATGGCAATAACGA-1_CRC0	Epithelial cells type1	2086
CTCTACGAGCTTTGGT-1_CRC0	Epithelial cells type1	2294
CGATTGAAGTCTCCTC-1_CRC0	Epithelial cells type1	2744
CTCTGGTCATATACCG-1_CRC0	Epithelial cells type1	3123
ACGCCGACAAGCTGTT-1_CRC0	Epithelial cells type1	2768
TTATGCTCAAAGAATC-1_CRC0	Epithelial cells type1	2245
CGTGTAATCTCCCTGA-1_CRC0	Epithelial cells type1	2391
AAGACCTCATCCCACT-1_CRC0	Epithelial cells type1	2392
AGGCCGTAGCACACAG-1_CRC0	Epithelial cells type1	1988
GCTTCCAGTCTTTCAT-1_CRC0	Epithelial cells type1	1691
CGTCTACAGCCACGTC-1_CRC0	Epithelial cells type1	2124
GAGCAGATCTGCGGCA-1_CRC0	Epithelial cells type1	1761
CATGCCTCACAACGCC-1_CRC0	Epithelial cells type1	2181
ACCCACTGTATGGTTC-1_CRC0	Epithelial cells type1	2003
GGGAATGTCCGCGGTA-1_CRC0	Epithelial cells type1	2926
GTACGTAAGTCAATAG-1_CRC1	Epithelial cells type1	1319
GACTGCGTCAGGTTCA-1_CRC1	Epithelial cells type1	1504
GCGCGATCAGCCAATT-1_CRC1	Epithelial cells type1	1990

ACAGCTAGTACTCGCG-1_CRC1	Epithelial cells type1	2839
AGTCTTTCAGCTCGCA-1_CRC1	Epithelial cells type1	2898
CCTATTACACTGTGTA-1_CRC1	Epithelial cells type1	2291
GTACGTAAGGTCATCT-1_CRC1	Epithelial cells type1	1681
CGGACGTGTACCTACA-1_CRC1	Epithelial cells type1	2767
ACGGCCACAGCCAGAA-1_CRC1	Epithelial cells type1	1037
AGGGATGAGGTCGGAT-1_CRC1	Epithelial cells type1	1429
ATCACGATCCAGAAGG-1_CRC1	Epithelial cells type1	1309
GTCGGGTAGAGCTGCA-1_CRC1	Epithelial cells type1	1635
GGATTACCATTCCTGC-1_CRC1	Epithelial cells type1	2446
GCACTCTCAAGCGAGT-1_CRC1	Epithelial cells type1	1765
CATGCCTCAATGAATG-1_CRC1	Epithelial cells type1	908
TAGGCATTCTTGGGTA-1_CRC1	Epithelial cells type1	1175
ATGAGGGTCAGCATGT-1_CRC1	Epithelial cells type1	1263
CTCGGAGCAGCCAATT-1_CRC1	Epithelial cells type1	966
AATCGGTGTTCATGGT-1_CRC1	Epithelial cells type1	976
TTCGGTCAGTATCTCG-1_CRC1	Epithelial cells type1	1144
GACCAATTCATCGCTC-1_CRC1	Epithelial cells type1	1379
GGATTACCAAGAGTCG-1_CRC1	Epithelial cells type1	816
ACGGGCTGTCAGAGGT-1_CRC1	Epithelial cells type1	1758
CAACTAGAGAGACGAA-1_CRC1	Epithelial cells type1	1558
TTAGGCACACGAAATA-1_CRC1	Epithelial cells type1	909
ACCTTTATCTCTGCTG-1_CRC1	Epithelial cells type1	1592
AACTCAGTCGAACGGA-1_CRC1	Epithelial cells type1	1072
CGGACGTAGCGACGTA-1_CRC1	Epithelial cells type1	767
CTGCTGTTCTATCGCC-1_CRC1	Epithelial cells type1	969
CATCAGACATTAGGCT-1_CRC1	Epithelial cells type1	1145
TGAGCCGCATGGGAAC-1_CRC1	Epithelial cells type1	916
GGCTGGTAGTAGTGCG-1_CRC1	Epithelial cells type1	1119
GTGTTAGTCGAATGCT-1_CRC1	Epithelial cells type1	1329
AAACGGGGTGCACGAA-1_CRC1	Epithelial cells type1	1531
TATGCCCGTCCCGACA-1_CRC1	Epithelial cells type1	1804
AGTTGGTTCGTTTAGG-1_CRC1	Epithelial cells type1	730
CTGTTTAAGGTCATCT-1_CRC1	Epithelial cells type1	1074
GACTAACTCAGTCCCT-1_CRC1	Epithelial cells type1	1474
CCCATACAGTCAAGCG-1_CRC1	Epithelial cells type1	1386
GACGGCTTCGGACAAG-1_CRC1	Epithelial cells type1	1235
GCTCTGTTCTTTCCTC-1_CRC1	Epithelial cells type1	1056
TATGCCCCAGCTGCAC-1_CRC1	Epithelial cells type1	1374
TCGCGTTGTTGGACCC-1_CRC1	Epithelial cells type1	1359
CCGGTAGAGTCGTTTG-1_CRC1	Epithelial cells type1	1524
ACTGAACGTAGAGTGC-1_CRC1	Epithelial cells type1	1597
TCTTCGGGTCCGTCAG-1_CRC1	Epithelial cells type1	1175
CAGAGAGTCCGTCAAA-1_CRC1	Epithelial cells type1	2035
TCCACACAGGTGATAT-1_CRC1	Epithelial cells type1	1989
TGGCGCAGTGGGTCAA-1_CRC1	Epithelial cells type1	1613
AGCATACGTTCTGGTA-1_CRC1	Epithelial cells type1	1335
GATTCAGAGACCTTTG-1_CRC1	Epithelial cells type1	1533
ACGAGGACATCGGGTC-1_CRC1	Epithelial cells type1	1222
CTACACCCACTCTGTC-1_CRC1	Epithelial cells type1	1504
CGCGGTAAGTAGGCCA-1_CRC1	Epithelial cells type1	1803

AGCATACGTCTCTCTG-1_CRC1	Epithelial cells type1	1242
ACACTGAAGAGTCTGG-1_CRC1	Epithelial cells type1	1445
CAGAGAGAGTGCCAGA-1_CRC1	Epithelial cells type1	1106
GACGGCTAGCTAGCCC-1_CRC1	Epithelial cells type1	1459
GCATGATCATGCGCAC-1_CRC1	Epithelial cells type1	904
GACGGCTTCTTCATGT-1_CRC1	Epithelial cells type1	810
CGATGGCAGGCAGGTT-1_CRC1	Epithelial cells type1	841
GCTCCTAGTACAGACG-1_CRC1	Epithelial cells type1	953
GACGCGTGTCGCCATG-1_CRC1	Epithelial cells type1	1905
GCTGGGTTCGCAAGCC-1_CRC1	Epithelial cells type1	1203
TTTCCTCAGGGATCTG-1_CRC1	Epithelial cells type1	972
CGCGGTACAGCGAACA-1_CRC1	Epithelial cells type1	1231
CGCTATCCATACAGCT-1_CRC1	Epithelial cells type1	885
CGTTGGGAGACTGGGT-1_CRC1	Epithelial cells type1	737
GACGGCTCAGGTCTCG-1_CRC1	Epithelial cells type1	1939
GTTCTCGGTAATTGGA-1_CRC1	Epithelial cells type1	1110
CCTAAAGTCTATCGCC-1_CRC1	Epithelial cells type1	758
CTGTTTACACTGTTAG-1_CRC1	Epithelial cells type1	755
TTCGAAGAGACAGACC-1_CRC1	Epithelial cells type1	1209
CGAGCACGTCGGCACT-1_CRC1	Epithelial cells type1	978
TCGCGAGCAGCTGCTG-1_CRC1	Epithelial cells type1	1007
TCTCATATCCGCGGTA-1_CRC1	Epithelial cells type1	628
TGGCCAGTCTTGCATT-1_CRC1	Epithelial cells type1	633
CATATGGGTAGCTTGT-1_CRC1	Epithelial cells type1	484
CTCAGAAAGATGCCTT-1_CRC1	Epithelial cells type1	709
CGACTTCCAACTGCGC-1_CRC1	Epithelial cells type1	806
CTAAGACGTCCGAAGA-1_CRC1	Epithelial cells type1	889
GTGCGGTGTATCACCA-1_CRC1	Epithelial cells type1	709
CGCGTTTGTTCGCTAA-1_CRC1	Epithelial cells type1	1116
AGTTGGTGTTTGGCGC-1_CRC1	Epithelial cells type1	588
GACGCGTGTGCACTTA-1_CRC1	Epithelial cells type1	736
CATTATCTCACGGTTA-1_CRC1	Epithelial cells type1	1099
TTTATGCGTTCCAACA-1_CRC1	Epithelial cells type1	1249
TCTTTCCCATCAGTAC-1_CRC1	Epithelial cells type1	1105
GTGCTTCCACGAGGTA-1_CRC1	Epithelial cells type1	1069
TCAGATGGTAGAGCTG-1_CRC1	Epithelial cells type1	806
TTTGTCATCGATAGAA-1_CRC1	Epithelial cells type1	1099
ATCTACTTCGCTGATA-1_CRC1	Epithelial cells type1	968
AGTTGGTTCCTATGTT-1_CRC1	Epithelial cells type1	678
TTCGGTCTCGAATGCT-1_CRC1	Epithelial cells type1	901
AAGGAGCAGGCTAGGT-1_CRC1	Epithelial cells type1	785
AGCATACTCTGCGACG-1_CRC1	Epithelial cells type1	577
CCGGTAGTCTTAGCCC-1_CRC1	Epithelial cells type1	665
GGCGACTTCCTGCAGG-1_CRC1	Epithelial cells type1	1118
AACTCAGGTCTCTTAT-1_CRC1	Epithelial cells type1	1035
CTCTGGTGTAGGACAC-1_CRC1	Epithelial cells type1	1458
TGGGCGTGTTGTGGAG-1_CRC1	Epithelial cells type1	1345
AGGCCACAGTGCCAGA-1_CRC1	Epithelial cells type1	1238
GGGCACTTCATCACCC-1_CRC1	Epithelial cells type1	1988
GAACCTATCGACGGAA-1_CRC1	Epithelial cells type1	1712
TGCTACCCAATCTGCA-1_CRC1	Epithelial cells type1	1650

CCTCAGTCAGCTGCAC-1_CRC1	Epithelial cells type1	1702
GCGCAACGTTCGTCTC-1_CRC1	Epithelial cells type1	1326
CGTAGGCCATTCACTT-1_CRC1	Epithelial cells type1	1515
CTCGGAGCAGGTGGAT-1_CRC1	Epithelial cells type1	1220
GCTGCTTGTACCGAGA-1_CRC1	Epithelial cells type1	1195
TAGCCGGCATTAGCCA-1_CRC1	Epithelial cells type1	1542
TTCTCAATCTAACTGG-1_CRC1	Epithelial cells type1	1230
GTAGTCATCCTCATTA-1_CRC1	Epithelial cells type1	1367
CGCCAAGCAGTCACTA-1_CRC1	Epithelial cells type1	1038
CGCTTCACACGGTTTA-1_CRC1	Epithelial cells type1	1038
TAGACCAGTCAAAGAT-1_CRC1	Epithelial cells type1	1146
AAAGATGGTAAACACA-1_CRC1	Epithelial cells type1	1803
TGACGGCTCCAGTAGT-1_CRC1	Epithelial cells type1	1780
AGAATAGCATTGGCGC-1_CRC1	Epithelial cells type1	1156
CCGTTCATCCGAGCCA-1_CRC1	Epithelial cells type1	848
CCTAGCTTCCGTTGTC-1_CRC1	Epithelial cells type1	1299
GATCGTACACTGCCAG-1_CRC1	Epithelial cells type1	1658
GCGCAACGTTTGACAC-1_CRC1	Epithelial cells type1	1203
GGGAGATGTGACCAAG-1_CRC1	Epithelial cells type1	1308
AACACGTAGGATGGAA-1_CRC1	Epithelial cells type1	3785
CCATGTCCACCACCAG-1_CRC1	Epithelial cells type1	1766
TAGACCAGTGGCTCCA-1_CRC1	Epithelial cells type1	1352
AGGGAGTAGTGAAGTT-1_CRC1	Epithelial cells type1	1225
TACTCATAGTGGCACA-1_CRC1	Epithelial cells type1	1360
GGCGTGTTCAACGGCC-1_CRC1	Epithelial cells type1	1831
AAAGATGCAATGGTCT-1_CRC1	Epithelial cells type1	1116
GACTACAGTGGTCCGT-1_CRC1	Epithelial cells type1	1099
AGCATACAGGCGACAT-1_CRC1	Epithelial cells type1	1024
CCCAGTTGTATGAATG-1_CRC1	Epithelial cells type1	720
GACTAACTCAACTCTT-1_CRC1	Epithelial cells type1	838
ATTGGACGTAGGCTGA-1_CRC1	Epithelial cells type1	994
CTGCGGACAGCGAACA-1_CRC1	Epithelial cells type1	747
TTAGTTCGTGCACGAA-1_CRC1	Epithelial cells type1	1599
CTCTGGTGTCGCTTTC-1_CRC1	Epithelial cells type1	620
GCTCTGTGTTCCATGA-1_CRC1	Epithelial cells type1	535
ACCTTTACATCGATGT-1_CRC1	Epithelial cells type1	878
ATGAGGGTCGCCTGAG-1_CRC1	Epithelial cells type1	912
CTGATAGTCGGCCGAT-1_CRC1	Epithelial cells type1	888
ATCTGCCCAACACCTA-1_CRC1	Epithelial cells type1	1016
TCGTAGATCCCTCAGT-1_CRC1	Epithelial cells type1	985
TCGAGGCAGGCTCTTA-1_CRC1	Epithelial cells type1	1149
CGTGTAAAGAACAATC-1_CRC1	Epithelial cells type1	962
TCTGGAAAGCTTCGCG-1_CRC1	Epithelial cells type1	1092
CGTCCATGTGATGTGG-1_CRC1	Epithelial cells type1	1305
ATTGGACCATTGCGGC-1_CRC1	Epithelial cells type1	1307
CTAGCCTGTAGCTCCG-1_CRC1	Epithelial cells type1	1171
TTTACTGTCCTCATTA-1_CRC1	Epithelial cells type1	1061
GCGCGATCAGACAGGT-1_CRC2	Epithelial cells type1	1668
CGCGGTACAATAGCAA-1_CRC2	Epithelial cells type1	1680
GGATGTTCAGTATGCT-1_CRC2	Epithelial cells type1	1012
GTGGGTCTCTCAAGTG-1_CRC2	Epithelial cells type1	955

CCACGGAAGGGCTCTC-1_CRC3	Epithelial cells type1	1258
TGTCCCATCCTCGCAT-1_CRC3	Epithelial cells type1	1210
CACATAGAGAGGGATA-1_CRC1	Epithelial cells type1	1168
ACTGAGTAGCTAGTTC-1_CRC2	Epithelial cells type1	1338
AAACCTGCATTTGCTT-1_CRC2	Epithelial cells type1	1523
CAAGATCCACCCATGG-1_CRC3	Epithelial cells type1	1097
CACACAAGTCTAGGTT-1_CRC3	Epithelial cells type1	1221
GGGAATGGTCCAGTGC-1_CRC2	Epithelial cells type1	610
TCAGGATAGGGCTTCC-1_CRC3	Epithelial cells type1	687
AAGGCAGAGGGATCTG-1_CRC3	Epithelial cells type1	879
TCATTTGCAATGAATG-1_CRC2	Epithelial cells type1	519
TCTCTAACAATCACAC-1_CRC2	Epithelial cells type1	309
ATTTCTGTCACCATAG-1_CRC3	Epithelial cells type1	442
GCTTGAAGTCGCTTTC-1_CRC2	Epithelial cells type1	573
AGCTCTCGTATCTGCA-1_CRC3	Epithelial cells type1	387
TTCTCCTTCGATCCCT-1_CRC3	Epithelial cells type1	481
CACCAGGAGCTAACTC-1_CRC2	Epithelial cells type1	149
CATCAAGCAATAACGA-1_CRC2	Epithelial cells type1	100
ACTGATGAGGCTATCT-1_CRC3	Epithelial cells type1	228
ATAGACCGTCCGAAGA-1_CRC2	Epithelial cells type1	588
GTCTCGTCAGCGTCCA-1_CRC2	Epithelial cells type1	642
CTCGGGACACGTAAGG-1_CRC3	Epithelial cells type1	648
GACTGCGCAGTCGTGC-1_CRC3	Epithelial cells type1	779
ACGCCGAGTGTGCGTC-1_CRC2	Epithelial cells type1	812
CAGCCGACAGCGTCCA-1_CRC2	Epithelial cells type1	762
ACACCGGGTTGGTAAA-1_CRC3	Epithelial cells type1	1127
ATCTGCCTCACAAACC-1_CRC3	Epithelial cells type1	686
CGAGCCATCCCATTAT-1_CRC2	Epithelial cells type1	514
GTAACGTTCCGAAGAG-1_CRC3	Epithelial cells type1	672
CGATCGGAGACTACAA-1_CRC2	Epithelial cells type1	975
TTCCCAGTCACCGGGT-1_CRC2	Epithelial cells type1	734
TTGCGTCTCGGTCCGA-1_CRC2	Epithelial cells type1	685
GTAGGCCAGTACGTAA-1_CRC3	Epithelial cells type1	904
CACAGTAAGCGTGAGT-1_CRC2	Epithelial cells type1	913
CTCGTCAGTCGACTGC-1_CRC2	Epithelial cells type1	923
GCGCGATGTTGTGGCC-1_CRC3	Epithelial cells type1	1177
AAGACCTGTCTTCTCG-1_CRC2	Epithelial cells type1	894
CGCTGGACAGGATCGA-1_CRC2	Epithelial cells type1	370
ACGTCAAAGTACGATA-1_CRC2	Epithelial cells type1	1042
GACGTTATCTGGCGTG-1_CRC0	Epithelial cells type1	965
TCTGAGATCAGGCCCA-1_CRC2	Epithelial cells type1	1182
CGGGTCACATTCCTCG-1_CRC2	Epithelial cells type1	1403
GACGTGCTCCGCAGTG-1_CRC3	Epithelial cells type1	1253
TCAGGTACAGTATAAG-1_CRC1	Epithelial cells type1	2548
TGAGCCGTCCACGAAT-1_CRC2	Epithelial cells type1	823
TGGGCGTGTTCGCGAC-1_CRC2	Epithelial cells type1	963
CGAGAAGAGACCTTTG-1_CRC2	Epithelial cells type1	1013
GTCAAGTGTAACGCGA-1_CRC2	Epithelial cells type1	1055
CTCTGGTCATTACCTT-1_CRC2	Epithelial cells type1	358
CGATGTATCTCACATT-1_CRC2	Epithelial cells type1	606
TCTGGAAAGACTGTAA-1_CRC2	Epithelial cells type1	557

AACTCAGAGAGGACGG-1_CRC2	Epithelial cells type1	395
CTCGTCACATAGACTC-1_CRC2	Epithelial cells type1	361
AAGGCAGGTCCTGCTT-1_CRC2	Epithelial cells type1	480
GATTCAGGTACACCGC-1_CRC2	Epithelial cells type1	462
ATCCGAAAGCTGCAAG-1_CRC2	Epithelial cells type1	350
GTCGGGTTCGGCATCG-1_CRC2	Epithelial cells type1	270
CACATTTGTCTAACGT-1_CRC2	Epithelial cells type1	536
TCAGATGCAATCACAC-1_CRC2	Epithelial cells type1	496
ACTTACTCATGCCTAA-1_CRC2	Epithelial cells type1	619
GGTGTTAAGGGAGTAA-1_CRC2	Epithelial cells type1	318
GTCTTCGTCTCTGTCG-1_CRC2	Epithelial cells type1	369
ACTTGTTAGATGGCGT-1_CRC2	Epithelial cells type1	315
AGCGGTCCAGTGACAG-1_CRC2	Epithelial cells type1	378
TTCGGTCTCTCTTATG-1_CRC2	Epithelial cells type1	353
TTTATGCGTACGAAAT-1_CRC2	Epithelial cells type1	267
GACTGCGCATTTGCCC-1_CRC2	Epithelial cells type1	800
CATATGGCATGCCCGA-1_CRC2	Epithelial cells type1	520
GGCGACTCATGCCACG-1_CRC2	Epithelial cells type1	589
CAGAATCCACATGACT-1_CRC2	Epithelial cells type1	287
ACTGTCCTCATGCAAC-1_CRC2	Epithelial cells type1	266
CGTTCTGCATAGTAAG-1_CRC2	Epithelial cells type1	225
CTAGCCTGTGCGGTAA-1_CRC2	Epithelial cells type1	257
GGCGACTTCCCTGACT-1_CRC2	Epithelial cells type1	157
ACAGCTACAATAAGCA-1_CRC2	Epithelial cells type1	242
AGATCTGGTCCGTGAC-1_CRC2	Epithelial cells type1	107
CGACCTTTCACATAGC-1_CRC2	Epithelial cells type1	212
ACGCAGCCAATAGCGG-1_CRC2	Epithelial cells type1	203
CTCTACGAGGCCCTTG-1_CRC2	Epithelial cells type1	162
GCGCAACAGCATCATC-1_CRC2	Epithelial cells type1	630
GCTGGGTTCTCTGAGA-1_CRC2	Epithelial cells type1	300
TCTTTCCGTTTGGGCC-1_CRC2	Epithelial cells type1	553
CTCGGAGAGCGTGTCC-1_CRC2	Epithelial cells type1	439
AACCGCGTCAACACGT-1_CRC2	Epithelial cells type1	130
GTCGTAACAAGTACCT-1_CRC2	Epithelial cells type1	70
AACGTTGGTGCTGTAT-1_CRC2	Epithelial cells type1	281
TTAGGACAGGTGTTAA-1_CRC2	Epithelial cells type1	410
AACTCTTGTTGCGTTA-1_CRC2	Epithelial cells type1	308
GCAAACTCATGCCTAA-1_CRC2	Epithelial cells type1	341
TTAGGACGTCCAACTA-1_CRC2	Epithelial cells type1	292
ACACCCTTCGCACTCT-1_CRC2	Epithelial cells type1	226
ATAACGCAGGTGATAT-1_CRC2	Epithelial cells type1	171
TTGGAACAGACAGGCT-1_CRC0	Epithelial cells type1	1519
GTCGGGTTCATAGCAC-1_CRC0	Epithelial cells type1	1153
CTACATTCAGCAGTTT-1_CRC0	Epithelial cells type1	915
TCGGGACAGCTGATAA-1_CRC0	Epithelial cells type1	703
AACTGGTTCACCCTCA-1_CRC1	Epithelial cells type1	461
AAGGCAGAGACTCGGA-1_CRC2	Epithelial cells type1	3443
AACTTTCAGGTGATAT-1_CRC2	Epithelial cells type1	3909
TACTCGCTCGATGAGG-1_CRC2	Epithelial cells type1	2286
GTCGGGTTCTCTAAGG-1_CRC0	Epithelial cells type1	1385
CCCTCCTAGGTGACCA-1_CRC2	Epithelial cells type1	2047

ACGATGTGTCACACGC-1_CRC2	Epithelial cells type1	2031
GCCAAATGTTTGACTG-1_CRC2	Epithelial cells type1	1208
CCTTCCCGTAATCACC-1_CRC2	Epithelial cells type1	944
GTGAAGGAGGCAAAGA-1_CRC2	Epithelial cells type1	1421
GGATGTTCATGAACCT-1_CRC3	Epithelial cells type1	3074
GTGCAGCTCGGCGCTA-1_CRC1	Epithelial cells type1	3516
TTGGCAACAATCACAC-1_CRC1	Epithelial cells type1	2333
CCTTCCCCATCATCCC-1_CRC1	Epithelial cells type1	2551
CTACACCTCCACTGGG-1_CRC1	Epithelial cells type1	2220
GGACAAGAGTAGCGGT-1_CRC1	Epithelial cells type1	1739
GAAACTCAGCCGATTT-1_CRC1	Epithelial cells type1	729
CGCGGTAGTCCTCCAT-1_CRC2	Epithelial cells type1	1518
TTAGTTCGTTGAGTTC-1_CRC2	Epithelial cells type1	1242
CGATTGATCCTGCCAT-1_CRC1	Epithelial cells type1	1971
CGAGCCATCCCTGACT-1_CRC1	Epithelial cells type1	1363
AACTCTTCAATAAGCA-1_CRC1	Epithelial cells type1	1321
GGCGACTCAATGGTCT-1_CRC1	Epithelial cells type1	990
TCGGTAAAGCTCCTCT-1_CRC1	Epithelial cells type1	1304
TTAGTTCCACGTTGGC-1_CRC1	Epithelial cells type1	1480
CACACCTTCTTGAGGT-1_CRC1	Epithelial cells type1	2205
AACTCAGGTTAGAACA-1_CRC1	Epithelial cells type1	1291
GGGACCTTCTCTTGAT-1_CRC1	Epithelial cells type1	1981
CACACAAAGAAACCTA-1_CRC1	Epithelial cells type1	2087
TCAGGTAAGGGCTTGA-1_CRC1	Epithelial cells type1	1726
CATATGGAGCATCATC-1_CRC1	Epithelial cells type1	1415
GGCAATTAGCGGATCA-1_CRC1	Epithelial cells type1	1795
AGCGTATAGTCTCGGC-1_CRC1	Epithelial cells type1	1801
CATCGAAAGCCACCTG-1_CRC1	Epithelial cells type1	1649
CGCTTCACATTCCTGC-1_CRC1	Epithelial cells type1	1464
GCATACATCTGTTTGT-1_CRC2	Epithelial cells type1	2843
CCGTACTGTCCTCCAT-1_CRC2	Epithelial cells type1	1912
TTTATGCCAAATCCGT-1_CRC2	Epithelial cells type1	1629
ATTGGACCAAATCCGT-1_CRC3	Epithelial cells type1	2031
CGTCACTCAAAGTGCG-1_CRC1	Epithelial cells type1	1949
CACTCCACAAGCGAGT-1_CRC1	Epithelial cells type1	1730
TTCTTAGGTCTCTTTA-1_CRC1	Epithelial cells type1	1548
TGCTACCCATGATCCA-1_CRC3	Epithelial cells type1	1605
GTGGGTCCAGTCAGAG-1_CRC2	Epithelial cells type1	1222
CCGTTCAAGCACGCCT-1_CRC3	Epithelial cells type1	1890
AAGGCAGTCCTTTACA-1_CRC0	Epithelial cells type2	2791
AAGGTTCCACGAAGCA-1_CRC0	Epithelial cells type2	2189
ACATCAGCAAGGCTCC-1_CRC0	Epithelial cells type2	1750
GCAAACTCAATCGGTT-1_CRC0	Epithelial cells type2	1765
CGTAGCGCAATGGACG-1_CRC0	Epithelial cells type2	1313
CTAATGGTCGGTCTAA-1_CRC0	Epithelial cells type2	1894
CTCGAGGGTCACACGC-1_CRC0	Epithelial cells type2	1642
TTCTCAATCGTATCAG-1_CRC0	Epithelial cells type2	2533
GATCGTAAGCAGCCTC-1_CRC0	Epithelial cells type2	2251
GGATGTTGTTTACTCT-1_CRC0	Epithelial cells type2	2497
CGGAGCTCACGCCAGT-1_CRC0	Epithelial cells type2	2506
ACATACGTCACCCGAG-1_CRC0	Epithelial cells type2	2025

TACGGTAAGACCCACC-1_CRC0	Epithelial cells type2	1468
CGCGTTTAGTTTCCTT-1_CRC0	Epithelial cells type2	2048
CGGTTAAGTTACGGAG-1_CRC0	Epithelial cells type2	1907
GATCGATAGCGCCTCA-1_CRC0	Epithelial cells type2	1870
ATTATCCTCAGTGCAT-1_CRC0	Epithelial cells type2	2117
ATTGGACGTAGCGCTC-1_CRC0	Epithelial cells type2	1868
AAGACCTTCCAAACTG-1_CRC0	Epithelial cells type2	2635
CATATGGCAAGACGTG-1_CRC0	Epithelial cells type2	2094
CCAGCGACAGGCGATA-1_CRC0	Epithelial cells type2	2391
CTAAGACGTACGACCC-1_CRC0	Epithelial cells type2	2094
GCACTCTAGAATGTTG-1_CRC0	Epithelial cells type2	1671
AGGGATGCAGTTCCCT-1_CRC0	Epithelial cells type2	2478
ATAAGAGGTAGGACAC-1_CRC0	Epithelial cells type2	1593
CCCAATCGTCTAGAGG-1_CRC0	Epithelial cells type2	1826
ATCTGCCAGTGTACGG-1_CRC0	Epithelial cells type2	1713
CATCAGAAGAGTTGGC-1_CRC0	Epithelial cells type2	2251
CTCTACGTCAGTTCGA-1_CRC0	Epithelial cells type2	2901
GCATGCGTCATTTGGG-1_CRC0	Epithelial cells type2	2217
TACGGATTCATTTGGG-1_CRC0	Epithelial cells type2	2196
GGAGCAATCCTCCTAG-1_CRC0	Epithelial cells type2	2480
GGTGAAGTCCAGAGGA-1_CRC0	Epithelial cells type2	2322
CAACCTCGTTAAGTAG-1_CRC0	Epithelial cells type2	1900
CATCCACAGGGCTTGA-1_CRC0	Epithelial cells type2	2022
GCGACCAGTTACCAGT-1_CRC0	Epithelial cells type2	1832
CTCTGGTGTCAATACC-1_CRC0	Epithelial cells type2	1870
CCACCTATCACTGGGC-1_CRC0	Epithelial cells type2	1717
ACACCCTCAGCTCCGA-1_CRC0	Epithelial cells type2	1718
CCGTGGAGTTACGACT-1_CRC0	Epithelial cells type2	1708
GGACAAGGTAGCGTAG-1_CRC0	Epithelial cells type2	1850
GGATGTTAGAGCTGCA-1_CRC0	Epithelial cells type2	1666
AACTTTCCATAAGACA-1_CRC0	Epithelial cells type2	1643
AGCCTAAAGTCTTGCA-1_CRC0	Epithelial cells type2	1748
TTGCCGTTCGCAAACT-1_CRC0	Epithelial cells type2	1319
GACCTGGAGATCGGGT-1_CRC0	Epithelial cells type2	1209
CGTCAGGTCTCAAACG-1_CRC0	Epithelial cells type2	1734
CCTAAAGGTGGCTCCA-1_CRC0	Epithelial cells type2	1530
GGTGTTACAAGCGCTC-1_CRC0	Epithelial cells type2	1523
ACGGGTCGTAGGGACT-1_CRC0	Epithelial cells type2	1663
TGACGGCAGAGGTTAT-1_CRC0	Epithelial cells type2	1677
GCTGCAGTCACATGCA-1_CRC0	Epithelial cells type2	2271
CGGGTCAGTCTTGTCC-1_CRC0	Epithelial cells type2	1941
AAGCCGCGTCGAGTTT-1_CRC0	Epithelial cells type2	1571
GTCCTCACATTGGTAC-1_CRC0	Epithelial cells type2	2249
ATGCGATCACAAGACG-1_CRC0	Epithelial cells type2	2059
ACTGAGTTCTGCTGCT-1_CRC0	Epithelial cells type2	2173
GCGCAACCAGGGAGAG-1_CRC0	Epithelial cells type2	1366
GTTCGGGGTATAAACG-1_CRC0	Epithelial cells type2	1475
TTGAACGCATCCAACA-1_CRC0	Epithelial cells type2	1528
AAATGCCCATGAAGTA-1_CRC0	Epithelial cells type2	2076
CATATTCTCGTAGGAG-1_CRC0	Epithelial cells type2	1672
CTGTGCTAGTCGTTTG-1_CRC0	Epithelial cells type2	1276

ACAGCCGTCTCGCATC-1_CRC0	Epithelial cells type2	1926
GTGCAGCAGTTTGCGT-1_CRC0	Epithelial cells type2	1684
TACCTATAGCCATCGC-1_CRC0	Epithelial cells type2	1405
CAGCTAAAGCCACGCT-1_CRC0	Epithelial cells type2	1718
GTACGTACAGTCGATT-1_CRC0	Epithelial cells type2	1764
ATCACGAAGAGACGAA-1_CRC0	Epithelial cells type2	1733
GTATTCTTCGTAGATC-1_CRC0	Epithelial cells type2	2182
CAGTAACGTAAGCACG-1_CRC0	Epithelial cells type2	2240
TTTACTGTCTTTCCTC-1_CRC0	Epithelial cells type2	1957
CATATGGAGTATGACA-1_CRC0	Epithelial cells type2	1638
CATTATCGTCTTGATG-1_CRC0	Epithelial cells type2	1753
GTTCATTGTGTTCGAT-1_CRC0	Epithelial cells type2	1665
ATAACGCAGATGCCTT-1_CRC0	Epithelial cells type2	2010
ACTGATGAGATAGGAG-1_CRC0	Epithelial cells type2	2068
GGCTCGAGTAAGAGGA-1_CRC0	Epithelial cells type2	1622
TGAAAGAGTAATAGCA-1_CRC0	Epithelial cells type2	2101
CCTAGCTTCGATAGAA-1_CRC0	Epithelial cells type2	1451
CTGCGGACACCCATTC-1_CRC0	Epithelial cells type2	964
ACACCAATCAGCGATT-1_CRC0	Epithelial cells type2	1975
GCATGCGAGTGCGATG-1_CRC0	Epithelial cells type2	1499
CTGTGCTTCCGAACGC-1_CRC0	Epithelial cells type2	2072
GGGATGATCTTCAACT-1_CRC0	Epithelial cells type2	1851
ACTTTCACAACGATCT-1_CRC0	Epithelial cells type2	1560
ATAACGCAGCAATATG-1_CRC0	Epithelial cells type2	1666
TACACGAGTTGCCTCT-1_CRC0	Epithelial cells type2	1676
CAGTCCTCATAGACTC-1_CRC0	Epithelial cells type2	914
GCGGGTTTCTCGTATT-1_CRC0	Epithelial cells type2	1339
ATAAGAGCAATCTGCA-1_CRC0	Epithelial cells type2	1573
CGTGAGCGTCTCGTTC-1_CRC0	Epithelial cells type2	1807
GACTACATCAACGAAA-1_CRC0	Epithelial cells type2	1765
CACTCCAGTTAAGAAC-1_CRC0	Epithelial cells type2	1349
ACTGATGTCAGCTCGG-1_CRC0	Epithelial cells type2	1070
GCAAACTTCAAGATCC-1_CRC0	Epithelial cells type2	598
GGACATTTCCATGAAC-1_CRC0	Epithelial cells type2	913
CGAGAAGTCGCAAGCC-1_CRC0	Epithelial cells type2	1148
TATGCCCTCTTGAGGT-1_CRC0	Epithelial cells type2	969
GAGGTGAAGTCTCGGC-1_CRC0	Epithelial cells type2	1108
TAGACCACACAAGTAA-1_CRC0	Epithelial cells type2	1510
GATCAGTCACGCTTTC-1_CRC0	Epithelial cells type2	1309
GTCTTCGCACGGCCAT-1_CRC0	Epithelial cells type2	1457
CGGCTAGTCCTCATTA-1_CRC0	Epithelial cells type2	1640
TCGCGAGAGGCAAAGA-1_CRC0	Epithelial cells type2	1347
CACACAATCTTTAGTC-1_CRC0	Epithelial cells type2	866
CTACCCACATGTCGAT-1_CRC0	Epithelial cells type2	964
CTCGGGAAGTCGTACT-1_CRC0	Epithelial cells type2	1281
AGTGGGACATGGTCTA-1_CRC0	Epithelial cells type2	1118
CGGACACAGTAAGTAC-1_CRC0	Epithelial cells type2	1135
TAAGAGAAGATGTTAG-1_CRC0	Epithelial cells type2	1105
CGATGTACATATACCG-1_CRC0	Epithelial cells type2	1642
AAAGATGTCCACGTGG-1_CRC0	Epithelial cells type2	1549
GATCTAGCAGACGCCT-1_CRC0	Epithelial cells type2	1862

ATCATCTAGGTGATTA-1_CRC0	Epithelial cells type2	1666
AACGTTGAGGAGTCTG-1_CRC0	Epithelial cells type2	1274
CTGAAGTTCCCTTGTG-1_CRC0	Epithelial cells type2	1341
CAAGGCCCAGCTTAAC-1_CRC0	Epithelial cells type2	1554
TAAGCGTTCTTGGGTA-1_CRC0	Epithelial cells type2	1347
GTGCTTCTCAACACCA-1_CRC0	Epithelial cells type2	1705
TACCTATCACTTCTGC-1_CRC0	Epithelial cells type2	1819
CTCTAATTCATTTGGG-1_CRC0	Epithelial cells type2	2050
GCAGTTATCTTTACAC-1_CRC0	Epithelial cells type2	1408
CTGTTTAAGTGTACCT-1_CRC0	Epithelial cells type2	2304
GGACATTGTGGTCCGT-1_CRC0	Epithelial cells type2	1891
AAACCTGGTTGGTAAA-1_CRC0	Epithelial cells type2	2194
GTCTTCGAGGACGAAA-1_CRC0	Epithelial cells type2	2622
CGGACGTGTACAGTGG-1_CRC0	Epithelial cells type2	1706
GATGAGGGTTTGACTG-1_CRC0	Epithelial cells type2	1590
CATCCACAGGAGTTGC-1_CRC0	Epithelial cells type2	1436
AACTCTTGTCTAGCGC-1_CRC0	Epithelial cells type2	1457
CGTCTACCAGTCGTGC-1_CRC0	Epithelial cells type2	1723
CGATCGGAGCCACCTG-1_CRC0	Epithelial cells type2	2031
ATGAGGGTCGTCTGCT-1_CRC0	Epithelial cells type2	1459
TGCCCATCACGGTGTC-1_CRC0	Epithelial cells type2	1402
CAAGGCCAGATGCCTT-1_CRC0	Epithelial cells type2	1760
GCTGCGACATTAGCCA-1_CRC0	Epithelial cells type2	1914
AACTTTCAGTGGGTTG-1_CRC0	Epithelial cells type2	1827
ACTTACTAGAGACTAT-1_CRC0	Epithelial cells type2	1373
TTAGGCACATTTCAGG-1_CRC0	Epithelial cells type2	1689
GTATTCTTCAGTTAGC-1_CRC0	Epithelial cells type2	2020
TGCGTGGCACCACCAG-1_CRC0	Epithelial cells type2	1120
CAGCAGCAGATACACA-1_CRC0	Epithelial cells type2	1318
CCTCTGACATGTCTCC-1_CRC0	Epithelial cells type2	1209
TTCTACAAGTTTGCGT-1_CRC0	Epithelial cells type2	1645
AGCATACAGAGTAAGG-1_CRC0	Epithelial cells type2	1786
GATCTAGAGCGATATA-1_CRC0	Epithelial cells type2	1793
AGTGTCAGTCAACATC-1_CRC0	Epithelial cells type2	1493
TAAACCGCATTGGTAC-1_CRC0	Epithelial cells type2	1370
TACTCATGTTGGACCC-1_CRC0	Epithelial cells type2	1145
AACTTTCCAGGCTGAA-1_CRC0	Epithelial cells type2	1752
GGGATGAAGCCGGTAA-1_CRC0	Epithelial cells type2	1831
CACATTTAGCTGTCTA-1_CRC0	Epithelial cells type2	1074
CACACTCGTAACGCGA-1_CRC0	Epithelial cells type2	1632
TTTGTCACAGGGTATG-1_CRC0	Epithelial cells type2	1691
TGGGAAGCAGGCGATA-1_CRC0	Epithelial cells type2	2236
CACATAGCAAACTGCT-1_CRC0	Epithelial cells type2	1928
CATTCGCTCTCAAGTG-1_CRC0	Epithelial cells type2	1641
AGCTTGAAGACGACGT-1_CRC0	Epithelial cells type2	1886
CATATTCTCGGAGGTA-1_CRC0	Epithelial cells type2	1191
TCACGAAAGCCGGTAA-1_CRC0	Epithelial cells type2	2188
CGCGTTTCAGGGTACA-1_CRC0	Epithelial cells type2	1764
CGGACTGAGGCTCAGA-1_CRC0	Epithelial cells type2	2144
ACATCAGGTCCCTACT-1_CRC0	Epithelial cells type2	2407
CATCAGAGTGTGACGA-1_CRC0	Epithelial cells type2	1798

CGTGTCTCACAACGCC-1_CRC0	Epithelial cells type2	1967
CTACACCGTCCAGTAT-1_CRC0	Epithelial cells type2	1766
GGGTCTGAGCCGTCGT-1_CRC0	Epithelial cells type2	1916
CCACGGATCGTGACAT-1_CRC0	Epithelial cells type2	2076
GGGTTGCCACAAGACG-1_CRC0	Epithelial cells type2	2108
TTATGCTTCAGCATGT-1_CRC0	Epithelial cells type2	1792
ACATCAGAGTGTTGAA-1_CRC0	Epithelial cells type2	1572
CTAGCCTCAAGAAAGG-1_CRC0	Epithelial cells type2	1573
TCAGGTATCTTGAGGT-1_CRC0	Epithelial cells type2	1907
CCTTTCTCACGAAGCA-1_CRC0	Epithelial cells type2	1495
TGGCGCATCATCATTC-1_CRC0	Epithelial cells type2	1772
GGAAAGCAGGAGTAGA-1_CRC0	Epithelial cells type2	2164
GTTCTCGGTCAAAGCG-1_CRC0	Epithelial cells type2	2035
CAGCTAAGTTACGTCA-1_CRC0	Epithelial cells type2	1625
AACTCTTCAGGATCGA-1_CRC0	Epithelial cells type2	1287
CACACCTGTTGATTCG-1_CRC0	Epithelial cells type2	1181
ACTGAACGTGCAACTT-1_CRC0	Epithelial cells type2	1936
ACCAGTAGTAGTACCT-1_CRC0	Epithelial cells type2	1639
GTCGTAATCCGGGTGT-1_CRC0	Epithelial cells type2	1256
CCAATCCTCCACGAAT-1_CRC0	Epithelial cells type2	1318
GTTCGGGAGATATGGT-1_CRC0	Epithelial cells type2	1004
GCACATACACGTCAGC-1_CRC0	Epithelial cells type2	1998
AGATTGCGTGGTTTCA-1_CRC0	Epithelial cells type2	929
CTCGTCATCACGCATA-1_CRC0	Epithelial cells type2	1722
ACGAGGAAGCACGCCT-1_CRC0	Epithelial cells type2	1339
GCAGTTAGTACAAGTA-1_CRC0	Epithelial cells type2	1253
TGCCCTACATTACCTT-1_CRC0	Epithelial cells type2	1460
GTGTGCGTCTAACCGA-1_CRC0	Epithelial cells type2	1124
ATTGGACGTAAACACA-1_CRC0	Epithelial cells type2	1023
GAAGCAGCAAGCGAGT-1_CRC0	Epithelial cells type2	1492
CATCGGGTCCACGACG-1_CRC0	Epithelial cells type2	1461
ACGGGCTGTATGCTTG-1_CRC0	Epithelial cells type2	1291
CACACCTTCCTAAGTG-1_CRC0	Epithelial cells type2	1517
CGTGTCTGTTGTTTGG-1_CRC0	Epithelial cells type2	1334
CACAGGCCACAAGACG-1_CRC0	Epithelial cells type2	976
GTAACTGAGGGAAACA-1_CRC0	Epithelial cells type2	1201
ATGCGATAGTATCTCG-1_CRC0	Epithelial cells type2	1329
TACAGTGGTCGAACAG-1_CRC0	Epithelial cells type2	1152
CCTCTGAGTAGTGAAT-1_CRC0	Epithelial cells type2	952
CCGTTCATCATCGATG-1_CRC0	Epithelial cells type2	1282
TCACGAAAGCAGCCTC-1_CRC0	Epithelial cells type2	1205
ACTGAGTAGGCCCTCA-1_CRC0	Epithelial cells type2	1115
ACTTGTTAGACCCACC-1_CRC0	Epithelial cells type2	1194
CGCTTCATCCACGTGG-1_CRC0	Epithelial cells type2	1195
GATGCTATCCAAACAC-1_CRC0	Epithelial cells type2	1903
CTGAAACTCGGCGCTA-1_CRC0	Epithelial cells type2	1092
GTAACTGCACCGCTAG-1_CRC0	Epithelial cells type2	1272
GTGCAGCTCCACGTTC-1_CRC0	Epithelial cells type2	1394
ACGCAGCCAGTTCCCT-1_CRC0	Epithelial cells type2	1730
CAGCTAAAGATCCCGC-1_CRC0	Epithelial cells type2	1588
GCAATCAGTAGGGACT-1_CRC0	Epithelial cells type2	1899

TGACTAGGTAATCACC-1_CRC0	Epithelial cells type2	1890
CCTTCCCAGACTGTAA-1_CRC0	Epithelial cells type2	2188
CAGCAGCCATGCAACT-1_CRC0	Epithelial cells type2	1710
TCCACACCACATGTGT-1_CRC0	Epithelial cells type2	1135
GCATGTAAGTGGAGAA-1_CRC0	Epithelial cells type2	2104
ACCCACTCAGCAGTTT-1_CRC0	Epithelial cells type2	2238
CCGTGGATCACCATAG-1_CRC0	Epithelial cells type2	1627
TGAGAGGGTTCGGGCT-1_CRC0	Epithelial cells type2	2272
AGATTGCTCGGATGTT-1_CRC0	Epithelial cells type2	1900
TGGACGCGTGGCGAAT-1_CRC0	Epithelial cells type2	1740
GAAATGAGTGAGCGAT-1_CRC0	Epithelial cells type2	2042
GGCTGGTGTCTCCACT-1_CRC0	Epithelial cells type2	2428
GGTATTGCAGCTTAAC-1_CRC0	Epithelial cells type2	1836
CGCGTTTAGACGCACA-1_CRC0	Epithelial cells type2	2153
CTCTAATCAATAGCGG-1_CRC0	Epithelial cells type2	2246
AGTCTTTCACCTGGTG-1_CRC0	Epithelial cells type2	2243
CCTAGCTAGCAACGGT-1_CRC0	Epithelial cells type2	2052
GGGAATGTCAGGCCCA-1_CRC0	Epithelial cells type2	2193
ATTTCTGCATGCCCGA-1_CRC0	Epithelial cells type2	1850
GTCTCGTTCGAGAACG-1_CRC0	Epithelial cells type2	2008
TCACGAAAGTGGGATC-1_CRC0	Epithelial cells type2	2474
TGGCCAGTCTAACCGA-1_CRC0	Epithelial cells type2	2399
TCTGGAACACGGCGTT-1_CRC0	Epithelial cells type2	2329
GAATAAGTCTTGTCAT-1_CRC0	Epithelial cells type2	1836
GGGAGATTCTTCCTTC-1_CRC0	Epithelial cells type2	1965
TGCTGCTCAACACCCG-1_CRC0	Epithelial cells type2	2163
GTGTTAGAGCCGATTT-1_CRC0	Epithelial cells type2	2271
AACTGGTTCACATGCA-1_CRC0	Epithelial cells type2	1356
CGCTGGAAGTCCCACG-1_CRC0	Epithelial cells type2	1415
GGGACCTAGAATCTCC-1_CRC0	Epithelial cells type2	2259
TCTTCGGTCATCACCC-1_CRC0	Epithelial cells type2	1759
CGTAGGCCACCCAGTG-1_CRC0	Epithelial cells type2	1567
GTGCATACATTATCTC-1_CRC0	Epithelial cells type2	1286
TCTCATATCAGAGCTT-1_CRC0	Epithelial cells type2	1231
GTCTTCGCAATCGAAA-1_CRC0	Epithelial cells type2	1762
CTGCTGTCATATGCTG-1_CRC0	Epithelial cells type2	1550
TTTACTGAGACGCTTT-1_CRC0	Epithelial cells type2	1761
ACGGGTCCAGACGCTC-1_CRC0	Epithelial cells type2	1763
TCAACGAGTAAGAGGA-1_CRC0	Epithelial cells type2	1885
CCCAGTTGTTCGAATC-1_CRC0	Epithelial cells type2	1934
AGCCTAATCTCGATGA-1_CRC0	Epithelial cells type2	1647
CCTTCCCTCGAACTGT-1_CRC0	Epithelial cells type2	1847
AGCGTCGGTTATCACG-1_CRC0	Epithelial cells type2	1638
TACTTGTCACCAGCAC-1_CRC1	Epithelial cells type2	2339
AACTCCCGTTAAGGGC-1_CRC0	Epithelial cells type2	1679
TCACGAAGTAACGCGA-1_CRC0	Epithelial cells type2	1917
AATCCAGGTTCAGCGC-1_CRC0	Epithelial cells type2	2099
CACAAACTCGCGTAGC-1_CRC0	Epithelial cells type2	2995
GGGTCTGGTGAGTGAC-1_CRC0	Epithelial cells type2	1465
TGCCAAACACGCATCG-1_CRC0	Epithelial cells type2	1146
TGCGGGTCAAGGTTCT-1_CRC0	Epithelial cells type2	1641

TCGAGGCCATGTTCCC-1_CRC0	Epithelial cells type2	1291
AGCGGTCAGCCGTCGT-1_CRC0	Epithelial cells type2	1211
CTCATTACAATGAAAC-1_CRC0	Epithelial cells type2	1315
CAACTAGGTCATCCCT-1_CRC0	Epithelial cells type2	1800
ACTGAACCAGGACCCT-1_CRC0	Epithelial cells type2	2042
CAGTCCTTCAACGAAA-1_CRC0	Epithelial cells type2	2367
ACTTACTGTTTAAGCC-1_CRC0	Epithelial cells type2	1499
CTTTGCGGTAATTGGA-1_CRC0	Epithelial cells type2	2013
TTTGCGCAGGTAGCTG-1_CRC0	Epithelial cells type2	1926
GCCTCTAGTTAAGGGC-1_CRC0	Epithelial cells type2	1317
CGCCAAGTCTTGTTTG-1_CRC0	Epithelial cells type2	1564
TCATTACTCCATGCTC-1_CRC0	Epithelial cells type2	1585
CATCCACTCGCCTGTT-1_CRC0	Epithelial cells type2	1920
ACGAGCCGTAGATTAG-1_CRC0	Epithelial cells type2	1530
TCTGGAACAGGAACGT-1_CRC0	Epithelial cells type2	1810
ATTTCTGGTGTTGAGG-1_CRC0	Epithelial cells type2	1185
CTGTGCTAGACCGGAT-1_CRC0	Epithelial cells type2	1177
AAGCCGCTCTTCCTTC-1_CRC0	Epithelial cells type2	2336
TGGTTAGGTCATCCCT-1_CRC0	Epithelial cells type2	2015
TGTATTCAGACGCTTT-1_CRC0	Epithelial cells type2	1811
CGTTGGGTCTACTTAC-1_CRC0	Epithelial cells type2	1253
CGACTTCAGCGCTCCA-1_CRC0	Epithelial cells type2	1275
TCTTCGGTCTAACTTC-1_CRC0	Epithelial cells type2	1799
GATTCAGAGTGTGGCA-1_CRC0	Epithelial cells type2	1458
CAGCTAAGTAAGTGGC-1_CRC0	Epithelial cells type2	1161
CCCTCCTCATGCTGGC-1_CRC0	Epithelial cells type2	944
CTACCCAGTCCATCCT-1_CRC0	Epithelial cells type2	1679
AAGACCTAGCAGATCG-1_CRC0	Epithelial cells type2	1924
CCGTTCAGTGATGCCC-1_CRC0	Epithelial cells type2	1642
TAAGCGTTCTTGACGA-1_CRC0	Epithelial cells type2	2398
CTGCGGACATCGGAAG-1_CRC0	Epithelial cells type2	1127
ATCCACCCACGGTAGA-1_CRC0	Epithelial cells type2	1406
CCCTCCTCATACGCCG-1_CRC0	Epithelial cells type2	1224
GTCATTTAGTGGTAAT-1_CRC0	Epithelial cells type2	1117
TGCACCTCAAAGCAAT-1_CRC0	Epithelial cells type2	1256
TTGAACGTCGAGAGCA-1_CRC0	Epithelial cells type2	2011
ACTGAACCACATGGGA-1_CRC0	Epithelial cells type2	1019
CCTTCGACACACATGT-1_CRC0	Epithelial cells type2	1464
TGAGCATAGAATAGGG-1_CRC0	Epithelial cells type2	1490
TGTCCCAAGGCATTGG-1_CRC0	Epithelial cells type2	2068
TTTGCGCAGTATGACA-1_CRC0	Epithelial cells type2	3019
CCGTGGAGTTACGTCA-1_CRC0	Epithelial cells type2	2230
TACTCGCCAGTACACT-1_CRC0	Epithelial cells type2	2351
CGAACATGTACCAGTT-1_CRC0	Epithelial cells type2	2129
AGAGCTTGTTCAGGCC-1_CRC0	Epithelial cells type2	1905
ATCCGAAGTTTGACTG-1_CRC0	Epithelial cells type2	2024
ATCATGGAGACAATAC-1_CRC0	Epithelial cells type2	2454
GGTGCGTTCAAGCCTA-1_CRC0	Epithelial cells type2	1386
TACTCGCGTCTCATCC-1_CRC0	Epithelial cells type2	2132
AACTTTCTCTGATACG-1_CRC0	Epithelial cells type2	2270
CCACTACAGTCGATAA-1_CRC0	Epithelial cells type2	2114

CTCGAGGAGGCTAGCA-1_CRC0	Epithelial cells type2	2409
TCAGCAATCTTGTCAT-1_CRC0	Epithelial cells type2	2644
TTTACTGAGTGGGCTA-1_CRC0	Epithelial cells type2	1636
GTGCAGCCAGAAGCAC-1_CRC0	Epithelial cells type2	1914
TGTTCCGTCGCGCCAA-1_CRC0	Epithelial cells type2	1833
CGGTTAATCGAATCCA-1_CRC1	Epithelial cells type2	4091
TACACGATCCTCATTA-1_CRC1	Epithelial cells type2	969
GGGCACTCAAGGACTG-1_CRC1	Epithelial cells type2	765
GACGGCTCAGCATGAG-1_CRC1	Epithelial cells type2	1217
TGCGCAGCACAAGACG-1_CRC1	Epithelial cells type2	967
TGTATTCCACCCATGG-1_CRC1	Epithelial cells type2	922
AAGGAGCGTGATGCCC-1_CRC1	Epithelial cells type2	1123
TTTGCGCTCGCTGATA-1_CRC1	Epithelial cells type2	1036
CCCAGTTGTTCCACAA-1_CRC1	Epithelial cells type2	1445
AACGTTGTCGGATGTT-1_CRC1	Epithelial cells type2	795
TACTTACAGACAAGCC-1_CRC1	Epithelial cells type2	760
TATCAGGTCAAGGTAA-1_CRC1	Epithelial cells type2	1239
CGTAGCGGTAGAGTGC-1_CRC1	Epithelial cells type2	1394
CTCATTACACAGAGGT-1_CRC1	Epithelial cells type2	984
TCTGAGATCCCTCTTT-1_CRC1	Epithelial cells type2	1093
GCGGGTTTCTACCAGA-1_CRC1	Epithelial cells type2	2089
GAAATGACATGTCGAT-1_CRC1	Epithelial cells type2	1390
TCATTTGGTAGAGCTG-1_CRC1	Epithelial cells type2	1686
CCGGGATGTGAAATCA-1_CRC1	Epithelial cells type2	1455
GAAACTCTCCTTGACC-1_CRC1	Epithelial cells type2	1411
ACGGGCTGTACCCAAT-1_CRC1	Epithelial cells type2	1462
CGCGTTTCAGGAACGT-1_CRC1	Epithelial cells type2	1280
GCATGTACAGTAAGCG-1_CRC1	Epithelial cells type2	1073
CTACCCACATTACGAC-1_CRC1	Epithelial cells type2	1454
TTCCCAGCAAGTTAAG-1_CRC1	Epithelial cells type2	1617
ATGGGAGTCGTCGTTC-1_CRC1	Epithelial cells type2	1089
AGTGGGATCTTACCTA-1_CRC1	Epithelial cells type2	1888
CTGATAGGTGATGTCT-1_CRC1	Epithelial cells type2	1669
ACTGTCCCACCACGTG-1_CRC1	Epithelial cells type2	2135
CTGTTTAAGGCATGTG-1_CRC1	Epithelial cells type2	1423
AGATCTGAGATATGGT-1_CRC1	Epithelial cells type2	1043
ATCTGCCAGGAACTGC-1_CRC1	Epithelial cells type2	1450
GACGGCTCATCGATGT-1_CRC1	Epithelial cells type2	1216
CCTTACGGTTAAAGTG-1_CRC1	Epithelial cells type2	1469
TTAGGCAGTAGGACAC-1_CRC1	Epithelial cells type2	1639
TGGTTCCTCCCTCAGT-1_CRC1	Epithelial cells type2	1468
TACGGGCCAAGCGCTC-1_CRC1	Epithelial cells type2	1133
TCGTACCTCAAGGCTT-1_CRC1	Epithelial cells type2	1511
AGGTCCGAGGACGAAA-1_CRC1	Epithelial cells type2	1334
CAGAGAGAGCGTGAGT-1_CRC1	Epithelial cells type2	1196
CAGTCCTAGTAGTGCG-1_CRC1	Epithelial cells type2	1373
CCGTTCAAGTTACCCA-1_CRC1	Epithelial cells type2	924
CGTTGGGCAATCACAC-1_CRC1	Epithelial cells type2	1719
ACCAGTACATCAGTAC-1_CRC1	Epithelial cells type2	1328
GCATGATCAAGTAGTA-1_CRC1	Epithelial cells type2	1351
GCGAGAAAGCCAGTAG-1_CRC1	Epithelial cells type2	1778

ACCCACTTCCTTGCCA-1_CRC1	Epithelial cells type2	1465
GTATTCTCATTCGACA-1_CRC1	Epithelial cells type2	1616
TCGCGAGGTATGCTTG-1_CRC1	Epithelial cells type2	1039
CCTTCGACAGGAATGC-1_CRC1	Epithelial cells type2	1164
CTACGTCTCGAGGTAG-1_CRC1	Epithelial cells type2	1394
TCGAGGCGTCGGCACT-1_CRC1	Epithelial cells type2	1260
TGAAAGATCTGTGCAA-1_CRC1	Epithelial cells type2	1862
GACCAATCAAAGGAAG-1_CRC1	Epithelial cells type2	1242
TACGGTACAGTCTTCC-1_CRC1	Epithelial cells type2	1484
AGGTCCGCAGCTGTAT-1_CRC1	Epithelial cells type2	1839
TGCTACCGTAATAGCA-1_CRC1	Epithelial cells type2	1891
CTTAACTGTTCCACAA-1_CRC1	Epithelial cells type2	1406
TAAGCGTCAGCGAACA-1_CRC1	Epithelial cells type2	1313
GATTCAGCACATTCGA-1_CRC1	Epithelial cells type2	1747
GAGTCCGCATCCTAGA-1_CRC1	Epithelial cells type2	1558
TCATTTGGTCTACCTC-1_CRC1	Epithelial cells type2	1647
AACTCAGCACGCATCG-1_CRC1	Epithelial cells type2	1339
CAAGATCGTCCGTTAA-1_CRC1	Epithelial cells type2	1150
AACTGGTTCAACACAC-1_CRC1	Epithelial cells type2	1633
GATCTAGCACGCGAAA-1_CRC1	Epithelial cells type2	1686
CACAGGCGTCGCGGTT-1_CRC1	Epithelial cells type2	1586
TCACAAGGTGCCTGTG-1_CRC1	Epithelial cells type2	1920
TGGCCAGCAAGAGTCG-1_CRC1	Epithelial cells type2	1706
CCACTACTCCACGTGG-1_CRC1	Epithelial cells type2	1421
TGTTCCGTCATCGGAT-1_CRC1	Epithelial cells type2	1949
ACACCGGAGGTAGCTG-1_CRC1	Epithelial cells type2	1484
CAGGTGCCATCACGTA-1_CRC1	Epithelial cells type2	1526
CTGTTTAGTCGGCATC-1_CRC1	Epithelial cells type2	1976
GTAACTGGTTGACGTT-1_CRC1	Epithelial cells type2	1802
CACAGGCGTTACGGAG-1_CRC1	Epithelial cells type2	1723
CTACCCAGTTCGCTAA-1_CRC1	Epithelial cells type2	2228
TTCTTAGAGACGCACA-1_CRC1	Epithelial cells type2	2325
TCAGATGTCGGATGGA-1_CRC1	Epithelial cells type2	1610
CGTCTACGTGCGAAAC-1_CRC1	Epithelial cells type2	1981
GAATAAGAGTGAACAT-1_CRC1	Epithelial cells type2	1166
GTGCTTCGTTTGCATG-1_CRC1	Epithelial cells type2	1302
CATGACACAGACACTT-1_CRC1	Epithelial cells type2	2039
TAAACCGCACACATGT-1_CRC1	Epithelial cells type2	2278
CCACTACTCCACGAAT-1_CRC1	Epithelial cells type2	1912
CGCGTTTTCCTTTCGG-1_CRC1	Epithelial cells type2	1630
ACACCGGTCTTCAACT-1_CRC1	Epithelial cells type2	1182
AGCCTAAAGCTCAACT-1_CRC1	Epithelial cells type2	1299
ACTGTCCCAGTGAGTG-1_CRC1	Epithelial cells type2	1552
GACTGCGAGCACCGTC-1_CRC1	Epithelial cells type2	1969
CAAGAAACAAAGTCAA-1_CRC1	Epithelial cells type2	1867
CTGAAACTCCCGACTT-1_CRC1	Epithelial cells type2	1906
CGTTCTGTCAGCCTAA-1_CRC1	Epithelial cells type2	1459
CACAAACGTCAACTGT-1_CRC1	Epithelial cells type2	1063
CTCGAGGCAGGTTTCA-1_CRC1	Epithelial cells type2	1072
GGCTCGACAGCGTCCA-1_CRC1	Epithelial cells type2	834
GATCAGTTCTATGTGG-1_CRC1	Epithelial cells type2	1415

TGCACCTAGCTGAAAT-1_CRC1	Epithelial cells type2	1306
GTCAAGTGTATGAAAC-1_CRC1	Epithelial cells type2	1198
TGGTTCCGTGCAGACA-1_CRC1	Epithelial cells type2	1520
GATGAGGCACTGAAGG-1_CRC1	Epithelial cells type2	1673
ACGCCGACAGGTTTCA-1_CRC1	Epithelial cells type2	1364
GAGTCCGGTAGGCTGA-1_CRC1	Epithelial cells type2	1202
CACAGTAAGTTTCCTT-1_CRC1	Epithelial cells type2	2421
AGCTCTCAGTGTGAAT-1_CRC1	Epithelial cells type2	1881
CTCACACAGCCATCGC-1_CRC1	Epithelial cells type2	1497
TCCACACCAGATGAGC-1_CRC1	Epithelial cells type2	1529
CACACTCAGTTTAGGA-1_CRC1	Epithelial cells type2	1693
ACATCAGGTTCTCATT-1_CRC1	Epithelial cells type2	1367
CACATTTTCCCTGACT-1_CRC1	Epithelial cells type2	1690
AACCATGCAGACAAAT-1_CRC1	Epithelial cells type2	1250
CTCCTAGGTTCTGAAC-1_CRC1	Epithelial cells type2	1325
AGCAGCCTCTCTGTCG-1_CRC1	Epithelial cells type2	1571
CTGAAGTTCGTAGGAG-1_CRC1	Epithelial cells type2	1125
GAACATCGTCATCCCT-1_CRC1	Epithelial cells type2	1773
TGGCTGGAGGACACCA-1_CRC1	Epithelial cells type2	924
TACACGATCTGGCGTG-1_CRC1	Epithelial cells type2	1241
CCTTTCTAGGCGTACA-1_CRC1	Epithelial cells type2	1558
CACACAAAGGTAAACT-1_CRC1	Epithelial cells type2	1241
AGCTTGATCCATGAAC-1_CRC1	Epithelial cells type2	1441
TGAAAGAAGATAGTCA-1_CRC1	Epithelial cells type2	1258
CGGACACAGACTACAA-1_CRC1	Epithelial cells type2	1187
GTGGGTCAGTAGTGCG-1_CRC1	Epithelial cells type2	1574
AGCGGTCGTTAAGGGC-1_CRC1	Epithelial cells type2	1294
ACATGGTTCCCTCAGT-1_CRC1	Epithelial cells type2	659
GTCACGGTCGTACGGC-1_CRC1	Epithelial cells type2	1253
CCCAATCGTATAGTAG-1_CRC1	Epithelial cells type2	1967
TCTGAGACAACACGCC-1_CRC1	Epithelial cells type2	1472
ATCTGCCTCCTGTAGA-1_CRC1	Epithelial cells type2	942
GCATGTAGTGAAGGCT-1_CRC1	Epithelial cells type2	1171
CTCCTAGTCGGCGCTA-1_CRC1	Epithelial cells type2	1410
TGACTAGGTAGCTCCG-1_CRC1	Epithelial cells type2	1761
GTCGTAAGTGTTAAGA-1_CRC1	Epithelial cells type2	1448
CGGACGTCATCCGCGA-1_CRC1	Epithelial cells type2	1224
CGTAGGCCATTGGTAC-1_CRC1	Epithelial cells type2	1082
CAGCTAAAGACCACGA-1_CRC1	Epithelial cells type2	1543
CTAGTGAAGGTGCTAG-1_CRC1	Epithelial cells type2	1463
GCACTCTAGGTAGCTG-1_CRC1	Epithelial cells type2	1002
TATTACCAGAGCCCAA-1_CRC1	Epithelial cells type2	1280
GCTGCTTTCAACGGCC-1_CRC1	Epithelial cells type2	1525
GTAGTCAAGCTAACAA-1_CRC1	Epithelial cells type2	1447
AAACCTGAGCGAAGGG-1_CRC1	Epithelial cells type2	1192
CGTAGCGTCAACTCTT-1_CRC1	Epithelial cells type2	1021
CTGTGCTCAGTTCATG-1_CRC1	Epithelial cells type2	1121
CAGTCCTCATACCATG-1_CRC1	Epithelial cells type2	1296
AGATCTGAGGTGGGTT-1_CRC1	Epithelial cells type2	902
AGTGAGGCACAGACAG-1_CRC1	Epithelial cells type2	908
CGCGGTAAGCGAGAAA-1_CRC1	Epithelial cells type2	1530

GCATGATCACATCCAA-1_CRC1	Epithelial cells type2	1295
TAGTGGTTCCCTTGTG-1_CRC1	Epithelial cells type2	1779
ATCCGAATCCTTTCTC-1_CRC1	Epithelial cells type2	1684
ATGAGGGAGTCAAGGC-1_CRC1	Epithelial cells type2	1138
CAGCTGGGTGCAGACA-1_CRC1	Epithelial cells type2	1800
GCTGGGTAGCGATGAC-1_CRC1	Epithelial cells type2	1113
TGTCCCAAGCTAGTCT-1_CRC1	Epithelial cells type2	1700
CTTGGCTGTGGCAAAC-1_CRC1	Epithelial cells type2	1327
GTCTTCGGTTACGACT-1_CRC1	Epithelial cells type2	1441
CTCGGAGTCAGGTTCA-1_CRC1	Epithelial cells type2	1290
ATCCACCTCACGCATA-1_CRC1	Epithelial cells type2	1490
GTGCATAGTGCCTGGT-1_CRC1	Epithelial cells type2	1122
CTGATAGCACAGCGTC-1_CRC1	Epithelial cells type2	1244
GAGGTGACAGACGTAG-1_CRC1	Epithelial cells type2	1131
GGATGTTTCGGTCCGA-1_CRC1	Epithelial cells type2	1313
TGTTCCGAGGGATCTG-1_CRC1	Epithelial cells type2	1533
CCGTTCAAGCTGAACG-1_CRC1	Epithelial cells type2	1370
TCTCATACATAGTAAG-1_CRC1	Epithelial cells type2	1181
CACAGTAAGGCGTACA-1_CRC1	Epithelial cells type2	1398
ACGGGCTGTTAGATGA-1_CRC1	Epithelial cells type2	1260
CGATCGGAGATCGATA-1_CRC1	Epithelial cells type2	1391
GGGACCTTCTGGTGTA-1_CRC1	Epithelial cells type2	1433
CTAAGACTCAGCTGGC-1_CRC1	Epithelial cells type2	1562
TGGACGCTCTGGTTCC-1_CRC1	Epithelial cells type2	1231
TTGAACGCACATGTGT-1_CRC1	Epithelial cells type2	1042
CTCTGGTTCCTGCTTG-1_CRC1	Epithelial cells type2	973
CTGCGGAAGACGCAAC-1_CRC1	Epithelial cells type2	860
GTCATTTCAGTAGAGC-1_CRC1	Epithelial cells type2	1127
GTCACAATCAAGCCTA-1_CRC1	Epithelial cells type2	1192
AAACCTGAGTCACGCC-1_CRC1	Epithelial cells type2	1576
TACACGACACCCATGG-1_CRC1	Epithelial cells type2	1597
GCGCCAACATTTCAGG-1_CRC1	Epithelial cells type2	1252
TAAGAGATCAGTTGAC-1_CRC1	Epithelial cells type2	1118
CGTGTCTCAAGTTAAG-1_CRC1	Epithelial cells type2	982
CTAACTTAGTTACCCA-1_CRC1	Epithelial cells type2	851
ATTGGTGTCGGTGTTA-1_CRC1	Epithelial cells type2	1435
TGTCCCATCCAGTATG-1_CRC1	Epithelial cells type2	972
CCACGGACAATGACCT-1_CRC1	Epithelial cells type2	754
TGACGGCGTTATGTGC-1_CRC1	Epithelial cells type2	1218
TGCGGGTAGTGTGAAT-1_CRC1	Epithelial cells type2	747
TTCGAAGCACCGCTAG-1_CRC1	Epithelial cells type2	731
TTGCGTCGTCTCGTTC-1_CRC1	Epithelial cells type2	1132
CATTATCCAGTCGATT-1_CRC1	Epithelial cells type2	1101
GCTCTGTTCTCGTTTA-1_CRC1	Epithelial cells type2	1265
CGGAGCTCAACACGCC-1_CRC1	Epithelial cells type2	1162
CAGCCGAGTTCGGCAC-1_CRC1	Epithelial cells type2	1251
GACGTTACATCTCCCA-1_CRC1	Epithelial cells type2	911
CAGATCAAGATCGATA-1_CRC1	Epithelial cells type2	1066
CAGCTGGAGACAAAGG-1_CRC1	Epithelial cells type2	1269
AAGGCAGAGACTAGGC-1_CRC1	Epithelial cells type2	1253
CGCTGGATCTATGTGG-1_CRC1	Epithelial cells type2	909

ACGCCGAAGGAACTGC-1_CRC1	Epithelial cells type2	964
AGAGTGGTCTGCCAGG-1_CRC1	Epithelial cells type2	892
TCAGCTCGTACACCGC-1_CRC1	Epithelial cells type2	984
TCAGCAATCAGGATCT-1_CRC1	Epithelial cells type2	2415
CTCGGGATCGGCGCAT-1_CRC1	Epithelial cells type2	2075
TGACTAGCAGGACGTA-1_CRC1	Epithelial cells type2	2166
ACAGCTATCAACCAAC-1_CRC1	Epithelial cells type2	1768
GGGTTGCCACATCTTT-1_CRC1	Epithelial cells type2	2298
TCGTAGAAGTTAACGA-1_CRC1	Epithelial cells type2	1460
TGAGGGATCTCTTATG-1_CRC1	Epithelial cells type2	1320
AGAGCGAAGAGTCTGG-1_CRC1	Epithelial cells type2	1466
AGGGTGAGTCTAAAGA-1_CRC1	Epithelial cells type2	1747
TGGACGCAGAGGTAGA-1_CRC1	Epithelial cells type2	1806
AGTGAGGTCTGGCGTG-1_CRC1	Epithelial cells type2	1894
CAGTCCTCATACGCTA-1_CRC1	Epithelial cells type2	1394
CGACCTTTCGCCAAAT-1_CRC1	Epithelial cells type2	1684
CCGTGGATCGTGACAT-1_CRC1	Epithelial cells type2	1169
GACTAACTCAACACGT-1_CRC1	Epithelial cells type2	1517
CACATTTCAAGTACCT-1_CRC1	Epithelial cells type2	899
TTGACTTTCCGAAGAG-1_CRC1	Epithelial cells type2	1063
AAGGCAGAGGCATGTG-1_CRC1	Epithelial cells type2	924
CTAATGGGTCATGCAT-1_CRC1	Epithelial cells type2	1015
AGTGAGGTCACGCATA-1_CRC0	Epithelial cells type2	1035
CGCGTTTCAAGCCGCT-1_CRC1	Epithelial cells type2	1179
CGTAGGCGTGCCTGGT-1_CRC1	Epithelial cells type2	1131
CCGTTCAGTATGAAAC-1_CRC1	Epithelial cells type2	774
GCGACCAAGTTGAGTA-1_CRC1	Epithelial cells type2	1049
CGTGTAATCAAGAAGT-1_CRC1	Epithelial cells type2	1014
TTGGAACAGAGCCCAA-1_CRC2	Epithelial cells type2	1126
TCTTCGGGTGTTTGTG-1_CRC1	Epithelial cells type2	1481
GTGCATATCTGCTGCT-1_CRC1	Epithelial cells type2	1198
AAGGCAGCAGCGATCC-1_CRC1	Epithelial cells type2	1025
CATCGAACAGCGTTCG-1_CRC1	Epithelial cells type2	1308
TACTTACAGATGTGGC-1_CRC1	Epithelial cells type2	617
GTACTCCCAAAGCGGT-1_CRC1	Epithelial cells type2	862
ATTTCTGCATAGAAAC-1_CRC1	Epithelial cells type2	954
CAGCAGCTCGGTCCGA-1_CRC1	Epithelial cells type2	717
CTGCCTACATCGGACC-1_CRC1	Epithelial cells type2	1045
ATCCACCTCAGTTTGG-1_CRC1	Epithelial cells type2	977
ACCCACTTCTCGAGTA-1_CRC1	Epithelial cells type2	749
AGTGTCAGTTTGTTTC-1_CRC1	Epithelial cells type2	947
CCTTCCCCAGCCTATA-1_CRC1	Epithelial cells type2	888
GAATGAATCTACTTAC-1_CRC1	Epithelial cells type2	776
CGCCAAGAGATGCCTT-1_CRC1	Epithelial cells type2	1151
CCACGGAGTTGAGGTG-1_CRC1	Epithelial cells type2	1196
AGCTTGAGTAGTAGTA-1_CRC1	Epithelial cells type2	646
GGGTTGCAGGACCACA-1_CRC1	Epithelial cells type2	1028
TTAGTTCCAATGGATA-1_CRC1	Epithelial cells type2	872
CTGCCTAAGGCTCAGA-1_CRC1	Epithelial cells type2	935
CTTTGCGAGGTGCTTT-1_CRC1	Epithelial cells type2	740
CAGCTGGCACCGGAAA-1_CRC1	Epithelial cells type2	1491

CCGGTAGCATGGTAGG-1_CRC1	Epithelial cells type2	655
TTCGAAGCATTAGCCA-1_CRC1	Epithelial cells type2	951
GCATACAAGGACACCA-1_CRC1	Epithelial cells type2	816
GTGGGTCGTACTTCTT-1_CRC1	Epithelial cells type2	743
AGGCCACTCACTCTTA-1_CRC1	Epithelial cells type2	902
CAGAGAGGTTGTGGAG-1_CRC1	Epithelial cells type2	939
CTAATGGTCACCGGGT-1_CRC1	Epithelial cells type2	784
TGCGGGTCAATAGAGT-1_CRC1	Epithelial cells type2	943
CGTCCATTCGATCCCT-1_CRC1	Epithelial cells type2	1037
AACTCCCAGGTCATCT-1_CRC1	Epithelial cells type2	1030
TCTATTGCACACTGCG-1_CRC1	Epithelial cells type2	964
CACACAAGTCCGTTAA-1_CRC1	Epithelial cells type2	1078
CTACATTAGAACTCGG-1_CRC1	Epithelial cells type2	909
ATCATCTGTGGTGTAG-1_CRC1	Epithelial cells type2	1027
CTAAGACGTGACTACT-1_CRC1	Epithelial cells type2	897
TCAACGAGTAGAGGAA-1_CRC1	Epithelial cells type2	1407
CGCTATCTCCACGTTC-1_CRC1	Epithelial cells type2	1105
TACGGTAGTCTGGAGA-1_CRC1	Epithelial cells type2	1065
AGATCTGTCAAACCGT-1_CRC1	Epithelial cells type2	1056
GGGATGAGTCGCATCG-1_CRC1	Epithelial cells type2	863
AATCCAGTCAACCATG-1_CRC2	Epithelial cells type2	2162
CGTAGCGCAGATCCAT-1_CRC1	Epithelial cells type2	1592
GACAGAGTCCTCAATT-1_CRC1	Epithelial cells type2	1710
CGCCAAGTCGGCATCG-1_CRC1	Epithelial cells type2	1949
TGAGCATTCTGCCCTA-1_CRC1	Epithelial cells type2	1911
CAGTAACCACAACGCC-1_CRC1	Epithelial cells type2	1562
TACTTGTCACAGGTTT-1_CRC1	Epithelial cells type2	2557
CGCCAAGCAGTAACGG-1_CRC1	Epithelial cells type2	2742
TTTGGTTAGACTGGGT-1_CRC2	Epithelial cells type2	960
CTACCCATCTGTCTAT-1_CRC2	Epithelial cells type2	1043
CATTATCCATACTCTT-1_CRC2	Epithelial cells type2	540
CCTACCACATCGATGT-1_CRC2	Epithelial cells type2	734
ACAGCCGGTGATAAAC-1_CRC0	Epithelial cells type2	1837
GAGTCCGGTCTGATTG-1_CRC0	Epithelial cells type2	873
ATCATCTTCTTCATGT-1_CRC2	Epithelial cells type2	1204
AGCTTGATCTATCCCG-1_CRC2	Epithelial cells type2	1080
TTCGGTCAGAAGCCCA-1_CRC2	Epithelial cells type2	649
CGTGTCTTCCATGAGT-1_CRC2	Epithelial cells type2	1582
AAAGTAGTCGTGACAT-1_CRC1	Epithelial cells type2	1761
CGAATGTCATCGACGC-1_CRC1	Epithelial cells type2	1412
CCGTGGACATTCCTCG-1_CRC2	Epithelial cells type2	921
GCAATCACATCGTCGG-1_CRC1	Epithelial cells type2	1649
GGTGCGTGTCCAGTTA-1_CRC1	Epithelial cells type2	1187
CCATGTCCAAGGACAC-1_CRC2	Epithelial cells type2	1450
TCTATTGAGCTAAGAT-1_CRC2	Epithelial cells type2	1643
CTCGGAGCACACAGAG-1_CRC3	Epithelial cells type2	1900
GCAGCCAAGGTAAACT-1_CRC2	Epithelial cells type2	1510
TAAGTGCGTATGGTTC-1_CRC3	Epithelial cells type2	1682
CGTGAGCAGAATTCCC-1_CRC3	Epithelial cells type2	924
TTTACTGGTTCACCTC-1_CRC3	Epithelial cells type2	1074
GGATTACCAGACAAGC-1_CRC2	Epithelial cells type2	945

CTGCTGTGTCTAAACC-1_CRC2	Epithelial cells type2	709
GCGACCAAGGACAGAA-1_CRC2	Epithelial cells type2	896
AAACCTGTCAAGGCTT-1_CRC2	Epithelial cells type2	794
TTTATGCGTAGTGAAT-1_CRC2	Epithelial cells type2	910
CTACGTCCACCCATTC-1_CRC2	Epithelial cells type2	690
GGGCACTAGAAGCCCA-1_CRC2	Epithelial cells type2	587
TGTGTTTTCCAAGCCG-1_CRC2	Epithelial cells type2	762
ATCATGGGTTTGCATG-1_CRC2	Epithelial cells type2	756
TAAGTGCAGGGCATGT-1_CRC2	Epithelial cells type2	801
CGTCACTTCTAACTGG-1_CRC1	Epithelial cells type2	422
GATGCTACACGTGAGA-1_CRC2	Epithelial cells type2	599
TGCGCAGAGCCATCGC-1_CRC2	Epithelial cells type2	568
GTCACAAGTACTCAAC-1_CRC2	Epithelial cells type2	510
GACAGAGGTGTGAATA-1_CRC2	Epithelial cells type2	482
TGGACGCGTACTTGAC-1_CRC2	Epithelial cells type2	364
AACTCAGGTGGTAACG-1_CRC2	Epithelial cells type2	864
GCTCTGTAGAGCTATA-1_CRC2	Epithelial cells type2	826
ACGGCCAAGATGCCAG-1_CRC1	Epithelial cells type2	837
AGCTCCTGTCATATGC-1_CRC2	Epithelial cells type2	670
CGAGCCAAGGCGATAC-1_CRC0	Epithelial cells type2	547
ACGAGCCAGGAGTAGA-1_CRC2	Epithelial cells type2	638
ACTTTCACATAAAGGT-1_CRC2	Epithelial cells type2	477
CACTCCACACGTCAGC-1_CRC2	Epithelial cells type2	728
CTTCTCTAGCTGCCCA-1_CRC2	Epithelial cells type2	599
AAGGCAGTCCTTGGTC-1_CRC2	Epithelial cells type2	604
TATTACCAGGGTCTCC-1_CRC2	Epithelial cells type2	538
ATCATGGCACTGTGTA-1_CRC2	Epithelial cells type2	637
CTACCCAGTTGGTTTG-1_CRC2	Epithelial cells type2	383
GTCACAAAGCTAGCCC-1_CRC2	Epithelial cells type2	841
ATTGGTGCATCCCACT-1_CRC2	Epithelial cells type2	638
TCATTTGTCTGACCTC-1_CRC2	Epithelial cells type2	473
ACTGAACAGCGATTCT-1_CRC2	Epithelial cells type2	438
CTCAGAAGTGTGACCC-1_CRC2	Epithelial cells type2	558
AGCAGCCGTTGTCGCG-1_CRC2	Epithelial cells type2	568
CACCTTGTCGCCTGTT-1_CRC2	Epithelial cells type2	535
CGTAGCGTCTTGACGA-1_CRC2	Epithelial cells type2	498
GCAAACTCAATGACCT-1_CRC2	Epithelial cells type2	412
TGGACGCAGTACGCCC-1_CRC2	Epithelial cells type2	588
CGATGTACACCCTATC-1_CRC2	Epithelial cells type2	452
GGCGACTAGGCTCATT-1_CRC2	Epithelial cells type2	455
GCGCCAACAAGCTGAG-1_CRC2	Epithelial cells type2	782
ATCTGCCCACATTAGC-1_CRC2	Epithelial cells type2	701
GGCAATTAGCAGGTCA-1_CRC2	Epithelial cells type2	600
TAAGCGTCAACACCCG-1_CRC2	Epithelial cells type2	1542
TCAGCTCCACACCGCA-1_CRC2	Epithelial cells type2	277
GGGAATGAGCAATCTC-1_CRC2	Epithelial cells type2	214
TGACTAGTCATCACCC-1_CRC2	Epithelial cells type2	181
AGCCTAACATCCGTGG-1_CRC2	Epithelial cells type2	650
TGCCAAATCGCGATCG-1_CRC2	Epithelial cells type2	422
CTGCTGTCAACACCCG-1_CRC2	Epithelial cells type2	296
TTTGCGCTCATTCACT-1_CRC2	Epithelial cells type2	233

CCGGGATGTAAAGTCA-1_CRC2	Epithelial cells type2	419
ATCCACCTCATGGTCA-1_CRC2	Epithelial cells type2	271
CCTAAAGAGGACATTA-1_CRC2	Epithelial cells type2	256
AACGTTGCAGTATAAG-1_CRC2	Epithelial cells type2	665
AACTCCCGTTCCACTC-1_CRC2	Epithelial cells type2	511
AACTCTTTCAGTCCCT-1_CRC2	Epithelial cells type2	441
CAACTAGCAAAGTGCG-1_CRC2	Epithelial cells type2	627
GACGCGTGTAATAGCA-1_CRC2	Epithelial cells type2	580
CCTAAAGCATGGTTGT-1_CRC2	Epithelial cells type2	910
TAAACCGCATTCTCAT-1_CRC2	Epithelial cells type2	664
TGAGCCGTCACCACCT-1_CRC2	Epithelial cells type2	585
AGTGGGAAGCGTTTAC-1_CRC2	Epithelial cells type2	715
TGTGGTATCCTGTACC-1_CRC2	Epithelial cells type2	726
TCTTTCCGTCAGCTAT-1_CRC2	Epithelial cells type2	703
AACCGCGCAGATCCAT-1_CRC2	Epithelial cells type2	719
GGGAGATTCGAGCCCA-1_CRC2	Epithelial cells type2	562
CGCTGGAAGATGAGAG-1_CRC0	Epithelial cells type2	908
GCTGGGTGTCCGAGTC-1_CRC0	Epithelial cells type2	615
TTGACTTTCAGCAACT-1_CRC0	Epithelial cells type2	729
TTTACTGGTGGTCCGT-1_CRC0	Epithelial cells type2	871
CGCGTTTGTCCGAATT-1_CRC2	Epithelial cells type2	1552
GGGAATGGTATAGTAG-1_CRC1	Epithelial cells type2	1173
TAGCCGGCAAAGGTGC-1_CRC1	Epithelial cells type2	563
TTATGCTCATGTCGAT-1_CRC1	Epithelial cells type2	734
AACACGTTCGAATGCT-1_CRC2	Epithelial cells type2	1683
TTATGCTCATCTACGA-1_CRC2	Epithelial cells type2	1393
TGGTTAGTCCCAAGTA-1_CRC2	Epithelial cells type2	1388
CACTCCAGTACTTGAC-1_CRC2	Epithelial cells type2	906
CGTCAGGCACGGCGTT-1_CRC2	Epithelial cells type2	1500
CAAGAAATCAGCTCTC-1_CRC2	Epithelial cells type2	1510
TACTCATCACGAAATA-1_CRC2	Epithelial cells type2	1039
CTGTGCTGTCTAACGT-1_CRC2	Epithelial cells type2	1717
CTAGAGTAGTACTTGC-1_CRC2	Epithelial cells type2	1077
ACACTGAAGAAGATTC-1_CRC2	Epithelial cells type2	1053
ATTCTACAGTTAAGTG-1_CRC2	Epithelial cells type2	1029
CCCAATCTCCGCATAA-1_CRC2	Epithelial cells type2	185
TTTCCTCGTCGGCTCA-1_CRC2	Epithelial cells type2	876
TTAGGACCACCAGCAC-1_CRC2	Epithelial cells type2	858
GTGCATAAGATCCGAG-1_CRC2	Epithelial cells type2	653
CACCAGGAGGGCTCTC-1_CRC2	Epithelial cells type2	452
TGCCCATCATGTCCTC-1_CRC2	Epithelial cells type2	506
CGTTCTGAGGCCATAG-1_CRC2	Epithelial cells type2	1463
AACGTTGAGGCTAGGT-1_CRC2	Epithelial cells type2	1301
AAGTCTGCAGCCTTTC-1_CRC2	Epithelial cells type2	1459
CATCAGACAGTTAACC-1_CRC2	Epithelial cells type2	1343
CATGCCTTCAACTCTT-1_CRC2	Epithelial cells type2	1018
CCACGGAAGTGCGATG-1_CRC2	Epithelial cells type2	1106
TGACTAGAGAGTACCG-1_CRC2	Epithelial cells type2	1221
TCAGATGAGGAGCGAG-1_CRC2	Epithelial cells type2	1280
CACATTTTCGGCGCAT-1_CRC2	Epithelial cells type2	935
CGCGTTTTCCCTTGCA-1_CRC2	Epithelial cells type2	769

GAAATGAGTCAAAGAT-1_CRC2	Epithelial cells type2	1043
GACTACAGTCCGCTGA-1_CRC2	Epithelial cells type2	811
GCAATCACAGTGAGTG-1_CRC2	Epithelial cells type2	1697
CATATTCCAAGGACTG-1_CRC2	Epithelial cells type2	871
TCAGGATCATGGAATA-1_CRC2	Epithelial cells type2	892
TGTGGTACAAACCTAC-1_CRC1	Epithelial cells type3	2240
AAGCCGCTCGTTACAG-1_CRC1	Epithelial cells type3	1505
GACACGCCAGTACACT-1_CRC1	Epithelial cells type3	1234
GTCGTAAAGGACATTA-1_CRC1	Epithelial cells type3	1153
TATGCCCGTGCAGGTA-1_CRC1	Epithelial cells type3	1023
CATCGGGCAGGACGTA-1_CRC1	Epithelial cells type3	868
CATATGGGTCTAGTCA-1_CRC1	Epithelial cells type3	1090
TACTTGTCAGAGTGTG-1_CRC1	Epithelial cells type3	1002
AAGGAGCCAGACGCCT-1_CRC1	Epithelial cells type3	1364
ACGAGCCCAGTAAGAT-1_CRC1	Epithelial cells type3	1171
CCTACACTCAATAAGG-1_CRC1	Epithelial cells type3	816
CCTACCAGTACCTACA-1_CRC1	Epithelial cells type3	958
AGCATACAGTATGACA-1_CRC1	Epithelial cells type3	1084
CGGAGCTCACGACGAA-1_CRC1	Epithelial cells type3	1547
CTCACACTCAGCAACT-1_CRC1	Epithelial cells type3	1582
GTCATTTTCGTTGACA-1_CRC1	Epithelial cells type3	1270
CAGCCGAGTACTTGAC-1_CRC1	Epithelial cells type3	1008
GAACATCCAAGCCTAT-1_CRC1	Epithelial cells type3	1211
TAGTGGTTCGTTTAGG-1_CRC1	Epithelial cells type3	1347
CCGGGATGTGACGCCT-1_CRC1	Epithelial cells type3	1313
CTCAGAAGTCGTTGTA-1_CRC1	Epithelial cells type3	1121
GCTGCAGAGGATGCGT-1_CRC1	Epithelial cells type3	1291
AGAGCGAAGTGGTCCC-1_CRC1	Epithelial cells type3	1610
GCATGTATCTTGCAAG-1_CRC1	Epithelial cells type3	1262
GCATGTAAGGCTCAGA-1_CRC1	Epithelial cells type3	2038
CACAGTACACGAGGTA-1_CRC1	Epithelial cells type3	945
ATTGGTGTCATAAAGG-1_CRC1	Epithelial cells type3	907
ACGGCCACAGTCAGAG-1_CRC1	Epithelial cells type3	938
CAGCAGCGTACACCGC-1_CRC1	Epithelial cells type3	1107
GGCGTGTAGTCTCAAC-1_CRC1	Epithelial cells type3	1245
GGGAGATGTTTGACAC-1_CRC1	Epithelial cells type3	1602
GAAATGAGTGACAAAT-1_CRC1	Epithelial cells type3	1065
TCGAGGCGTGAGTGAC-1_CRC1	Epithelial cells type3	1187
TTCGGTCCAGTAAGCG-1_CRC1	Epithelial cells type3	1436
TCACGAAAGGATGCGT-1_CRC2	Epithelial cells type3	2188
CATATGGTCCCGGATG-1_CRC1	Epithelial cells type3	1544
CTGAAACCATCCTAGA-1_CRC2	Epithelial cells type3	1502
CTACCCAAGCTGCAAG-1_CRC1	Epithelial cells type3	1065
CGGTTAAAGCTGTTCA-1_CRC2	Epithelial cells type3	1216
TTCCCAGAGAAACGAG-1_CRC2	Epithelial cells type3	851
ACGCCAGTCCTAGGGC-1_CRC1	Epithelial cells type3	2813
GAACGGAAGTGTGGCA-1_CRC1	Epithelial cells type3	1181
GTACTTTTCGTAGATC-1_CRC1	Epithelial cells type3	1059
TAGACCACAGTAGAGC-1_CRC1	Epithelial cells type3	1018
TTAGGCAAGATGTTAG-1_CRC1	Epithelial cells type3	1258
CCTTACGGTGCGCTTG-1_CRC1	Epithelial cells type3	858

TTCGAAGAGCTCCTTC-1_CRC1	Epithelial cells type3	1117
GACCTGGCACCCAGTG-1_CRC1	Epithelial cells type3	1513
AAGTCTGCACAAGTAA-1_CRC1	Epithelial cells type3	909
CTCTACGAGTGTTAGA-1_CRC1	Epithelial cells type3	1104
GTCGGGTAGGCAAAGA-1_CRC1	Epithelial cells type3	1267
CCCTCCTAGCTCCTTC-1_CRC1	Epithelial cells type3	1605
CAGAATCAGGGCTCTC-1_CRC1	Epithelial cells type3	1208
ACGCCGAGTACCATCA-1_CRC1	Epithelial cells type3	950
CTTGGCTTCAGTTAGC-1_CRC1	Epithelial cells type3	1112
ATTGGACTCCCGGATG-1_CRC1	Epithelial cells type3	941
CAACTAGTCTGGTATG-1_CRC1	Epithelial cells type3	720
TATCTCAGTAAGTGTA-1_CRC1	Epithelial cells type3	1026
TGTATTCAGTGAAGAG-1_CRC1	Epithelial cells type3	1729
CAACTAGCACCCATTC-1_CRC1	Epithelial cells type3	1477
TTTACTGCAATGGAAT-1_CRC1	Epithelial cells type3	1601
TGACAACCAACACGCC-1_CRC1	Epithelial cells type3	1736
ATCCACCAGAGGGCTT-1_CRC1	Epithelial cells type3	1145
CATCAGAGTCTTGCGG-1_CRC1	Epithelial cells type3	1550
CCACGGAAGTGTCTCA-1_CRC1	Epithelial cells type3	1265
TAAACCGAGCGATCCC-1_CRC1	Epithelial cells type3	1814
CCTACCAGTGCCTGTG-1_CRC1	Epithelial cells type3	1730
GCACTCTCACTTAACG-1_CRC1	Epithelial cells type3	1635
CTTCTCTAGCTTTGGT-1_CRC1	Epithelial cells type3	1826
GCACATATCTCGTTTA-1_CRC1	Epithelial cells type3	1018
CGGACACGTTAGGGTG-1_CRC1	Epithelial cells type3	1584
CGGACGTTCTATCCCG-1_CRC1	Epithelial cells type3	1183
GCTTCCAGTCTCCATC-1_CRC1	Epithelial cells type3	1272
TGCGCAGCAGACAGGT-1_CRC1	Epithelial cells type3	1560
CGTTCTGGTCCGTGAC-1_CRC1	Epithelial cells type3	1272
TTATGCTCAAGCTGTT-1_CRC1	Epithelial cells type3	1004
CGGCTAGCAACACCCG-1_CRC1	Epithelial cells type3	1117
TTTGTCAAGATGCCAG-1_CRC1	Epithelial cells type3	1142
CTTAGGAGTAGAGCTG-1_CRC1	Epithelial cells type3	1376
TGTATTCTCCTGTACC-1_CRC1	Epithelial cells type3	974
AAACGGGAGAATCTCC-1_CRC1	Epithelial cells type3	2053
GATCTAGTCTATCCCG-1_CRC1	Epithelial cells type3	1874
GTCGGGTAGCTTTGGT-1_CRC1	Epithelial cells type3	1905
CACAGTACACCAGGTC-1_CRC1	Epithelial cells type3	1465
CGTGAGCTCCGCAGTG-1_CRC1	Epithelial cells type3	1626
GGACATTCAAGCGATG-1_CRC1	Epithelial cells type3	1898
TCGTAGAAGGGATCTG-1_CRC1	Epithelial cells type3	2436
GTATCTTTCAGTTGAC-1_CRC1	Epithelial cells type3	1771
CCGTACTAGCCCTAAT-1_CRC1	Epithelial cells type3	1352
TGGACGCCAATGTTGC-1_CRC1	Epithelial cells type3	1166
CGTCCATCAACGCACC-1_CRC1	Epithelial cells type3	1369
ATAAGAGTCCCGGATG-1_CRC1	Epithelial cells type3	1207
GTCGTAAGTAGCGCAA-1_CRC1	Epithelial cells type3	1324
CACCTTGGTAATCGTC-1_CRC1	Epithelial cells type3	1160
TCAGGTAGTAGCCTCG-1_CRC1	Epithelial cells type3	1330
TGATTTCAGTCGCCGT-1_CRC1	Epithelial cells type3	1336
TAGACCACATGCCTAA-1_CRC0	Epithelial cells type3	2160

CATGCCTTCTAACTTC-1_CRC0	Epithelial cells type3	1839
GAGTCCGAGCGATATA-1_CRC0	Epithelial cells type3	1734
GTCATTTAGTCCAGGA-1_CRC0	Epithelial cells type3	2065
TGCCAAAGTGAGGCTA-1_CRC0	Epithelial cells type3	1330
CATCAGAGTCGACTAT-1_CRC0	Epithelial cells type3	2088
GGAGCAAAGTGACTCT-1_CRC0	Epithelial cells type3	2084
TCAGCAAGTGTTTGTG-1_CRC0	Epithelial cells type3	1725
GAGCAGACAGCCAATT-1_CRC0	Epithelial cells type3	2236
GCATACATCATCTGCC-1_CRC0	Epithelial cells type3	1530
ACCAGTACAATAGCAA-1_CRC0	Epithelial cells type3	2046
CCGTTCAAGCCGGTAA-1_CRC0	Epithelial cells type3	1573
CTAGCCTCACAAGTAA-1_CRC0	Epithelial cells type3	1378
CCTTCGAGTGGTGTAG-1_CRC0	Epithelial cells type3	2074
GATCGCGTCAGAAATG-1_CRC0	Epithelial cells type3	1551
AACGTTGTCAAGGTAA-1_CRC0	Epithelial cells type3	1812
CGGGTCAAGATGTTAG-1_CRC0	Epithelial cells type3	2179
TCTCATATCAAGGTAA-1_CRC0	Epithelial cells type3	1746
GGTGAAGAGAGCTTCT-1_CRC2	Epithelial cells type3	2318
AATCGGTGTCGCATCG-1_CRC2	Epithelial cells type3	1572
CCATGTCTCGGCGCAT-1_CRC2	Epithelial cells type3	2445
GGATGTTGTAGCTTGT-1_CRC1	Epithelial cells type3	1639
AAAGCAAAGAGTGACC-1_CRC1	Epithelial cells type3	1431
GACACGCCAGTCACTA-1_CRC1	Epithelial cells type3	1451
CATGGCGCAGTATAAG-1_CRC1	Epithelial cells type3	1564
ATCGAGTTCCTTGGTC-1_CRC1	Epithelial cells type3	1239
AGCATACAGCCTCGTG-1_CRC1	Epithelial cells type3	1314
CTCTAATCAGGCGATA-1_CRC1	Epithelial cells type3	1350
GTATCTTAGTGAACAT-1_CRC1	Epithelial cells type3	1614
AGGGTGAGTGAGGCTA-1_CRC1	Epithelial cells type3	1885
ATGTGTGTCTCTGTCG-1_CRC1	Epithelial cells type3	1655
CGCCAAGCAGTAAGCG-1_CRC1	Epithelial cells type3	1492
GTTCGGGGTCAGCTAT-1_CRC1	Epithelial cells type3	1395
AACTGGTAGACCTAGG-1_CRC1	Epithelial cells type3	1377
GGATGTTGTTCCAACA-1_CRC1	Epithelial cells type3	1250
AACTCCCCAGGAATCG-1_CRC1	Epithelial cells type3	964
CAGCCGACACAACGTT-1_CRC1	Epithelial cells type3	1401
TAGTTGGCAAAGGTGC-1_CRC1	Epithelial cells type3	1208
TGGCCAGAGAGTGACC-1_CRC1	Epithelial cells type3	1103
TACAGTGAGACGACGT-1_CRC1	Epithelial cells type3	1886
ATTTCTGTCACAGTAC-1_CRC1	Epithelial cells type3	1576
CACCACTTCGTCACGG-1_CRC1	Epithelial cells type3	1509
TCTCATACAGCGATCC-1_CRC1	Epithelial cells type3	1584
TACTTGTGTCTAGGTT-1_CRC1	Epithelial cells type3	1012
TAGTGGTAGTGAATTG-1_CRC1	Epithelial cells type3	1275
GAACATCTCCACGTGG-1_CRC1	Epithelial cells type3	656
CACAGTACAATAAGCA-1_CRC1	Epithelial cells type3	778
ACTATCTAGAGCCTAG-1_CRC1	Epithelial cells type3	542
TCAGGATCATACAGCT-1_CRC1	Epithelial cells type3	761
AATCGGTGTAGCGTCC-1_CRC1	Epithelial cells type3	734
CGGTTAATCCAAATGC-1_CRC1	Epithelial cells type3	924
CTACATTTCATTGCGA-1_CRC1	Epithelial cells type3	654

AGTCTTTCATTCACTT-1_CRC2	Epithelial cells type3	617
AGAGTGGAGCTCCTTC-1_CRC1	Epithelial cells type3	1502
TACTTACTCGTATCAG-1_CRC1	Epithelial cells type3	2161
GCTGGGTTCCCAGGTG-1_CRC1	Epithelial cells type3	1229
CCTCTGACACTGAAGG-1_CRC1	Epithelial cells type3	1505
TCAACGAGTTAAGACA-1_CRC1	Epithelial cells type3	1505
CAGCTAACAAGCCGTC-1_CRC1	Epithelial cells type3	1183
CGCTGGACAATGGAGC-1_CRC1	Epithelial cells type3	976
GTAGTCACATTTCACT-1_CRC1	Epithelial cells type3	1186
CGTTAGATCGCTGATA-1_CRC1	Epithelial cells type3	1226
ACATACGAGCAATATG-1_CRC1	Epithelial cells type3	878
CTAAGACAGATAGGAG-1_CRC1	Epithelial cells type3	1063
TTGCCGTAGTACGTTC-1_CRC1	Epithelial cells type3	1042
ACTTTCACAGGAATGC-1_CRC1	Epithelial cells type3	1375
GTAGTCATCGTAGGTT-1_CRC1	Epithelial cells type3	870
CGGACTGTCTGCTTGC-1_CRC1	Epithelial cells type3	2897
TTCTACATCCAAGCCG-1_CRC1	Epithelial cells type3	1415
ATCATCTGTGACTCAT-1_CRC1	Epithelial cells type3	1467
ACGATGTAGATCCCGC-1_CRC1	Epithelial cells type3	1801
TTGACTTTCGAATGGG-1_CRC1	Epithelial cells type3	1141
CATCAAGAGGACGAAA-1_CRC1	Epithelial cells type3	1652
ATGTGTGCACATTCGA-1_CRC1	Epithelial cells type3	1616
ACTGAACCAGACGTAG-1_CRC1	Epithelial cells type3	1221
CAGAATCTCAGCACAT-1_CRC1	Epithelial cells type3	1315
CTGAAGTAGAGTTGGC-1_CRC1	Epithelial cells type3	1715
TGACAACGTCCAGTAT-1_CRC1	Epithelial cells type3	1098
AACTCTTCACGAAACG-1_CRC1	Epithelial cells type3	1235
TCACAAGTCTAACTGG-1_CRC1	Epithelial cells type3	1982
GACGGCTTCATAGCAC-1_CRC1	Epithelial cells type3	1059
GGACAAGAGAGACGAA-1_CRC1	Epithelial cells type3	1882
GTATTCTCACATGGGA-1_CRC1	Epithelial cells type3	1669
GACTGCGTCCATGCTC-1_CRC1	Epithelial cells type3	1284
GTAACGTCACAACGCC-1_CRC1	Epithelial cells type3	1105
AGGGTGAAGAAACCTA-1_CRC1	Epithelial cells type3	1222
CTAGAGTAGCTAGTCT-1_CRC1	Epithelial cells type3	813
GCATGTACACAAGACG-1_CRC1	Epithelial cells type3	1286
TGCACCTCAAGTCATC-1_CRC1	Epithelial cells type3	987
CGGACACGTGTTAAGA-1_CRC1	Epithelial cells type3	1086
GCTTCCAAGTGGTAAT-1_CRC1	Epithelial cells type3	946
ATGTGTGAGCCTCGTG-1_CRC1	Epithelial cells type3	602
CGATGTAGTACTCAAC-1_CRC1	Epithelial cells type3	945
CTCGTCAAGTCAAGCG-1_CRC1	Epithelial cells type3	1084
CAAGTTGCAGTATAAG-1_CRC1	Epithelial cells type3	1363
GTGTGCGCAGACAAGC-1_CRC1	Epithelial cells type3	987
CTCATTATCGCTTGTC-1_CRC1	Epithelial cells type3	1916
CAGTAACGTTACAGAA-1_CRC1	Epithelial cells type3	1073
CGACCTTTCTGCGGCA-1_CRC1	Epithelial cells type3	1416
CGATGTAGTCGCATCG-1_CRC1	Epithelial cells type3	1391
GCATGCGCATGCTAGT-1_CRC1	Epithelial cells type3	1128
GGCGTGTTCTGAAAGA-1_CRC1	Epithelial cells type3	3171
AACTCTTGTTCGTCTC-1_CRC1	Epithelial cells type3	1835

CCGGTAGTCTCCGGTT-1_CRC1	Epithelial cells type3	1243
CAGAATCAGCGTAATA-1_CRC1	Epithelial cells type3	1424
CTAATGGAGGGCTTCC-1_CRC1	Epithelial cells type3	1659
CACTCCAAGGATGCGT-1_CRC1	Epithelial cells type3	1286
CAGAGAGCATGCATGT-1_CRC1	Epithelial cells type3	735
TCGTAGAAGAGGTAGA-1_CRC1	Epithelial cells type3	1220
AAGGAGCTCTGCTTGC-1_CRC1	Epithelial cells type3	1128
ATAGACCAGTGGACGT-1_CRC1	Epithelial cells type3	1132
CGGCTAGGTCTGATTG-1_CRC1	Epithelial cells type3	977
CTCATTAGTAGCGCAA-1_CRC1	Epithelial cells type3	805
GGAATAAGTCGCTTCT-1_CRC1	Epithelial cells type3	1181
GATCTAGGTACATGTC-1_CRC1	Epithelial cells type3	975
CATATTCAGGCTATCT-1_CRC1	Epithelial cells type3	1092
ACGATGTTCTCGGACG-1_CRC1	Epithelial cells type3	873
GGGCACTTCCTAGGGC-1_CRC1	Epithelial cells type3	1097
TTGACTTGTATATGGA-1_CRC1	Epithelial cells type3	1699
ATTATCCAGGTGACCA-1_CRC1	Epithelial cells type3	1508
GTTACAGCATCCTAGA-1_CRC1	Epithelial cells type3	1003
CATGGCGCAAAGGTGC-1_CRC1	Epithelial cells type3	760
CTGAAACAGAAGGGTA-1_CRC1	Epithelial cells type3	713
CTCGAGGAGCCCAGCT-1_CRC1	Epithelial cells type3	1196
TGGCCAGCATCCGGGT-1_CRC1	Epithelial cells type3	1280
AAAGATGTCCCATTAT-1_CRC1	Epithelial cells type3	1008
GATGAAAGTCAAAGCG-1_CRC1	Epithelial cells type3	1224
GATGAAATCAGAGACG-1_CRC1	Epithelial cells type3	1487
TACACGACAGGGTTAG-1_CRC1	Epithelial cells type3	1753
GAACATCTCCTTGGTC-1_CRC1	Epithelial cells type3	1067
TCAGCAAGTATATCCG-1_CRC1	Epithelial cells type3	1232
AGCAGCCCACAGACAG-1_CRC1	Epithelial cells type3	765
CCTTCCCAGCTACCTA-1_CRC1	Epithelial cells type3	1377
CGGACACTCACCGTAA-1_CRC1	Epithelial cells type3	1648
ATAGACCCAGGAACGT-1_CRC1	Epithelial cells type3	1754
GACAGAGGTACGCTGC-1_CRC1	Epithelial cells type3	2263
AACTCCCCACGACGAA-1_CRC1	Epithelial cells type3	2789
TGGGAAGCACAAGACG-1_CRC1	Epithelial cells type3	2461
TGCGTGGCACTCGACG-1_CRC1	Epithelial cells type3	2059
TGTGGTACAGGTGGAT-1_CRC1	Epithelial cells type3	1862
AAAGATGGTAGCGCAA-1_CRC1	Epithelial cells type3	1930
CAGCCGAAGAGAGCTC-1_CRC1	Epithelial cells type3	1007
TGATTTCAGCACACAG-1_CRC1	Epithelial cells type3	1211
CCGTACTGTTCGTGAT-1_CRC1	Epithelial cells type3	2862
TTTATGCCACATGGGA-1_CRC1	Epithelial cells type3	1226
CCCTCCTCAATAGAGT-1_CRC1	Epithelial cells type3	1400
TGTATTCGTCTCAACA-1_CRC1	Epithelial cells type3	1704
CATCGAATCAGCCTAA-1_CRC1	Epithelial cells type3	1376
TGCCAAAAGTTTGCGT-1_CRC1	Epithelial cells type3	1215
ATTGGACGTGCCTGTG-1_CRC1	Epithelial cells type3	1187
GCTGGGTTCCGTACAA-1_CRC1	Epithelial cells type3	1079
AAGGTTCTCAAGGTAA-1_CRC1	Epithelial cells type3	1663
TGGCCAGTCACAGGCC-1_CRC1	Epithelial cells type3	1067
GCCTCTATCACCACCT-1_CRC1	Epithelial cells type3	2141

TTGCGTCGTAAGTAGT-1_CRC1	Epithelial cells type3	1346
CTTAACTAGGAGTACC-1_CRC1	Epithelial cells type3	1527
TTAGGACTCAACACTG-1_CRC1	Epithelial cells type3	1138
TCAGCTCAGTATGACA-1_CRC1	Epithelial cells type3	2696
TCAATCTCATTGAGCT-1_CRC1	Epithelial cells type3	1510
TGCTGCTAGTGGGCTA-1_CRC1	Epithelial cells type3	1299
GTGGGTCCACAGCGTC-1_CRC1	Epithelial cells type3	1499
TGCCCTAGTTACGGAG-1_CRC1	Epithelial cells type3	1288
CTCACACAGCTGTTCA-1_CRC1	Epithelial cells type3	1469
GCCTCTATCTGATACG-1_CRC1	Epithelial cells type3	872
TACACGATCCCTAACC-1_CRC1	Epithelial cells type3	975
ATTGGACTCAACGGCC-1_CRC1	Epithelial cells type3	1986
TACTCGCAGTGGTAAT-1_CRC1	Epithelial cells type3	1349
AACTCCCAGGACAGAA-1_CRC1	Epithelial cells type3	1164
GTCCTCAAGCCAGAAC-1_CRC1	Epithelial cells type3	1239
CACAAACTCGCGTTTC-1_CRC1	Epithelial cells type3	1181
TGCGCAGAGTGCCAGA-1_CRC1	Epithelial cells type3	1042
GATCGATTCCGTAGGC-1_CRC1	Epithelial cells type3	1000
TTGCGTCAGAATTGTG-1_CRC1	Epithelial cells type3	753
CGTGTAAGTTGTCTTT-1_CRC1	Epithelial cells type3	963
CTTAGGATCTACTATC-1_CRC1	Epithelial cells type3	992
GCATGCGCACTGCCAG-1_CRC1	Epithelial cells type3	1093
TATGCCCGTACCGTTA-1_CRC1	Epithelial cells type3	1492
TACGGTAGTGAGGCTA-1_CRC1	Epithelial cells type3	953
ACATACGTCGCGGATC-1_CRC1	Epithelial cells type3	872
CTCGAAAGTTGCGCAC-1_CRC1	Epithelial cells type3	1115
TGCGCAGTCAAAGACA-1_CRC1	Epithelial cells type3	996
CGTGAGCAGCAATCTC-1_CRC1	Epithelial cells type3	758
GTCACGGGTCTAACGT-1_CRC1	Epithelial cells type3	1354
CGAACATAGCCACGCT-1_CRC1	Epithelial cells type3	1320
TGTGGTATCTTGCATT-1_CRC1	Epithelial cells type3	1105
ACATCAGAGCTGAAAT-1_CRC1	Epithelial cells type3	1461
ATTCTACAGTCGTTTG-1_CRC1	Epithelial cells type3	1277
AACGTTGGTGGTAACG-1_CRC1	Epithelial cells type3	1196
GCTCCTATCAGCTGGC-1_CRC1	Epithelial cells type3	1219
CTCAGAAAGAATCTCC-1_CRC1	Epithelial cells type3	1208
TTTATGCTCTGTCCGT-1_CRC1	Epithelial cells type3	1251
CGCTGGACAATCTGCA-1_CRC1	Epithelial cells type3	913
CGCGTTTGTATGGTTC-1_CRC1	Epithelial cells type3	1632
CGATTGACACGGTAAG-1_CRC1	Epithelial cells type3	875
TCGTAGATCAGTGTTG-1_CRC1	Epithelial cells type3	1216
ATCACGAGTTGTCTTT-1_CRC1	Epithelial cells type3	1202
CTGATAGGTCTCTCTG-1_CRC1	Epithelial cells type3	917
CGGAGCTCACCGCTAG-1_CRC1	Epithelial cells type3	1623
CACACCTTCACTTACT-1_CRC1	Epithelial cells type3	1492
CGAATGTGTCTCATCC-1_CRC1	Epithelial cells type3	1505
CTGCCTAGTCAGAATA-1_CRC1	Epithelial cells type3	1231
GCTGGGTTCCGTCATC-1_CRC1	Epithelial cells type3	1259
CGGGTCAGTTCAGGCC-1_CRC1	Epithelial cells type3	1629
GGAAAGCGTCTCTTAT-1_CRC1	Epithelial cells type3	1643
ATCACGATCTCGTTTA-1_CRC1	Epithelial cells type3	1308

CTGTTTATCATGCATG-1_CRC1	Epithelial cells type3	1353
GTTACAGGTGACTACT-1_CRC1	Epithelial cells type3	1360
GTGGGTCGTAGCGTCC-1_CRC1	Epithelial cells type3	1399
ACATACGAGATAGCAT-1_CRC1	Epithelial cells type3	885
TATCTCAAGTGTACGG-1_CRC1	Epithelial cells type3	796
TTCTACAAGACGCAAC-1_CRC1	Epithelial cells type3	1196
AGGGAGTTCGTTACGA-1_CRC1	Epithelial cells type3	1127
ACATACGCATCTGGTA-1_CRC1	Epithelial cells type3	929
ATGAGGGGTACTTGAC-1_CRC1	Epithelial cells type3	864
AAGGAGCGTCGGCACT-1_CRC1	Epithelial cells type3	1555
ACGTCAAGTTTCGCTC-1_CRC1	Epithelial cells type3	1020
TTGCCGTAGTGTACGG-1_CRC1	Epithelial cells type3	988
GAGCAGATCCGTACAA-1_CRC1	Epithelial cells type3	1228
GCTGCTTCAGATGGGT-1_CRC1	Epithelial cells type3	1277
CCTCAGTGTACTCTCC-1_CRC1	Epithelial cells type3	855
CGCCAAGCAATCCAAC-1_CRC1	Epithelial cells type3	1013
CGTCACTCACCAGATT-1_CRC1	Epithelial cells type3	1923
TCTATTGGTTATTCTC-1_CRC1	Epithelial cells type3	2098
TTCTTAGAGGACCACA-1_CRC1	Epithelial cells type3	1979
CACAAACGTTGTTTGG-1_CRC1	Epithelial cells type3	1305
GAGTCCGGTGGCTCCA-1_CRC1	Epithelial cells type3	1176
GCGACCACAGGACGTA-1_CRC1	Epithelial cells type3	950
ACACCGGGTATAGGTA-1_CRC1	Epithelial cells type3	895
CTGAAACCAGCTGGCT-1_CRC1	Epithelial cells type3	729
CATTATCCAAACAACA-1_CRC1	Epithelial cells type3	1561
GCGACCAAGGAGTTGC-1_CRC1	Epithelial cells type3	1207
GGTATTGAGCTCCTTC-1_CRC1	Epithelial cells type3	1290
GATGCTAAGGCTAGAC-1_CRC1	Epithelial cells type3	1292
CTGATCCGTAAGTAGT-1_CRC1	Epithelial cells type3	1114
GTCACAAAGAAGGCCT-1_CRC1	Epithelial cells type3	651
CAGAGAGTCGCATGGC-1_CRC1	Epithelial cells type3	997
CTGATAGTCCCTGACT-1_CRC1	Epithelial cells type3	871
GTCACGGGTAACGTTC-1_CRC1	Epithelial cells type3	1259
CTGCTGTCACTAGTAC-1_CRC1	Epithelial cells type3	782
CTGCCTACATCGGAAG-1_CRC1	Epithelial cells type3	834
TTGGCAAGTCGGCACT-1_CRC1	Epithelial cells type3	1061
AACCATGCATAAGACA-1_CRC1	Epithelial cells type3	1354
GGAATAAGTGACAAAT-1_CRC1	Epithelial cells type3	1113
CAGCCGAAGTGTTTGC-1_CRC1	Epithelial cells type3	860
GCACTCTCATGCCACG-1_CRC1	Epithelial cells type3	736
AGTTGGTTCAACGGGA-1_CRC1	Epithelial cells type3	1126
GGCTGGTGTATAGTAG-1_CRC1	Epithelial cells type3	1043
CAACCTCCAATCTGCA-1_CRC1	Epithelial cells type3	1115
TGCCCATTCGAGGTAG-1_CRC1	Epithelial cells type3	1100
ACGCCGACAAGCCGCT-1_CRC1	Epithelial cells type3	1270
GTCGGGTAGTAGGCCA-1_CRC1	Epithelial cells type3	1236
CATGCCTGTCTTGCGG-1_CRC1	Epithelial cells type3	1356
AGATCTGTCAGTCAGT-1_CRC1	Epithelial cells type3	1385
CTGGTCTTCTTATCTG-1_CRC1	Epithelial cells type3	1417
CCCAATCAGATCCGAG-1_CRC1	Epithelial cells type3	1115
AGGGATGAGAGCTGGT-1_CRC1	Epithelial cells type3	911

CGGACGTTCAGCAACT-1_CRC1	Epithelial cells type3	1124
CGAGAAGGTGCGAAAC-1_CRC1	Epithelial cells type3	741
TTAACTCCAGCTGTGC-1_CRC1	Epithelial cells type3	571
GGTGCGTCAGATGAGC-1_CRC1	Epithelial cells type3	808
AAGGTTCCAACACGCC-1_CRC1	Epithelial cells type3	1115
GTAGGCCGTTGTGGCC-1_CRC1	Epithelial cells type3	1002
AAGACCTCATTGGGCC-1_CRC1	Epithelial cells type3	714
TATCTCAGTACCATCA-1_CRC1	Epithelial cells type3	998
GGAAAGCAGGTGATAT-1_CRC1	Epithelial cells type3	994
CATGGCGCAGACGCCT-1_CRC1	Epithelial cells type3	728
GGCGTGTTCAGCTTAG-1_CRC1	Epithelial cells type3	858
ACTGATGTCCGTAGTA-1_CRC1	Epithelial cells type3	1075
AGGGATGCACCTCGGA-1_CRC1	Epithelial cells type3	1002
GACGTTAGTTTGCATG-1_CRC1	Epithelial cells type3	1260
TACTTGTAGTCCGTAT-1_CRC1	Epithelial cells type3	1210
CAGCGACGTCCGAGTC-1_CRC1	Epithelial cells type3	1002
CGTCAGGGTCGAGTTT-1_CRC1	Epithelial cells type3	852
CGCTGGACAGGGTTAG-1_CRC1	Epithelial cells type3	1436
GTGCATAGTATTCGTG-1_CRC1	Epithelial cells type3	1247
CTACACCAGGGATGGG-1_CRC1	Epithelial cells type3	1271
TTCGAAGAGTCGAGTG-1_CRC1	Epithelial cells type3	1311
TAAGAGAAGCGCTTAT-1_CRC1	Epithelial cells type3	1126
TTTGGTTTCCTAGTGA-1_CRC1	Epithelial cells type3	880
AGCGGTCGTCGTTGTA-1_CRC1	Epithelial cells type3	1013
TATCAGGGTGCAGTAG-1_CRC1	Epithelial cells type3	832
GCGACCAGTGGCTCCA-1_CRC1	Epithelial cells type3	843
GTGCATACAGGGTACA-1_CRC1	Epithelial cells type3	916
AGTGGGATCTGGCGAC-1_CRC1	Epithelial cells type3	1443
TGGCCAGGTGTATGGG-1_CRC1	Epithelial cells type3	1190
TGACTAGGTGCAACTT-1_CRC1	Epithelial cells type3	1044
GTACTTTGTGGCAAAC-1_CRC1	Epithelial cells type3	979
TCAGCAAAGAGCTGCA-1_CRC1	Epithelial cells type3	832
ATCCACCAGGCGCTCT-1_CRC1	Epithelial cells type3	1367
CTGATAGTCATTGCCC-1_CRC1	Epithelial cells type3	949
GACGGCTCAATCACAC-1_CRC1	Epithelial cells type3	1069
GTATTCTAGATCTGAA-1_CRC1	Epithelial cells type3	994
CAGAGAGAGAAGGGTA-1_CRC1	Epithelial cells type3	1232
TTGGCAAGTCACTGGC-1_CRC1	Epithelial cells type3	1073
AGCTTGAGTAGCGTCC-1_CRC1	Epithelial cells type3	1087
CTACGTCAGCGACGTA-1_CRC1	Epithelial cells type3	1111
AACTGGTAGCTAACTC-1_CRC1	Epithelial cells type3	1133
CCGTACTCATGGATGG-1_CRC1	Epithelial cells type3	916
AACTCTTAGACACGAC-1_CRC1	Epithelial cells type3	1328
GTGCGGTGTTTGTTGG-1_CRC1	Epithelial cells type3	1505
CAACCTCAGGGCATGT-1_CRC2	Epithelial cells type4	4776
CTCTAATCAGATGGCA-1_CRC2	Epithelial cells type4	4012
AGGCCACTCCGTAGTA-1_CRC2	Epithelial cells type4	2008
TCGCGTTCAGTCCTTC-1_CRC2	Epithelial cells type4	1989
ACGTCAAGTCTAGGTT-1_CRC2	Epithelial cells type4	1966
GGGACCTGTATGAATG-1_CRC2	Epithelial cells type4	2075
TCGCGAGCAGCTGCAC-1_CRC2	Epithelial cells type4	2129

TGGTTCCAGGCACATG-1_CRC2	Epithelial cells type4	2229
CGACTTCGTCTGATCA-1_CRC2	Epithelial cells type4	2356
CGTGAGCTCGGTCTAA-1_CRC2	Epithelial cells type4	2539
ACGGAGAAGTCGCCGT-1_CRC2	Epithelial cells type4	1431
GACGCGTCACGGACAA-1_CRC2	Epithelial cells type4	1479
TGACAACGTGTGTGCC-1_CRC2	Epithelial cells type4	2196
CAACTAGAGGCAAAGA-1_CRC2	Epithelial cells type4	1732
CGATTGACACCTGGTG-1_CRC2	Epithelial cells type4	2611
ACGCCAGAGGAACTGC-1_CRC2	Epithelial cells type4	2162
CCTCTGAAGACTTGAA-1_CRC2	Epithelial cells type4	2077
GGAAAGCGTAGTAGTA-1_CRC2	Epithelial cells type4	1789
TGCGTGGTCCCAGGTG-1_CRC2	Epithelial cells type4	1781
CCCAATCTCTCGAGTA-1_CRC2	Epithelial cells type4	2290
GTGTTAGGTAATAGCA-1_CRC2	Epithelial cells type4	2273
TGGTTCCTCAACGGCC-1_CRC2	Epithelial cells type4	2196
CGATGTATCTCGCTTG-1_CRC2	Epithelial cells type4	2350
CTCCTAGGTAACGCGA-1_CRC2	Epithelial cells type4	2225
ATAGACCAGGTAGCTG-1_CRC2	Epithelial cells type4	1604
CAAGGCCTCGTAGATC-1_CRC2	Epithelial cells type4	1921
TTCTCAACATCAGTAC-1_CRC2	Epithelial cells type4	1527
AACTCTTAGGATGTAT-1_CRC2	Epithelial cells type4	2197
GAGCAGATCATCTGCC-1_CRC2	Epithelial cells type4	2632
GTCCTCATCTCTGAGA-1_CRC2	Epithelial cells type4	3180
TGTCCCACACTCAGGC-1_CRC2	Epithelial cells type4	2845
ACACTGAGTATAGTAG-1_CRC2	Epithelial cells type4	2347
GCACATAGTCGAAAGC-1_CRC2	Epithelial cells type4	2444
GGACATTCAAACCTAC-1_CRC2	Epithelial cells type4	1222
TGACTTTGTATATGAG-1_CRC2	Epithelial cells type4	1861
CCGTGGAAGGTGTGGT-1_CRC2	Epithelial cells type4	1275
TGGGAAGAGTACACCT-1_CRC2	Epithelial cells type4	1623
TTGCGTCAGATCACGG-1_CRC2	Epithelial cells type4	1050
CATCCACGTCACCTAA-1_CRC2	Epithelial cells type4	1765
TCATTACAGCAGATCG-1_CRC2	Epithelial cells type4	1296
ACTGAGTGTTGTCTTT-1_CRC2	Epithelial cells type4	2078
ACGCCGACATGCCTAA-1_CRC2	Epithelial cells type4	3441
TCGGGACTCAAGGTAA-1_CRC2	Epithelial cells type4	3296
CACATAGAGTTTAGGA-1_CRC2	Epithelial cells type4	2380
AGTGAGGAGCCACGCT-1_CRC2	Epithelial cells type4	2616
AGCTTGAAGTCCATAC-1_CRC2	Epithelial cells type4	1998
CACCAGGTCGTGGACC-1_CRC2	Epithelial cells type4	1628
CCGTGGAAGCAATATG-1_CRC2	Epithelial cells type4	1450
ATAGACCCATGACGGA-1_CRC2	Epithelial cells type4	1555
CGTTAGAAGTTAGGTA-1_CRC2	Epithelial cells type4	1880
AGATTGCGTCTTCTCG-1_CRC2	Epithelial cells type4	1891
GACTGCGGTACTCAAC-1_CRC2	Epithelial cells type4	1329
ACGAGCCAGGGTCTCC-1_CRC2	Epithelial cells type4	1269
CTCGAGGGTTGATTCG-1_CRC2	Epithelial cells type4	2101
GGGCACTGTTAGATGA-1_CRC2	Epithelial cells type4	1943
CAAGTTGAGAGTAATC-1_CRC2	Epithelial cells type4	1575
CAGCCGACAGTATGCT-1_CRC2	Epithelial cells type4	1209
ACCGTAAAGAATTGTG-1_CRC2	Epithelial cells type4	2362

CCATGTCGTAAGTAGT-1_CRC2	Epithelial cells type4	1803
CCTATTACAGTATCTG-1_CRC2	Epithelial cells type4	2815
AATCCAGCACGACTCG-1_CRC2	Epithelial cells type4	2542
GCACATAGTAGCGTAG-1_CRC2	Epithelial cells type4	2755
CAGCTAACAATCGAAA-1_CRC2	Epithelial cells type4	2184
AGGCCGTAGTCACGCC-1_CRC2	Epithelial cells type4	2212
GCCTCTAGTCGCTTTC-1_CRC2	Epithelial cells type4	2231
CATATGGAGGGCTCTC-1_CRC2	Epithelial cells type4	2451
GTCGGGTAGAATTGTG-1_CRC2	Epithelial cells type4	2577
CATCGGGGTCGAATCT-1_CRC2	Epithelial cells type4	2134
CAGTAACCAGGTCCAC-1_CRC2	Epithelial cells type4	2047
GTGCAGCAGGCCCTCA-1_CRC2	Epithelial cells type4	1703
AGCTTGAGTGCCTGGT-1_CRC2	Epithelial cells type4	1666
TCATTTGCAGTATAAG-1_CRC2	Epithelial cells type4	2414
CATCGAAGTAGAAGGA-1_CRC2	Epithelial cells type4	2423
GCAGTTAGTTAGTGGG-1_CRC2	Epithelial cells type4	2165
ACGCCGAGTCTCCCTA-1_CRC2	Epithelial cells type4	1790
GCCTCTACATACTCTT-1_CRC2	Epithelial cells type4	1945
GGACAGAGTCGCGTGT-1_CRC2	Epithelial cells type4	2297
TTTGGTTCACATGTGT-1_CRC2	Epithelial cells type4	2094
ACATACGTCATCACCC-1_CRC2	Epithelial cells type4	2068
CAACCTCTCGCTAGCG-1_CRC2	Epithelial cells type4	2134
ACCTTTAGTTCCTCCA-1_CRC2	Epithelial cells type4	2229
GCTGCTTAGCTCAACT-1_CRC2	Epithelial cells type4	2483
TACACGACATGCCCGA-1_CRC2	Epithelial cells type4	2002
TTGGCAACACTACAGT-1_CRC2	Epithelial cells type4	2109
CGCCAAGTCTGATTCT-1_CRC2	Epithelial cells type4	2596
GCAAACTGTTCGTTGA-1_CRC2	Epithelial cells type4	2465
CCGTTCAAGTTTAGGA-1_CRC2	Epithelial cells type4	2271
ACGCCAGGTAGATTAG-1_CRC2	Epithelial cells type4	1916
CTAGCCTTCGGCGGTT-1_CRC2	Epithelial cells type4	1986
CATGCCTCAGCTTAAC-1_CRC2	Epithelial cells type4	2273
CAGATCAGTTCACCTC-1_CRC2	Epithelial cells type4	1689
TACTTACTCCGAATGT-1_CRC2	Epithelial cells type4	2115
CTGTGCTAGCTGATAA-1_CRC2	Epithelial cells type4	2250
GGGATGACACTTAACG-1_CRC2	Epithelial cells type4	2140
AACTCCCGTTTGACTG-1_CRC2	Epithelial cells type4	1936
GTGTGCGGTTCCTCCA-1_CRC2	Epithelial cells type4	1844
ACACCCTTCCGCGTTT-1_CRC2	Epithelial cells type4	2613
ACATCAGAGCCAGGAT-1_CRC2	Epithelial cells type4	2300
CGATTGAAGCCGGTAA-1_CRC2	Epithelial cells type4	1714
GGCTGGTCATTGTGCA-1_CRC2	Epithelial cells type4	1698
GATGAAATCGCAGGCT-1_CRC2	Epithelial cells type4	1497
TTTGCGCAGTATGACA-1_CRC2	Epithelial cells type4	2026
AAAGCAAGTTTAGGAA-1_CRC2	Epithelial cells type4	1790
CGATGGCCAAGCCGCT-1_CRC2	Epithelial cells type4	2001
TTCGGTCCACATTTCT-1_CRC2	Epithelial cells type4	3656
CGCTGGAGTAACGACG-1_CRC2	Epithelial cells type4	3336
CATATGGTCTGTCTCG-1_CRC2	Epithelial cells type4	2897
TGTATTCGTATGAAAC-1_CRC2	Epithelial cells type4	3129
ACGAGCCTCGCCATAA-1_CRC2	Epithelial cells type4	3173

AACTCTTAGTACTTGC-1_CRC2	Epithelial cells type4	2956
AACCGCGCATTGGTAC-1_CRC2	Epithelial cells type4	3008
GCTCCTACAAGCTGAG-1_CRC2	Epithelial cells type4	2888
CGAATGTGTATAATGG-1_CRC2	Epithelial cells type4	2106
TATTACCCACGGTAAG-1_CRC2	Epithelial cells type4	3304
GTTTCTAGTCAGCTAT-1_CRC2	Epithelial cells type4	3901
CTGATCCAGCCTATGT-1_CRC2	Epithelial cells type4	3522
TAGACCATCGCAAGCC-1_CRC2	Epithelial cells type4	3400
TTCTACACATATACCG-1_CRC2	Epithelial cells type4	3014
GATGAAACATCACGAT-1_CRC2	Epithelial cells type4	2961
GGACAGAAGCAGACTG-1_CRC2	Epithelial cells type4	3190
CACCTTGTCTATCCTA-1_CRC2	Epithelial cells type4	2301
CATCGAAGTGTGACCC-1_CRC2	Epithelial cells type4	3250
GAGTCCGAGACAGAGA-1_CRC2	Epithelial cells type4	2453
TGCACCTGTAACGTTC-1_CRC2	Epithelial cells type4	2637
ATAACGCAGATATACG-1_CRC2	Epithelial cells type4	2332
CTCTAATCACGACTCG-1_CRC2	Epithelial cells type4	2659
GGATGTTAGAGCCTAG-1_CRC2	Epithelial cells type4	3201
CACATAGCATTTCACT-1_CRC2	Epithelial cells type4	1969
TTCGAAGAGCCACCTG-1_CRC2	Epithelial cells type4	2504
ATCATGGGTTTGTTGG-1_CRC2	Epithelial cells type4	2530
CGTCTACGTCATTAGC-1_CRC2	Epithelial cells type4	2092
AACACGTGTGTAATGA-1_CRC2	Epithelial cells type4	2704
GCAGCCATCACCGTAA-1_CRC2	Epithelial cells type4	2785
TACTTACGTATCAGTC-1_CRC2	Epithelial cells type4	3327
CCACGGATCAGGCAAG-1_CRC2	Epithelial cells type4	2656
CACTCCAGTCTCCCTA-1_CRC2	Epithelial cells type4	2571
CCGTTCATCACCTCGT-1_CRC2	Epithelial cells type4	2841
TGCACCTTCTTCTGGC-1_CRC2	Epithelial cells type4	2794
AGTGAGGTCTCGCATC-1_CRC2	Epithelial cells type4	1841
CCACGGACATTCTTAC-1_CRC2	Epithelial cells type4	2629
CGGGTCAAGACCGGAT-1_CRC2	Epithelial cells type4	2862
TCTGGAAGTCCCTACT-1_CRC2	Epithelial cells type4	2329
ATCATCTCACAGATTC-1_CRC2	Epithelial cells type4	2251
ACTGTCCAGTGAACAT-1_CRC2	Epithelial cells type4	1625
CGAGAAGCACGAAACG-1_CRC2	Epithelial cells type4	2047
GATGAGGTCAAAGTAG-1_CRC2	Epithelial cells type4	2408
CCGTACTGTCGCGAAA-1_CRC2	Epithelial cells type4	2023
CTTAGGATCAAACAAG-1_CRC2	Epithelial cells type4	2153
CTCGGAGTCCCTGACT-1_CRC2	Epithelial cells type4	2092
CTTACCGGTGTGTGCC-1_CRC2	Epithelial cells type4	2320
CTAGTGAAGATCCGAG-1_CRC2	Epithelial cells type4	2162
GGTGCGTGTGCGAAAC-1_CRC2	Epithelial cells type4	2127
CGAGCCACACGGTAGA-1_CRC2	Epithelial cells type4	3130
TGACTAGTCGGTGTCG-1_CRC2	Epithelial cells type4	2691
GAACCTAAGATCTGCT-1_CRC2	Epithelial cells type4	2588
GGTGAAGTCTTGTTTG-1_CRC2	Epithelial cells type4	2689
CAGTCCTCACGCATCG-1_CRC2	Epithelial cells type4	2354
TTAACTCTCGTAGGTT-1_CRC2	Epithelial cells type4	2506
TGGCTGGTCCTCTAGC-1_CRC2	Epithelial cells type4	3071
CGTAGGCGTGCTAGCC-1_CRC2	Epithelial cells type4	2379

CTGCGGACATTTGCCC-1_CRC2	Epithelial cells type4	3102
GCTGGGTTCTTCAACT-1_CRC2	Epithelial cells type4	3039
CACACAATCACAATGC-1_CRC2	Epithelial cells type4	3041
TACGGATAGGCAAAGA-1_CRC2	Epithelial cells type4	2807
CAAGGCCTCACAGGCC-1_CRC2	Epithelial cells type4	2038
GAGGTGACAGAGCCAA-1_CRC2	Epithelial cells type4	1851
GTACGTAAGAGTACCG-1_CRC2	Epithelial cells type4	2498
ACCAGTAGTTGGTAAA-1_CRC2	Epithelial cells type4	2124
AGCGTCGGTAGGGACT-1_CRC2	Epithelial cells type4	1981
TAGTGGTCATCACCCT-1_CRC2	Epithelial cells type4	1808
AAAGATGGTGCAACGA-1_CRC2	Epithelial cells type4	2182
GCGCAGTAGTGACTCT-1_CRC2	Epithelial cells type4	2075
CAGCCGACATGACGGA-1_CRC2	Epithelial cells type4	1922
CTCGGAGTCGTTACAG-1_CRC2	Epithelial cells type4	2021
AACACGTTCGTACGGC-1_CRC2	Epithelial cells type4	2124
TGTGTTTCAAGCCATT-1_CRC2	Epithelial cells type4	1871
ACTTTCATCAATACCG-1_CRC2	Epithelial cells type4	2213
ATTGGACGTCCATGAT-1_CRC2	Epithelial cells type4	2298
TGCGTGGAGTCGCCGT-1_CRC2	Epithelial cells type4	2627
CTCAGAACAAGAGTCG-1_CRC2	Epithelial cells type4	2947
AACCATGAGGCGATAC-1_CRC2	Epithelial cells type4	2654
TCACGAAAGCGTTCCG-1_CRC2	Epithelial cells type4	2504
ACGCCGAGTCTTCGTC-1_CRC2	Epithelial cells type4	2091
TGGCCAGGTAAATGTG-1_CRC2	Epithelial cells type4	2216
AGTTGGTGTTCGTTGA-1_CRC2	Epithelial cells type4	1915
GACAGAGCAGCTGTAT-1_CRC2	Epithelial cells type4	2051
TCGAGGCAGATATGGT-1_CRC2	Epithelial cells type4	2297
GATCGTAAGTGTCCCG-1_CRC2	Epithelial cells type4	1849
AAGGAGCGTGTGGCTC-1_CRC2	Epithelial cells type4	1872
CTGAAACGTGTGCGTC-1_CRC2	Epithelial cells type4	1444
CTCGAAAGTTTGTTGG-1_CRC2	Epithelial cells type4	2182
GACTGCGCAATGAAAC-1_CRC2	Epithelial cells type4	1977
GAATAAGCAAGAGTCG-1_CRC2	Epithelial cells type4	1606
GTTCGGGTCAGTTGAC-1_CRC2	Epithelial cells type4	1606
AATCCAGGTATGAAAC-1_CRC2	Epithelial cells type4	1899
CAGCGACCACGAGGTA-1_CRC2	Epithelial cells type4	2136
CAACCAAGTGTCGCTG-1_CRC2	Epithelial cells type4	2216
TATCAGGAGCTAACTC-1_CRC2	Epithelial cells type4	2064
AGGCCGTTCCTGTAGA-1_CRC2	Epithelial cells type4	2015
CTCAGAAAGGAACTGC-1_CRC2	Epithelial cells type4	2318
CCTTTCTAGAAACCTA-1_CRC2	Epithelial cells type4	2320
CGGTTAAGTCTTCTCG-1_CRC2	Epithelial cells type4	2229
GTTCGGGGTATAGGTA-1_CRC2	Epithelial cells type4	2119
TACCTATGTTCTGTTT-1_CRC2	Epithelial cells type4	2451
AGCGTCGGTCTACCTC-1_CRC2	Epithelial cells type4	2583
TCGCGAGGTAGCGTAG-1_CRC2	Epithelial cells type4	2601
AGAGTGGAGATGCGAC-1_CRC2	Epithelial cells type4	2484
TCACAAGGTGGTGTAG-1_CRC2	Epithelial cells type4	3057
TTTACTGGTGTTGGGA-1_CRC2	Epithelial cells type4	2199
CAAGAAAGTTGTCGCG-1_CRC2	Epithelial cells type4	2500
GTATTCTTCGGACAAG-1_CRC2	Epithelial cells type4	2396

ACCTTTACAGTCACTA-1_CRC2	Epithelial cells type4	2566
GGCCGATTCCGAAGAG-1_CRC2	Epithelial cells type4	2501
GGTATTGTCAGTACGT-1_CRC2	Epithelial cells type4	2808
TGTGGTAAGTGTCTCA-1_CRC2	Epithelial cells type4	2828
AACCGCGGTCAAGCGA-1_CRC2	Epithelial cells type4	1837
TACGGTAAGCGTAGTG-1_CRC2	Epithelial cells type4	2273
TTGACTTGTAAACACA-1_CRC2	Epithelial cells type4	2573
GTTAAGCCAGCGAACA-1_CRC2	Epithelial cells type4	2410
TGTCCCATCGGCATCG-1_CRC2	Epithelial cells type4	2396
AGAGCGACAAGTCTAC-1_CRC2	Epithelial cells type4	2215
CACAAACCATACAGCT-1_CRC2	Epithelial cells type4	1997
GGCTCGACATGGTTGT-1_CRC2	Epithelial cells type4	2768
TGACTTTTCCACGTTC-1_CRC2	Epithelial cells type4	3220
CGACTTCCAAGTTAAG-1_CRC2	Epithelial cells type4	2363
TACCTTACACAGGAGT-1_CRC2	Epithelial cells type4	2832
AACTCCCGTACCGTTA-1_CRC2	Epithelial cells type4	2500
GTGAAGGTCTTAGCCC-1_CRC2	Epithelial cells type4	2585
CAGAATCTCGAGAACG-1_CRC2	Epithelial cells type4	2283
CGGACACTCCTTGGTC-1_CRC2	Epithelial cells type4	2135
GCCAAATAGATCCCGC-1_CRC2	Epithelial cells type4	2404
GATGCTAAGGTGCTAG-1_CRC2	Epithelial cells type4	2102
ACATCAGCAGTCAGCC-1_CRC2	Epithelial cells type4	2362
GTGCATACATGGGACA-1_CRC2	Epithelial cells type4	2015
AACCATGAGGTGTTAA-1_CRC2	Epithelial cells type4	2177
ACGCAGCAGCCTATGT-1_CRC2	Epithelial cells type4	1803
CCGTACTGTCTGCAAT-1_CRC2	Epithelial cells type4	2427
CAGCGACAGGACATTA-1_CRC2	Epithelial cells type4	2507
CTTAACTCAAACGTGG-1_CRC2	Epithelial cells type4	2022
TATCTCAGTAGAAGGA-1_CRC2	Epithelial cells type4	1991
GGACATTTCATGCATG-1_CRC2	Epithelial cells type4	2413
TTCTACATCGTCCAGG-1_CRC2	Epithelial cells type4	1496
CGAATGTGTCCTAGCG-1_CRC2	Epithelial cells type4	1953
TTTCCTCAGAAACCGC-1_CRC2	Epithelial cells type4	1965
CTAAGACTCTAACTTC-1_CRC2	Epithelial cells type4	2035
CTTAACTGTCCGAGTC-1_CRC2	Epithelial cells type4	2042
GTTACAGAGTTGAGAT-1_CRC2	Epithelial cells type4	2350
TGGTTCCGTACGACCC-1_CRC2	Epithelial cells type4	1902
AGCATACGTCCCTACT-1_CRC2	Epithelial cells type4	2184
CGAATGTAGTTTAGGA-1_CRC2	Epithelial cells type4	2051
CTCTACGAGCGGCTTC-1_CRC2	Epithelial cells type4	1776
GGGACCTCAAGCTGTT-1_CRC2	Epithelial cells type4	2055
GGGCACTAGATCACGG-1_CRC2	Epithelial cells type4	2178
GTAACGTTCTCCAACC-1_CRC2	Epithelial cells type4	2089
GCGACCACACAGGAGT-1_CRC2	Epithelial cells type4	1967
GCTGCAGCAGGTCCAC-1_CRC2	Epithelial cells type4	1852
AAAGATGCATCACGTA-1_CRC2	Epithelial cells type4	1834
GCTGGGTTCGTTTATC-1_CRC2	Epithelial cells type4	2281
GCGACCACACCTTGTC-1_CRC2	Epithelial cells type4	1931
CGCGTTTTCCTTTACA-1_CRC2	Epithelial cells type4	2048
CTAACTTTCCAGAAGG-1_CRC2	Epithelial cells type4	2126
TGAGCCGCAGACAGGT-1_CRC2	Epithelial cells type4	1567

ATCCACCCACGGACAA-1_CRC2	Epithelial cells type4	1626
AGTAGTCTCTGAGTGT-1_CRC2	Epithelial cells type4	2303
CCGTACTGTGGTTTCA-1_CRC2	Epithelial cells type4	2263
CTTGGCTAGGAGTACC-1_CRC2	Epithelial cells type4	1812
TAGACCATCGCATGGC-1_CRC2	Epithelial cells type4	1794
TGCGTGGTCTTCATGT-1_CRC2	Epithelial cells type4	1727
GTTAAGCAGTACGATA-1_CRC2	Epithelial cells type4	1168
TCGTACCCAGGCTGAA-1_CRC2	Epithelial cells type4	796
GTGGGTCCAGGGATTG-1_CRC2	Epithelial cells type4	2777
TGGACGCTCGTCTGAA-1_CRC2	Epithelial cells type4	2581
AGACGTTGTTCGTGAT-1_CRC2	Epithelial cells type4	1781
GTCACGGGTCCGTCAG-1_CRC2	Epithelial cells type4	1856
TACACGATCTACCTGC-1_CRC2	Epithelial cells type4	2331
TAGGCATAGGACACCA-1_CRC2	Epithelial cells type4	2032
CGAATGTAGCCGCCTA-1_CRC2	Epithelial cells type4	2009
CTCCTAGGTCACAAGG-1_CRC2	Epithelial cells type4	2461
CAGCTAATCTCGTTTA-1_CRC2	Epithelial cells type4	1723
AACTGGTAGCCAACAG-1_CRC2	Epithelial cells type4	2495
CACCTTGAGTTACGGG-1_CRC2	Epithelial cells type4	2432
GTCGTAAGTGCAGGTA-1_CRC2	Epithelial cells type4	3058
AAGCCGCTCGAATGGG-1_CRC2	Epithelial cells type4	2042
TCTCTAACACATGGGA-1_CRC2	Epithelial cells type4	2136
GCGCGATCATGTCGAT-1_CRC2	Epithelial cells type4	2112
TACGGTAAGTGTACTC-1_CRC2	Epithelial cells type4	2182
GTGCAGCGTATGAAAC-1_CRC2	Epithelial cells type4	3188
GATGAAAGTCACCTAA-1_CRC2	Epithelial cells type4	2606
AGAGCTTGTCCGAATT-1_CRC2	Epithelial cells type4	2639
TACTCGCGTAGCAAAT-1_CRC2	Epithelial cells type4	2622
AAGGTTCAGGAATCGC-1_CRC2	Epithelial cells type4	2960
ACGATGTAGCGTCAAG-1_CRC2	Epithelial cells type4	2460
CACATTTGTTCGTTGA-1_CRC2	Epithelial cells type4	2007
AACGTTGCATCGATGT-1_CRC2	Epithelial cells type4	2956
TCGCGAGAGACAGAGA-1_CRC2	Epithelial cells type4	2370
GCAGCCACATGACATC-1_CRC2	Epithelial cells type4	3889
GTCTCGTTCAACGGGA-1_CRC2	Epithelial cells type4	4563
GCTTGAATCCTATGTT-1_CRC2	Epithelial cells type4	2759
TTGGAACGTAGCCTAT-1_CRC2	Epithelial cells type4	2801
GGAGCAATCTTAGAGC-1_CRC2	Epithelial cells type4	2208
GACAGAGAGTCGTACT-1_CRC2	Epithelial cells type4	2138
GATCTAGAGCTAGTGG-1_CRC2	Epithelial cells type4	2465
AGCGTCGGTTTGACAC-1_CRC2	Epithelial cells type4	2902
CTCTGGTGTAGGGTAC-1_CRC2	Epithelial cells type4	2559
GACTAACCAATCGAAA-1_CRC2	Epithelial cells type4	2853
CCTAAAGTCAACACCA-1_CRC2	Epithelial cells type4	3500
TGCGGGTCATCACGTA-1_CRC2	Epithelial cells type4	3113
AGACGTTAGAAGAAGC-1_CRC2	Epithelial cells type4	3000
GGGCATCTCATGGTCA-1_CRC2	Epithelial cells type4	2133
TGTATTCAGCTACCGC-1_CRC2	Epithelial cells type4	1899
AGTAGTCAGTCACGCC-1_CRC2	Epithelial cells type4	2403
AACTCAGGTGTGGTTT-1_CRC2	Epithelial cells type4	2512
CCTTCCCCAGCGAACA-1_CRC2	Epithelial cells type4	2317

GCAGTTATCTCAACTT-1_CRC2	Epithelial cells type4	2743
CGATCGGAGTGGGCTA-1_CRC2	Epithelial cells type4	2607
GACTGCGTCTTACCGC-1_CRC2	Epithelial cells type4	3363
ATGCGATAGCAAATCA-1_CRC2	Epithelial cells type4	2092
TGCTGCTAGCTTATCG-1_CRC2	Epithelial cells type4	2025
AAACCTGCAGTTCATG-1_CRC2	Epithelial cells type4	2748
TACACGACACGTCTCT-1_CRC2	Epithelial cells type4	2448
ACGGAGATCTCCCTGA-1_CRC2	Epithelial cells type4	1880
CACATTTGTTGGGACA-1_CRC2	Epithelial cells type4	2289
GAATGAAAGGCACATG-1_CRC2	Epithelial cells type4	2147
CTGTTTAAGTGGAGTC-1_CRC2	Epithelial cells type4	2360
AGCGTCGAGACAGGCT-1_CRC2	Epithelial cells type4	2466
GCGAGAAAGAACTCGG-1_CRC2	Epithelial cells type4	2603
CCTCTGACAACAACCT-1_CRC3	Epithelial cells type4	2190
CGTTGGGCATTTCAGG-1_CRC3	Epithelial cells type4	1380
GTAGGCCAGACTGGGT-1_CRC3	Epithelial cells type4	1866
GGATTACTCTACCAGA-1_CRC3	Epithelial cells type4	860
ACGATGTGTTCAGTAC-1_CRC3	Epithelial cells type4	896
GCATGCGTCTTTAGGG-1_CRC3	Epithelial cells type4	976
AGGGATGGTCTAACGT-1_CRC3	Epithelial cells type4	1253
TTTATGCAGATCACGG-1_CRC3	Epithelial cells type4	1000
GGCTGGTGTACATCCA-1_CRC3	Epithelial cells type4	1636
CTCAGAAAGGGTGTTG-1_CRC3	Epithelial cells type4	938
GCACTCTAGAGGGCTT-1_CRC3	Epithelial cells type4	1083
TGCTACCGTGTGCCTG-1_CRC3	Epithelial cells type4	804
CCTTCCCGTCCAGTAT-1_CRC3	Epithelial cells type4	726
GTTAAGCTCGTACGGC-1_CRC3	Epithelial cells type4	835
TCGTAGACAAACGCGA-1_CRC3	Epithelial cells type4	1419
CACAGTATCGGACAAG-1_CRC3	Epithelial cells type4	699
GTACTCCTCCACTGGG-1_CRC3	Epithelial cells type4	708
CTTACCGCATCTACGA-1_CRC3	Epithelial cells type4	621
GCTCTGTAGATCACGG-1_CRC3	Epithelial cells type4	701
TTCTTAGGTACCGCTG-1_CRC3	Epithelial cells type4	685
GCGGGTTGTGCCTTGG-1_CRC3	Epithelial cells type4	1189
GTCACGGGTCCATGAT-1_CRC3	Epithelial cells type4	830
TGAAAGAGTCGAACAG-1_CRC3	Epithelial cells type4	931
GACCAATGTGAAGGCT-1_CRC3	Epithelial cells type4	960
GGGATGAAGAATCTCC-1_CRC3	Epithelial cells type4	963
CCAGCGAAGTACGCCC-1_CRC3	Epithelial cells type4	1320
CCTCTGAAGAGTAAGG-1_CRC3	Epithelial cells type4	944
AGATTGCTCACGCGGT-1_CRC3	Epithelial cells type4	985
ATGAGGGGTCAATGTC-1_CRC3	Epithelial cells type4	739
ACACCCTGTCGCTTTC-1_CRC3	Epithelial cells type4	815
CTTAACTGTAAGGATT-1_CRC3	Epithelial cells type4	983
TTAGTTCGTAGTAGTA-1_CRC3	Epithelial cells type4	749
TACCTTAGTCTCCATC-1_CRC3	Epithelial cells type4	960
CGGACTGCAGCTGTTA-1_CRC3	Epithelial cells type4	1190
GACACGCTCTGTGCAA-1_CRC1	Epithelial cells type4	968
ATCCACCGTGACGGTA-1_CRC3	Epithelial cells type4	575
CCGGTAGAGAAGGTTT-1_CRC3	Epithelial cells type4	670
CGAACATGTGATGTCT-1_CRC3	Epithelial cells type4	762

GTGCGGTGTAGCTGCC-1_CRC3	Epithelial cells type4	483
CGAGAAGTCCCAAGAT-1_CRC3	Epithelial cells type4	857
ACCCACTGTCACTTCC-1_CRC3	Epithelial cells type4	703
AGGTCCGCAAGGACAC-1_CRC3	Epithelial cells type4	837
CGAGCACAGCACCGCT-1_CRC3	Epithelial cells type4	907
CCCAATCAGGACAGAA-1_CRC3	Epithelial cells type4	856
CAACTAGCAGAAGCAC-1_CRC3	Epithelial cells type4	558
TAGCCGGTCCGCAGTG-1_CRC3	Epithelial cells type4	717
AGAATAGGTATCTGCA-1_CRC3	Epithelial cells type4	2557
CAGCATAGTCGCGTGT-1_CRC3	Epithelial cells type4	2364
CTAAGACAGTCCGGTC-1_CRC3	Epithelial cells type4	1390
GGAATAAAGCTAACTC-1_CRC3	Epithelial cells type4	1753
ATGAGGGAGCCAGGAT-1_CRC3	Epithelial cells type4	1638
TGGCCAGTCCAAACTG-1_CRC3	Epithelial cells type4	1827
CCTAAAGTCCTATTCA-1_CRC3	Epithelial cells type4	1492
TCATTACAGCCCAATT-1_CRC3	Epithelial cells type4	1509
TGACTTTCACGAGGTA-1_CRC3	Epithelial cells type4	1392
AGGTCCGTCAGTGTTG-1_CRC3	Epithelial cells type4	982
GATGAAATCTGCTGTC-1_CRC3	Epithelial cells type4	989
ACCCACTGTCCCTTGT-1_CRC3	Epithelial cells type4	939
TGCTACCAGATGCCAG-1_CRC3	Epithelial cells type4	1492
ACTTTCAGTCACACGC-1_CRC3	Epithelial cells type4	1141
TGAGCATGTCCGAACC-1_CRC3	Epithelial cells type4	1034
AAGTCTGGTCTCCACT-1_CRC3	Epithelial cells type4	1346
CTTGGCTTCCGGGTGT-1_CRC3	Epithelial cells type4	1239
GCGCGATAGTTCGCGC-1_CRC3	Epithelial cells type4	1414
TCTGAGACATTAGCCA-1_CRC3	Epithelial cells type4	1554
ATGTGTGGTGGCGAAT-1_CRC3	Epithelial cells type4	2061
CTCTAATTCGTGTAGT-1_CRC3	Epithelial cells type4	1538
ATTCTACGTTAAGAAC-1_CRC3	Epithelial cells type4	1973
AGCCTAAAGGCATTGG-1_CRC3	Epithelial cells type4	1913
CTCCTAGAGAATAGGG-1_CRC3	Epithelial cells type4	1785
GCTGCAGTCGTACCGG-1_CRC2	Epithelial cells type4	1263
TCAACGATCTCCCTGA-1_CRC2	Epithelial cells type4	2147
GAAACTCAGCAAATCA-1_CRC2	Epithelial cells type4	934
TCGCGTTCAATAGAGT-1_CRC2	Epithelial cells type4	286
CCAATCCTCGTAGGAG-1_CRC2	Epithelial cells type4	943
TACGGTATCAGGCCCA-1_CRC2	Epithelial cells type4	510
GAATAAGTCTCTAAGG-1_CRC2	Epithelial cells type4	1008
GTACGTACAGTCGTGC-1_CRC2	Epithelial cells type4	829
AGCTTGACATACGCTA-1_CRC2	Epithelial cells type4	1187
GTAGTCAAGTTCGATC-1_CRC2	Epithelial cells type4	1229
CACAGTAGTACTCTCC-1_CRC2	Epithelial cells type4	1452
ACTTACTTCTGTGCAA-1_CRC2	Epithelial cells type4	1599
GCAATCACACCCTATC-1_CRC2	Epithelial cells type4	1796
GCGCAACTCGGACAAG-1_CRC2	Epithelial cells type4	1974
CGGAGCTCATTGCGGC-1_CRC2	Epithelial cells type4	1390
GGAAAGCAGGCAGTCA-1_CRC2	Epithelial cells type4	1665
AGTCTTTCATTACGAC-1_CRC2	Epithelial cells type4	2114
ATGGGAGTCCGAAGAG-1_CRC2	Epithelial cells type4	1269
CCCTCCTAGATCACGG-1_CRC2	Epithelial cells type4	2195

ACATCAGTCCATTCTA-1_CRC2	Epithelial cells type4	1984
TAGAGCTTCACTCTTA-1_CRC2	Epithelial cells type4	2090
AACTCCCCATGACATC-1_CRC2	Epithelial cells type4	2082
CACATAGCATTACGAC-1_CRC2	Epithelial cells type4	1724
ACCAGTACACGGTAAG-1_CRC2	Epithelial cells type4	1108
ACAGCCGCACTCAGGC-1_CRC1	Epithelial cells type4	1107
GTCACGGTCGCGGATC-1_CRC2	Epithelial cells type4	1010
TGCTGCTGTCCAGTGC-1_CRC2	Epithelial cells type4	647
CCACTACCAATAAGCA-1_CRC3	Epithelial cells type4	1687
TAGAGCTGTAAACGCG-1_CRC2	Epithelial cells type4	1690
TCGTACCTCCGCAAGC-1_CRC2	Epithelial cells type4	1074
GAACCTAAGCTACCGC-1_CRC3	Epithelial cells type4	1617
TTAGGACCATCGGACC-1_CRC3	Epithelial cells type4	1559
ACTATCTGTTTGTGTG-1_CRC3	Epithelial cells type4	1158
ATTTCTGCAATTCCTT-1_CRC3	Epithelial cells type4	1301
CCTTTCTTCCGCAGTG-1_CRC3	Epithelial cells type4	2259
ATCCGAAAGGCTATCT-1_CRC2	Epithelial cells type4	756
ACTGTCCGTCTGCAAT-1_CRC2	Epithelial cells type4	686
CCATTCGCACAAGACG-1_CRC2	Epithelial cells type4	978
ACGGCCACATTCACTT-1_CRC3	Epithelial cells type4	1616
GAACGGACAATAACGA-1_CRC3	Epithelial cells type4	1307
GCATGTATCGGATGTT-1_CRC0	Epithelial cells type4	2731
TACACGAAGGACAGCT-1_CRC0	Epithelial cells type4	2011
TACAGTGCATATACGC-1_CRC0	Epithelial cells type4	2285
CAGGTGCCAAGAAGAG-1_CRC0	Epithelial cells type4	1107
ATGGGAGGTCTGCGGT-1_CRC0	Epithelial cells type4	821
CGCCAAGTCCCTCTTT-1_CRC0	Epithelial cells type4	780
GGAAAGCTCGCTAGCG-1_CRC0	Epithelial cells type4	1211
CCTTTCTAGGAACTGC-1_CRC1	Epithelial cells type4	1398
CGTTGGGAGACAGACC-1_CRC1	Epithelial cells type4	787
GGAAAGCGTCATGCAT-1_CRC1	Epithelial cells type4	780
CTTTGCGTCCAGAAGG-1_CRC1	Epithelial cells type4	1465
TTCTCAATCATGTCCC-1_CRC1	Epithelial cells type4	1092
CAGAATCCAGACTCGC-1_CRC1	Epithelial cells type5	1402
TCAGCAAAGGAATTAC-1_CRC1	Epithelial cells type5	1405
GTAGTCAAGGAGCGAG-1_CRC1	Epithelial cells type5	1124
GGACGTCGTAAACCTC-1_CRC1	Epithelial cells type5	516
AGCTCTCGTTCTCATT-1_CRC1	Epithelial cells type5	946
GCTCCTAAGGAGTAGA-1_CRC1	Epithelial cells type5	1411
ACTGAACCAAAGAATC-1_CRC1	Epithelial cells type5	1323
ACACCGGAGCGACGTA-1_CRC1	Epithelial cells type5	1169
CTCTGGTGTGCGAAAC-1_CRC1	Epithelial cells type5	1048
ATTGGACCAGATCGGA-1_CRC1	Epithelial cells type5	1146
CAGCATAGTCCAACTA-1_CRC1	Epithelial cells type5	1229
GCAAACTTCCCAGGTG-1_CRC1	Epithelial cells type5	839
GGTGCGTGTGGGTCAA-1_CRC1	Epithelial cells type5	1359
CGAGAAGAGGAGTCTG-1_CRC1	Epithelial cells type5	1528
GAACCTATCTCGCTTG-1_CRC1	Epithelial cells type5	1270
ATGAGGGGTGTCAATC-1_CRC1	Epithelial cells type5	1424
CCACGGATCCAAGCCG-1_CRC1	Epithelial cells type5	1177
CGGAGTCAGTAAGTAC-1_CRC1	Epithelial cells type5	1092

AGCATACGTATAGTAG-1_CRC1	Epithelial cells type5	1372
TCAGCAAAGAATGTTG-1_CRC1	Epithelial cells type5	1022
TTGAACGTCAGCTTAG-1_CRC1	Epithelial cells type5	1162
CAAGATCGTTGTCGCG-1_CRC1	Epithelial cells type5	5800
GTCGGGTTCATGGTCA-1_CRC2	Epithelial cells type5	1625
GATCAGTAGGCATGTG-1_CRC1	Epithelial cells type5	1247
AGGCCGTCACTTCGAA-1_CRC1	Epithelial cells type5	891
GCCTCTACACCTCGTT-1_CRC1	Epithelial cells type5	1020
ACATGGTAGTGTGAAT-1_CRC1	Epithelial cells type5	872
CAGCTAACAGCTGTGC-1_CRC1	Epithelial cells type5	1121
CCTATTAGTTAGAACA-1_CRC2	Epithelial cells type5	1611
ACTGATGCACAGGTTT-1_CRC2	Epithelial cells type5	1187
AGCGTCGTCTTTAGTC-1_CRC2	Epithelial cells type5	683
TGAGCATCATCACAAC-1_CRC2	Epithelial cells type5	603
CCTTCGATCATTATCC-1_CRC2	Epithelial cells type5	420
GTGTTAGTCTTGTCAT-1_CRC2	Epithelial cells type5	375
GCGCCAAAGGAGCGTT-1_CRC2	Epithelial cells type5	510
TCAGGATAGGTGCAAC-1_CRC2	Epithelial cells type5	410
CATGGCGGTCGAATCT-1_CRC2	Epithelial cells type5	707
TGCGGGTTCTCATTCA-1_CRC2	Epithelial cells type5	197
AGCGTATCAGCTGCAC-1_CRC2	Epithelial cells type5	247
TCGGTAAGTACATGTC-1_CRC2	Epithelial cells type5	288
CACAGGCAGGTGGGTT-1_CRC2	Epithelial cells type5	1244
CGTGTAAAGGCCCTTG-1_CRC2	Epithelial cells type5	989
ACGATGTGTTCGGCAC-1_CRC2	Epithelial cells type5	904
CTACACCCAGTTTACG-1_CRC2	Epithelial cells type5	968
TCACAAGAGGAATCGC-1_CRC2	Epithelial cells type5	1085
CATTATCAGCGTAGTG-1_CRC2	Epithelial cells type5	1448
GATCAGTAGAGTACAT-1_CRC2	Epithelial cells type5	1091
CAGCCGAAGTCAATAG-1_CRC3	Epithelial cells type5	933
ATTGGTGCAGGTTTCA-1_CRC2	Epithelial cells type5	593
TTTATGCAGCTGTCTA-1_CRC2	Epithelial cells type5	820
CTAACTTCAATAGAGT-1_CRC2	Epithelial cells type5	520
GACCAATGTATTCGTG-1_CRC2	Epithelial cells type5	469
GTGTTAGCATCCGCGA-1_CRC2	Epithelial cells type5	970
TACTCGCCACATGACT-1_CRC2	Epithelial cells type5	689
ATGTGTGCATAAAGGT-1_CRC3	Epithelial cells type5	881
GCCAAATAGCCTATGT-1_CRC2	Epithelial cells type5	1399
AGCATACGTACCCAAT-1_CRC2	Epithelial cells type5	623
CAGCAGCAGCGAAGGG-1_CRC2	Epithelial cells type5	979
GCGCAGTCAGCTGTGC-1_CRC2	Epithelial cells type5	1223
TCATTACAGAGTTGGC-1_CRC2	Epithelial cells type5	866
CTCATTAGTACGCACC-1_CRC1	Epithelial cells type5	1986
CGTTGGGAGATGCCAG-1_CRC1	Epithelial cells type5	1168
CTCGAGGAGCCCTAAT-1_CRC1	Epithelial cells type5	1104
TTTACTGAGCTACCGC-1_CRC1	Epithelial cells type5	1285
CTAAGACTCCAAATGC-1_CRC1	Epithelial cells type5	1211
GTACGTAAGACACTAA-1_CRC1	Epithelial cells type5	856
GTAGTCAAGATGCGAC-1_CRC1	Epithelial cells type5	1487
GGACGTCGTCTAGCCG-1_CRC1	Epithelial cells type5	1609
GGTGTTAGTCATTAGC-1_CRC1	Epithelial cells type5	1490

GAGGTGATCTAACGGT-1_CRC1	Epithelial cells type5	1101
TCATTACCATGTCTCC-1_CRC1	Epithelial cells type5	1373
AGTGAGGCATGAAGTA-1_CRC1	Epithelial cells type5	1303
GTTTCTAGTTCCCTTG-1_CRC1	Epithelial cells type5	1104
TCGCGAGGTAAACGCG-1_CRC1	Epithelial cells type5	1742
TCACGAAGTCTTCGTC-1_CRC1	Epithelial cells type5	1073
TTAGGCAAGTGGAGTC-1_CRC1	Epithelial cells type5	831
GAGGTGACACACCGCA-1_CRC1	Epithelial cells type5	871
AGTGAGGAGGGTGTGT-1_CRC1	Epithelial cells type5	1316
ATTTCTGAGTGCCAGA-1_CRC1	Epithelial cells type5	914
CTTACCGTCAGCAACT-1_CRC1	Epithelial cells type5	1015
ATTGGTGCATATGCTG-1_CRC1	Epithelial cells type5	1024
GAGCAGATCTTGTATC-1_CRC1	Epithelial cells type5	757
CAGCGACTCGTTACAG-1_CRC1	Epithelial cells type5	1183
TCATTACGTACGACCC-1_CRC1	Epithelial cells type5	1232
GTATCTTTCTAAGCCA-1_CRC1	Epithelial cells type5	1054
CTAACTTCAAGCGAGT-1_CRC1	Epithelial cells type5	1064
AAGGTTCCACAGGAGT-1_CRC1	Epithelial cells type5	1029
CGATGGCAGTTCCACA-1_CRC1	Epithelial cells type5	903
AGGCCGTGTAACGCGA-1_CRC1	Epithelial cells type5	758
CCCTCCTGTGATGATA-1_CRC1	Epithelial cells type5	916
TGTTCCGCAATCCAAC-1_CRC1	Epithelial cells type5	995
ACGGCCACATAACCTG-1_CRC1	Epithelial cells type5	1126
TCGCGAGTCGCTAGCG-1_CRC1	Epithelial cells type5	962
AGGTCCGCAAATCCGT-1_CRC1	Epithelial cells type5	659
ATTGGACAGTATGACA-1_CRC1	Epithelial cells type5	1162
CCTACCAGTCCGTGAC-1_CRC1	Epithelial cells type5	1017
AGGGTGAGTACCATCA-1_CRC1	Epithelial cells type5	994
CGTAGGCGTCTCTCTG-1_CRC1	Epithelial cells type5	945
CTACATTAGAGACGAA-1_CRC1	Epithelial cells type5	999
GCAGCCACAGTATGCT-1_CRC1	Epithelial cells type5	1657
GCCTCTAGTGCGCTTG-1_CRC1	Epithelial cells type5	1019
GGAATAATCTGCTGCT-1_CRC1	Epithelial cells type5	1318
CCATTCGCACGCATCG-1_CRC1	Epithelial cells type5	1283
TGAGCCGTCAGTTGAC-1_CRC1	Epithelial cells type5	1183
TTCTCCTGTTTGGCGC-1_CRC1	Epithelial cells type5	1457
TACCTTAAGATGGCGT-1_CRC1	Epithelial cells type5	1316
CGGACTGCAAACTGTC-1_CRC1	Epithelial cells type5	1273
TACCTTAAGAGCAATT-1_CRC1	Epithelial cells type5	1111
ACTGATGGTCGGCACT-1_CRC1	Epithelial cells type5	1118
CGCGGTAAGGTGTGGT-1_CRC1	Epithelial cells type5	1279
CGTTGGGTCTGCCCTA-1_CRC1	Epithelial cells type5	1288
TGCACCTGTCATATCG-1_CRC1	Epithelial cells type5	1323
TTCTACACAGCGATCC-1_CRC1	Epithelial cells type5	1198
CAACTAGAGAGCAATT-1_CRC1	Epithelial cells type5	1168
AACTGGTAGGATGGTC-1_CRC1	Epithelial cells type5	1078
GCGCAGTTCCCAGGTG-1_CRC1	Epithelial cells type5	1112
TCGTAGAGTCGGCATC-1_CRC1	Epithelial cells type5	1382
CTTTGCGGTGATGATA-1_CRC1	Epithelial cells type5	981
TGACTAGGTTCAACCA-1_CRC1	Epithelial cells type5	1424
GACTGCGAGTGTTGAA-1_CRC1	Epithelial cells type5	958

GATTCAGGTGCAACGA-1_CRC1	Epithelial cells type5	837
AGCATACTCGTCCGTT-1_CRC1	Epithelial cells type5	1241
TACGGTAAGGAGTCTG-1_CRC1	Epithelial cells type5	1388
CTCACACTCAACACCA-1_CRC1	Epithelial cells type5	1471
CTTAACTGTCATATCG-1_CRC1	Epithelial cells type5	1635
TTTATGCGTACAGACG-1_CRC1	Epithelial cells type5	1256
GAGCAGACAGTGACAG-1_CRC1	Epithelial cells type5	1269
ACATACGAGTAGTGCG-1_CRC1	Epithelial cells type5	1333
AGCATACAGACACGAC-1_CRC1	Epithelial cells type5	1394
GCACATACACCATCCT-1_CRC1	Epithelial cells type5	1285
TAAGTGCTCTTGTACT-1_CRC1	Epithelial cells type5	2098
CGAACATAGGCTACGA-1_CRC1	Epithelial cells type5	1576
GGTGTTACATATACCG-1_CRC1	Epithelial cells type5	1780
GTACTCCGTCCAGTGC-1_CRC1	Epithelial cells type5	2236
TAAACCGTCAAGATCC-1_CRC1	Epithelial cells type5	1423
GACCTGGCATTTGCCC-1_CRC1	Epithelial cells type5	1524
GCTGCTTCATTGGGCC-1_CRC1	Epithelial cells type5	1121
AACTCTTAGCTAGTGG-1_CRC1	Epithelial cells type5	1456
GAACCTACATTAGGCT-1_CRC1	Epithelial cells type5	1285
GCGCAACAGATTACCC-1_CRC1	Epithelial cells type5	1026
CGTGTCTCACCACCAG-1_CRC1	Epithelial cells type5	1212
GATGAGGAGTCATCCA-1_CRC1	Epithelial cells type5	1345
GTACTTTAGTTAGCGG-1_CRC1	Epithelial cells type5	1336
TGCGGGTAGGTGATAT-1_CRC1	Epithelial cells type5	1103
TTGGCAATCCCGGATG-1_CRC1	Epithelial cells type5	992
ACTGAGTCATGCAACT-1_CRC1	Epithelial cells type5	1051
CGACTTCAGTACACCT-1_CRC1	Epithelial cells type5	1142
ACGGGTCGTAAGAGAG-1_CRC1	Epithelial cells type5	1030
TCTCATAGTGATGTGG-1_CRC1	Epithelial cells type5	1267
AGTCTTTTCCTGCCAT-1_CRC1	Epithelial cells type5	1095
GTGAAGGGTGAGGGTT-1_CRC1	Epithelial cells type5	868
CTGTTTAGTTGGTTTG-1_CRC1	Epithelial cells type5	1070
CGGACTGTCACTATTC-1_CRC1	Epithelial cells type5	830
CGGCTAGTCTTCAACT-1_CRC1	Epithelial cells type5	1267
AACCGCGAGCCGCCTA-1_CRC1	Epithelial cells type5	996
TCGAGGCAGCTTATCG-1_CRC1	Epithelial cells type5	940
ATTACTCTCAGGCCCA-1_CRC1	Epithelial cells type5	1438
CTAATGGCACCCATTC-1_CRC1	Epithelial cells type5	1247
TTTCCTCCAGAGTGTG-1_CRC1	Epithelial cells type5	1392
CGGAGTCGTAAGGATT-1_CRC1	Epithelial cells type5	1167
TAGCCGGAGCAGATCG-1_CRC1	Epithelial cells type5	1333
GAGGTGATCTAACTTC-1_CRC1	Epithelial cells type5	1130
CGGTTAATCAGGATCT-1_CRC1	Epithelial cells type5	1393
GGGAATGCAGACTCGC-1_CRC1	Epithelial cells type5	1815
TAAGCGTAGAGCAATT-1_CRC1	Epithelial cells type5	1276
AAAGTAGGTGACTACT-1_CRC1	Epithelial cells type5	1533
GTATCTTAGACTAAGT-1_CRC1	Epithelial cells type5	1591
TCGTACCTCACAATGC-1_CRC1	Epithelial cells type5	1208
CAAGGCCTCTCGATGA-1_CRC1	Epithelial cells type5	1696
TGGACGCTCAGCGATT-1_CRC1	Epithelial cells type5	1323
TTGTAGGGTTGAGGTG-1_CRC1	Epithelial cells type5	1629

TCAGGTAAGTGAAGAG-1_CRC1	Epithelial cells type5	1096
TGCCCATAGACTCGGA-1_CRC1	Epithelial cells type5	1311
GCACTCTAGACTAGAT-1_CRC1	Epithelial cells type5	1755
TTTACTGTCAGTCAGT-1_CRC1	Epithelial cells type5	1536
CTGCTGTAGAGCTGGT-1_CRC1	Epithelial cells type5	1524
CTAGAGTTCACCATAG-1_CRC1	Epithelial cells type5	1263
CAAGGCCAGGTGTGGT-1_CRC1	Epithelial cells type5	1196
CATCAGAAGACTGGGT-1_CRC1	Epithelial cells type5	1373
ATCGAGTCACCTCGTT-1_CRC1	Epithelial cells type5	1705
CCTCAGTGTTCGTTGA-1_CRC1	Epithelial cells type5	1421
TCGCGTTCACACGCTG-1_CRC1	Epithelial cells type5	1625
TGAGAGGAGTACGATA-1_CRC1	Epithelial cells type5	1456
CACAAACTCCTATTCA-1_CRC1	Epithelial cells type5	1809
CCCAATCGTCAAGCGA-1_CRC1	Epithelial cells type5	1504
GTCTTCGCACTATCTT-1_CRC1	Epithelial cells type5	1499
CGATGGCCAGCATGAG-1_CRC1	Epithelial cells type5	1504
GATCGCGAGTATCTCG-1_CRC1	Epithelial cells type5	1097
AAGCCGCCATCACGAT-1_CRC1	Epithelial cells type5	1183
CTCGTACCATTGGGCC-1_CRC1	Epithelial cells type5	1430
CGTCACTCACCAACCG-1_CRC1	Epithelial cells type5	1312
CGAGCACGTCTGCGGT-1_CRC1	Epithelial cells type5	1096
CGCTTCATCTTGTATC-1_CRC1	Epithelial cells type5	1098
CAGATCATCGGAAATA-1_CRC1	Epithelial cells type5	1303
TCAACGAAGTTTGCGT-1_CRC1	Epithelial cells type5	1367
AGGGTGAGTCTTCAAG-1_CRC1	Epithelial cells type5	1594
GAAACTCAGCCAACAG-1_CRC1	Epithelial cells type5	958
AGCTCCTAGTACGTTC-1_CRC1	Epithelial cells type5	942
GCTTGAAAGGAGTAGA-1_CRC1	Epithelial cells type5	1192
ACTGTCCAGTTCGATC-1_CRC1	Epithelial cells type5	1437
CTCGGAGGTCTAGTGT-1_CRC1	Epithelial cells type5	967
CTGAAACTCCGAGCCA-1_CRC1	Epithelial cells type5	1237
GGATTACAGCCTTGAT-1_CRC1	Epithelial cells type5	1030
GGCCGATTCAAGATCC-1_CRC1	Epithelial cells type5	1147
TGAGGGAGTTGCCTCT-1_CRC1	Epithelial cells type5	809
CCTTTCTCATGAACCT-1_CRC1	Epithelial cells type5	1230
GCCAAATTCGACCAGC-1_CRC1	Epithelial cells type5	877
TATCAGGCAAACAACA-1_CRC1	Epithelial cells type5	1090
CTGCGGACAATGGATA-1_CRC1	Epithelial cells type5	1025
CTCGAGGAGTAACCCT-1_CRC1	Epithelial cells type5	875
GGAATAAAGGGCTTCC-1_CRC1	Epithelial cells type5	936
AGAGCGAAGCACGCCT-1_CRC1	Epithelial cells type5	1123
CGGTTAATCGTGGGAA-1_CRC1	Epithelial cells type5	1010
CAGCATAGTCGCATCG-1_CRC1	Epithelial cells type5	825
ACGATGTCACGAGAGT-1_CRC1	Epithelial cells type5	997
CAAGAAATCCCGGATG-1_CRC1	Epithelial cells type5	922
CTAACTTCAAGAGTCG-1_CRC1	Epithelial cells type5	1244
ATAAGAGTCGTCCAGG-1_CRC1	Epithelial cells type5	1124
ATTACTCGTTCAGGCC-1_CRC1	Epithelial cells type5	918
CGTCCATCAAGTAATG-1_CRC1	Epithelial cells type5	1172
CGAGAAGTCCGCATCT-1_CRC1	Epithelial cells type5	936
GAACCTAGTGGTCCGT-1_CRC1	Epithelial cells type5	1040

GGAGCAAAGGGATGGG-1_CRC1	Epithelial cells type5	1168
ACGGGCTGTACGAAAT-1_CRC1	Epithelial cells type5	747
CGCTATCGTGCAGTAG-1_CRC1	Epithelial cells type5	955
GCATGCGGTGCATCTA-1_CRC1	Epithelial cells type5	1504
GGGTTGCAGTGGTAAT-1_CRC1	Epithelial cells type5	1198
AGCTCTCAGGACAGAA-1_CRC1	Epithelial cells type5	1971
AGTTGGTAGAGTGAGA-1_CRC1	Epithelial cells type5	1269
TGTGTTTCAAGCCTAT-1_CRC1	Epithelial cells type5	1480
CGGAGCTCAGTAAGAT-1_CRC1	Epithelial cells type5	1697
TGTCCCAGTCTAGTCA-1_CRC1	Epithelial cells type5	1509
GTTCATTCACAAGTAA-1_CRC1	Epithelial cells type5	1154
CTCGAGGAGTGCCATT-1_CRC1	Epithelial cells type5	1339
GTGTGCGTCAGCAACT-1_CRC1	Epithelial cells type5	1523
AGGGATGCAGGATCGA-1_CRC1	Epithelial cells type5	1165
TCGCGAGCATGCCCGA-1_CRC1	Epithelial cells type5	1392
GTACTCCGTCTTCGTC-1_CRC1	Epithelial cells type5	873
CCTACACCACAGACAG-1_CRC1	Epithelial cells type5	984
TTAGGCACACATTAGC-1_CRC1	Epithelial cells type5	1248
TACCTTAAGCAGGTCA-1_CRC1	Epithelial cells type5	991
TACTCGCTCGGAGCAA-1_CRC1	Epithelial cells type5	867
TATGCCCAGTTAGGTA-1_CRC1	Epithelial cells type5	906
CTCCTAGGTGCCTGTG-1_CRC1	Epithelial cells type5	1259
AACTCCCCAGGACCCT-1_CRC1	Epithelial cells type5	1016
GTTCGGGAGGATGTAT-1_CRC1	Epithelial cells type5	986
TTGCGTCGTATATGAG-1_CRC1	Epithelial cells type5	1718
GCTCCTAAGGATTCGG-1_CRC1	Epithelial cells type5	840
CGATCGGTCATTCACT-1_CRC1	Epithelial cells type5	927
TATGCCCCACACGCTG-1_CRC1	Epithelial cells type5	872
AACTCAGCACGTCTCT-1_CRC1	Epithelial cells type5	1176
CACCAGGAGACTAAGT-1_CRC1	Epithelial cells type5	1015
CGGAGCTTCGAATGGG-1_CRC1	Epithelial cells type5	1006
ACGGCCACATCACCCT-1_CRC1	Epithelial cells type5	773
TGCGGGTGTATATGAG-1_CRC1	Epithelial cells type5	1032
ACTGTCCGTAGAGTGC-1_CRC1	Epithelial cells type5	1221
AGGCCACCACCACCAG-1_CRC1	Epithelial cells type5	1227
CAGCTGGGTGGTCCGT-1_CRC1	Epithelial cells type5	1358
ACAGCTACAATGTAAG-1_CRC1	Epithelial cells type5	1170
TCTGAGACACGGTAGA-1_CRC1	Epithelial cells type5	1008
GATCGTACAAGCGATG-1_CRC1	Epithelial cells type5	1602
AACACGTCAGACGCTC-1_CRC1	Epithelial cells type5	1419
CGCTGGAAGACTAGAT-1_CRC1	Epithelial cells type5	1289
AGGGATGGTTACAGAA-1_CRC1	Epithelial cells type5	1408
GCGCAGTGTAACGCGA-1_CRC1	Epithelial cells type5	1477
GATGCTAAGATCCCAT-1_CRC1	Epithelial cells type5	1020
ATCACGACAGCTGTGC-1_CRC1	Epithelial cells type5	1039
TGGGAAGCAGTTTACG-1_CRC1	Epithelial cells type5	1098
CAAGGCCAGAGCCTAG-1_CRC1	Epithelial cells type5	1259
GGCGACTTCCTCAATT-1_CRC1	Epithelial cells type5	1168
ATCATCTCAGTTTACG-1_CRC1	Epithelial cells type5	1425
TAAGTGCAGTTAACGA-1_CRC1	Epithelial cells type5	2252
TTGCCGTCACCAGTTA-1_CRC1	Epithelial cells type5	1903

CATCAGATCAGTACGT-1_CRC1	Epithelial cells type5	1597
GGACAGATCCACGTGG-1_CRC1	Epithelial cells type5	1778
CCTTCGATCTGCCCTA-1_CRC1	Epithelial cells type5	1670
GCTGCAGGTGTAAGTA-1_CRC1	Epithelial cells type5	1700
CGTTGGGCAAGTCTGT-1_CRC1	Epithelial cells type5	1283
TTTCCTCTCATGGTCA-1_CRC1	Epithelial cells type5	1423
ACGTCAATCCTAGAAC-1_CRC1	Epithelial cells type5	1031
TTAGGACAGTTATCGC-1_CRC1	Epithelial cells type5	704
ATCTACTTCAACCATG-1_CRC1	Epithelial cells type5	1135
CGTTCTGCATTACGAC-1_CRC1	Epithelial cells type5	865
ATCACGACAAGTACCT-1_CRC1	Epithelial cells type5	802
GTTACAGCACAGACAG-1_CRC1	Epithelial cells type5	1140
AGTTGGTCACCAGCAC-1_CRC1	Epithelial cells type5	1226
ACTTACTAGAATTCCC-1_CRC1	Epithelial cells type5	1047
TCATTACGTACAGTGG-1_CRC1	Epithelial cells type5	911
CACATAGCAGGCAGTA-1_CRC1	Epithelial cells type5	1482
CCTTCGATCTCGTTTA-1_CRC1	Epithelial cells type5	1473
CAGCATATCTTTACAC-1_CRC1	Epithelial cells type5	763
AACTGGTAGTGCGATG-1_CRC1	Epithelial cells type5	791
TAAGCGTTCCGAATGT-1_CRC1	Epithelial cells type5	661
AGGTCATCAATGAATG-1_CRC1	Epithelial cells type5	980
CATCAGAAGTCATCCA-1_CRC1	Epithelial cells type5	982
TCGAGGCCAGACGCAA-1_CRC1	Epithelial cells type5	843
ACTTTCAGTCTGGAGA-1_CRC1	Epithelial cells type5	1091
ACGGGTCGTGTTTGGT-1_CRC1	Epithelial cells type5	1123
GTACGTATCCCTCAGT-1_CRC1	Epithelial cells type5	1003
GTCACGGGTATTACCG-1_CRC1	Epithelial cells type5	1409
TAGAGCTGTGTTAAGA-1_CRC1	Epithelial cells type5	1107
GAAATGAGTGAGCGAT-1_CRC1	Epithelial cells type5	976
TTTACTGTCTTGCCGT-1_CRC1	Epithelial cells type5	1219
AAGACCTTCTAACTGG-1_CRC1	Epithelial cells type5	814
TGCGGGTTCAACGCTA-1_CRC1	Epithelial cells type5	574
CGGACTGAGCTACCGC-1_CRC1	Epithelial cells type5	818
GTGCATAGTCATGCAT-1_CRC1	Epithelial cells type5	1185
AGTTGGTTCCAAGCCG-1_CRC1	Epithelial cells type5	911
CGGCTAGTCCACGAAT-1_CRC1	Epithelial cells type5	576
CTTAGGAAGAAACCGC-1_CRC1	Epithelial cells type5	1267
TTCTTAGAGAACTCGG-1_CRC1	Epithelial cells type5	966
CACCTTGCAATGTTGC-1_CRC1	Epithelial cells type5	1393
CGTCAGGAGTCCGGTC-1_CRC1	Epithelial cells type5	1260
ACAGCTACAAAGGCGT-1_CRC1	Epithelial cells type5	1058
AGATTGCAGACACGAC-1_CRC1	Epithelial cells type5	1081
CTCGGGAGTTTAGGAA-1_CRC1	Epithelial cells type5	1240
AAGACCTGTCGGCACT-1_CRC1	Epithelial cells type5	950
ACTGAGTGTACACCGC-1_CRC1	Epithelial cells type5	809
GCGACCAAGATATGCA-1_CRC1	Epithelial cells type5	743
CGAATGTCACGTAAGG-1_CRC1	Epithelial cells type5	1102
CGAGAAGAGACAGACC-1_CRC1	Epithelial cells type5	1144
CTGCGGAGTACACCGC-1_CRC1	Epithelial cells type5	1148
GCAGCCATCTACCAGA-1_CRC1	Epithelial cells type5	1146
CAGCCGAAGTTCCACA-1_CRC1	Epithelial cells type5	1233

TGAGCATCATATGGTC-1_CRC1	Epithelial cells type5	1134
ATCATCTGTACAGTGG-1_CRC1	Epithelial cells type5	783
ACAGCCGTCTTACCGC-1_CRC1	Epithelial cells type5	704
TCGGGACTCTCTGCTG-1_CRC1	Epithelial cells type5	631
CCGTACTCAAGCCCAC-1_CRC1	Epithelial cells type5	1164
AACTCTTCACCGATAT-1_CRC1	Epithelial cells type5	957
GAACGGATCCCTTGCA-1_CRC1	Epithelial cells type5	999
TTCTTAGGTAACGACG-1_CRC1	Epithelial cells type5	800
GTAACTGTCCACGTGG-1_CRC1	Epithelial cells type5	1078
TATCTCATCTGCGACG-1_CRC1	Epithelial cells type5	866
ACACCAAAGAGCCTAG-1_CRC1	Epithelial cells type5	617
GCACTCTAGGTTACCT-1_CRC1	Epithelial cells type5	758
TGCGTGGAGAGTGAGA-1_CRC1	Epithelial cells type5	2115
CAGAATCTCTTAGAGC-1_CRC1	Epithelial cells type5	1607
AGGCCGTCATTATCTC-1_CRC1	Epithelial cells type5	1094
AAGGCAGAGCGATCCC-1_CRC1	Epithelial cells type5	1222
CGTGTAAGTAGGGACT-1_CRC1	Epithelial cells type5	1141
TGGCGCAGTTAAGGGC-1_CRC1	Epithelial cells type5	867
GGCTCGACACCTATCC-1_CRC1	Epithelial cells type5	1164
TGCCAAACATAAGACA-1_CRC1	Epithelial cells type5	1529
ACGTCAATCTGCTGCT-1_CRC1	Epithelial cells type5	1628
ATAACGCAGCTGCGAA-1_CRC1	Epithelial cells type5	1442
GAGCAGATCTGTCTAT-1_CRC1	Epithelial cells type5	1066
GGTGAAGAGTGACTCT-1_CRC1	Epithelial cells type5	1161
CAGAGAGTCCTCATTA-1_CRC0	Epithelial cells type5	1727
CATTATCCAACGCACC-1_CRC1	Epithelial cells type5	1823
CCTAAAGCAGTCTTCC-1_CRC2	Epithelial cells type5	3745
AACTCTTAGGGTATCG-1_CRC2	Epithelial cells type5	1661
ACGAGGATCGGAATCT-1_CRC2	Epithelial cells type5	2129
AGTAGTCCATGCTAGT-1_CRC1	Epithelial cells type5	1443
CTACCCACACATAACC-1_CRC1	Epithelial cells type5	961
TTTGCGCAGAAGGACA-1_CRC1	Epithelial cells type5	1442
CCTTCGACAGGGTTAG-1_CRC1	Epithelial cells type5	1014
CGATGTAGTCCGCTGA-1_CRC1	Epithelial cells type5	970
GCAAACTTCATCGCTC-1_CRC1	Epithelial cells type5	1122
TTTATGCAGTGTTAGA-1_CRC1	Epithelial cells type5	1177
AAGTCTGGTACAGACG-1_CRC1	Epithelial cells type5	918
TGGCGCATCACCCTCA-1_CRC1	Epithelial cells type5	1015
GCTGGGTTCAGGCCCA-1_CRC1	Epithelial cells type5	780
GTCGTAAGTCCGTGAC-1_CRC1	Epithelial cells type5	1019
GGCTGGTGTTCCAACA-1_CRC1	Epithelial cells type5	913
CGATTGACACTCTGTC-1_CRC1	Epithelial cells type5	1240
CTAAGACTCACAATGC-1_CRC1	Epithelial cells type5	1040
TCAGGATAGACAAAGG-1_CRC1	Epithelial cells type5	1160
TGGCTGGCACGACGAA-1_CRC1	Epithelial cells type5	1416
CATCCACTCACCGTAA-1_CRC1	Epithelial cells type5	1095
CCTAAAGTCTTACCGC-1_CRC1	Epithelial cells type5	1111
CGGTTAAAGACGACGT-1_CRC1	Epithelial cells type5	1385
CAGCCGATCGTTGCCT-1_CRC1	Epithelial cells type5	1040
GTCTCGTTCTCCTATA-1_CRC1	Epithelial cells type5	1494
AGGCCACTCAGTGTTG-1_CRC1	Epithelial cells type5	1648

AGGTCCGTCAGCTGGC-1_CRC1	Epithelial cells type5	1599
CTCATTACAGACTCGC-1_CRC1	Epithelial cells type5	1308
GGGTCTGTCTCATTCA-1_CRC1	Epithelial cells type5	1446
CCTTACGAGACTGTAA-1_CRC1	Epithelial cells type5	1627
GTGGGTCAGACAAGCC-1_CRC1	Epithelial cells type5	1754
CGACTTCCAAAGTCAA-1_CRC2	Epithelial cells type6	1425
AGCGGTCAGGAGTCTG-1_CRC2	Epithelial cells type6	1253
ACTTGTTGTTGGACCC-1_CRC2	Epithelial cells type6	930
TCGTACCTCGCGATCG-1_CRC2	Epithelial cells type6	1079
CAGAATCCAATCCAAC-1_CRC2	Epithelial cells type6	1754
TCGTAGAAGAAGAAGC-1_CRC2	Epithelial cells type6	1347
CGTCACTGTCTGATTG-1_CRC2	Epithelial cells type6	1861
CGCTTCAAGCTAGTCT-1_CRC2	Epithelial cells type6	1398
CAACCAACACAAGTAA-1_CRC2	Epithelial cells type6	968
CAAGTTGGTCTGCAAT-1_CRC2	Epithelial cells type6	1052
GATGCTAAGATGGCGT-1_CRC2	Epithelial cells type6	525
TACAGTGTCAGTGTTG-1_CRC2	Epithelial cells type6	547
CGGAGTCTCCTCTAGC-1_CRC2	Epithelial cells type6	602
GTCGTAAGTGCGGTAA-1_CRC2	Epithelial cells type6	720
AAAGCAAGTCTAACGT-1_CRC2	Epithelial cells type6	878
ACGGGCTCATACGCTA-1_CRC2	Epithelial cells type6	630
TCTCTAAAGACACTAA-1_CRC2	Epithelial cells type6	710
ACGCCGAGTCGCATCG-1_CRC2	Epithelial cells type6	1037
GTGTTAGTCGTTGACA-1_CRC2	Epithelial cells type6	780
CACAGTAGTACAGTGG-1_CRC2	Epithelial cells type6	717
TGGCCAGAGCACACAG-1_CRC2	Epithelial cells type6	737
CGCTGGATCTCACATT-1_CRC2	Epithelial cells type6	615
CTTAACTGTTCCCGAG-1_CRC2	Epithelial cells type6	698
CGAATGTCACGGATAG-1_CRC2	Epithelial cells type6	633
GGAGCAAGTTCCATGA-1_CRC2	Epithelial cells type6	731
ATCTGCCCAAGCTGTT-1_CRC2	Epithelial cells type6	799
ACGGAGATCCCTGACT-1_CRC2	Epithelial cells type6	487
TCCACACAGCTCTCGG-1_CRC2	Epithelial cells type6	546
ATCCGAAGTCACTTCC-1_CRC2	Epithelial cells type6	847
TCGCGAGCACTTAACG-1_CRC2	Epithelial cells type6	1052
CCTATTAAGTCCATAC-1_CRC2	Epithelial cells type6	665
TTCTCCTTCCACTGGG-1_CRC2	Epithelial cells type6	1075
GTCCTCAAGAACAACT-1_CRC2	Epithelial cells type6	814
ACTGATGCACCGGAAA-1_CRC2	Epithelial cells type6	829
GTCACAAGTAAGCACG-1_CRC2	Epithelial cells type6	708
ATCGAGTCAAGGACTG-1_CRC2	Epithelial cells type6	417
GAAACTCCATACGCTA-1_CRC2	Epithelial cells type6	683
TTAGGACTCTGTTTGT-1_CRC2	Epithelial cells type6	732
GGTGTTACATTAACCG-1_CRC2	Epithelial cells type6	554
CAAGATCTCACCTTAT-1_CRC2	Epithelial cells type6	486
CTACACCGTCTCTCTG-1_CRC2	Epithelial cells type6	475
CCTATTACAGGGATTG-1_CRC2	Epithelial cells type6	1019
TGAGAGGGTGATGTCT-1_CRC2	Epithelial cells type6	794
ACCGTAAAGGCCATAG-1_CRC2	Epithelial cells type6	575
AGGCCGTGTAAAGGAG-1_CRC2	Epithelial cells type6	603
AGTCTTTAGACGCTTT-1_CRC2	Epithelial cells type6	443

GACGCGTGTCTAGCGC-1_CRC2	Epithelial cells type6	670
CTCCTAGAGCACACAG-1_CRC2	Epithelial cells type6	568
TGTGTTTCACATCTTT-1_CRC2	Epithelial cells type6	370
CGTTAGAGTAGCTAAA-1_CRC2	Epithelial cells type6	682
GACCTGGGTCGCTTCT-1_CRC2	Epithelial cells type6	626
CTGAAGTTCTGAGGGA-1_CRC2	Epithelial cells type6	447
GTTCTCGAGGAGTTTA-1_CRC2	Epithelial cells type6	435
CCACCTATCTGTTTGT-1_CRC2	Epithelial cells type6	546
GAACATCCAATAGAGT-1_CRC2	Epithelial cells type6	494
CAAGATCGTCAACATC-1_CRC2	Epithelial cells type6	578
CGCTTCAAGTTGAGAT-1_CRC2	Epithelial cells type6	261
ATAAGAGCAGCTCCGA-1_CRC2	Epithelial cells type6	792
TTGCCGTGTGTTTGTG-1_CRC2	Epithelial cells type6	744
CTTAGGATCACCTCGT-1_CRC2	Epithelial cells type6	875
CACTCCAGTAAGGGAA-1_CRC2	Epithelial cells type6	780
TCATTTGAGTGAATTG-1_CRC2	Epithelial cells type6	722
TACGGTAGTGGACGAT-1_CRC2	Epithelial cells type6	1759
GAGTCCGTCCAAGTAC-1_CRC2	Epithelial cells type6	923
TTTATGCGTCATCCCT-1_CRC2	Epithelial cells type6	1026
CTGTTTAAGGGCTTCC-1_CRC2	Epithelial cells type6	1009
CCAATCCCACTATCTT-1_CRC2	Epithelial cells type6	851
ATTACTCAGAAACCTA-1_CRC2	Epithelial cells type6	647
CTGTTTAAGGTGTTAA-1_CRC2	Epithelial cells type6	1113
ACACCGGGTCTTCGTC-1_CRC2	Epithelial cells type6	976
ATCTACTGTAATCACC-1_CRC2	Epithelial cells type6	622
GTGCTTCTCCTCATTA-1_CRC2	Epithelial cells type6	452
GCTCTGTAGTTAGCGG-1_CRC2	Epithelial cells type6	714
GTACGTAGTTCGCTAA-1_CRC2	Epithelial cells type6	1074
CAGTAACCACTAGTAC-1_CRC2	Epithelial cells type6	922
GGCGTGTAGTCACGCC-1_CRC2	Epithelial cells type6	772
GCGACCATCGTTACGA-1_CRC2	Epithelial cells type6	885
AATCCAGCAAGCCTAT-1_CRC2	Epithelial cells type6	804
CCACTACCATGGTCTA-1_CRC2	Epithelial cells type6	964
GACTACAGTGACAAAT-1_CRC2	Epithelial cells type6	1020
TTGCGTCAGCTGGAAC-1_CRC2	Epithelial cells type6	836
ATGAGGGGTTAAGAAC-1_CRC2	Epithelial cells type6	969
GTTCATTCAAGTTAAG-1_CRC2	Epithelial cells type6	792
GTCAAGTTCAACGGCC-1_CRC2	Epithelial cells type6	1120
CAGCTGGAGTATGACA-1_CRC2	Epithelial cells type6	1091
ACTTACTAGCTGGAAC-1_CRC1	Epithelial cells type6	878
GAGGTGACATGGTTGT-1_CRC1	Epithelial cells type6	856
ACAGCCGCATAAGACA-1_CRC2	Epithelial cells type6	536
CCGTGGAGTACCGAGA-1_CRC2	Epithelial cells type6	429
ACTGAGTAGGACCACA-1_CRC2	Epithelial cells type6	432
CAGAGAGTCCCTCAGT-1_CRC2	Epithelial cells type6	419
GAGGTGATCGGTTCGG-1_CRC2	Epithelial cells type6	437
TGGTTAGGTAACGTTC-1_CRC2	Epithelial cells type6	543
GCTGGGTTCACCATAG-1_CRC2	Epithelial cells type6	413
GATCAGTCATGTTCCC-1_CRC2	Epithelial cells type6	210
GGTGAAGTCGTTACAG-1_CRC2	Epithelial cells type6	283
ACGCAGCAGATATGGT-1_CRC2	Epithelial cells type6	179

GACGTTAAGGTAAACT-1_CRC2	Epithelial cells type6	534
GTGGGTCGTAAGTTCC-1_CRC2	Epithelial cells type6	234
GTTTCTAAGGCGTACA-1_CRC2	Epithelial cells type6	371
CGTAGCGGTGATGCCC-1_CRC2	Epithelial cells type6	345
CTGTGCTGTCTAGTCA-1_CRC2	Epithelial cells type6	233
TCTCTAAGTCTCCCTA-1_CRC2	Epithelial cells type6	253
GCATGATAGTCACGCC-1_CRC2	Epithelial cells type6	226
TGCTACCCATACAGCT-1_CRC2	Epithelial cells type6	249
GGATGTTTCATCGGAT-1_CRC2	Epithelial cells type6	843
TTCTACACATCGATGT-1_CRC2	Epithelial cells type6	681
CGAATGTAGAGCAATT-1_CRC2	Epithelial cells type6	371
TCATTACAGTCTCCTC-1_CRC2	Epithelial cells type6	351
CTTACCGAGCCGATTT-1_CRC2	Epithelial cells type6	825
GTGCATAAGAAGCCCA-1_CRC2	Epithelial cells type6	675
CAGCAGCGTCCAACTA-1_CRC2	Epithelial cells type6	486
CCCTCCTGTCAAAGAT-1_CRC2	Epithelial cells type6	515
TCTCATAGTCTGCCAG-1_CRC2	Epithelial cells type6	488
CATCCACAGTTTCCTT-1_CRC2	Epithelial cells type6	751
CACCACTAGCTGTCTA-1_CRC2	Epithelial cells type6	649
TGAGCCGCAAGTCATC-1_CRC2	Epithelial cells type6	699
ATCGAGTAGTCTTGCA-1_CRC2	Epithelial cells type6	816
AGCATACGTGCGCTTG-1_CRC2	Epithelial cells type6	507
GCATGATTCCAGGGCT-1_CRC2	Epithelial cells type6	515
CCACCTACATTACGAC-1_CRC2	Epithelial cells type6	466
AAGACCTGTTCCACAA-1_CRC2	Epithelial cells type6	301
TTAGGACGTTCGTTGA-1_CRC2	Epithelial cells type6	474
CGGACGTGTTAGATGA-1_CRC2	Epithelial cells type6	552
GTAGGCCGTATAATGG-1_CRC2	Epithelial cells type6	730
CCATTCGAGTCGTACT-1_CRC2	Epithelial cells type6	879
CAACCTCCAGGATCGA-1_CRC2	Epithelial cells type6	839
CAAGTTGGTGCCTGCA-1_CRC2	Epithelial cells type6	596
CATGCCTCATCAGTCA-1_CRC2	Epithelial cells type6	472
TCATTTGAGCAGGCTA-1_CRC2	Epithelial cells type6	657
CGTCCATCACTCTGTC-1_CRC2	Epithelial cells type6	640
GAAACTCAGTCTCAAC-1_CRC2	Epithelial cells type6	684
GTAGTCACACACGCTG-1_CRC2	Epithelial cells type6	985
CGAATGTCAGCGTTCG-1_CRC2	Epithelial cells type6	621
CATCGAATCTAACTCT-1_CRC2	Epithelial cells type6	687
CACCACTCAGCCTGTG-1_CRC2	Epithelial cells type6	598
ACCCACTCACGGACAA-1_CRC2	Epithelial cells type6	485
CCTATTACATTTCAGG-1_CRC2	Epithelial cells type6	704
GACTACACACGAAATA-1_CRC2	Epithelial cells type6	459
AGCAGCCTCCGCGTTT-1_CRC2	Epithelial cells type6	479
CAGCTGGTCCTAGTGA-1_CRC2	Epithelial cells type6	264
ACACCCTTCAACGGCC-1_CRC2	Epithelial cells type6	1186
AGAGCGATCAAACAAG-1_CRC2	Epithelial cells type6	892
TAAACCGCAGCTCGCA-1_CRC2	Epithelial cells type6	978
CAAGGCCGTCTCCACT-1_CRC2	Epithelial cells type6	991
CAAGATCAGTGTTTGC-1_CRC2	Epithelial cells type6	624
CGCGTTTTCTGCCAGG-1_CRC2	Epithelial cells type6	799
TGCTGCTTCACCATAG-1_CRC2	Epithelial cells type6	817

ACGGGTCTCATTATCC-1_CRC2	Epithelial cells type6	1061
ATGAGGGGTACCCAAT-1_CRC2	Epithelial cells type6	590
TCTGGAACAGTGGAGT-1_CRC2	Epithelial cells type6	906
TGAGCATCATCTCGCT-1_CRC2	Epithelial cells type6	848
CAGCGACGTCCAAGTT-1_CRC2	Epithelial cells type6	871
GCATACAAGCGTGTCC-1_CRC2	Epithelial cells type6	706
TCGGTAAGTTCGAATC-1_CRC2	Epithelial cells type6	739
CACATTTGTGGAAAGA-1_CRC2	Epithelial cells type6	1087
ACGCCAGGTGCTGTAT-1_CRC2	Epithelial cells type6	763
CCTTCCCGTATTCGTG-1_CRC2	Epithelial cells type6	812
AGCGGTCTCGAACGGA-1_CRC2	Epithelial cells type6	1862
TGGGCGTGTCAAACTC-1_CRC2	Epithelial cells type6	1381
GTACGTAAGCTGCAAG-1_CRC2	Epithelial cells type6	637
ACTTGTTCAAAGGTGC-1_CRC2	Epithelial cells type6	900
CACCACTCAGTGGAGT-1_CRC2	Epithelial cells type6	660
CTGTTTACAAGAAAGG-1_CRC2	Epithelial cells type6	1115
AGTTGGTGTACCGCTG-1_CRC2	Epithelial cells type6	720
CACATAGAGTAAGTAC-1_CRC2	Epithelial cells type6	290
CCGGGATAGTGAAGTT-1_CRC2	Epithelial cells type6	632
CCACCTACAATGTAAG-1_CRC2	Epithelial cells type6	529
CCTACCAAGCACACAG-1_CRC2	Epithelial cells type6	376
CAGAGAGCAAGGTGTG-1_CRC2	Epithelial cells type6	591
CACCACTAGATGTGGC-1_CRC2	Epithelial cells type6	564
ATTACTCCACAGTCGC-1_CRC2	Epithelial cells type6	460
CTGAAGTTCAAACCGT-1_CRC2	Epithelial cells type6	472
AACTGGTTCTACCTGC-1_CRC2	Epithelial cells type6	537
AAGGCAGTCGCTGATA-1_CRC2	Epithelial cells type6	454
CGATCGGCAGATAATG-1_CRC2	Epithelial cells type6	457
ACCGTAAGTAGAAGGA-1_CRC2	Epithelial cells type6	269
TACGGATGTCTAGGTT-1_CRC2	Epithelial cells type6	328
ACGATACAGGCCCGTT-1_CRC2	Epithelial cells type6	436
ATGAGGGCAACTGCTA-1_CRC2	Epithelial cells type6	419
TAGCCGGTCTTAACCT-1_CRC2	Epithelial cells type6	453
TGACTAGCATTCACTT-1_CRC2	Epithelial cells type6	576
GTTCATTAGCGTCAAG-1_CRC2	Epithelial cells type6	582
GTTCGGGAGGGCACTA-1_CRC2	Epithelial cells type6	615
TCAACGACAACACCTA-1_CRC2	Epithelial cells type6	732
GTGGGTCGTAGCGTGA-1_CRC2	Epithelial cells type6	393
TAAACCGCATTCCTGC-1_CRC2	Epithelial cells type6	455
AGCGGTCTCCTTCAAT-1_CRC2	Epithelial cells type6	662
AAGTCTGAGACCGGAT-1_CRC2	Epithelial cells type6	648
ATTGGTGGTGTTCGAT-1_CRC2	Epithelial cells type6	606
CGATTGATCGGACAAG-1_CRC2	Epithelial cells type6	590
GTTTCTATCATCACCC-1_CRC2	Epithelial cells type6	594
CAGCGACGTAGCAAAT-1_CRC2	Epithelial cells type6	556
CTAATGGGTCAAAGCG-1_CRC2	Epithelial cells type6	391
GGGCACTCAAAGAATC-1_CRC2	Epithelial cells type6	692
CACCACTTCTGCGACG-1_CRC2	Epithelial cells type6	653
AAGCCGCCAGTCGATT-1_CRC2	Epithelial cells type6	777
CAACTAGCACCATCCT-1_CRC2	Epithelial cells type6	790
AAGGAGCGTGGCAAAC-1_CRC2	Epithelial cells type6	1299

TATTACCCAGGTGGAT-1_CRC2	Epithelial cells type6	1039
TAGAGCTTCACCAGGC-1_CRC2	Epithelial cells type6	890
GTGCATAGTTCGAATC-1_CRC2	Epithelial cells type6	863
AAACGGGCATGCCTAA-1_CRC2	Epithelial cells type6	769
CCACCTAGTAGCTAAA-1_CRC2	Epithelial cells type6	709
CCAATCCGTACCAGTT-1_CRC2	Epithelial cells type6	636
CTCTACGTCTGAAAGA-1_CRC2	Epithelial cells type6	377
GGAGCAAGTCGTCTTC-1_CRC2	Epithelial cells type6	651
GTCTCGTCAGTAAGCG-1_CRC2	Epithelial cells type6	972
CAAGTTGCAAAGGTGC-1_CRC2	Epithelial cells type6	835
GCCAAATTCCTAGAAC-1_CRC2	Epithelial cells type6	550
ACATACGGTTTGGGCC-1_CRC2	Epithelial cells type6	563
GCCAAATAGCGTCAAG-1_CRC2	Epithelial cells type6	702
CGAACATCACTTCGAA-1_CRC2	Epithelial cells type6	854
GTGGGTCCAGATCCAT-1_CRC2	Epithelial cells type6	902
TGCGTGGGTACGCACC-1_CRC2	Epithelial cells type6	608
CGCTTCAAGTCAAGGC-1_CRC2	Epithelial cells type6	475
CTTAGGAGTCGGCTCA-1_CRC2	Epithelial cells type6	479
GATCAGTCACAAGCCC-1_CRC2	Epithelial cells type6	751
CAAGATCCATTATCTC-1_CRC2	Epithelial cells type6	450
TCGGGACAGTGGGATC-1_CRC2	Epithelial cells type6	418
CGAATGTCATGGTAGG-1_CRC2	Epithelial cells type6	384
CTGGTCTTCGTACCGG-1_CRC2	Epithelial cells type6	451
ACGCCGATCCTTGCCA-1_CRC2	Epithelial cells type6	326
GACGTTATCAATACCG-1_CRC2	Epithelial cells type6	367
GTACTTTTCTTGACGA-1_CRC0	Epithelial cells type6	1591
TAGACCAGTACGACCC-1_CRC0	Epithelial cells type6	1978
GCGACCATCCACTCCA-1_CRC0	Epithelial cells type6	1400
TCTTTCCTCTGTACGA-1_CRC0	Epithelial cells type6	1664
AACTTTCAGAGCTTCT-1_CRC2	Epithelial cells type6	543
GATGAGGTCATGGTCA-1_CRC2	Epithelial cells type6	692
ACGGGTCCACCAGGTC-1_CRC2	Epithelial cells type6	494
CAGGTGCGTGAAATCA-1_CRC2	Epithelial cells type6	492
GGAATAATCTCTGAGA-1_CRC2	Epithelial cells type6	273
CGTCTACAGACAAGCC-1_CRC2	Epithelial cells type6	201
GGAACTTTCGATCCCT-1_CRC2	Epithelial cells type6	218
CTCGAAATCGTGGGAA-1_CRC1	Epithelial cells type6	428
CATATGGCATGTAAGA-1_CRC2	Epithelial cells type6	466
ACATCAGGTTATGTGC-1_CRC2	Epithelial cells type6	320
CAGAATCAGTACGTAA-1_CRC2	Epithelial cells type6	292
TAGAGCTGTTACGACT-1_CRC2	Epithelial cells type6	177
TACGGATAGCCAGAAC-1_CRC2	Epithelial cells type6	298
TCGGGACCAAACGTGG-1_CRC2	Epithelial cells type6	196
AACCATGCATAGGATA-1_CRC2	Epithelial cells type6	259
CAGCCGAAGCAGGCTA-1_CRC2	Epithelial cells type6	274
GAACATCCAGGGTATG-1_CRC2	Epithelial cells type6	137
TCGCGTTGTTCGGCAC-1_CRC2	Epithelial cells type6	315
GATCGATCAATGTAAG-1_CRC2	Epithelial cells type6	609
GTCTTCGGTGCAACGA-1_CRC2	Epithelial cells type6	285
TACCTATCAGCAGTTT-1_CRC2	Epithelial cells type6	227
GACGGCTTCACGCGGT-1_CRC2	Epithelial cells type6	423

ATGTGTGAGCCTTGAT-1_CRC2	Epithelial cells type6	182
TGCGTGGGTACAGCAG-1_CRC2	Epithelial cells type6	120
GGGATGACAGCATGAG-1_CRC2	Epithelial cells type6	223
ACTGTCCGTTCCAACA-1_CRC2	Epithelial cells type6	186
GGGTCTGGTAAGAGAG-1_CRC2	Epithelial cells type6	339
CGTAGGCTCTGTCTAT-1_CRC2	Epithelial cells type6	214
GGCTGGTGTCCAAGTT-1_CRC2	Epithelial cells type6	292
TTTACTGGTGCGCTTG-1_CRC2	Epithelial cells type6	187
GCTGCAGGTTCGTCTC-1_CRC2	Epithelial cells type6	299
TTCGAAGGTTCCTCCA-1_CRC2	Epithelial cells type6	225
ATCATGGTCTGCCAGG-1_CRC2	Epithelial cells type6	204
TCAGCAAGTTCCTCCA-1_CRC2	Epithelial cells type6	342
TTATGCTTCATAGCAC-1_CRC2	Epithelial cells type6	349
TGGCCAGAGACTGGGT-1_CRC2	Epithelial cells type6	1102
CACACTCCACATCCGG-1_CRC2	Epithelial cells type6	377
AGACGTTTCAGCAACT-1_CRC2	Epithelial cells type6	447
CTCGAGGCAGGATCGA-1_CRC2	Epithelial cells type6	236
GGAGCAACAGTAAGCG-1_CRC2	Epithelial cells type6	225
TCGCGAGCAAGAAAGG-1_CRC2	Epithelial cells type6	205
CGTCCATAGCACCGTC-1_CRC2	Epithelial cells type6	797
CGGACGTGTATCACCA-1_CRC2	Epithelial cells type6	450
CTGCCTAGTTAAGACA-1_CRC2	Epithelial cells type6	541
TAGACCAAGTGTACTC-1_CRC2	Epithelial cells type6	425
CCCAATCGTCTAAAGA-1_CRC2	Epithelial cells type6	207
ATCTACTGTACCGCTG-1_CRC2	Epithelial cells type6	132
GCTGCGATCATAAAGG-1_CRC2	Epithelial cells type6	346
CACACTCGTTTGTTGG-1_CRC2	Epithelial cells type6	609
CCAATCCCAAGCGTAG-1_CRC2	Epithelial cells type6	443
CGATTGAAGACTACAA-1_CRC2	Epithelial cells type6	950
CACAAACTCCTCCTAG-1_CRC2	Epithelial cells type6	523
TGAGCCGAGTGGTAGC-1_CRC2	Epithelial cells type6	485
GGCTCGATCCTGCAGG-1_CRC2	Epithelial cells type6	365
AAAGCAAGTCCATCCT-1_CRC2	Epithelial cells type6	371
CAACCAATCGCCATAA-1_CRC2	Epithelial cells type6	457
GTTAAGCAGTAGGTGC-1_CRC2	Epithelial cells type6	806
AGATCTGGTAGCGTGA-1_CRC2	Epithelial cells type6	294
AGATTGCCACGGTAGA-1_CRC2	Epithelial cells type6	249
GCATGTAAGGGAAACA-1_CRC2	Epithelial cells type6	318
ACATACGCACAGCCCA-1_CRC2	Epithelial cells type6	400
TTTATGCCAATCCAAC-1_CRC2	Epithelial cells type6	316
CGCGGTACAGCTATTG-1_CRC2	Epithelial cells type6	876
TAAACCGTCGAACTGT-1_CRC2	Epithelial cells type6	404
AGGGAGTGTCGCATCG-1_CRC2	Epithelial cells type6	559
GGGAATGGTTTGGCGC-1_CRC2	Epithelial cells type6	553
ACTGAGTCAAGTAGTA-1_CRC2	Epithelial cells type6	475
TCGGGACTCGGCTTGG-1_CRC2	Epithelial cells type6	442
TCTATTGTCTCGCATC-1_CRC2	Epithelial cells type6	273
TTAGGACGTACTTCTT-1_CRC2	Epithelial cells type6	315
TGCCCTAAGCCACCTG-1_CRC2	Epithelial cells type6	393
CTAATGGCAACGATGG-1_CRC2	Epithelial cells type6	318
TGCCCATTCGGAAACG-1_CRC2	Epithelial cells type6	530

GACTGCGCAGCCTTTC-1_CRC2	Epithelial cells type6	308
TCGGTAATCCTTGGTC-1_CRC2	Epithelial cells type6	143
ATTGGACCATGGTCTA-1_CRC2	Epithelial cells type6	29
GTTTCTAAGATAGTCA-1_CRC2	Epithelial cells type6	225
TTTGTCAGTCGTCTTC-1_CRC2	Epithelial cells type6	151
TTTACTGTCGGAGCAA-1_CRC2	Epithelial cells type6	360
GACAGAGAGGGATACC-1_CRC2	Epithelial cells type6	174
TTTGCGCCAAAGTGCG-1_CRC2	Epithelial cells type6	257
GGAATAACAATGACCT-1_CRC2	Epithelial cells type6	610
TCTATTGCAGGCTGAA-1_CRC2	Epithelial cells type6	250
TACCTATTCCACTGGG-1_CRC2	Epithelial cells type6	561
TACTTACGTCCAAGTT-1_CRC2	Epithelial cells type6	407
ACCAGTAAGATACACA-1_CRC2	Epithelial cells type6	292
AGAGCTTAGATACACA-1_CRC2	Epithelial cells type6	131
CAGCGACCAACACGCC-1_CRC2	Epithelial cells type6	652
GGGCATCCAAGCCATT-1_CRC2	Epithelial cells type6	524
TAGACCATCCTAGTGA-1_CRC2	Epithelial cells type6	389
CACTCCATCTACTATC-1_CRC2	Epithelial cells type6	445
TAAACCGAGATATGGT-1_CRC2	Epithelial cells type6	298
CTGAAGTTCTATGTGG-1_CRC2	Epithelial cells type6	446
AACCGCGCAAACTGCT-1_CRC2	Epithelial cells type6	131
TACGGTACATGGTCTA-1_CRC2	Epithelial cells type6	299
CAAGTTGTCTTTACGT-1_CRC2	Epithelial cells type6	478
TGAAAGAGTAGCGCTC-1_CRC2	Epithelial cells type6	628
AGACGTTAGTCGCCGT-1_CRC2	Epithelial cells type6	426
CGCGTTTGTGCACTTA-1_CRC2	Epithelial cells type6	507
ATCCGAAGTTCTGAAC-1_CRC2	Epithelial cells type6	2011
CCACGGACACCGAATT-1_CRC2	Epithelial cells type6	785
CAGCGACCAGCATACT-1_CRC2	Epithelial cells type6	1143
TGGTTAGAGCACCGCT-1_CRC2	Epithelial cells type6	1375
CCACCTATCAACGGGA-1_CRC2	Epithelial cells type6	1091
AACTCAGGTCCGAGTC-1_CRC2	Epithelial cells type6	990
CACCTTGCACAAGACG-1_CRC2	Epithelial cells type6	1302
TACTTGTTCAGGCAAG-1_CRC2	Epithelial cells type6	980
AACGTTGTCATAGCAC-1_CRC2	Epithelial cells type6	776
GGACATTCAAGAAGAG-1_CRC2	Epithelial cells type6	1048
CTCTGGTGTGCCTTGG-1_CRC2	Epithelial cells type6	243
CAACCTCAGAGACTTA-1_CRC2	Epithelial cells type6	478
CTCAGAAAGATATGCA-1_CRC2	Epithelial cells type6	250
GAATGAAAGTCTCAAC-1_CRC2	Epithelial cells type6	272
ACACCAAGTACACCGC-1_CRC2	Epithelial cells type6	365
GGGTTGCGTCCGAATT-1_CRC2	Epithelial cells type6	396
AACCATGAGTGCGTGA-1_CRC2	Epithelial cells type6	764
GATCTAGGTAGGGACT-1_CRC2	Epithelial cells type6	394
ACGGCCACACGCATCG-1_CRC2	Epithelial cells type6	441
GTGAAGGCAATGGAAT-1_CRC2	Epithelial cells type6	471
CTCATTACACGTTGGC-1_CRC2	Epithelial cells type6	485
GTAGGCCTCCCTCTTT-1_CRC2	Epithelial cells type6	448
ATCACGAAGCTAAGAT-1_CRC2	Epithelial cells type6	280
ACACCAACAGCCACCA-1_CRC2	Epithelial cells type6	417
TTTGTCATCTCGAGTA-1_CRC2	Epithelial cells type6	418

CTGATCCCACCTATCC-1_CRC2	Epithelial cells type6	355
GGAACTTGTTAAGTAG-1_CRC2	Epithelial cells type6	416
GTCATTTGTCGCGTGT-1_CRC2	Epithelial cells type6	642
ACTGATGAGGCTAGGT-1_CRC2	Epithelial cells type6	397
CAGTAACAGGAGCGAG-1_CRC2	Epithelial cells type6	528
AATCCAGAGTGGAGAA-1_CRC2	Epithelial cells type6	622
CGTCTACGTGTGAAAT-1_CRC2	Epithelial cells type6	440
TTCTACAAGATGTCGG-1_CRC2	Epithelial cells type6	383
GGGCATCCAGGAATGC-1_CRC2	Epithelial cells type6	501
GACGCGTGTACGACCC-1_CRC2	Epithelial cells type6	827
ACTGATGAGTCATCCA-1_CRC2	Epithelial cells type6	479
GTTCATTAGAGTTGGC-1_CRC2	Epithelial cells type6	587
CATTCGCTCATGTCTT-1_CRC2	Epithelial cells type6	1612
CGAGCACAGGTAAACT-1_CRC2	Epithelial cells type6	823
ATAGACCTCAGTCAGT-1_CRC2	Epithelial cells type6	607
CGCGTTTAGACAGAGA-1_CRC2	Epithelial cells type6	978
TCGGGACGTTCGCGAC-1_CRC2	Epithelial cells type6	902
CACATAGAGTGTTAGA-1_CRC2	Epithelial cells type6	599
TGAGCATAGGTGCTTT-1_CRC2	Epithelial cells type6	729
ACACCAAGTCATATGC-1_CRC2	Epithelial cells type6	789
GACTGCGAGCACGCCT-1_CRC2	Epithelial cells type6	521
AGAGTGGCATGCCCGA-1_CRC2	Epithelial cells type6	877
CCATGTCCATGGGAAC-1_CRC2	Epithelial cells type6	508
TGTTCCGTCTCGTTTA-1_CRC2	Epithelial cells type6	811
CCGGGATAGTGGAGAA-1_CRC2	Epithelial cells type6	1056
AGTTGGTCACATGACT-1_CRC2	Epithelial cells type6	753
GACACGCTCCAAGTAC-1_CRC2	Epithelial cells type6	504
