# Supplementary material for: Single-Cell RNA Sequencing Reveals the Role of Epithelial Cell Marker Genes in Predicting the Prognosis of Colorectal Cancer Patients
Source: Dis Markers. 2022 Aug 1;2022:8347125. doi: 10.1155/2022/8347125 (PMC9372514; doi:10.1155/2022/8347125)
Supplement: Supplementary 2 — Table S1: clinical characteristics of the four CRC patients. Table S2: CNV scores of epithelial cell subclusters. Table S3: MEC subclusters of marker genes. Table S4: MECRG signature using the TCGA-CRC training cohort. Table S5: MECRG signature using the GSE17538 validation cohort. [file 8347125.f2.zip › Table S3.pdf]

TableS3 MEC subclusters marker genes

gene	p_val	avg_log2FC	pct.1	pct.2	fdr	cluster	Epithelial cell type
LEFTY1	0	1.979067087	0.713	0.119	0	0	1
TACSTD2	0	1.97479066	0.739	0.078	0	0	1
OLFM4	0	1.966423716	0.88	0.227	0	0	1
IFITM3	0	1.872314207	0.882	0.324	0	0	1
LCN2	0	1.786094489	0.79	0.181	0	0	1
MT1E	0	1.747966621	0.743	0.139	0	0	1
SMIM22	0	1.726396352	0.923	0.31	0	0	1
TSPAN8	0	1.670541211	0.947	0.339	0	0	1
S100P	0	1.649633037	0.779	0.166	0	0	1
CEACAM5	0	1.61692211	0.877	0.226	0	0	1
PIGR	0	1.608086524	0.848	0.255	0	0	1
ATP1B1	0	1.528015307	0.904	0.306	0	0	1
TMEM141	0	1.521926782	0.873	0.341	0	0	1
KRT18	0	1.512966764	0.95	0.418	0	0	1
S100A11	0	1.507094314	0.97	0.665	0	0	1
AREG	0	1.479826204	0.793	0.25	0	0	1
IFITM1	0	1.427289414	0.83	0.346	0	0	1
MT-ATP6	0	1.359829063	0.996	0.985	0	0	1
PRDX5	0	1.357767992	0.937	0.61	0	0	1
UQCRH	0	1.312470716	0.944	0.66	0	0	1
NQO1	0	1.293719735	0.736	0.186	0	0	1
PPDPF	0	1.285673896	0.966	0.672	0	0	1
TXN	0	1.28384061	0.947	0.563	0	0	1
TSPAN1	0	1.281711607	0.752	0.183	0	0	1
CEACAM6	0	1.275863839	0.614	0.127	0	0	1
NBL1	0	1.274264316	0.83	0.249	0	0	1
C19orf33	0	1.270335705	0.822	0.22	0	0	1
TNFRSF12	0	1.264858348	0.654	0.151	0	0	1
UQCRQ	0	1.258991996	0.948	0.594	0	0	1
CD24	0	1.248403201	0.939	0.398	0	0	1
ATP5MD	0	1.239143132	0.946	0.644	0	0	1
MT-CO2	0	1.238309061	0.998	0.992	0	0	1
CA9	0	1.236290859	0.597	0.105	0	0	1
ELOB	0	1.222227076	0.963	0.714	0	0	1
SPINT2	0	1.219812371	0.934	0.417	0	0	1
CSTB	0	1.215948137	0.943	0.537	0	0	1
PLA2G16	0	1.215190595	0.729	0.217	0	0	1
AGR3	0	1.205093309	0.781	0.182	0	0	1
MT1M	0	1.198492026	0.518	0.046	0	0	1
GSTP1	1.07E-298	1.184360866	0.95	0.645	2.08E-294	0	1
IFI27	0	1.178575462	0.937	0.388	0	0	1
SOD1	3.51E-275	1.178450607	0.918	0.608	6.80E-271	0	1
SLC12A2	0	1.171135744	0.876	0.282	0	0	1
MT-ND3	0	1.168180708	0.993	0.981	0	0	1
MLEC	4.99E-288	1.151886161	0.861	0.388	9.68E-284	0	1
DSTN	9.31E-306	1.138288596	0.926	0.463	1.81E-301	0	1
EPCAM	2.78E-293	1.134750406	0.951	0.388	5.40E-289	0	1
TESC	0	1.134454137	0.58	0.083	0	0	1

MT-ND1	0	1.131197501	0.993	0.959	0	0	1
PPA1	1.38E-293	1.129256562	0.909	0.458	2.68E-289	0	1
PDZK1IP1	0	1.123742146	0.665	0.142	0	0	1
TMEM123	3.78E-226	1.112299872	0.908	0.515	7.33E-222	0	1
AQP5	0	1.097972271	0.478	0.034	0	0	1
CYSTM1	0	1.095210942	0.881	0.336	0	0	1
S100A6	5.33E-258	1.072127113	0.985	0.834	1.03E-253	0	1
MINOS1	4.57E-252	1.071787831	0.912	0.536	8.86E-248	0	1
ST14	0	1.062487337	0.804	0.258	0	0	1
FXYD3	4.74E-302	1.050257473	0.925	0.383	9.19E-298	0	1
MT-CYB	0	1.045905788	0.997	0.981	0	0	1
TGFBI	0	1.045700407	0.758	0.227	0	0	1
REXO2	0	1.030498433	0.77	0.269	0	0	1
S100A10	1.72E-271	1.029286318	0.974	0.658	3.34E-267	0	1
EPB41L2	0	1.023395635	0.686	0.156	0	0	1
PERP	1.04E-289	1.020056314	0.883	0.341	2.02E-285	0	1
MT-CO3	0	1.014009258	0.999	0.993	0	0	1
RAC1	2.82E-259	1.012535477	0.953	0.6	5.48E-255	0	1
CD151	5.24E-275	0.990268757	0.807	0.313	1.02E-270	0	1
TMSB10	2.41E-293	0.981870893	0.997	0.983	4.67E-289	0	1
PCBD1	4.94E-299	0.981641895	0.809	0.299	9.59E-295	0	1
ADIRF	4.11E-234	0.974150373	0.497	0.123	7.97E-230	0	1
KRT8	1.95E-245	0.972749687	0.951	0.451	3.79E-241	0	1
RAB25	0	0.965909904	0.763	0.236	0	0	1
ATP5IF1	5.49E-200	0.962142235	0.886	0.53	1.06E-195	0	1
GPRC5A	0	0.959100592	0.645	0.156	0	0	1
HSPE1	8.41E-226	0.958262945	0.928	0.596	1.63E-221	0	1
ALDH2	4.72E-273	0.957027836	0.755	0.272	9.15E-269	0	1
HSPB1	1.24E-180	0.956481702	0.874	0.454	2.40E-176	0	1
GPR160	0	0.948805697	0.707	0.219	0	0	1
NPDC1	0	0.947476149	0.747	0.202	0	0	1
PHLDA2	3.71E-255	0.946727556	0.718	0.244	7.19E-251	0	1
ELF3	6.32E-264	0.946144412	0.929	0.368	1.23E-259	0	1
HIST1H4C	1.18E-126	0.945618185	0.729	0.386	2.29E-122	0	1
ANXA3	0	0.943298656	0.623	0.153	0	0	1
EIF1AX	3.37E-227	0.941279367	0.877	0.482	6.53E-223	0	1
FHL2	0	0.939498254	0.675	0.181	0	0	1
CLDN3	1.27E-269	0.929861232	0.898	0.337	2.46E-265	0	1
NDUFC2	9.91E-212	0.924905636	0.877	0.483	1.92E-207	0	1
QTRT1	1.80E-278	0.924301259	0.707	0.233	3.49E-274	0	1
MCRIP2	1.20E-282	0.920365149	0.691	0.227	2.33E-278	0	1
BACE2	1.44E-307	0.919472384	0.686	0.194	2.80E-303	0	1
TRPM4	0	0.911619193	0.589	0.136	0	0	1
MT-ND4	0	0.906926443	0.995	0.991	0	0	1
ATP5MG	6.93E-238	0.905240146	0.964	0.838	1.34E-233	0	1
GNG5	1.59E-213	0.90523128	0.902	0.5	3.08E-209	0	1
MUC1	0	0.905080372	0.574	0.123	0	0	1
ATP5MC3	1.44E-204	0.899394975	0.916	0.58	2.79E-200	0	1
S100A16	9.85E-282	0.897046382	0.712	0.222	1.91E-277	0	1
ZFAS1	5.93E-151	0.895944423	0.931	0.615	1.15E-146	0	1
SH3YL1	2.14E-298	0.894610909	0.722	0.23	4.15E-294	0	1

LDHA	2.67E-180	0.891049051	0.938	0.623	5.18E-176	0	1
TM4SF1	2.49E-235	0.886289346	0.553	0.143	4.82E-231	0	1
TMEM54	7.83E-289	0.878837462	0.758	0.243	1.52E-284	0	1
NDUFB3	3.47E-210	0.877121835	0.838	0.421	6.73E-206	0	1
TMEM176A	1.89E-307	0.867752627	0.696	0.198	3.67E-303	0	1
SFN	2.71E-294	0.865688884	0.675	0.188	5.25E-290	0	1
TSTA3	1.74E-227	0.86539063	0.769	0.314	3.38E-223	0	1
PRDX1	1.22E-191	0.864987431	0.905	0.552	2.36E-187	0	1
COX5B	7.28E-221	0.864515693	0.956	0.692	1.41E-216	0	1
PDLIM1	8.01E-259	0.864230872	0.787	0.291	1.55E-254	0	1
EDN1	5.92E-260	0.862411902	0.399	0.07	1.15E-255	0	1
ASS1	2.32E-177	0.859600051	0.468	0.139	4.50E-173	0	1
NDUFAB1	2.78E-203	0.855225401	0.832	0.409	5.38E-199	0	1
GPX2	1.14E-240	0.845550431	0.839	0.316	2.20E-236	0	1
CLDN4	7.46E-221	0.843751345	0.902	0.385	1.45E-216	0	1
DBI	5.69E-171	0.843635048	0.895	0.567	1.10E-166	0	1
MT1G	5.13E-293	0.842840088	0.502	0.097	9.96E-289	0	1
RPL37A	0	0.842040623	0.992	0.975	0	0	1
C12orf75	9.94E-227	0.836342419	0.831	0.348	1.93E-222	0	1
TMEM106C	3.51E-238	0.834652195	0.707	0.255	6.81E-234	0	1
MDK	0	0.83077159	0.815	0.263	0	0	1
FAM84A	5.35E-287	0.828803812	0.651	0.181	1.04E-282	0	1
LIPH	3.94E-290	0.828368951	0.56	0.133	7.65E-286	0	1
RPL37	5.74E-294	0.827501551	0.986	0.974	1.11E-289	0	1
LAPTM4B	8.28E-290	0.82657054	0.59	0.153	1.61E-285	0	1
RPS7	5.56E-281	0.822331288	0.981	0.964	1.08E-276	0	1
AGPAT2	3.30E-242	0.81821255	0.699	0.245	6.40E-238	0	1
KIF5B	3.44E-175	0.817179138	0.85	0.438	6.67E-171	0	1
EIF4EBP1	1.62E-266	0.815816886	0.647	0.199	3.14E-262	0	1
COX6A1	5.49E-204	0.814941977	0.948	0.673	1.07E-199	0	1
ATP5PF	3.28E-179	0.813801221	0.907	0.565	6.36E-175	0	1
TMC5	3.72E-223	0.809957802	0.579	0.177	7.21E-219	0	1
TPM2	0	0.804638346	0.419	0.032	0	0	1
SELENBP1	1.09E-256	0.802313943	0.575	0.154	2.11E-252	0	1
MUC4	1.34E-268	0.800387817	0.482	0.099	2.59E-264	0	1
CLDN2	0	0.800374763	0.463	0.061	0	0	1
RPL7A	2.61E-274	0.800197046	0.983	0.943	5.05E-270	0	1
BCL2L15	0	0.793342257	0.546	0.106	0	0	1
RBP4	0	0.792649554	0.453	0.056	0	0	1
RPS3	2.27E-287	0.792549442	0.995	0.991	4.40E-283	0	1
APP	1.01E-222	0.791332855	0.825	0.318	1.96E-218	0	1
RHOC	1.76E-193	0.790973208	0.84	0.388	3.41E-189	0	1
ANXA2	7.64E-185	0.788455181	0.925	0.499	1.48E-180	0	1
SNRPF	2.69E-160	0.788090793	0.821	0.449	5.22E-156	0	1
PDCD5	7.41E-228	0.785591919	0.771	0.314	1.44E-223	0	1
PEBP1	4.39E-156	0.781515659	0.864	0.518	8.51E-152	0	1
SERINC2	2.65E-238	0.781284351	0.665	0.218	5.14E-234	0	1
TMEM176B	2.89E-230	0.777741492	0.74	0.263	5.61E-226	0	1
COX20	5.14E-202	0.777565694	0.785	0.336	9.97E-198	0	1
MT-ND2	3.73E-244	0.775582252	0.996	0.988	7.24E-240	0	1
COX8A	7.10E-161	0.773356644	0.918	0.645	1.38E-156	0	1

CLDN7	1.81E-233	0.769575455	0.784	0.28	3.51E-229	0	1
NOXO1	0	0.769062156	0.473	0.081	0	0	1
TCEA3	0	0.767182131	0.562	0.119	0	0	1
ASCL2	2.20E-221	0.766012238	0.623	0.191	4.26E-217	0	1
NDUFC1	3.27E-188	0.761953273	0.8	0.376	6.35E-184	0	1
TALDO1	1.71E-170	0.757309145	0.806	0.406	3.32E-166	0	1
SOD3	6.99E-295	0.755354033	0.51	0.102	1.36E-290	0	1
RPS2	5.12E-248	0.752463391	0.992	0.997	9.93E-244	0	1
TDGF1	0	0.750004705	0.477	0.068	0	0	1
NDUFS5	2.18E-157	0.749206312	0.906	0.575	4.23E-153	0	1
MACROD1	1.31E-283	0.747262219	0.556	0.139	2.53E-279	0	1
COX6B1	5.68E-189	0.744588453	0.954	0.75	1.10E-184	0	1
LSR	3.87E-211	0.742368221	0.74	0.277	7.51E-207	0	1
TKT	3.93E-190	0.739370409	0.806	0.374	7.62E-186	0	1
MT1X	1.52E-190	0.739106063	0.659	0.24	2.95E-186	0	1
EMP2	4.63E-235	0.736903675	0.62	0.196	8.99E-231	0	1
LGALS3	1.40E-218	0.73611805	0.943	0.504	2.72E-214	0	1
LGALS4	1.38E-280	0.735400371	0.928	0.376	2.68E-276	0	1
PRSS23	2.57E-285	0.733612506	0.589	0.137	4.98E-281	0	1
FAM3D	2.60E-263	0.730179563	0.762	0.233	5.04E-259	0	1
MSRB2	5.20E-220	0.729986019	0.636	0.223	1.01E-215	0	1
MT-CO1	1.26E-264	0.727210221	0.998	0.994	2.44E-260	0	1
KRTCAP3	3.52E-250	0.723530035	0.601	0.177	6.82E-246	0	1
MDH2	5.74E-156	0.72086517	0.811	0.427	1.11E-151	0	1
TINAGL1	3.16E-212	0.719587392	0.514	0.149	6.13E-208	0	1
NDUFA1	1.11E-150	0.713375964	0.911	0.599	2.16E-146	0	1
DYNLL1	1.21E-142	0.711786013	0.937	0.629	2.34E-138	0	1
BLVRB	1.02E-221	0.710079639	0.678	0.242	1.97E-217	0	1
CXADR	2.07E-233	0.709170589	0.683	0.219	4.01E-229	0	1
NACA	2.59E-212	0.707636561	0.976	0.894	5.01E-208	0	1
CD46	2.54E-185	0.705844153	0.808	0.354	4.93E-181	0	1
RPS24	2.63E-266	0.702721956	0.99	0.98	5.09E-262	0	1
COX7C	3.42E-196	0.70244311	0.964	0.838	6.62E-192	0	1
MT2A	1.14E-168	0.700322165	0.671	0.261	2.21E-164	0	1
TIMM8B	6.82E-166	0.700301225	0.766	0.364	1.32E-161	0	1
HSPD1	3.45E-138	0.697364548	0.861	0.494	6.68E-134	0	1
HINT1	5.70E-183	0.697281702	0.956	0.775	1.11E-178	0	1
MPC2	5.13E-159	0.695451186	0.816	0.398	9.95E-155	0	1
AP1S1	7.84E-208	0.69359877	0.671	0.248	1.52E-203	0	1
NTHL1	8.81E-229	0.69146357	0.515	0.139	1.71E-224	0	1
PLCB4	1.21E-222	0.689420755	0.431	0.099	2.34E-218	0	1
PSMB2	7.17E-165	0.687011588	0.785	0.381	1.39E-160	0	1
TMPRSS4	1.17E-264	0.685378693	0.516	0.122	2.27E-260	0	1
COX7B	9.80E-147	0.684224412	0.923	0.648	1.90E-142	0	1
NDRG1	1.76E-89	0.68120488	0.465	0.204	3.42E-85	0	1
CLTB	5.55E-153	0.679782112	0.787	0.373	1.08E-148	0	1
MAL2	3.16E-178	0.679563796	0.624	0.218	6.13E-174	0	1
SNRPG	7.60E-132	0.679536279	0.889	0.574	1.47E-127	0	1
NME4	3.74E-220	0.67499947	0.601	0.193	7.25E-216	0	1
LMNA	8.00E-191	0.674961376	0.776	0.319	1.55E-186	0	1
SEM1	1.04E-147	0.67459882	0.892	0.526	2.02E-143	0	1

MPZL2	7.81E-296	0.674577714	0.469	0.09	1.52E-291	0	1
CDX2	2.28E-218	0.671636625	0.607	0.191	4.41E-214	0	1
TUBB4B	9.06E-128	0.671149853	0.718	0.348	1.76E-123	0	1
FERMT1	5.05E-272	0.669921861	0.493	0.109	9.80E-268	0	1
COX7A2	2.40E-152	0.667870463	0.957	0.746	4.66E-148	0	1
RPS21	6.08E-192	0.660913402	0.977	0.918	1.18E-187	0	1
TFF3	4.02E-282	0.657042191	0.95	0.507	7.79E-278	0	1
NAPRT	7.04E-204	0.655937337	0.623	0.215	1.37E-199	0	1
RPL36A	8.13E-181	0.655242336	0.966	0.88	1.58E-176	0	1
TRIM2	1.55E-215	0.654930439	0.595	0.186	3.00E-211	0	1
VDAC1	2.05E-150	0.654600019	0.878	0.472	3.98E-146	0	1
NDUFB4	2.78E-136	0.653783304	0.88	0.51	5.40E-132	0	1
C8orf59	1.59E-153	0.652934339	0.811	0.419	3.08E-149	0	1
MRPS34	2.24E-143	0.652416583	0.748	0.374	4.34E-139	0	1
ARPC1A	2.10E-182	0.651973123	0.725	0.303	4.07E-178	0	1
MARCKSL	5.89E-197	0.651550697	0.898	0.405	1.14E-192	0	1
ATP5MF	6.36E-117	0.650505352	0.889	0.607	1.23E-112	0	1
TMBIM1	4.38E-172	0.650317296	0.534	0.179	8.50E-168	0	1
GOLIM4	3.12E-159	0.649769863	0.627	0.251	6.06E-155	0	1
VDAC2	4.71E-132	0.649458056	0.844	0.463	9.14E-128	0	1
ETHE1	4.87E-181	0.646724767	0.712	0.282	9.44E-177	0	1
SPATS2L	5.64E-195	0.645380834	0.636	0.23	1.09E-190	0	1
IER3	5.83E-184	0.643370481	0.734	0.289	1.13E-179	0	1
HES1	1.59E-175	0.643220565	0.684	0.256	3.09E-171	0	1
HLA-DRB5	0	0.642814064	0.315	0.016	0	0	1
PPIA	1.05E-133	0.639806656	0.946	0.786	2.03E-129	0	1
PRSS8	1.16E-165	0.637377867	0.524	0.173	2.25E-161	0	1
ACTN4	9.21E-154	0.637044407	0.768	0.347	1.79E-149	0	1
GOLM1	3.24E-219	0.636742961	0.631	0.197	6.29E-215	0	1
RPL22	1.34E-197	0.635655634	0.973	0.923	2.61E-193	0	1
CYB5A	1.46E-177	0.635499668	0.672	0.261	2.84E-173	0	1
COX5A	8.79E-131	0.63479561	0.874	0.518	1.71E-126	0	1
AGR2	1.71E-267	0.633473626	0.927	0.394	3.31E-263	0	1
NDUFB2	1.80E-121	0.630979815	0.88	0.572	3.49E-117	0	1
MGST3	3.05E-135	0.629924759	0.835	0.432	5.92E-131	0	1
ECHS1	7.92E-153	0.629045538	0.735	0.343	1.54E-148	0	1
GMDS	3.57E-178	0.62848514	0.672	0.257	6.92E-174	0	1
PKP3	1.48E-209	0.627352366	0.542	0.165	2.86E-205	0	1
DEK	2.44E-123	0.625688593	0.847	0.48	4.74E-119	0	1
TXNDC17	8.63E-154	0.625431515	0.756	0.353	1.67E-149	0	1
CHCHD2	9.36E-137	0.623118798	0.947	0.749	1.82E-132	0	1
MYC	1.29E-192	0.622339336	0.55	0.169	2.50E-188	0	1
SERPINB6	3.86E-161	0.621334674	0.768	0.346	7.49E-157	0	1
SNRPE	2.55E-128	0.62063463	0.857	0.48	4.95E-124	0	1
CAMTA1	1.38E-149	0.619601032	0.746	0.349	2.67E-145	0	1
RHOBTB3	1.40E-215	0.618552988	0.585	0.176	2.71E-211	0	1
C1orf21	2.11E-229	0.617841863	0.553	0.157	4.09E-225	0	1
ATP5F1C	4.05E-118	0.613594336	0.844	0.501	7.86E-114	0	1
ERBB3	3.39E-197	0.61044188	0.597	0.198	6.58E-193	0	1
UQCRC1	2.32E-141	0.609555862	0.758	0.365	4.49E-137	0	1
LIMA1	8.93E-168	0.608673211	0.715	0.278	1.73E-163	0	1

PPP1R1B	1.40E-185	0.607944469	0.676	0.234	2.72E-181	0	1
NENF	1.91E-152	0.607882699	0.752	0.354	3.71E-148	0	1
PLP2	1.87E-125	0.606894807	0.804	0.443	3.63E-121	0	1
RPL24	1.66E-201	0.603706396	0.974	0.941	3.21E-197	0	1
CENPW	4.11E-158	0.603695817	0.326	0.076	7.98E-154	0	1
HEBP2	2.34E-155	0.603593854	0.728	0.319	4.53E-151	0	1
CDC42EP5	3.44E-210	0.603482422	0.579	0.172	6.67E-206	0	1
VIL1	1.75E-180	0.601898397	0.588	0.203	3.39E-176	0	1
SLC25A3	2.71E-114	0.601043042	0.893	0.582	5.26E-110	0	1
PTPRF	1.14E-195	0.59770995	0.561	0.181	2.21E-191	0	1
CMBL	1.89E-202	0.597684331	0.504	0.143	3.67E-198	0	1
ALDOA	2.36E-160	0.597174563	0.579	0.219	4.57E-156	0	1
CTTN	8.39E-169	0.590357227	0.582	0.21	1.63E-164	0	1
ATP1A1	7.59E-132	0.589851503	0.844	0.441	1.47E-127	0	1
MZT2B	8.62E-114	0.588612297	0.867	0.532	1.67E-109	0	1
TUBA1C	5.69E-153	0.581991111	0.591	0.233	1.10E-148	0	1
NUPR1	1.86E-228	0.581557702	0.7	0.22	3.61E-224	0	1
BNIP3	1.52E-178	0.580554915	0.396	0.094	2.94E-174	0	1
MUC13	1.11E-80	0.578949593	0.431	0.188	2.15E-76	0	1
RPL31	4.10E-184	0.578927107	0.989	0.977	7.96E-180	0	1
CKS1B	2.21E-111	0.578841152	0.454	0.179	4.29E-107	0	1
CLIC1	1.29E-128	0.576929782	0.965	0.789	2.49E-124	0	1
GCSH	8.75E-185	0.575552478	0.553	0.185	1.70E-180	0	1
CYC1	5.29E-112	0.572225654	0.78	0.409	1.03E-107	0	1
NDUFA10	5.35E-151	0.572135867	0.666	0.286	1.04E-146	0	1
SLC9A3R2	2.76E-215	0.57187938	0.484	0.127	5.35E-211	0	1
NDUFS6	3.26E-103	0.571764817	0.81	0.475	6.32E-99	0	1
SELENOW	5.04E-116	0.571538807	0.786	0.414	9.78E-112	0	1
AC004556.	6.53E-247	0.56993055	0.483	0.11	1.27E-242	0	1
COX4I1	8.83E-144	0.568509025	0.96	0.853	1.71E-139	0	1
DSG2	8.26E-151	0.566258658	0.602	0.223	1.60E-146	0	1
BOLA3	2.92E-160	0.565951671	0.542	0.195	5.67E-156	0	1
ATP5F1B	5.08E-103	0.564996271	0.873	0.531	9.85E-99	0	1
ATP5PO	2.44E-104	0.564776495	0.873	0.576	4.73E-100	0	1
S100A13	8.70E-181	0.564052222	0.521	0.165	1.69E-176	0	1
ANXA4	8.29E-223	0.563033502	0.795	0.316	1.61E-218	0	1
RPS16	6.50E-184	0.561924033	0.982	0.982	1.26E-179	0	1
AP1M2	1.12E-178	0.561872416	0.534	0.176	2.18E-174	0	1
EI24	2.38E-148	0.559461247	0.681	0.29	4.61E-144	0	1
HSBP1	2.18E-122	0.559334145	0.819	0.435	4.23E-118	0	1
EBPL	5.81E-146	0.558340216	0.615	0.253	1.13E-141	0	1
TM9SF3	4.75E-142	0.557072822	0.739	0.332	9.21E-138	0	1
SYAP1	5.82E-139	0.556636196	0.69	0.314	1.13E-134	0	1
PAFAH1B3	5.73E-167	0.556087575	0.6	0.221	1.11E-162	0	1
MPC1	1.44E-142	0.556086683	0.628	0.269	2.79E-138	0	1
POLR2L	2.12E-88	0.552892317	0.88	0.635	4.11E-84	0	1
PDCD6	4.19E-124	0.55007881	0.788	0.405	8.12E-120	0	1
ETS2	7.97E-162	0.549543306	0.647	0.246	1.55E-157	0	1
KLF5	6.37E-175	0.548860631	0.714	0.259	1.24E-170	0	1
SLC25A6	2.74E-111	0.548179694	0.954	0.809	5.32E-107	0	1
TPM1	7.29E-186	0.54789227	0.817	0.32	1.41E-181	0	1

BICDL2	1.57E-176	0.545148043	0.48	0.147	3.04E-172	0	1
APLP2	1.36E-129	0.544396751	0.764	0.361	2.65E-125	0	1
CDX1	2.42E-165	0.544396472	0.59	0.208	4.69E-161	0	1
PSMB1	4.30E-92	0.543845158	0.866	0.564	8.34E-88	0	1
NDUFAF2	3.62E-183	0.543371144	0.537	0.176	7.03E-179	0	1
POLR1D	3.44E-111	0.53762088	0.793	0.429	6.67E-107	0	1
PPP1R14B	1.24E-147	0.535884928	0.657	0.274	2.41E-143	0	1
VSNL1	1.26E-188	0.53582324	0.354	0.076	2.45E-184	0	1
ADAM15	1.84E-208	0.535473966	0.45	0.114	3.57E-204	0	1
MGAT4B	6.24E-171	0.535470059	0.46	0.141	1.21E-166	0	1
GNG12	2.19E-182	0.534285176	0.46	0.133	4.25E-178	0	1
METTL26	5.21E-145	0.533475806	0.69	0.311	1.01E-140	0	1
S100A14	3.00E-163	0.532516113	0.615	0.218	5.81E-159	0	1
NDUFB10	4.11E-102	0.531941013	0.819	0.486	7.97E-98	0	1
PTTG1IP	1.01E-114	0.529022484	0.594	0.266	1.95E-110	0	1
ZNF703	6.73E-170	0.529009578	0.444	0.131	1.31E-165	0	1
SNHG8	9.51E-114	0.528862969	0.796	0.434	1.84E-109	0	1
POF1B	7.94E-172	0.528056938	0.443	0.13	1.54E-167	0	1
ACP1	3.42E-119	0.526737251	0.732	0.375	6.63E-115	0	1
RAN	1.34E-81	0.526003917	0.878	0.6	2.60E-77	0	1
ECI1	3.23E-162	0.523930771	0.47	0.155	6.26E-158	0	1
TAGLN2	4.11E-98	0.523853408	0.842	0.509	7.97E-94	0	1
GGCT	7.49E-127	0.52235168	0.562	0.233	1.45E-122	0	1
HOXB8	8.21E-181	0.519765809	0.37	0.086	1.59E-176	0	1
ATP5PB	2.33E-98	0.519318798	0.813	0.472	4.53E-94	0	1
HADH	5.64E-160	0.518257858	0.489	0.164	1.09E-155	0	1
ARF5	3.42E-121	0.518176794	0.734	0.363	6.64E-117	0	1
RPL35A	1.37E-162	0.517873768	0.992	0.984	2.66E-158	0	1
FAM162A	7.69E-143	0.51735366	0.743	0.327	1.49E-138	0	1
TFRC	3.79E-159	0.517327531	0.558	0.197	7.36E-155	0	1
MRPL14	6.07E-130	0.517191151	0.724	0.341	1.18E-125	0	1
PSMA7	3.78E-85	0.516714992	0.915	0.665	7.34E-81	0	1
CHMP4B	2.17E-120	0.515868634	0.78	0.375	4.20E-116	0	1
KIF9	1.90E-183	0.514716821	0.383	0.094	3.69E-179	0	1
CD59	3.78E-138	0.513045515	0.588	0.24	7.32E-134	0	1
PLPP2	2.56E-148	0.51288118	0.536	0.191	4.96E-144	0	1
PTTG1	2.17E-55	0.512393769	0.33	0.145	4.20E-51	0	1
HOXB6	1.20E-171	0.511994785	0.398	0.105	2.32E-167	0	1
PPIC	3.27E-166	0.510381022	0.555	0.186	6.34E-162	0	1
GFPT1	1.61E-135	0.510333026	0.584	0.239	3.11E-131	0	1
MT-ND4L	2.89E-98	0.509997101	0.825	0.483	5.61E-94	0	1
HIGD2A	1.02E-97	0.509537435	0.864	0.529	1.98E-93	0	1
LAD1	3.55E-155	0.509462264	0.498	0.168	6.89E-151	0	1
ITM2C	4.20E-156	0.507466258	0.829	0.4	8.15E-152	0	1
MYH14	1.77E-153	0.507359781	0.469	0.154	3.42E-149	0	1
AURKAIP1	5.56E-100	0.506925094	0.803	0.464	1.08E-95	0	1
KCNN4	3.88E-183	0.506891441	0.477	0.135	7.52E-179	0	1
NDUFA12	4.07E-105	0.506337049	0.754	0.403	7.89E-101	0	1
NHP2	1.03E-107	0.506283471	0.744	0.388	2.01E-103	0	1
RPL29	9.59E-149	0.506101495	0.986	0.961	1.86E-144	0	1
SORL1	1.92E-123	0.504787602	0.514	0.198	3.72E-119	0	1

RPL14	7.15E-151	0.50380682	0.978	0.965	1.39E-146	0	1
ICA1	4.83E-189	0.503163926	0.498	0.147	9.36E-185	0	1
NFIA	2.28E-175	0.503110518	0.496	0.153	4.41E-171	0	1
SPINK4	4.85E-116	4.99473869	0.395	0.117	9.41E-112	4	2
FCGBP	0	3.827795204	0.579	0.08	0	4	2
REG4	5.60E-286	3.763049904	0.479	0.072	1.09E-281	4	2
MUC2	0	3.101749685	0.457	0.038	0	4	2
SOX4	2.88E-204	2.586273748	0.815	0.334	5.59E-200	4	2
HES6	0	2.528317292	0.703	0.142	0	4	2
WFDC2	1.22E-241	2.297374869	0.495	0.092	2.36E-237	4	2
HEPACAM	0	2.202880825	0.658	0.008	0	4	2
ANXA13	0	2.155042496	0.594	0.067	0	4	2
KLK1	1.52E-228	2.069818296	0.542	0.128	2.95E-224	4	2
KLK12	0	1.976819007	0.51	0.015	0	4	2
IGFBP2	6.54E-132	1.770769707	0.471	0.136	1.27E-127	4	2
SPINK1	2.01E-227	1.769445652	0.867	0.336	3.89E-223	4	2
STARD10	5.21E-232	1.480981128	0.792	0.282	1.01E-227	4	2
LRRC26	0	1.479108526	0.562	0.057	0	4	2
SERPINA1	8.90E-153	1.471569391	0.52	0.143	1.73E-148	4	2
GSN	1.02E-116	1.442181945	0.572	0.219	1.98E-112	4	2
ST6GALNA	6.26E-198	1.424733851	0.453	0.095	1.21E-193	4	2
KLK11	0	1.391371827	0.509	0.046	0	4	2
PHGR1	6.69E-152	1.356783272	0.825	0.373	1.30E-147	4	2
RASD1	1.62E-267	1.275216235	0.334	0.03	3.14E-263	4	2
IL13RA1	2.85E-206	1.264796831	0.576	0.153	5.52E-202	4	2
STMN1	1.23E-118	1.231464094	0.67	0.287	2.38E-114	4	2
ATOH1	0	1.223273251	0.434	0.005	0	4	2
ID1	7.53E-24	1.10855534	0.373	0.221	1.46E-19	4	2
RASSF6	4.01E-133	1.10256474	0.439	0.12	7.78E-129	4	2
CADPS	8.47E-175	1.060638358	0.343	0.056	1.64E-170	4	2
KIAA1324	2.00E-261	1.054934143	0.536	0.098	3.89E-257	4	2
TFF1	5.22E-68	1.049979043	0.431	0.174	1.01E-63	4	2
RAB26	0	1.0484279	0.417	0.011	0	4	2
EGR1	1.04E-127	1.039656998	0.75	0.323	2.01E-123	4	2
FAM213A	9.87E-188	1.01449069	0.583	0.163	1.91E-183	4	2
AZGP1	5.29E-110	1.008246904	0.462	0.151	1.03E-105	4	2
GADD45G	8.29E-103	1.006814505	0.302	0.07	1.61E-98	4	2
H3F3A	2.03E-137	1.001517753	0.985	0.92	3.94E-133	4	2
NREP	5.99E-157	0.985863547	0.311	0.051	1.16E-152	4	2
HSPA2	1.09E-299	0.979334075	0.32	0.022	2.12E-295	4	2
SELENOM	6.00E-96	0.964760684	0.486	0.172	1.16E-91	4	2
COX6C	2.96E-90	0.950861482	0.952	0.712	5.74E-86	4	2
SEC11C	8.47E-79	0.947102302	0.65	0.317	1.64E-74	4	2
TUBA1A	1.99E-30	0.931133466	0.458	0.264	3.86E-26	4	2
TSPAN13	7.47E-142	0.923803203	0.718	0.299	1.45E-137	4	2
PHLDA1	8.43E-119	0.886120243	0.58	0.199	1.64E-114	4	2
FOXA3	1.12E-168	0.84934976	0.484	0.124	2.18E-164	4	2
FABP2	1.67E-91	0.829244112	0.319	0.088	3.24E-87	4	2
ITGA6	2.49E-81	0.824276047	0.539	0.232	4.84E-77	4	2
MARCKS	3.39E-80	0.817119845	0.733	0.379	6.57E-76	4	2
BCAS1	1.39E-108	0.812861124	0.33	0.081	2.70E-104	4	2

BIK	1.59E-92	0.810186121	0.428	0.149	3.08E-88	4	2
PROX1	1.39E-221	0.790294875	0.389	0.055	2.69E-217	4	2
FOXA2	4.35E-135	0.788687952	0.39	0.095	8.43E-131	4	2
RAB2A	7.78E-90	0.776338557	0.802	0.47	1.51E-85	4	2
COLCA1	0	0.775864461	0.358	0.028	0	4	2
KLF4	2.74E-73	0.768874264	0.484	0.203	5.31E-69	4	2
ASRGL1	7.87E-164	0.764892268	0.488	0.125	1.53E-159	4	2
MGLL	2.74E-109	0.763197241	0.508	0.181	5.32E-105	4	2
TPD52	1.41E-84	0.762082725	0.778	0.438	2.73E-80	4	2
OCIAD2	8.14E-96	0.756541803	0.848	0.482	1.58E-91	4	2
NEURL1	0	0.753953417	0.363	0.009	0	4	2
ASPH	6.96E-93	0.750928551	0.587	0.252	1.35E-88	4	2
CAMK2N1	9.55E-94	0.749309977	0.659	0.283	1.85E-89	4	2
HMGB3	3.65E-77	0.74685427	0.43	0.168	7.07E-73	4	2
MAP7	1.22E-166	0.74312237	0.536	0.15	2.37E-162	4	2
CCDC14	8.68E-116	0.738146378	0.568	0.203	1.68E-111	4	2
CD63	7.24E-91	0.737479483	0.958	0.708	1.41E-86	4	2
CREB3L1	1.53E-90	0.732459125	0.36	0.114	2.97E-86	4	2
PTMS	5.76E-91	0.721740447	0.681	0.316	1.12E-86	4	2
CKAP4	4.60E-112	0.719774747	0.491	0.171	8.93E-108	4	2
RAP1GAP	1.10E-305	0.714742776	0.343	0.026	2.13E-301	4	2
KCNQ1OT	6.99E-61	0.714092436	0.394	0.159	1.36E-56	4	2
KDELR1	3.48E-81	0.705741318	0.707	0.384	6.75E-77	4	2
SMIM31	1.29E-129	0.70012599	0.435	0.119	2.50E-125	4	2
SPDEF	8.06E-257	0.699880631	0.3	0.024	1.56E-252	4	2
ARFGEF3	5.98E-170	0.694720556	0.427	0.093	1.16E-165	4	2
CDH1	5.20E-101	0.691348182	0.575	0.228	1.01E-96	4	2
CHD7	1.17E-85	0.688102845	0.443	0.162	2.27E-81	4	2
SMAD9	4.24E-279	0.683027056	0.308	0.022	8.22E-275	4	2
ZKSCAN1	1.07E-99	0.680167776	0.647	0.274	2.08E-95	4	2
SLC44A1	9.50E-95	0.677122703	0.644	0.29	1.84E-90	4	2
CLRN3	3.74E-139	0.675396718	0.425	0.111	7.25E-135	4	2
RAB13	7.01E-116	0.672785185	0.594	0.226	1.36E-111	4	2
EHF	8.66E-112	0.672656601	0.503	0.173	1.68E-107	4	2
PLEKHB1	1.55E-195	0.672547698	0.372	0.058	3.00E-191	4	2
FNBP1L	5.01E-93	0.66656225	0.466	0.171	9.72E-89	4	2
RNASE1	3.09E-74	0.665302866	0.367	0.127	6.00E-70	4	2
GSE1	7.93E-163	0.664092568	0.462	0.109	1.54E-158	4	2
C4orf48	1.18E-57	0.664007491	0.7	0.39	2.30E-53	4	2
PDXDC1	9.90E-129	0.66035379	0.598	0.215	1.92E-124	4	2
LGALS3BP	1.57E-79	0.652547837	0.632	0.281	3.04E-75	4	2
AKAP9	1.35E-76	0.646579333	0.815	0.479	2.63E-72	4	2
HOXA10	8.46E-112	0.645448967	0.46	0.148	1.64E-107	4	2
H1F0	1.15E-82	0.643492485	0.516	0.21	2.22E-78	4	2
DSP	4.49E-84	0.633761344	0.569	0.236	8.70E-80	4	2
TRIB1	2.89E-49	0.633534386	0.43	0.202	5.60E-45	4	2
PRUNE2	3.50E-159	0.63252283	0.378	0.073	6.79E-155	4	2
ZFHX3	1.51E-64	0.627578882	0.353	0.132	2.92E-60	4	2
SOX9	3.05E-66	0.626353487	0.477	0.207	5.91E-62	4	2
HPCAL1	3.10E-88	0.615700209	0.544	0.224	6.01E-84	4	2
VPS13A	4.76E-66	0.614377353	0.445	0.185	9.23E-62	4	2

DYNLT1	1.39E-71	0.611515441	0.818	0.481	2.69E-67	4	2
RTN4	8.55E-46	0.604174743	0.78	0.503	1.66E-41	4	2
H2AFY	4.78E-67	0.600244233	0.8	0.46	9.28E-63	4	2
IFI6	3.09E-64	0.598789255	0.575	0.262	6.00E-60	4	2
INSR	8.33E-63	0.598659142	0.349	0.13	1.62E-58	4	2
NECTIN1	4.54E-160	0.593782252	0.371	0.072	8.81E-156	4	2
CERS6	6.38E-70	0.593036019	0.451	0.188	1.24E-65	4	2
ITPR2	3.17E-48	0.58302307	0.343	0.138	6.14E-44	4	2
PRDX2	5.29E-55	0.577723103	0.778	0.474	1.03E-50	4	2
CNPY2	4.04E-74	0.576838835	0.726	0.378	7.84E-70	4	2
HSD11B2	1.82E-108	0.566936478	0.354	0.093	3.54E-104	4	2
CNN3	2.25E-69	0.561597261	0.44	0.177	4.36E-65	4	2
TMEM258	4.07E-55	0.560742724	0.922	0.647	7.90E-51	4	2
MLXIP	2.00E-66	0.558909816	0.527	0.244	3.88E-62	4	2
BMP4	4.34E-67	0.556785938	0.315	0.1	8.42E-63	4	2
CDH17	5.95E-61	0.555748992	0.566	0.264	1.15E-56	4	2
CBFA2T2	5.65E-99	0.555021358	0.337	0.087	1.10E-94	4	2
CCDC112	1.16E-111	0.553475251	0.427	0.126	2.25E-107	4	2
DYRK4	9.18E-130	0.5506783	0.342	0.073	1.78E-125	4	2
ATF3	8.28E-26	0.550470796	0.43	0.251	1.61E-21	4	2
ANKS4B	2.06E-116	0.548113223	0.323	0.074	4.00E-112	4	2
PTS	3.65E-90	0.544488708	0.505	0.195	7.07E-86	4	2
CD9	2.34E-61	0.543941895	0.777	0.415	4.53E-57	4	2
PRDX4	4.78E-46	0.53994575	0.609	0.341	9.27E-42	4	2
TSEN34	2.40E-80	0.538346664	0.569	0.251	4.66E-76	4	2
CASP6	3.75E-83	0.535404805	0.458	0.174	7.27E-79	4	2
SMARCC1	3.07E-60	0.533405924	0.538	0.264	5.95E-56	4	2
ARHGAP3[illegible]	5.66E-99	0.530296759	0.391	0.12	1.10E-94	4	2
BTG2	7.97E-24	0.529857921	0.722	0.512	1.55E-19	4	2
ZNF428	7.12E-53	0.527173506	0.491	0.239	1.38E-48	4	2
MGST2	1.05E-74	0.523620605	0.677	0.315	2.03E-70	4	2
CRACR2B	1.30E-78	0.522754023	0.399	0.143	2.53E-74	4	2
HOXA9	4.71E-94	0.522523361	0.372	0.112	9.13E-90	4	2
BAG1	1.11E-71	0.520697172	0.691	0.346	2.15E-67	4	2
CXXC5	4.20E-75	0.519762226	0.524	0.225	8.15E-71	4	2
STAP2	2.60E-85	0.517579287	0.471	0.178	5.04E-81	4	2
ENY2	7.09E-32	0.515758415	0.692	0.459	1.38E-27	4	2
TSC22D1	6.92E-64	0.515381955	0.705	0.349	1.34E-59	4	2
TMEM98	6.12E-77	0.508702142	0.438	0.164	1.19E-72	4	2
USH1C	4.42E-72	0.506433419	0.49	0.205	8.58E-68	4	2
SEC61B	2.76E-42	0.504900005	0.882	0.621	5.35E-38	4	2
SCP2	1.87E-58	0.504872922	0.798	0.492	3.63E-54	4	2
PI3	2.53E-159	2.290523758	0.422	0.067	4.91E-155	7	3
SLPI	1.16E-171	2.175008425	0.699	0.193	2.25E-167	7	3
NEAT1	2.27E-98	2.174210399	0.893	0.802	4.40E-94	7	3
PRSS3	2.60E-227	1.968375551	0.862	0.271	5.04E-223	7	3
FTH1	5.03E-129	1.656494193	0.998	0.981	9.75E-125	7	3
CST3	1.69E-141	1.607800473	0.953	0.558	3.27E-137	7	3
HMGA1	1.16E-89	1.55707021	0.781	0.381	2.24E-85	7	3
PRAC1	0	1.553872937	0.65	0.073	0	7	3
PRAP1	4.72E-210	1.48072522	0.674	0.135	9.15E-206	7	3

VEGFA	1.67E-168	1.44504145	0.699	0.182	3.24E-164	7	3
TRIM31	1.46E-241	1.430745667	0.587	0.09	2.83E-237	7	3
TPT1	1.99E-141	1.426732318	0.998	0.984	3.86E-137	7	3
KRT19	2.04E-124	1.407649474	0.906	0.412	3.96E-120	7	3
LMO7	1.21E-133	1.353408386	0.632	0.18	2.36E-129	7	3
CLTA	8.43E-125	1.332581244	0.884	0.504	1.63E-120	7	3
RPS6	1.84E-89	1.240669142	0.987	0.993	3.57E-85	7	3
CES2	2.10E-92	1.177953521	0.554	0.18	4.07E-88	7	3
RRBP1	8.93E-82	1.146253046	0.766	0.382	1.73E-77	7	3
RPS18	5.35E-98	1.135477299	1	0.996	1.04E-93	7	3
HNF4A	3.81E-118	1.133221365	0.56	0.158	7.39E-114	7	3
DST	7.39E-104	1.116475158	0.569	0.179	1.43E-99	7	3
ABHD11	3.92E-129	1.109162615	0.65	0.2	7.61E-125	7	3
CKB	1.37E-32	1.085714821	0.638	0.352	2.65E-28	7	3
UFM1	2.08E-94	1.047094328	0.779	0.37	4.03E-90	7	3
RNF43	2.35E-126	1.044026474	0.616	0.176	4.55E-122	7	3
DDAH2	4.23E-77	1.030620839	0.703	0.341	8.20E-73	7	3
LY6G6F-LY	0	1.029876556	0.442	0.014	0	7	3
RND3	1.85E-148	1.022481604	0.554	0.124	3.58E-144	7	3
PCK1	5.70E-127	1.003594438	0.375	0.063	1.11E-122	7	3
SAP18	1.13E-79	0.994786393	0.888	0.62	2.19E-75	7	3
RPS10	1.25E-55	0.980344505	0.96	0.956	2.42E-51	7	3
PYGB	8.11E-109	0.976922822	0.569	0.171	1.57E-104	7	3
SEC61G	8.95E-71	0.973470068	0.922	0.66	1.74E-66	7	3
HSPA1A	4.97E-56	0.973218914	0.656	0.291	9.63E-52	7	3
ARHGAP5	4.94E-122	0.966972765	0.65	0.203	9.59E-118	7	3
EFNB2	4.68E-176	0.961097256	0.536	0.102	9.09E-172	7	3
EFNA1	4.62E-118	0.959723427	0.545	0.144	8.97E-114	7	3
HOXB7	5.58E-133	0.959416119	0.638	0.186	1.08E-128	7	3
EPS8	9.47E-133	0.94599125	0.645	0.184	1.84E-128	7	3
KDELR2	1.94E-60	0.93895925	0.819	0.502	3.77E-56	7	3
SLC6A8	1.19E-78	0.932893208	0.359	0.089	2.30E-74	7	3
BRI3	5.47E-72	0.923715406	0.694	0.334	1.06E-67	7	3
SELENOP	1.76E-142	0.914951684	0.587	0.133	3.41E-138	7	3
SLC44A4	4.69E-100	0.908902264	0.623	0.209	9.09E-96	7	3
CHMP5	6.46E-64	0.907371991	0.719	0.36	1.25E-59	7	3
MISP	1.30E-111	0.891032282	0.621	0.191	2.51E-107	7	3
B4GALT1	1.78E-62	0.888291917	0.549	0.214	3.45E-58	7	3
DNAJC15	2.11E-69	0.871383143	0.795	0.421	4.10E-65	7	3
JUP	4.05E-114	0.869154089	0.609	0.186	7.86E-110	7	3
FABP1	8.22E-14	0.840502306	0.431	0.275	1.60E-09	7	3
C6orf132	5.31E-130	0.834738399	0.509	0.12	1.03E-125	7	3
YWHAE	4.63E-61	0.832653254	0.85	0.545	8.99E-57	7	3
SBDS	3.65E-55	0.830635649	0.721	0.39	7.07E-51	7	3
PHKG1	5.01E-82	0.830207309	0.406	0.107	9.71E-78	7	3
POMP	2.03E-54	0.829884067	0.875	0.655	3.94E-50	7	3
NFIB	3.18E-91	0.82113204	0.527	0.164	6.16E-87	7	3
NPC2	3.51E-62	0.81521268	0.848	0.51	6.80E-58	7	3
CBWD5	1.05E-90	0.81352354	0.618	0.216	2.04E-86	7	3
FAM177A1	9.51E-65	0.802275827	0.759	0.383	1.85E-60	7	3
GAPDH	1.38E-69	0.797252044	0.984	0.916	2.68E-65	7	3

MRPL57	3.99E-62	0.790717126	0.763	0.429	7.74E-58	7	3
TAX1BP3	7.23E-79	0.790382013	0.623	0.244	1.40E-74	7	3
C6orf48	1.23E-50	0.790212469	0.781	0.482	2.39E-46	7	3
YBX3	6.89E-82	0.787482835	0.75	0.332	1.34E-77	7	3
TAX1BP1	3.60E-44	0.78729468	0.812	0.543	6.98E-40	7	3
DDR1	5.75E-96	0.786195688	0.54	0.167	1.12E-91	7	3
HOXB9	1.29E-113	0.763960119	0.538	0.146	2.50E-109	7	3
KRT20	8.12E-77	0.759894165	0.48	0.141	1.57E-72	7	3
AMN	5.84E-99	0.759696487	0.52	0.144	1.13E-94	7	3
MYO6	1.40E-72	0.755505333	0.636	0.253	2.72E-68	7	3
GNAQ	9.27E-89	0.754617172	0.571	0.196	1.80E-84	7	3
TMEM45B	8.14E-96	0.744088386	0.6	0.194	1.58E-91	7	3
LDLR	1.80E-62	0.740667473	0.473	0.168	3.49E-58	7	3
DGKH	1.93E-145	0.739247482	0.496	0.097	3.74E-141	7	3
NUDT4	2.47E-63	0.734432765	0.701	0.333	4.79E-59	7	3
BRD2	7.81E-58	0.734369275	0.712	0.355	1.51E-53	7	3
SLC25A5	1.54E-36	0.729742436	0.864	0.617	2.99E-32	7	3
DDIT3	1.06E-43	0.727704804	0.449	0.178	2.07E-39	7	3
SPTSSA	3.88E-77	0.727170967	0.661	0.268	7.53E-73	7	3
MGST1	1.77E-51	0.725004247	0.625	0.286	3.43E-47	7	3
CFDP1	7.29E-56	0.724978025	0.685	0.332	1.41E-51	7	3
UBE2R2	1.27E-81	0.723346223	0.598	0.223	2.47E-77	7	3
TNRC18	1.80E-154	0.723217718	0.525	0.105	3.49E-150	7	3
NDFIP2	7.90E-87	0.717099493	0.616	0.223	1.53E-82	7	3
TP53	3.80E-105	0.712525052	0.455	0.107	7.37E-101	7	3
HSP90AB1	1.48E-35	0.711489086	0.931	0.775	2.87E-31	7	3
ROMO1	1.11E-49	0.710932161	0.833	0.511	2.16E-45	7	3
HIST1H2A(	6.84E-76	0.710891232	0.522	0.174	1.33E-71	7	3
STOML2	1.33E-51	0.709567939	0.67	0.352	2.58E-47	7	3
SDC4	3.61E-62	0.69467709	0.516	0.194	7.00E-58	7	3
KLF3	5.57E-73	0.69363597	0.616	0.235	1.08E-68	7	3
TNNC2	1.39E-130	0.692547508	0.379	0.063	2.70E-126	7	3
HMGB1	2.51E-33	0.692178818	0.949	0.877	4.87E-29	7	3
EZR	1.86E-34	0.691196042	0.752	0.458	3.60E-30	7	3
TSTD1	1.42E-44	0.688473572	0.781	0.463	2.76E-40	7	3
LAMB3	7.12E-100	0.687645765	0.455	0.114	1.38E-95	7	3
TPM4	1.72E-41	0.68735744	0.761	0.443	3.34E-37	7	3
SULT2B1	2.69E-124	0.686252684	0.379	0.067	5.21E-120	7	3
COMMD6	4.19E-49	0.685905828	0.915	0.758	8.13E-45	7	3
MTIF3	2.16E-50	0.685158693	0.688	0.362	4.18E-46	7	3
CD2AP	1.53E-76	0.683876866	0.6	0.227	2.97E-72	7	3
NEU1	7.22E-76	0.67610819	0.558	0.2	1.40E-71	7	3
YIPF3	4.02E-65	0.675901822	0.621	0.264	7.79E-61	7	3
BROX	6.24E-74	0.674192606	0.592	0.221	1.21E-69	7	3
DYNLRB1	7.02E-33	0.673654524	0.746	0.473	1.36E-28	7	3
MUC3A	4.66E-112	0.669495364	0.491	0.121	9.04E-108	7	3
RPL8	6.46E-53	0.668841074	0.998	0.99	1.25E-48	7	3
RPL7	7.57E-36	0.667537665	0.987	0.986	1.47E-31	7	3
MIF	1.54E-24	0.667330527	0.897	0.681	2.99E-20	7	3
MLXIPL	1.04E-123	0.665428304	0.368	0.064	2.02E-119	7	3
ADM	5.95E-76	0.663620926	0.386	0.099	1.15E-71	7	3

PGRMC1	1.98E-49	0.663290836	0.603	0.28	3.84E-45	7	3
CCND1	3.21E-55	0.660706557	0.558	0.227	6.22E-51	7	3
INAVA	1.10E-107	0.660077872	0.444	0.105	2.13E-103	7	3
CTNND1	1.62E-74	0.658687728	0.605	0.234	3.14E-70	7	3
PLGRKT	7.74E-68	0.65521434	0.585	0.232	1.50E-63	7	3
HNMT	5.67E-66	0.652701597	0.531	0.197	1.10E-61	7	3
SELENOH	1.27E-37	0.651679089	0.853	0.622	2.47E-33	7	3
ZFAND5	5.17E-55	0.648516638	0.685	0.331	1.00E-50	7	3
CEACAM1	1.76E-53	0.648192968	0.359	0.115	3.42E-49	7	3
ACOT13	1.34E-57	0.645467678	0.52	0.207	2.59E-53	7	3
EFNA3	1.26E-133	0.643866455	0.411	0.074	2.44E-129	7	3
SUGT1	1.76E-62	0.643033801	0.641	0.279	3.41E-58	7	3
SUMF2	1.17E-49	0.642437532	0.567	0.252	2.26E-45	7	3
AL161431.	0	0.640695944	0.342	0.009	0	7	3
SPTBN1	7.27E-70	0.639832661	0.612	0.238	1.41E-65	7	3
EIF5	1.59E-34	0.639344576	0.754	0.48	3.09E-30	7	3
SCIN	4.46E-209	0.637558237	0.35	0.032	8.65E-205	7	3
KNOP1	4.45E-53	0.636353038	0.516	0.206	8.64E-49	7	3
H2AFJ	6.54E-53	0.636207203	0.728	0.368	1.27E-48	7	3
TIMM10	1.05E-57	0.635830783	0.558	0.234	2.04E-53	7	3
SLIRP	2.22E-38	0.633516364	0.763	0.454	4.31E-34	7	3
HSPH1	2.38E-49	0.623252825	0.641	0.317	4.61E-45	7	3
MRPL52	4.27E-41	0.619896631	0.723	0.417	8.28E-37	7	3
CYP3A5	4.42E-49	0.61980055	0.493	0.202	8.58E-45	7	3
EMP1	2.27E-74	0.617358872	0.312	0.07	4.40E-70	7	3
PLOD2	5.17E-84	0.615688292	0.312	0.064	1.00E-79	7	3
MALL	1.27E-69	0.605183847	0.458	0.14	2.47E-65	7	3
RPL39	7.94E-11	0.605051607	0.993	0.991	1.54E-06	7	3
CSNK2B	5.72E-41	0.604878314	0.757	0.449	1.11E-36	7	3
PLEC	9.82E-58	0.603466589	0.504	0.189	1.90E-53	7	3
MVP	1.81E-52	0.602212272	0.618	0.281	3.52E-48	7	3
CALM1	7.15E-36	0.602091133	0.906	0.755	1.39E-31	7	3
ATP10B	3.90E-62	0.599772339	0.442	0.15	7.57E-58	7	3
MIDN	3.69E-41	0.59777261	0.587	0.286	7.16E-37	7	3
NEK3	3.77E-108	0.596925166	0.413	0.089	7.31E-104	7	3
MMP24OS	9.44E-56	0.596081493	0.618	0.275	1.83E-51	7	3
LAMP1	1.33E-36	0.592880497	0.708	0.396	2.58E-32	7	3
HOXB3	1.48E-128	0.591720945	0.406	0.074	2.88E-124	7	3
TM9SF2	3.55E-51	0.588163565	0.609	0.296	6.88E-47	7	3
STK24	9.89E-81	0.588074411	0.567	0.19	1.92E-76	7	3
FOXC1	1.38E-302	0.584224427	0.326	0.015	2.67E-298	7	3
MPST	1.28E-46	0.583968821	0.67	0.339	2.48E-42	7	3
IGBP1	2.27E-42	0.582585507	0.632	0.326	4.41E-38	7	3
ATP5MPL	3.74E-34	0.57933404	0.906	0.715	7.26E-30	7	3
TMEM63A	1.29E-51	0.579174397	0.496	0.199	2.51E-47	7	3
NUCKS1	1.57E-28	0.57739598	0.741	0.486	3.05E-24	7	3
LLGL2	1.08E-70	0.577388417	0.52	0.181	2.10E-66	7	3
CAPN1	4.53E-39	0.573428391	0.605	0.309	8.78E-35	7	3
ZG16B	1.46E-58	0.572318598	0.478	0.173	2.83E-54	7	3
PAPOLA	1.71E-36	0.57042397	0.786	0.517	3.32E-32	7	3
ERO1A	2.39E-53	0.569375317	0.556	0.231	4.63E-49	7	3

GSKIP	1.39E-77	0.564898417	0.536	0.175	2.70E-73	7	3
RAB4A	1.48E-40	0.564347248	0.618	0.327	2.87E-36	7	3
IRS2	2.62E-92	0.564278176	0.475	0.126	5.08E-88	7	3
CBWD1	3.24E-53	0.564173276	0.58	0.257	6.28E-49	7	3
FARP1	7.62E-170	0.56396556	0.404	0.055	1.48E-165	7	3
LINC01133	1.36E-54	0.563825548	0.344	0.104	2.63E-50	7	3
INTS6	2.66E-49	0.561273815	0.536	0.22	5.16E-45	7	3
C6orf222	6.88E-144	0.561189965	0.342	0.046	1.33E-139	7	3
MAPK13	3.70E-47	0.560992418	0.516	0.23	7.17E-43	7	3
SERPINE2	3.15E-163	0.557830295	0.353	0.042	6.11E-159	7	3
TPI1	3.94E-27	0.557726908	0.906	0.72	7.65E-23	7	3
IFT172	1.70E-55	0.556749926	0.46	0.168	3.29E-51	7	3
ANG	2.26E-59	0.555518327	0.44	0.151	4.38E-55	7	3
PLS1	4.72E-57	0.554629088	0.473	0.172	9.16E-53	7	3
SLC40A1	3.79E-46	0.553719765	0.438	0.173	7.36E-42	7	3
SLC39A4	8.69E-50	0.553532223	0.58	0.254	1.69E-45	7	3
TSPAN6	2.74E-63	0.551657711	0.493	0.177	5.31E-59	7	3
SMC5	1.52E-65	0.5512726	0.578	0.221	2.96E-61	7	3
GDF15	1.38E-63	0.550395497	0.502	0.168	2.68E-59	7	3
MACC1	2.25E-71	0.546267526	0.384	0.105	4.37E-67	7	3
EGLN3	2.29E-38	0.546027057	0.33	0.12	4.44E-34	7	3
RIOK3	2.76E-58	0.545337078	0.533	0.206	5.36E-54	7	3
PKP2	8.91E-74	0.544283208	0.46	0.142	1.73E-69	7	3
EIF3B	9.66E-64	0.542470255	0.507	0.184	1.87E-59	7	3
PRPF6	1.85E-59	0.541982375	0.598	0.249	3.60E-55	7	3
TMEM14C	1.94E-40	0.541927276	0.737	0.416	3.76E-36	7	3
NUDT15	4.14E-84	0.539340608	0.406	0.104	8.03E-80	7	3
AC103702.1	1.42E-52	0.538097865	0.498	0.192	2.76E-48	7	3
EIF6	1.48E-38	0.537860472	0.741	0.417	2.86E-34	7	3
TOMM20	6.44E-36	0.53734566	0.746	0.466	1.25E-31	7	3
VPS51	2.53E-49	0.536926843	0.549	0.246	4.91E-45	7	3
GUK1	1.70E-28	0.535728576	0.882	0.681	3.30E-24	7	3
TM4SF5	2.08E-125	0.534233546	0.342	0.053	4.03E-121	7	3
BAIAP2L1	1.37E-72	0.529963002	0.415	0.119	2.65E-68	7	3
ALG5	1.65E-41	0.529922444	0.587	0.288	3.20E-37	7	3
PRR15	3.75E-79	0.524329106	0.44	0.126	7.28E-75	7	3
DMAC1	3.96E-37	0.523819142	0.638	0.334	7.68E-33	7	3
AKAP1	7.85E-69	0.520951014	0.489	0.163	1.52E-64	7	3
MAPK3	1.74E-72	0.520902488	0.496	0.161	3.38E-68	7	3
DSC2	5.40E-45	0.519653841	0.5	0.214	1.05E-40	7	3
CYB5B	2.50E-38	0.519040152	0.598	0.304	4.85E-34	7	3
DNAJA1	2.36E-25	0.518630002	0.75	0.515	4.58E-21	7	3
ATP6V1D	1.92E-46	0.515622933	0.531	0.23	3.73E-42	7	3
SUN1	1.29E-64	0.51492591	0.444	0.143	2.50E-60	7	3
PAWR	2.15E-47	0.512946379	0.556	0.243	4.17E-43	7	3
CAPN8	1.29E-86	0.512456062	0.364	0.083	2.50E-82	7	3
AK3	5.03E-50	0.511926342	0.603	0.264	9.76E-46	7	3
KCNK1	3.29E-106	0.511565131	0.391	0.08	6.38E-102	7	3
EPPK1	6.05E-76	0.511557795	0.346	0.084	1.17E-71	7	3
LSM5	2.12E-25	0.509800921	0.721	0.46	4.11E-21	7	3
EBAG9	8.04E-56	0.50915078	0.518	0.199	1.56E-51	7	3

ERGIC3	2.07E-32	0.509037293	0.75	0.472	4.02E-28	7	3
PIP5K1B	3.39E-78	0.50866769	0.402	0.107	6.57E-74	7	3
BLCAP	3.45E-43	0.508141574	0.487	0.209	6.69E-39	7	3
BCL2L1	6.06E-53	0.506595019	0.449	0.162	1.18E-48	7	3
SQLE	1.68E-19	0.504807061	0.379	0.202	3.26E-15	7	3
MMP7	0	2.978511109	0.833	0.099	0	9	4
ANPEP	0	2.348930215	0.694	0.056	0	9	4
SCD	2.28E-196	1.960886151	0.74	0.172	4.42E-192	9	4
C8orf33	8.48E-181	1.915141918	0.798	0.229	1.64E-176	9	4
PLAC8	7.20E-163	1.729548907	0.823	0.23	1.40E-158	9	4
MYL9	2.46E-180	1.622682486	0.336	0.032	4.78E-176	9	4
LGALS2	7.55E-221	1.563112328	0.679	0.115	1.46E-216	9	4
FDPS	4.88E-112	1.528351773	0.801	0.335	9.46E-108	9	4
PKM	2.04E-141	1.45972352	0.985	0.565	3.96E-137	9	4
PGK1	5.58E-113	1.379840578	0.967	0.586	1.08E-108	9	4
FAM13A	3.80E-136	1.364311003	0.561	0.129	7.38E-132	9	4
ENO1	1.21E-104	1.342980425	0.955	0.639	2.35E-100	9	4
PTGR1	2.63E-237	1.313139897	0.699	0.122	5.11E-233	9	4
PLA2G2A	4.40E-66	1.292805079	0.447	0.13	8.54E-62	9	4
SLC11A2	3.15E-136	1.290876238	0.566	0.131	6.11E-132	9	4
IDH2	2.01E-111	1.285231963	0.884	0.421	3.90E-107	9	4
C15orf48	5.11E-139	1.258435719	0.917	0.342	9.91E-135	9	4
GLO1	1.54E-128	1.253683059	0.828	0.315	2.98E-124	9	4
MRLN	0	1.240142208	0.513	0.012	0	9	4
RBP2	0	1.22339191	0.333	0.006	0	9	4
P4HB	1.40E-97	1.220328232	0.924	0.509	2.71E-93	9	4
TUBA1B	9.89E-35	1.209928089	0.811	0.531	1.92E-30	9	4
FBXO2	1.44E-285	1.208445853	0.566	0.059	2.80E-281	9	4
EBP	2.45E-110	1.195431657	0.75	0.279	4.75E-106	9	4
CFD	1.03E-150	1.183755252	0.604	0.126	2.00E-146	9	4
PGAM1	4.94E-103	1.155850566	0.919	0.482	9.58E-99	9	4
JPT1	1.60E-104	1.152185783	0.914	0.418	3.10E-100	9	4
RAB5IF	1.57E-95	1.151658613	0.879	0.418	3.04E-91	9	4
PLBD1	7.56E-164	1.146993064	0.758	0.21	1.47E-159	9	4
GPX4	2.91E-93	1.141737901	0.965	0.602	5.64E-89	9	4
ACAT2	8.83E-135	1.138754917	0.601	0.146	1.71E-130	9	4
ATP5ME	2.20E-87	1.11823384	0.965	0.683	4.26E-83	9	4
MYL12B	1.04E-109	1.11619835	0.99	0.765	2.01E-105	9	4
PCLAF	2.70E-103	1.109031603	0.399	0.08	5.23E-99	9	4
SMIM24	2.01E-126	1.093926169	0.5	0.105	3.91E-122	9	4
TSPAN3	1.03E-126	1.081578257	0.808	0.286	1.99E-122	9	4
H2AFZ	2.49E-46	1.07797151	0.909	0.652	4.84E-42	9	4
EIF2S2	7.10E-83	1.073414933	0.902	0.521	1.38E-78	9	4
FTL	4.64E-109	1.056547588	0.995	0.967	9.00E-105	9	4
RARRES1	6.31E-132	1.046376189	0.391	0.062	1.22E-127	9	4
BSG	5.33E-80	1.041534948	0.927	0.518	1.03E-75	9	4
PDXK	2.74E-136	1.040704879	0.77	0.244	5.32E-132	9	4
NAA20	2.25E-47	1.037710733	0.619	0.281	4.36E-43	9	4
TDP2	1.08E-107	1.033936189	0.753	0.265	2.09E-103	9	4
QPRT	1.30E-112	1.02844026	0.707	0.232	2.53E-108	9	4
DDT	4.66E-104	1.02687446	0.912	0.443	9.04E-100	9	4

EIF5A	3.71E-87	1.018549361	0.864	0.403	7.19E-83	9	4
PPARG	2.54E-196	0.998527773	0.621	0.113	4.92E-192	9	4
HSD17B2	5.25E-156	0.997110996	0.333	0.037	1.02E-151	9	4
GSTM3	4.09E-186	0.987048709	0.581	0.101	7.92E-182	9	4
MSMO1	5.16E-122	0.987009912	0.598	0.153	1.00E-117	9	4
RAB11A	1.91E-102	0.965780752	0.904	0.398	3.70E-98	9	4
PRELID1	7.14E-90	0.962517794	0.957	0.568	1.38E-85	9	4
ZNF706	6.74E-81	0.954621656	0.912	0.521	1.31E-76	9	4
GPI	8.30E-122	0.946561027	0.811	0.281	1.61E-117	9	4
CNIH4	2.37E-118	0.945528089	0.826	0.301	4.60E-114	9	4
PRELID3B	4.61E-96	0.942709658	0.768	0.293	8.95E-92	9	4
C4orf3	2.60E-64	0.935499247	0.932	0.632	5.05E-60	9	4
AKR1B10	0	0.93309979	0.48	0.024	0	9	4
C17orf78	0	0.931022223	0.323	0.004	0	9	4
GCHFR	3.59E-122	0.929591507	0.687	0.195	6.97E-118	9	4
IDH1	1.45E-129	0.925233293	0.753	0.232	2.82E-125	9	4
NDUFB9	2.31E-72	0.923226855	0.914	0.528	4.48E-68	9	4
CISD1	7.96E-107	0.898812012	0.763	0.271	1.54E-102	9	4
PPP1R14D	9.13E-183	0.895215589	0.619	0.115	1.77E-178	9	4
RNF186	1.43E-92	0.892709215	0.432	0.101	2.77E-88	9	4
SPINT1	2.14E-104	0.887037052	0.712	0.236	4.14E-100	9	4
PRR15L	2.63E-134	0.866943209	0.654	0.168	5.10E-130	9	4
AKAP7	3.26E-168	0.857608903	0.515	0.083	6.33E-164	9	4
RANBP1	2.16E-40	0.855306441	0.79	0.457	4.19E-36	9	4
GGH	4.80E-97	0.847072958	0.591	0.177	9.31E-93	9	4
TSPO	2.14E-73	0.843457182	0.947	0.538	4.14E-69	9	4
COMT	1.36E-93	0.839391648	0.803	0.305	2.64E-89	9	4
HSD17B12	1.30E-85	0.834516611	0.773	0.307	2.52E-81	9	4
YWHAB	3.98E-52	0.834142793	0.932	0.652	7.72E-48	9	4
RPLP0	1.13E-66	0.833051967	0.98	0.935	2.20E-62	9	4
MUC17	0	0.830719908	0.414	0.024	0	9	4
PPP2R1A	6.26E-86	0.816498945	0.828	0.345	1.21E-81	9	4
ANKRD9	3.02E-198	0.815738314	0.568	0.09	5.87E-194	9	4
NDUFA6	1.22E-79	0.815361469	0.854	0.396	2.38E-75	9	4
CHMP2B	3.57E-95	0.810139596	0.775	0.297	6.93E-91	9	4
RABL6	2.41E-97	0.808817676	0.687	0.234	4.68E-93	9	4
TIMM13	1.41E-70	0.801630718	0.831	0.398	2.74E-66	9	4
CEBPD	1.82E-79	0.801454524	0.692	0.259	3.53E-75	9	4
ANKRD37	1.07E-26	0.801374956	0.313	0.128	2.07E-22	9	4
SERPINB1	2.18E-61	0.800459834	0.871	0.465	4.23E-57	9	4
CHP1	1.78E-78	0.79405392	0.727	0.29	3.45E-74	9	4
ACTG1	9.91E-54	0.791906342	0.99	0.91	1.92E-49	9	4
LACTB2	1.51E-139	0.78590143	0.646	0.158	2.92E-135	9	4
YBX1	5.88E-49	0.785309026	0.98	0.84	1.14E-44	9	4
LSM4	2.12E-61	0.784397048	0.79	0.391	4.11E-57	9	4
CCNG1	3.49E-76	0.783068446	0.78	0.333	6.76E-72	9	4
MVD	1.09E-105	0.781231534	0.525	0.132	2.12E-101	9	4
PSMB5	8.42E-87	0.772670297	0.826	0.346	1.63E-82	9	4
PHB	3.52E-71	0.771812135	0.818	0.377	6.82E-67	9	4
AKR1C3	1.10E-62	0.771104093	0.465	0.149	2.14E-58	9	4
TMPRSS2	3.25E-108	0.770607614	0.571	0.152	6.31E-104	9	4

IDI1	3.14E-51	0.768096623	0.621	0.276	6.09E-47	9	4
DHCR7	3.12E-107	0.760615758	0.429	0.09	6.06E-103	9	4
SLC16A3	7.39E-82	0.756119481	0.624	0.21	1.43E-77	9	4
PSAT1	2.51E-167	0.755280609	0.419	0.056	4.87E-163	9	4
WDR1	4.36E-75	0.749737559	0.831	0.375	8.45E-71	9	4
HMGCS1	1.14E-79	0.747656792	0.5	0.147	2.21E-75	9	4
ATP5F1E	1.94E-34	0.746548268	0.992	0.927	3.77E-30	9	4
GCNT3	1.52E-133	0.744584451	0.399	0.064	2.95E-129	9	4
MTCH2	6.29E-77	0.743568999	0.755	0.313	1.22E-72	9	4
CFL1	5.24E-72	0.73870815	1	0.913	1.02E-67	9	4
PRDX6	2.47E-33	0.736398351	0.78	0.431	4.79E-29	9	4
SDCBP	3.46E-62	0.736296129	0.896	0.462	6.72E-58	9	4
GSTM4	4.11E-160	0.73515632	0.487	0.079	7.98E-156	9	4
SMAGP	4.62E-100	0.731989602	0.697	0.228	8.96E-96	9	4
CHMP4C	1.73E-144	0.728588076	0.619	0.141	3.35E-140	9	4
C10orf99	1.75E-92	0.728575388	0.485	0.122	3.39E-88	9	4
TPMT	2.19E-132	0.726982776	0.705	0.187	4.26E-128	9	4
CPS1	0	0.725128218	0.336	0.011	0	9	4
UQCR10	2.83E-50	0.722909545	0.967	0.616	5.49E-46	9	4
CEBPG	9.34E-96	0.722707324	0.654	0.209	1.81E-91	9	4
CYP2S1	2.62E-172	0.721059042	0.439	0.06	5.08E-168	9	4
F3	3.81E-82	0.720554293	0.343	0.072	7.38E-78	9	4
SMS	8.56E-69	0.719029711	0.788	0.355	1.66E-64	9	4
FLNB	2.58E-70	0.717212524	0.485	0.155	5.00E-66	9	4
NME1	7.22E-48	0.715471626	0.621	0.289	1.40E-43	9	4
RHEB	2.10E-59	0.715147709	0.818	0.417	4.07E-55	9	4
CKS2	1.11E-36	0.711435874	0.538	0.254	2.16E-32	9	4
PMEPA1	3.77E-51	0.711381079	0.49	0.184	7.31E-47	9	4
UQCRC2	1.20E-52	0.710487711	0.843	0.442	2.33E-48	9	4
GNAS	2.69E-53	0.708309906	0.912	0.556	5.22E-49	9	4
STAU1	1.35E-69	0.705294196	0.78	0.337	2.62E-65	9	4
CCT5	9.37E-62	0.705283449	0.76	0.348	1.82E-57	9	4
RPN2	3.86E-51	0.703139257	0.785	0.412	7.48E-47	9	4
AHCY	2.69E-62	0.701739308	0.568	0.219	5.23E-58	9	4
PDIA3	3.77E-39	0.701729153	0.886	0.599	7.31E-35	9	4
CALM2	7.96E-37	0.700503767	0.965	0.834	1.54E-32	9	4
ELOC	3.60E-59	0.694959052	0.861	0.454	6.99E-55	9	4
AL121761.2	7.24E-240	0.6946598	0.346	0.023	1.40E-235	9	4
MRPL13	4.18E-73	0.692666408	0.783	0.325	8.10E-69	9	4
PRELID2	7.51E-160	0.689702404	0.535	0.097	1.46E-155	9	4
MTHFD2	5.22E-60	0.687569721	0.596	0.232	1.01E-55	9	4
EIF3H	4.07E-52	0.679835263	0.939	0.623	7.90E-48	9	4
NANS	2.35E-72	0.679513603	0.76	0.32	4.56E-68	9	4
USP53	1.05E-56	0.674743031	0.472	0.166	2.04E-52	9	4
PSME2	1.21E-29	0.672985779	0.856	0.56	2.34E-25	9	4
ATP5PD	2.89E-46	0.669650741	0.861	0.487	5.60E-42	9	4
CIB1	2.46E-54	0.668933119	0.944	0.568	4.76E-50	9	4
CTNNA1	1.11E-77	0.66888176	0.722	0.279	2.16E-73	9	4
SLC25A39	1.90E-65	0.667673443	0.699	0.293	3.69E-61	9	4
TYMS	1.00E-165	0.667393484	0.386	0.047	1.95E-161	9	4
GIPC1	7.75E-90	0.663972425	0.667	0.223	1.50E-85	9	4

NPM1	1.09E-28	0.66364111	0.96	0.862	2.12E-24	9	4
SLC35A3	6.50E-82	0.662388872	0.682	0.249	1.26E-77	9	4
ATP5F1D	1.03E-41	0.658525343	0.944	0.635	2.00E-37	9	4
MYL6	8.71E-53	0.658330788	0.99	0.909	1.69E-48	9	4
TSPAN15	1.85E-108	0.651686756	0.591	0.159	3.60E-104	9	4
MZT2A	7.31E-57	0.650439057	0.907	0.485	1.42E-52	9	4
CISD2	1.35E-72	0.649878274	0.745	0.304	2.61E-68	9	4
PRR13	1.01E-43	0.646534683	0.96	0.623	1.97E-39	9	4
DDIT4	8.79E-26	0.644914114	0.54	0.277	1.70E-21	9	4
PLCB3	2.57E-140	0.644021788	0.525	0.103	4.99E-136	9	4
C3orf85	5.13E-242	0.643471832	0.374	0.028	9.94E-238	9	4
CMAS	5.10E-80	0.642887471	0.644	0.221	9.90E-76	9	4
ETFB	1.68E-67	0.642668113	0.851	0.391	3.26E-63	9	4
HIGD1A	6.14E-69	0.642287173	0.753	0.31	1.19E-64	9	4
SDCBP2	8.83E-105	0.641489759	0.457	0.099	1.71E-100	9	4
TUBB	6.74E-24	0.638673302	0.775	0.477	1.31E-19	9	4
INSIG1	1.83E-54	0.637369526	0.525	0.192	3.56E-50	9	4
ADRM1	4.86E-55	0.636828203	0.763	0.352	9.42E-51	9	4
UBE2V2	2.61E-58	0.633937716	0.73	0.326	5.07E-54	9	4
FKBP4	4.86E-91	0.633856802	0.646	0.205	9.42E-87	9	4
TMEM97	4.09E-119	0.632635943	0.48	0.101	7.93E-115	9	4
PTGES3	6.05E-35	0.632001328	0.927	0.664	1.17E-30	9	4
LYZ	4.25E-91	0.631916412	0.891	0.391	8.24E-87	9	4
ME1	1.45E-168	0.630609256	0.452	0.065	2.82E-164	9	4
HEPH	9.51E-85	0.629331493	0.485	0.133	1.84E-80	9	4
PHPT1	2.25E-56	0.62890611	0.859	0.447	4.37E-52	9	4
OLA1	2.19E-60	0.627630133	0.725	0.315	4.24E-56	9	4
UQCR11	1.26E-44	0.625662737	0.972	0.731	2.44E-40	9	4
SLC25A1	2.66E-106	0.625510009	0.558	0.143	5.17E-102	9	4
HSD17B11	1.25E-52	0.622863339	0.795	0.401	2.42E-48	9	4
TMBIM6	6.68E-32	0.620969492	0.899	0.708	1.30E-27	9	4
CHCHD10	3.15E-45	0.620956916	0.886	0.497	6.11E-41	9	4
ERG28	3.28E-78	0.619737212	0.596	0.197	6.35E-74	9	4
PRSS22	1.03E-94	0.618121941	0.414	0.09	2.00E-90	9	4
TECR	3.97E-57	0.618091511	0.768	0.37	7.70E-53	9	4
GSTO1	7.02E-51	0.617955436	0.793	0.393	1.36E-46	9	4
KPNA2	5.76E-39	0.616673396	0.331	0.111	1.12E-34	9	4
AP3S1	5.74E-49	0.61565608	0.841	0.419	1.11E-44	9	4
PPP1R16A	1.17E-89	0.61495926	0.588	0.179	2.26E-85	9	4
CYP2W1	6.22E-86	0.614325479	0.333	0.065	1.21E-81	9	4
GALNT1	3.23E-69	0.613395778	0.591	0.211	6.26E-65	9	4
RPS27L	1.41E-39	0.611386865	0.949	0.635	2.73E-35	9	4
ADI1	1.10E-57	0.610284505	0.684	0.298	2.13E-53	9	4
YWHAZ	2.48E-42	0.610234306	0.947	0.706	4.80E-38	9	4
AL133453.	2.31E-100	0.609423775	0.412	0.083	4.49E-96	9	4
HDHD3	1.29E-77	0.608313316	0.583	0.194	2.50E-73	9	4
DYNC1I2	2.71E-52	0.603322612	0.788	0.379	5.25E-48	9	4
GID8	2.99E-79	0.602749955	0.692	0.247	5.80E-75	9	4
PDIA6	1.19E-33	0.601877969	0.841	0.552	2.30E-29	9	4
ALKBH7	8.84E-57	0.599947636	0.705	0.315	1.72E-52	9	4
CHCHD3	7.79E-78	0.599653398	0.604	0.203	1.51E-73	9	4

ATF4	5.57E-41	0.596675429	0.758	0.404	1.08E-36	9	4
GTF3C6	7.11E-52	0.596189083	0.732	0.35	1.38E-47	9	4
CENPV	8.88E-96	0.595619027	0.593	0.172	1.72E-91	9	4
SRI	3.98E-45	0.594636053	0.922	0.522	7.71E-41	9	4
ANAPC11	6.01E-50	0.593621012	0.864	0.462	1.17E-45	9	4
HDAC1	3.16E-57	0.59144366	0.753	0.343	6.14E-53	9	4
SEPHS2	7.52E-53	0.591153977	0.679	0.298	1.46E-48	9	4
TMCC1	8.52E-133	0.590487194	0.437	0.075	1.65E-128	9	4
SLC2A1	1.36E-66	0.589682576	0.404	0.111	2.64E-62	9	4
CDS1	3.67E-99	0.589437152	0.523	0.135	7.12E-95	9	4
TMEM92	2.30E-159	0.586630238	0.379	0.048	4.46E-155	9	4
PFKP	5.96E-110	0.585757043	0.561	0.137	1.16E-105	9	4
ACOT7	1.51E-99	0.584323391	0.497	0.121	2.93E-95	9	4
SNORC	8.10E-116	0.580294508	0.482	0.102	1.57E-111	9	4
STXBP6	2.89E-72	0.580075095	0.528	0.169	5.60E-68	9	4
CCT6A	8.74E-46	0.579217074	0.783	0.402	1.70E-41	9	4
HMGN2	8.32E-15	0.57886359	0.886	0.683	1.61E-10	9	4
FDFT1	1.52E-54	0.578025003	0.616	0.257	2.96E-50	9	4
PFKL	3.98E-67	0.577877411	0.669	0.262	7.72E-63	9	4
WDR45B	4.10E-66	0.576663982	0.586	0.21	7.94E-62	9	4
PXMP2	9.00E-90	0.576239878	0.533	0.148	1.75E-85	9	4
ETFA	5.23E-63	0.575526045	0.763	0.333	1.01E-58	9	4
ERRFI1	1.39E-41	0.575306934	0.328	0.105	2.69E-37	9	4
EIF3E	1.26E-30	0.572446844	0.942	0.75	2.44E-26	9	4
HSPA9	3.43E-45	0.572130223	0.717	0.352	6.66E-41	9	4
TCEAL9	6.83E-66	0.571043476	0.515	0.173	1.33E-61	9	4
GPA33	9.79E-91	0.567568301	0.475	0.12	1.90E-86	9	4
PON2	1.50E-73	0.566171601	0.634	0.22	2.90E-69	9	4
SRSF9	1.86E-44	0.565264432	0.876	0.502	3.60E-40	9	4
CMPK1	1.15E-38	0.564122562	0.922	0.575	2.23E-34	9	4
BID	9.57E-68	0.562655665	0.616	0.226	1.86E-63	9	4
AC020656.	1.72E-63	0.560867888	0.477	0.151	3.33E-59	9	4
TK1	1.86E-83	0.560642429	0.316	0.06	3.60E-79	9	4
HEBP1	2.40E-84	0.560163497	0.492	0.136	4.66E-80	9	4
MORF4L2	4.33E-49	0.560042579	0.775	0.385	8.40E-45	9	4
DDAH1	5.21E-96	0.558140143	0.553	0.152	1.01E-91	9	4
OAT	6.71E-34	0.557459972	0.465	0.201	1.30E-29	9	4
UQCRB	5.60E-38	0.556192631	0.985	0.867	1.09E-33	9	4
ARF4	1.32E-48	0.552922323	0.823	0.42	2.55E-44	9	4
SLC52A2	1.31E-63	0.551597777	0.593	0.221	2.54E-59	9	4
NDUFV2	1.85E-46	0.549599059	0.846	0.452	3.60E-42	9	4
CISD3	1.81E-60	0.549475082	0.735	0.308	3.51E-56	9	4
LAP3	1.75E-63	0.54896755	0.568	0.196	3.39E-59	9	4
DNM2	4.66E-76	0.546739839	0.621	0.211	9.03E-72	9	4
FBP1	4.29E-59	0.546601674	0.503	0.173	8.32E-55	9	4
SNRPD1	2.79E-28	0.545750094	0.722	0.431	5.41E-24	9	4
TRAM1	3.07E-43	0.540488225	0.823	0.434	5.95E-39	9	4
SELENOS	8.92E-37	0.53809626	0.755	0.414	1.73E-32	9	4
CLDN15	3.09E-142	0.53806959	0.442	0.071	6.00E-138	9	4
TM7SF2	1.14E-77	0.535422712	0.437	0.112	2.22E-73	9	4
ATP1B3	2.39E-47	0.533350085	0.783	0.401	4.63E-43	9	4

REEP3	1.47E-67	0.533167266	0.621	0.226	2.85E-63	9	4
P4HA1	1.38E-76	0.531443955	0.52	0.154	2.67E-72	9	4
RTN3	2.24E-48	0.530399528	0.71	0.327	4.34E-44	9	4
NETO2	5.89E-143	0.530170634	0.437	0.07	1.14E-138	9	4
PAICS	4.66E-77	0.529761838	0.52	0.156	9.04E-73	9	4
TFG	2.08E-56	0.529717322	0.641	0.267	4.04E-52	9	4
GLRX5	1.20E-66	0.529580476	0.629	0.234	2.33E-62	9	4
ATP5MC1	4.89E-27	0.52867841	0.737	0.453	9.49E-23	9	4
DHRS11	8.45E-131	0.528652554	0.371	0.055	1.64E-126	9	4
RAB1A	1.92E-44	0.528579488	0.788	0.406	3.73E-40	9	4
CANX	5.46E-35	0.5283753	0.823	0.48	1.06E-30	9	4
CRB3	1.72E-88	0.527320104	0.48	0.125	3.34E-84	9	4
WDR34	7.18E-71	0.527291486	0.457	0.131	1.39E-66	9	4
NUDT14	2.07E-76	0.525829948	0.593	0.189	4.02E-72	9	4
PYCR1	4.95E-76	0.525433464	0.487	0.145	9.60E-72	9	4
CYCS	3.40E-24	0.524381872	0.894	0.629	6.59E-20	9	4
UGDH	1.68E-89	0.523348294	0.548	0.153	3.26E-85	9	4
PPIB	3.64E-25	0.523138784	0.891	0.637	7.07E-21	9	4
MDH1	8.55E-46	0.522196638	0.715	0.333	1.66E-41	9	4
DCTPP1	4.15E-47	0.520212751	0.626	0.272	8.05E-43	9	4
RPS4Y1	3.20E-29	0.518565156	0.768	0.431	6.20E-25	9	4
CBR1	7.68E-41	0.517675797	0.457	0.181	1.49E-36	9	4
SORD	3.31E-74	0.51698456	0.429	0.117	6.42E-70	9	4
PSMA3	4.00E-44	0.515524845	0.758	0.376	7.77E-40	9	4
ERH	2.44E-29	0.515058997	0.851	0.553	4.74E-25	9	4
HNRNPAB	2.15E-42	0.51380618	0.755	0.375	4.17E-38	9	4
CENPX	1.64E-47	0.513610867	0.631	0.272	3.17E-43	9	4
ENC1	4.86E-67	0.513009686	0.452	0.132	9.43E-63	9	4
RCN1	1.44E-60	0.512629754	0.591	0.226	2.79E-56	9	4
NUTF2	1.07E-57	0.512527209	0.71	0.307	2.08E-53	9	4
ABHD12	5.69E-68	0.512254655	0.513	0.163	1.10E-63	9	4
FCGRT	1.00E-40	0.512145475	0.76	0.386	1.94E-36	9	4
SNRPA1	6.99E-73	0.510337273	0.616	0.211	1.36E-68	9	4
DPM1	5.97E-55	0.510029391	0.71	0.301	1.16E-50	9	4
ACAA2	3.23E-60	0.508645804	0.614	0.232	6.27E-56	9	4
LSM3	8.86E-33	0.508499371	0.813	0.461	1.72E-28	9	4
CBLC	3.31E-87	0.507777533	0.47	0.119	6.43E-83	9	4
IL18	1.31E-79	0.507329502	0.419	0.103	2.54E-75	9	4
MYDGF	5.75E-38	0.505723602	0.818	0.449	1.11E-33	9	4
UQCRFS1	3.19E-36	0.505321183	0.806	0.448	6.19E-32	9	4
ARF1	7.80E-35	0.505166482	0.889	0.562	1.51E-30	9	4
LRRC59	1.25E-57	0.504998977	0.606	0.239	2.42E-53	9	4
IMPDH2	1.43E-46	0.504482776	0.664	0.303	2.78E-42	9	4
CAPZA2	4.03E-43	0.504078722	0.831	0.434	7.82E-39	9	4
CTNNB1	4.31E-42	0.50362591	0.77	0.386	8.36E-38	9	4
TBX3	1.83E-87	0.501841179	0.361	0.074	3.56E-83	9	4
DNPH1	3.19E-48	0.501340535	0.737	0.346	6.19E-44	9	4
REEP6	5.10E-102	0.500882732	0.341	0.058	9.89E-98	9	4
CAST	2.79E-33	0.500604986	0.833	0.485	5.42E-29	9	4
NOTUM	0	2.654140167	0.582	0.01	0	10	5
NKD1	0	2.165277502	0.703	0.035	0	10	5

ASPSCR1	5.40E-157	1.875686036	0.558	0.105	1.05E-152	10	5
BAMBI	0	1.783375387	0.568	0.05	0	10	5
TNFRSF11	0	1.783297288	0.461	0.025	0	10	5
PROM1	0	1.535163954	0.732	0.078	0	10	5
MAP3K20	0	1.51874903	0.811	0.097	0	10	5
ALDH1A1	1.13E-239	1.469247237	0.534	0.062	2.19E-235	10	5
PCP4	0	1.344137745	0.353	0.003	0	10	5
AXIN2	0	1.314745407	0.787	0.079	0	10	5
EPHB2	1.34E-241	1.244077748	0.763	0.144	2.60E-237	10	5
CEMIP	0	1.238901968	0.653	0.035	0	10	5
SP5	0	1.220470569	0.563	0.038	0	10	5
MYO10	1.09E-238	1.216648595	0.803	0.168	2.11E-234	10	5
SLC1A5	4.34E-51	1.210791847	0.487	0.189	8.42E-47	10	5
DPEP1	1.30E-122	1.180662678	0.463	0.09	2.53E-118	10	5
GADD45A	3.73E-168	1.178387327	0.695	0.152	7.24E-164	10	5
ID3	1.64E-72	1.134012859	0.639	0.231	3.17E-68	10	5
ABHD2	6.05E-193	1.078059657	0.739	0.156	1.17E-188	10	5
ZFP36L1	1.57E-86	1.074437002	0.926	0.553	3.05E-82	10	5
TCEAL4	2.43E-213	1.052209245	0.753	0.145	4.71E-209	10	5
RFK	3.76E-155	1.047172595	0.75	0.196	7.29E-151	10	5
GTF2I	1.58E-145	1.039635393	0.911	0.317	3.07E-141	10	5
NDUFA4	3.62E-109	1.031736054	0.992	0.734	7.02E-105	10	5
SERINC5	4.11E-135	1.022153211	0.8	0.247	7.98E-131	10	5
PTK7	0	1.02069037	0.611	0.047	0	10	5
ACTN1	6.39E-169	1.017414052	0.797	0.206	1.24E-164	10	5
BST2	3.22E-52	1.00741988	0.768	0.414	6.25E-48	10	5
TGIF1	1.16E-126	0.981534375	0.829	0.277	2.25E-122	10	5
CDCA7	3.30E-189	0.964219519	0.687	0.134	6.39E-185	10	5
LAPTM4A	5.65E-90	0.93316655	0.968	0.505	1.10E-85	10	5
RCN2	1.38E-108	0.930533481	0.813	0.295	2.67E-104	10	5
SORBS2	1.17E-285	0.929394121	0.626	0.074	2.26E-281	10	5
WFS1	0	0.91323428	0.542	0.033	0	10	5
SBSPON	1.42E-285	0.898903947	0.532	0.05	2.75E-281	10	5
TSPAN5	9.95E-219	0.892885628	0.621	0.091	1.93E-214	10	5
IGFBP4	1.89E-81	0.891998521	0.532	0.153	3.67E-77	10	5
ARL4A	4.96E-112	0.88637154	0.832	0.283	9.61E-108	10	5
MRFAP1	7.07E-109	0.883269983	0.9	0.367	1.37E-104	10	5
HACD3	8.19E-172	0.877453935	0.761	0.178	1.59E-167	10	5
SESN1	8.81E-192	0.861664176	0.616	0.103	1.71E-187	10	5
CTSH	1.25E-123	0.860923591	0.787	0.249	2.42E-119	10	5
DAAM1	6.92E-178	0.852490334	0.753	0.165	1.34E-173	10	5
SCG5	4.12E-291	0.845869475	0.429	0.029	7.99E-287	10	5
FBXO21	2.22E-192	0.84461795	0.708	0.137	4.31E-188	10	5
ZNF503	4.92E-264	0.843717068	0.532	0.054	9.54E-260	10	5
C9orf16	1.09E-61	0.837736569	0.918	0.514	2.11E-57	10	5
CD81	2.23E-129	0.833118232	0.821	0.246	4.33E-125	10	5
IRF2BP2	3.39E-101	0.830622668	0.861	0.339	6.57E-97	10	5
PDZD8	1.22E-165	0.827373582	0.75	0.178	2.37E-161	10	5
TTR	6.29E-235	0.826840366	0.358	0.024	1.22E-230	10	5
HDAC2	2.12E-103	0.825371101	0.842	0.326	4.12E-99	10	5
MAP2K6	1.80E-174	0.818109015	0.632	0.119	3.50E-170	10	5

ZNF704	2.01E-243	0.817609545	0.553	0.067	3.89E-239	10	5
OBP2B	0	0.8154339	0.376	0.005	0	10	5
CRNDE	2.18E-227	0.811940625	0.597	0.083	4.23E-223	10	5
APEX1	9.99E-106	0.811905887	0.837	0.31	1.94E-101	10	5
HYAL2	3.02E-155	0.80664067	0.529	0.093	5.86E-151	10	5
CEMIP2	1.26E-155	0.798871472	0.729	0.171	2.44E-151	10	5
TMEM205	3.55E-124	0.79792255	0.818	0.265	6.88E-120	10	5
H2AFY2	0	0.794095165	0.626	0.063	0	10	5
PATJ	1.10E-157	0.789413191	0.692	0.157	2.14E-153	10	5
TMED9	5.61E-73	0.787926507	0.926	0.479	1.09E-68	10	5
RHOB	9.37E-54	0.78653091	0.776	0.364	1.82E-49	10	5
SEZ6L2	5.19E-199	0.786207581	0.595	0.096	1.01E-194	10	5
MSX2	0	0.780624262	0.445	0.027	0	10	5
LGMN	1.24E-149	0.771824838	0.697	0.165	2.40E-145	10	5
SULT1E1	3.93E-277	0.770705744	0.303	0.012	7.63E-273	10	5
SNTB1	2.28E-246	0.766678709	0.513	0.054	4.41E-242	10	5
SKP1	4.37E-74	0.766016969	0.992	0.766	8.47E-70	10	5
QDPR	1.34E-158	0.763401848	0.687	0.154	2.60E-154	10	5
ATP9A	1.11E-215	0.753484678	0.668	0.11	2.15E-211	10	5
ADH5	2.26E-101	0.751598786	0.797	0.282	4.38E-97	10	5
NMNAT3	0	0.751326722	0.545	0.03	0	10	5
NORAD	2.04E-93	0.744563077	0.834	0.318	3.97E-89	10	5
SUPT4H1	6.32E-103	0.738539462	0.813	0.275	1.23E-98	10	5
CHKA	1.02E-187	0.734276746	0.595	0.1	1.97E-183	10	5
DEPTOR	1.08E-246	0.729852516	0.532	0.06	2.09E-242	10	5
LGR5	0	0.727131259	0.458	0.031	0	10	5
TOX3	3.42E-165	0.721936578	0.603	0.116	6.63E-161	10	5
LRPAP1	1.33E-90	0.710191219	0.816	0.312	2.58E-86	10	5
FILIP1L	3.76E-191	0.707629749	0.382	0.038	7.29E-187	10	5
LAMB1	5.25E-274	0.700491419	0.542	0.054	1.02E-269	10	5
ROBO2	0	0.700358324	0.489	0.02	0	10	5
EPHB3	1.85E-176	0.698905971	0.521	0.079	3.59E-172	10	5
VPS28	1.36E-65	0.697392822	0.918	0.502	2.64E-61	10	5
OLFML3	2.99E-280	0.696589534	0.311	0.013	5.79E-276	10	5
ENAH	2.87E-216	0.690488859	0.608	0.089	5.57E-212	10	5
VGLL4	6.37E-138	0.688979521	0.708	0.175	1.23E-133	10	5
CSNK1A1	2.64E-70	0.686463859	0.932	0.478	5.13E-66	10	5
VWA1	5.03E-150	0.685228952	0.487	0.081	9.76E-146	10	5
DHRS7	5.19E-73	0.680196451	0.853	0.371	1.01E-68	10	5
TMEM9	4.28E-159	0.679680036	0.589	0.113	8.30E-155	10	5
PODXL2	4.33E-170	0.677047806	0.571	0.1	8.40E-166	10	5
TTC3	1.40E-65	0.675509973	0.921	0.484	2.72E-61	10	5
MFSD10	1.80E-105	0.672254771	0.795	0.258	3.50E-101	10	5
MFAP2	0	0.671735912	0.432	0.017	0	10	5
HOOK2	9.99E-122	0.670194473	0.671	0.179	1.94E-117	10	5
DMKN	3.69E-213	0.669223002	0.468	0.052	7.17E-209	10	5
DAD1	1.60E-65	0.668340674	0.966	0.553	3.10E-61	10	5
CCT2	8.98E-72	0.665654463	0.832	0.361	1.74E-67	10	5
CDK6	5.27E-121	0.665139874	0.671	0.172	1.02E-116	10	5
PLPP1	1.50E-141	0.662279191	0.482	0.081	2.90E-137	10	5
C19orf48	2.27E-106	0.662065504	0.595	0.156	4.39E-102	10	5

SCML1	2.12E-169	0.6609466	0.553	0.093	4.11E-165	10	5
TAF7	9.84E-55	0.656351452	0.882	0.511	1.91E-50	10	5
CDK4	1.39E-106	0.653299119	0.755	0.239	2.71E-102	10	5
GLUL	1.29E-87	0.649371177	0.832	0.321	2.51E-83	10	5
GMPR	3.98E-117	0.645323559	0.355	0.055	7.71E-113	10	5
BZW2	2.55E-109	0.645261085	0.724	0.218	4.94E-105	10	5
CTSV	1.51E-172	0.644748981	0.382	0.042	2.92E-168	10	5
TMC4	2.14E-143	0.644574625	0.613	0.133	4.16E-139	10	5
CAPS	2.05E-214	0.643099375	0.439	0.044	3.98E-210	10	5
GSTA4	6.24E-233	0.643095283	0.489	0.052	1.21E-228	10	5
PHF14	4.89E-83	0.642931502	0.839	0.327	9.49E-79	10	5
CTSZ	3.04E-67	0.641694285	0.687	0.265	5.90E-63	10	5
SCOC	7.52E-101	0.64000014	0.755	0.247	1.46E-96	10	5
MGAT5	3.02E-152	0.639447188	0.568	0.105	5.87E-148	10	5
EXOSC4	2.57E-122	0.636949636	0.668	0.175	4.98E-118	10	5
SRP14	4.90E-56	0.634001694	1	0.836	9.50E-52	10	5
PLEKHA5	1.15E-163	0.633963213	0.584	0.107	2.24E-159	10	5
ZNRF3	5.79E-277	0.632665962	0.492	0.043	1.12E-272	10	5
MSI2	4.89E-123	0.63011506	0.75	0.209	9.49E-119	10	5
NR2F1	3.87E-297	0.629030978	0.461	0.033	7.51E-293	10	5
SCARB2	8.61E-102	0.626907243	0.734	0.233	1.67E-97	10	5
ILF2	3.36E-74	0.626841198	0.821	0.336	6.53E-70	10	5
PPFIBP1	4.29E-145	0.624173103	0.445	0.069	8.32E-141	10	5
ABCC3	3.42E-82	0.620781415	0.584	0.18	6.63E-78	10	5
ACSL5	1.81E-83	0.616949707	0.692	0.234	3.51E-79	10	5
SLC5A1	1.74E-224	0.615408385	0.495	0.056	3.38E-220	10	5
CACNA1D	8.64E-285	0.614733253	0.474	0.038	1.68E-280	10	5
PAM	5.42E-145	0.614064425	0.484	0.081	1.05E-140	10	5
STRAP	1.30E-75	0.609639502	0.805	0.332	2.53E-71	10	5
SQSTM1	1.12E-59	0.608461484	0.879	0.435	2.18E-55	10	5
EPHX1	2.83E-163	0.607816188	0.453	0.065	5.49E-159	10	5
MEX3A	0	0.606713982	0.442	0.027	0	10	5
SET	4.60E-47	0.605484825	0.966	0.62	8.92E-43	10	5
CDC34	3.88E-174	0.60547588	0.529	0.082	7.53E-170	10	5
UBE2E3	2.14E-106	0.603015803	0.747	0.229	4.16E-102	10	5
SMTN	1.75E-197	0.601064514	0.524	0.071	3.40E-193	10	5
WLS	1.46E-172	0.599457269	0.524	0.082	2.84E-168	10	5
PIGT	1.22E-74	0.598972794	0.763	0.296	2.36E-70	10	5
SDC1	4.77E-92	0.598238611	0.632	0.194	9.26E-88	10	5
MAOB	0	0.597644689	0.303	0.006	0	10	5
PTK2	1.81E-153	0.594336285	0.642	0.133	3.51E-149	10	5
GSTA1	6.81E-160	0.592143627	0.326	0.033	1.32E-155	10	5
SCAND1	2.52E-59	0.591931066	0.913	0.46	4.89E-55	10	5
PDGFA	3.30E-151	0.591918062	0.458	0.07	6.40E-147	10	5
ALDH1B1	1.86E-121	0.590613796	0.492	0.099	3.61E-117	10	5
PCMTD1	7.44E-76	0.589637849	0.758	0.284	1.44E-71	10	5
TRABD2A	2.68E-89	0.589138626	0.584	0.169	5.20E-85	10	5
GDE1	8.59E-91	0.587390369	0.726	0.242	1.67E-86	10	5
SMARCA4	2.87E-108	0.58684951	0.755	0.228	5.56E-104	10	5
TMEM59	7.24E-45	0.583580383	0.966	0.688	1.40E-40	10	5
MANSC1	4.46E-172	0.583073303	0.505	0.077	8.64E-168	10	5

SYNJ2BP	2.79E-110	0.581848044	0.671	0.183	5.40E-106	10	5
ARSE	1.83E-141	0.581766375	0.529	0.1	3.54E-137	10	5
NET1	3.83E-95	0.58059949	0.661	0.199	7.43E-91	10	5
TMED3	3.17E-88	0.579001846	0.771	0.267	6.14E-84	10	5
STT3B	3.50E-71	0.577933314	0.753	0.3	6.80E-67	10	5
PUF60	1.35E-80	0.575191216	0.816	0.311	2.62E-76	10	5
ATP6V0B	6.74E-49	0.574972977	0.905	0.487	1.31E-44	10	5
AFDN	8.39E-95	0.574964695	0.65	0.194	1.63E-90	10	5
SMAD5	4.62E-116	0.573626887	0.597	0.145	8.96E-112	10	5
TSN	1.26E-107	0.572438299	0.684	0.19	2.45E-103	10	5
XRCC5	3.52E-57	0.571316329	0.895	0.448	6.83E-53	10	5
GRINA	1.21E-106	0.569172975	0.624	0.165	2.36E-102	10	5
LEPROT	3.56E-76	0.568745324	0.776	0.292	6.90E-72	10	5
SHTN1	3.26E-118	0.567864838	0.529	0.117	6.32E-114	10	5
HSP90B1	3.16E-44	0.567622393	0.974	0.635	6.14E-40	10	5
ANXA5	9.72E-62	0.567480817	0.842	0.38	1.89E-57	10	5
REPIN1	3.82E-89	0.566665564	0.689	0.224	7.42E-85	10	5
MPZL1	2.72E-103	0.565518749	0.658	0.188	5.27E-99	10	5
KDM1A	3.99E-134	0.563642818	0.566	0.118	7.74E-130	10	5
SSB	7.72E-57	0.562960646	0.887	0.438	1.50E-52	10	5
PAIP1	1.02E-82	0.561662886	0.774	0.283	1.98E-78	10	5
SCCPDH	9.79E-101	0.559211618	0.661	0.188	1.90E-96	10	5
TRAF4	3.04E-116	0.557793358	0.647	0.166	5.89E-112	10	5
RNF128	1.45E-153	0.557491159	0.511	0.086	2.81E-149	10	5
ARL2	3.07E-84	0.557488697	0.761	0.266	5.96E-80	10	5
LPP	8.67E-76	0.557240192	0.774	0.289	1.68E-71	10	5
DACH1	9.88E-133	0.556267427	0.437	0.073	1.92E-128	10	5
POLR2I	1.18E-61	0.556075649	0.818	0.368	2.29E-57	10	5
KLHL23	1.10E-122	0.555167924	0.518	0.107	2.14E-118	10	5
SLC6A6	3.42E-97	0.554719512	0.455	0.1	6.64E-93	10	5
FAM84B	1.26E-182	0.553327257	0.542	0.081	2.44E-178	10	5
PCDHB14	3.74E-291	0.550665115	0.426	0.028	7.25E-287	10	5
PGM2L1	3.76E-79	0.550150767	0.508	0.143	7.29E-75	10	5
GPAA1	6.13E-86	0.548630786	0.729	0.247	1.19E-81	10	5
GAS6	6.83E-86	0.547474236	0.561	0.16	1.32E-81	10	5
TERF1	3.31E-94	0.546693637	0.684	0.209	6.43E-90	10	5
PSMA4	2.24E-49	0.546470174	0.934	0.516	4.34E-45	10	5
COPRS	1.00E-122	0.546292333	0.584	0.133	1.94E-118	10	5
RUNX1	1.26E-88	0.545976184	0.476	0.115	2.44E-84	10	5
TMED10	7.15E-50	0.545092603	0.874	0.46	1.39E-45	10	5
AGO2	4.32E-127	0.544932387	0.682	0.165	8.38E-123	10	5
STK38L	5.69E-115	0.544590658	0.511	0.108	1.10E-110	10	5
TLE2	2.47E-219	0.543720641	0.474	0.052	4.80E-215	10	5
SERPINH1	2.10E-102	0.542967794	0.611	0.16	4.07E-98	10	5
SYNE2	8.71E-67	0.54163327	0.774	0.306	1.69E-62	10	5
FGFR4	4.82E-181	0.536662639	0.508	0.072	9.34E-177	10	5
NAPG	1.26E-127	0.534248746	0.539	0.109	2.44E-123	10	5
HM13	2.08E-75	0.534145562	0.795	0.303	4.03E-71	10	5
GLB1	1.21E-107	0.531880707	0.587	0.147	2.35E-103	10	5
FAM198B	8.67E-184	0.531526442	0.368	0.036	1.68E-179	10	5
DDOST	1.33E-56	0.530854659	0.824	0.381	2.57E-52	10	5

ADGRG1	1.02E-118	0.530064739	0.582	0.135	1.98E-114	10	5
HMGN3	2.96E-57	0.52972062	0.832	0.378	5.73E-53	10	5
APMAP	2.03E-97	0.528681893	0.7	0.208	3.94E-93	10	5
FAT1	1.79E-92	0.527413437	0.518	0.134	3.48E-88	10	5
PRMT1	2.17E-75	0.526299854	0.776	0.289	4.21E-71	10	5
HNRNPA0	3.69E-43	0.525593912	0.895	0.527	7.16E-39	10	5
IP6K2	6.60E-87	0.523621093	0.718	0.24	1.28E-82	10	5
TOP1	2.10E-60	0.520460807	0.797	0.35	4.08E-56	10	5
MRPL51	2.68E-57	0.519763824	0.897	0.435	5.21E-53	10	5
RDH10	4.03E-135	0.517135161	0.426	0.068	7.81E-131	10	5
CBX1	7.72E-113	0.516994265	0.639	0.162	1.50E-108	10	5
C1orf122	1.22E-74	0.516626385	0.632	0.214	2.36E-70	10	5
ITGB8	4.67E-155	0.516248067	0.447	0.064	9.05E-151	10	5
SRPK1	1.32E-58	0.515712338	0.703	0.286	2.55E-54	10	5
PBX1	1.49E-119	0.515482403	0.5	0.101	2.90E-115	10	5
GCC2	2.02E-47	0.513877198	0.9	0.494	3.92E-43	10	5
ALCAM	4.33E-126	0.512181337	0.497	0.096	8.39E-122	10	5
PLEKHA1	1.58E-112	0.511933304	0.658	0.169	3.07E-108	10	5
TP53I3	2.61E-85	0.51100929	0.487	0.128	5.07E-81	10	5
SH3PXD2A	1.89E-113	0.510986692	0.482	0.099	3.66E-109	10	5
ZBTB20	3.73E-66	0.51068218	0.75	0.289	7.24E-62	10	5
SNRPD3	1.26E-66	0.50952479	0.855	0.364	2.45E-62	10	5
CLCN3	5.32E-86	0.508120166	0.642	0.198	1.03E-81	10	5
STARD3	1.09E-73	0.507896349	0.374	0.085	2.12E-69	10	5
NCKAP1	8.07E-95	0.507474503	0.542	0.142	1.57E-90	10	5
EMC2	8.35E-114	0.507049282	0.632	0.156	1.62E-109	10	5
C9orf3	8.56E-103	0.506797987	0.403	0.078	1.66E-98	10	5
NDUFA8	2.71E-72	0.50373493	0.779	0.299	5.25E-68	10	5
PTOV1	2.90E-90	0.503383953	0.666	0.206	5.63E-86	10	5
EIF4G2	5.05E-39	0.503194176	0.955	0.628	9.79E-35	10	5
POLR2F	1.65E-74	0.502891197	0.858	0.342	3.20E-70	10	5
FKBP3	1.22E-69	0.501642278	0.761	0.291	2.36E-65	10	5
BTF3	3.04E-36	0.501504452	0.997	0.867	5.91E-32	10	5
RAB3D	9.30E-129	0.501493179	0.495	0.094	1.80E-124	10	5
LMAN2	1.89E-48	0.501311731	0.884	0.454	3.67E-44	10	5
UGT8	3.91E-143	0.501304489	0.482	0.081	7.59E-139	10	5
CPNE1	1.26E-73	0.50084778	0.742	0.274	2.44E-69	10	5
YAP1	2.80E-122	0.50039865	0.489	0.097	5.43E-118	10	5
