# Supplementary material for: Single-Cell RNA Sequencing Reveals the Role of Epithelial Cell Marker Genes in Predicting the Prognosis of Colorectal Cancer Patients
Source: Dis Markers. 2022 Aug 1;2022:8347125. doi: 10.1155/2022/8347125 (PMC9372514; doi:10.1155/2022/8347125)
Supplement: Supplementary 2 — Table S1: clinical characteristics of the four CRC patients. Table S2: CNV scores of epithelial cell subclusters. Table S3: MEC subclusters of marker genes. Table S4: MECRG signature using the TCGA-CRC training cohort. Table S5: MECRG signature using the GSE17538 validation cohort. [file 8347125.f2.zip › Table S4.pdf]

TableS4 MECRGs signature in TCGA-CRC training set

id	survival time	state	LGALS2	GDE1	MPC1	GNG5	BST2	TPM2	PRELID2	CAPS	CACNA1D	riskScore	risk
TCGA-AA-3511	0.580821918	0	3.807485	6.691154	5.464104	8.55487	7.448894	5.799264	3.775885	2.982107	3.856826	0.348859193	low
TCGA-CM-5864	1.252054795	0	2.881656	5.886878	5.63271	8.628573	6.217215	4.794617	3.730971	3.247117	3.251735	0.913413157	low
TCGA-G4-6320	2.202739726	0	5.239596	6.778319	5.476244	8.712277	5.087023	4.751993	4.242616	4.445011	3.31609	0.594948147	low
TCGA-AA-3846	1.419178082	0	4.419436	7.04178	6.285063	8.594475	5.027386	4.588173	4.18875	3.19042	2.94988	0.282700651	low
TCGA-NH-A6GB	1.304109589	0	4.025493	6.180099	4.714672	8.170237	5.428786	4.490364	4.049477	3.269368	3.285437	0.971230044	low
TCGA-A6-6648	2.098630137	0	4.943689	6.899807	5.199751	8.115458	4.488755	4.540314	4.233506	3.137429	3.652108	0.280654166	low
TCGA-F4-6854	0.043835616	0	4.761769	6.263482	5.265092	8.529817	6.483902	6.234097	3.810679	3.371999	3.763976	0.574021873	low
TCGA-AA-3947	2.750684932	0	3.116881	7.311679	6.467407	8.629098	6.569433	4.445004	4.412234	2.60295	2.831771	0.222341268	low
TCGA-AA-3525	0.002739726	0	6.110559	6.797746	5.847647	8.366632	5.655257	5.174065	4.237648	3.363077	2.836376	0.449618672	low
TCGA-A6-5656	2.742465753	0	3.55782	6.311501	5.433676	8.115035	4.245774	5.223182	5.13785	4.525491	4.243969	0.478356731	low
TCGA-AA-3864	4.416438356	0	2.891905	7.198681	5.934663	8.663952	5.983486	5.235565	4.204499	2.859517	2.52953	0.514312047	low
TCGA-CM-6161	1.252054795	0	5.193646	6.831173	4.864899	8.248579	8.20254	5.13564	4.270466	4.587683	3.661869	0.862543663	low
TCGA-AA-3675	3.920547945	0	2.527324	6.582883	5.87758	8.626899	6.671667	5.272496	3.34428	3.489869	3.39572	0.644949769	low
TCGA-AA-3861	2.504109589	0	3.746759	6.700863	5.995509	8.654672	6.46127	3.595905	4.075112	3.041591	2.87346	0.419057827	low
TCGA-CM-6170	1.252054795	0	3.789704	6.948162	4.970693	8.375979	5.740069	5.358343	4.059642	3.849316	3.951638	0.456852266	low
TCGA-DM-A28E	9.994520548	0	2.39195	6.231803	5.308952	8.36123	4.1347	4.41753	3.858038	3.222654	4.777083	0.223728876	low
TCGA-CM-4752	1.084931507	0	4.855129	6.666178	5.704277	8.005622	6.684277	5.950952	3.73275	3.864019	3.466199	0.640007244	low
TCGA-AZ-6603	2.463013699	1	3.364809	6.602481	5.136867	8.230356	6.712215	6.002489	3.922787	3.682766	3.925395	0.627360807	low
TCGA-AA-3534	2.416438356	0	5.621427	6.640866	4.700556	8.206408	5.119812	5.258226	3.599923	4.029685	3.445257	0.873377307	low
TCGA-AA-3821	0.084931507	0	4.537684	6.95551	5.678886	8.160811	6.644844	5.528956	4.357709	3.809961	2.954592	0.700591814	low
TCGA-AA-A02Y	3.331506849	0	5.974583	6.103116	5.225661	8.468062	8.406668	3.670257	4.049036	2.558363	3.148469	0.481109223	low
TCGA-CM-5860	2.668493151	0	3.506488	6.889284	5.86881	8.614339	8.121468	6.536145	3.828716	3.131593	3.080955	0.607896798	low
TCGA-AA-A024	3.254794521	1	2.610626	6.587945	5.578769	8.554698	5.232534	4.308328	4.147543	3.754932	2.861712	0.949883179	low
TCGA-AY-A71X	1.610958904	0	2.914008	6.632435	5.110226	8.726557	4.418672	4.832034	4.242859	4.340701	3.334911	0.971445393	low
TCGA-AZ-6606	0.978082192	1	3.336283	6.475461	5.841264	8.668725	8.700152	4.320283	4.414195	3.165195	2.829331	0.709540381	low
TCGA-G4-6297	6.865753425	0	3.609274	6.742911	4.664536	8.129731	7.347147	6.183094	3.708159	3.029285	3.405809	0.889489179	low
TCGA-AA-3526	1.589041096	0	3.757025	6.690879	5.13588	8.791641	8.924234	5.727809	4.408531	2.96158	2.903479	0.841023598	low
TCGA-AA-3678	3.917808219	0	6.416198	6.687935	4.991046	8.484967	5.849621	5.148549	4.63836	4.535924	3.419768	0.703463914	low
TCGA-G4-6625	7.649315068	0	5.137342	6.501433	5.629611	8.400285	7.138127	5.606235	3.799602	3.450186	3.45989	0.519733224	low
TCGA-F4-6808	2.805479452	0	4.667518	6.469836	5.085966	8.226856	5.507694	6.535039	3.793586	2.995105	3.314365	0.714711479	low
TCGA-A6-5659	2.536986301	0	2.269723	6.493361	4.888018	7.917009	4.793772	5.889427	4.553938	2.751644	3.602372	0.623864568	low
TCGA-CA-5797	1.049315068	0	6.122101	6.615461	5.465991	8.566657	7.407036	6.615055	4.305095	2.532075	3.583236	0.272575209	low
TCGA-A6-6780	1.676712329	0	2.628279	6.363167	5.636022	8.451783	7.786785	3.484144	4.83088	2.457376	2.305545	0.847931827	low
TCGA-G4-6626	0.002739726	1	2.20208	6.738079	4.975705	8.025003	4.67084	5.718503	4.268606	3.413614	3.524295	0.762397268	low
TCGA-CK-4947	1.463013699	0	6.588815	6.820336	5.354454	8.337422	7.18425	5.022224	4.336792	3.263108	2.877405	0.516411373	low
TCGA-AA-3858	2.589041096	0	4.441582	6.463792	5.169628	8.639038	5.796558	6.007282	3.343281	3.50583	3.291179	0.872248169	low
TCGA-D5-6929	1.117808219	0	4.10209	7.33822	5.382605	8.106536	7.157533	6.593069	4.313615	3.558913	3.306367	0.540389094	low
TCGA-NH-A8F7	1.487671233	0	3.081898	6.481302	5.310407	8.283925	4.181538	4.761204	4.446166	4.016629	3.778844	0.574217343	low
TCGA-AA-3561	1.161643836	0	5.166844	7.025007	7.101323	8.984036	7.130635	5.023595	4.277317	3.347499	2.852953	0.218147542	low
TCGA-AA-3538	2.167123288	0	5.819846	7.123845	5.267117	8.279641	5.20475	5.564053	4.060417	5.429816	3.389848	0.944195019	low
TCGA-AA-A01I	2.583561644	0	3.122679	6.616957	5.690289	8.353808	7.492081	4.660733	4.215101	2.559394	3.508246	0.327590827	low
TCGA-AA-3544	1.167123288	0	5.21873	6.405502	5.660469	9.087597	8.234253	6.126861	3.678265	3.008403	2.550617	0.935839507	low
TCGA-AA-3850	0	1	5.25657	6.62194	6.286934	8.504683	5.949439	5.471337	3.914477	3.698949	2.744719	0.62283952	low
TCGA-AA-3815	2.753424658	0	5.232032	6.877025	5.243034	8.175769	8.201155	5.032708	4.239959	2.726764	2.838586	0.562186598	low
TCGA-AA-3663	0.580821918	0	3.245808	7.181094	5.91681	8.31249	6.048413	4.63316	3.805922	2.88616	2.630681	0.496034713	low
TCGA-DM-A1HB	11.30410959	0	3.66702	6.215261	5.396527	8.501593	8.630561	4.588518	4.477673	2.391867	2.972371	0.63707591	low
TCGA-QG-A5Z2	2.608219178	0	4.184988	6.90102	5.446612	8.316236	6.427965	4.599996	4.247716	4.008874	2.783156	0.937685715	low
TCGA-AA-3986	1.589041096	0	6.734078	6.859418	5.169143	8.506464	5.845811	4.520971	4.024268	4.371298	2.790243	0.884380288	low
TCGA-CM-5868	1.419178082	0	3.489737	6.892771	5.227	8.782849	7.085847	6.336312	3.823454	3.391998	3.022471	0.885965325	low

TCGA-AA-A00L	3.169863014	0	3.768427	6.474125	5.631117	8.679174	4.62241	4.829558	3.946964	3.492896	3.840046	0.341764369	low
TCGA-A6-A567	5.153424658	1	3.799832	6.72988	5.196157	8.482013	4.086931	7.062995	3.929999	3.597696	3.863186	0.504052052	low
TCGA-AA-3681	0.498630137	0	3.632893	6.54162	5.554113	8.407804	7.275948	4.990314	4.468567	3.255806	3.343413	0.539115586	low
TCGA-AA-3543	0.082191781	0	6.432608	6.222861	5.689239	8.739608	8.922021	5.032802	4.224542	2.766081	2.398242	0.781582803	low
TCGA-D5-6533	2.123287671	0	4.0771	6.774478	6.120038	8.335801	5.561736	4.907733	4.906424	3.338185	3.106169	0.358264999	low
TCGA-A6-2675	3.619178082	0	5.264427	6.951776	5.433024	8.182134	6.446447	5.922214	4.048924	3.363289	3.378008	0.441902889	low
TCGA-AA-3819	2.084931507	0	5.229596	6.660481	5.053639	8.919059	4.672671	5.517445	4.315819	3.947562	3.451319	0.564869259	low
TCGA-AA-A03J	3.41369863	0	5.480269	6.04044	5.276447	8.165067	6.997544	5.6678	3.369866	3.855668	3.98251	0.692822825	low
TCGA-G4-6295	0.695890411	0	3.538366	6.833391	4.957598	8.157692	8.711185	6.19124	3.600163	2.757591	3.832815	0.492301418	low
TCGA-AA-3518	0.084931507	0	4.179296	6.414262	5.574427	8.184766	8.578974	4.752126	3.783666	3.065303	3.295481	0.621824073	low
TCGA-AZ-5407	7.350684932	0	4.069507	6.733678	5.694186	8.491378	5.241643	4.376998	4.610097	3.744886	2.772329	0.683586916	low
TCGA-CA-5796	1.032876712	0	4.562349	6.373419	6.131047	8.609589	5.134285	3.675931	4.785332	2.784653	2.572356	0.40907202	low
TCGA-D5-6538	1.42739726	0	3.708372	6.474645	5.305254	7.833841	3.995095	5.098325	3.764123	4.254335	3.665232	0.832210621	low
TCGA-AA-A00E	2.501369863	0	3.146776	7.012138	6.015469	8.42263	9.532102	4.703187	4.052058	2.783078	2.45351	0.674303858	low
TCGA-AA-A00D	1.583561644	0	4.173157	6.898604	5.098631	8.204249	9.540516	5.556458	3.753348	3.498196	3.282234	0.85837323	low
TCGA-AA-3713	1.58630137	0	4.43281	6.464915	5.262596	8.215248	5.385221	5.157256	4.477539	3.016579	3.516007	0.430328859	low
TCGA-AA-3548	2.832876712	0	3.565797	6.975178	6.119746	9.401084	8.52867	5.480079	3.805201	3.884931	2.665345	0.787991953	low
TCGA-G4-6294	2.350684932	1	2.645892	6.447931	5.212892	8.473814	5.294989	4.735409	3.683634	3.464412	3.743685	0.595233164	low
TCGA-CM-6172	0.917808219	0	3.919273	6.930789	5.929953	8.393044	5.10148	4.907239	4.47754	3.620134	3.549949	0.298913791	low
TCGA-AA-A02W	3.416438356	0	2.974422	7.041964	4.962687	8.232582	5.380636	5.533836	4.067798	4.395291	3.899761	0.679826835	low
TCGA-CM-6162	1	0	5.934232	6.651978	5.193612	8.19517	6.984364	6.772186	3.961979	2.56349	2.836908	0.677204678	low
TCGA-A6-6137	2.257534247	0	4.310086	6.596114	5.410379	8.427547	5.512067	4.870952	4.538774	4.573703	3.246888	0.923085786	low
TCGA-AA-3697	7.087671233	0	4.932269	6.614953	5.362428	8.787494	5.216913	6.081021	4.244082	3.231218	3.643499	0.359259441	low
TCGA-CA-6716	1.016438356	0	2.155487	6.8404	5.012343	8.241994	4.000551	6.053261	3.621667	4.092169	3.807904	0.831167431	low
TCGA-DM-A1D0	10.88767123	0	1.945398	6.28273	5.435089	9.037747	4.374198	4.881456	4.337626	3.307711	3.379951	0.630448676	low
TCGA-RU-A8FL	3.224657534	0	5.883175	6.378267	6.576111	8.926178	3.902366	4.061755	3.699051	4.698114	3.008492	0.529021793	low
TCGA-CK-5912	4.090410959	1	3.622851	7.327089	5.713414	8.610072	5.040878	4.849786	3.907648	4.127448	3.680912	0.324679031	low
TCGA-A6-5667	2.430136986	0	3.296747	6.827053	4.821037	8.394032	5.645392	7.268104	4.692387	3.108264	3.515296	0.650239511	low
TCGA-AZ-6605	0.435616438	1	3.044192	6.864385	5.457197	8.381891	7.05264	6.429821	3.779595	3.416862	3.012775	0.963383471	low
TCGA-G4-6322	2.169863014	0	3.119049	6.790711	5.877183	8.756208	5.849822	5.110504	4.310092	3.495864	2.710271	0.725200722	low
TCGA-A6-2678	3.523287671	0	2.406092	6.668399	5.432629	8.761697	7.879094	5.081284	4.415408	3.323148	3.197543	0.718381678	low
TCGA-G4-6323	1.147945205	0	5.55793	6.944875	4.949286	8.750394	5.148425	4.240714	4.136364	3.476282	3.356301	0.382838604	low
TCGA-A6-6650	1.717808219	0	3.047258	6.430087	6.239489	8.236104	4.607254	4.230701	5.387146	3.253432	3.836569	0.203822241	low
TCGA-DM-A28M	7.931506849	0	3.633236	5.692884	5.833093	8.86005	4.717064	4.518601	4.516876	2.797468	2.72286	0.781216117	low
TCGA-CM-5344	1.835616438	0	3.791537	7.244017	5.402455	8.740993	5.748447	6.59617	3.795115	3.468701	3.179108	0.556834247	low
TCGA-AA-3494	0.084931507	0	4.187789	7.114538	5.209469	8.101417	4.544912	5.194764	4.288668	4.033897	3.758765	0.416632255	low
TCGA-CM-4743	1.920547945	0	6.606605	6.502017	6.402398	8.965251	9.495738	5.128249	4.245459	2.595142	2.178198	0.497786879	low
TCGA-QG-A5YX	2.747945205	0	5.624138	6.529453	6.280579	8.646079	4.078138	3.873609	5.352325	4.418632	2.948528	0.421384035	low
TCGA-AA-3664	4.501369863	0	5.440831	6.535874	5.839884	7.94641	7.708698	5.629868	4.203855	4.025691	3.462709	0.617265833	low
TCGA-A6-6653	2.032876712	0	6.033326	7.00398	5.402215	8.479803	8.100159	4.994093	4.605318	2.368431	2.514309	0.415967016	low
TCGA-AY-A54L	1.438356164	0	3.30731	6.319072	5.710677	9.038088	4.431683	6.202528	4.375388	3.941798	3.25392	0.799168825	low
TCGA-AA-3685	3.087671233	0	5.221409	6.829377	5.559868	8.617954	7.040379	5.541207	4.273709	3.182931	2.894668	0.535881363	low
TCGA-CK-4952	1.301369863	0	4.47297	6.041038	6.056218	8.231566	7.481229	4.22812	4.58698	2.673581	3.681772	0.268750786	low
TCGA-AA-3530	1.589041096	0	3.815275	6.373691	5.940051	8.776061	6.120359	6.016092	4.3185	4.057578	3.4873	0.640201927	low
TCGA-A6-5660	2.432876712	0	3.484838	7.29662	5.609785	8.203027	5.623512	6.241755	4.128332	4.498301	3.311045	0.751058005	low
TCGA-CM-6674	1.079452055	0	4.791239	6.357598	5.587851	8.492086	7.115882	6.48114	3.934948	2.641091	2.622692	0.887297152	low
TCGA-AA-A00J	1.504109589	0	3.154157	6.244118	5.691236	8.234623	7.887383	5.560548	3.890054	2.993949	3.33418	0.726450606	low
TCGA-AA-3939	1.082191781	0	3.41169	6.877643	6.363435	8.819078	6.120025	5.138062	4.782216	3.527518	2.425629	0.622730609	low
TCGA-T9-A92H	0.991780822	0	2.945775	7.541867	5.755123	8.702605	4.403503	4.540707	4.740374	3.066663	2.882936	0.282710293	low
TCGA-A6-2672	3.887671233	0	5.4094	6.479468	4.92355	7.850013	8.205169	5.07529	4.705224	3.114196	3.75428	0.456345359	low
TCGA-A6-2684	3.087671233	0	3.886974	6.140616	5.504098	8.079974	6.816133	6.07986	3.980606	3.104303	3.649524	0.636982942	low

TCGA-AA-A01F	2.668493151	0	3.987825	5.825533	5.601993	7.993288	4.9367	3.699209	3.77119	3.246996	3.345074	0.700211722	low
TCGA-AA-3977	2.084931507	0	3.034403	6.921526	6.78329	8.900177	6.530907	4.827923	4.396857	3.400218	2.258923	0.577661099	low
TCGA-AA-3955	1.747945205	0	3.944873	7.506979	6.116015	8.804944	5.264761	4.813235	4.793962	2.747153	3.599884	0.104268311	low
TCGA-AA-3971	1.339726027	0	5.67744	7.268858	5.802341	8.497077	7.081528	6.385021	4.05971	3.710524	3.512795	0.312066364	low
TCGA-AA-3818	0.082191781	1	3.228537	6.767235	5.294205	8.735346	7.985514	4.895549	4.080909	3.158347	3.264342	0.590131442	low
TCGA-AA-A01V	0.084931507	0	7.04198	6.187765	4.838488	8.034801	5.827395	3.911978	3.67836	3.979	3.257367	0.904862794	low
TCGA-AA-3524	3.002739726	0	5.701486	7.122839	5.731353	8.566588	6.852268	5.010377	4.852753	2.677002	2.142238	0.480889553	low
TCGA-AA-3989	0.663013699	1	3.128527	6.979298	5.954241	8.357876	6.089012	5.306686	3.888159	4.145059	3.326733	0.614636586	low
TCGA-D5-5541	4.660273973	0	3.964007	6.360933	4.927449	8.130545	6.49087	6.081962	4.111286	3.560199	3.719174	0.821687607	low
TCGA-AA-3666	0.167123288	1	6.420061	5.550845	6.16573	9.020802	6.473982	6.423053	4.323375	3.086809	3.09701	0.585753646	low
TCGA-AA-A01Z	3.084931507	0	2.127025	6.383555	6.008857	8.178611	5.354892	4.924641	4.216179	3.507115	3.391078	0.614314666	low
TCGA-AA-3519	0.756164384	0	5.926926	6.611673	5.876033	8.462084	6.566701	4.551374	4.508325	3.421041	3.429347	0.289839736	low
TCGA-D5-6539	1.04109589	0	5.145587	7.319016	6.525034	8.443512	5.963765	4.731794	4.52927	3.161466	3.041319	0.183520334	low
TCGA-D5-6920	1.032876712	0	5.103482	6.481	5.573109	8.439606	5.482201	4.877912	4.377406	3.054837	2.778919	0.593918841	low
TCGA-CM-6678	0.917808219	0	2.724429	6.281189	5.355576	8.651408	5.715873	5.27058	4.185214	3.286457	3.119666	0.915104008	low
TCGA-AA-3941	2	0	2.577099	6.584712	5.714024	9.22474	7.468907	4.514934	4.410354	4.67402	3.669354	0.689357932	low
TCGA-AA-3855	2.671232877	0	8.78439	6.41519	5.419563	8.222094	5.266317	5.294127	4.670143	3.032057	2.896791	0.375914126	low
TCGA-AY-5543	2.750684932	0	3.200708	7.052741	6.581246	9.105397	7.931992	4.249778	5.321207	3.066013	2.674814	0.285247576	low
TCGA-AA-A00W	1.249315068	0	3.476242	6.462222	5.576802	8.582119	5.729416	4.519644	4.256793	3.248772	3.747398	0.349929128	low
TCGA-AA-3560	1.665753425	0	5.560345	6.758511	5.604943	7.858517	7.411338	5.886191	3.58164	3.167555	3.415675	0.468716411	low
TCGA-AZ-6598	4.117808219	1	2.708507	5.799968	5.446354	8.392609	7.552203	6.392815	4.072383	2.371906	3.221609	0.951883244	low
TCGA-G4-6321	1.84109589	0	4.026026	6.87032	6.245627	8.694198	6.255261	3.64935	4.702413	2.922073	2.40211	0.400694909	low
TCGA-AA-3866	1.419178082	0	2.919363	6.930235	5.899021	8.284162	6.315196	6.154829	4.480433	4.038069	2.94696	0.908914879	low
TCGA-AY-6197	1.78630137	0	6.353035	6.333801	5.081654	7.958166	5.98515	4.894098	4.363939	2.796176	2.668523	0.771475039	low
TCGA-AA-A01T	2.753424658	0	4.312946	6.697491	5.85987	8.831944	7.678741	5.033819	3.956852	4.41275	3.08617	0.858848171	low
TCGA-AA-A00Z	1.832876712	0	4.065302	6.244323	5.519588	8.682554	6.671195	4.973539	4.571868	3.959733	3.282572	0.82297361	low
TCGA-CK-5914	1.832876712	0	2.864602	7.174835	5.276966	8.451526	6.403811	4.913431	4.059203	2.998595	3.809435	0.27878484	low
TCGA-AD-A5EJ	0	0	5.213842	6.706601	5.696521	8.380523	5.191445	5.451382	3.828785	3.029489	2.831649	0.532460919	low
TCGA-DM-A282	11.59726027	0	5.814616	6.13443	5.355799	8.84741	5.786436	5.856146	4.269655	4.384933	3.472569	0.854713861	low
TCGA-AA-3949	2.167123288	0	4.460346	6.817525	6.022345	9.008465	8.197402	6.099935	3.714044	2.741309	2.121003	0.853893313	low
TCGA-A6-4107	2.704109589	0	5.315115	7.094752	6.152376	8.653988	6.605937	5.877464	4.533843	3.881747	2.6611	0.555526591	low
TCGA-G4-6293	11.09863014	0	4.296365	6.608426	4.983041	8.40476	5.845567	4.722655	4.647714	3.370878	2.941587	0.805556062	low
TCGA-AA-3522	3.087671233	0	3.829692	6.9543	6.51038	8.379204	6.072048	4.785423	5.017157	3.262974	2.657336	0.379011645	low
TCGA-CM-4748	2.169863014	0	3.1047	6.759564	5.846444	8.388335	7.614708	6.572374	4.928073	3.849778	3.447315	0.624163308	low
TCGA-AA-3869	2.252054795	1	5.284723	6.649259	5.809352	8.297025	6.076553	5.047625	3.555167	3.411383	2.935567	0.605195792	low
TCGA-AY-6386	1.484931507	0	4.185923	7.06254	5.740147	8.841112	5.336173	4.075222	4.866959	4.163488	2.289141	0.853688045	low
TCGA-A6-2680	2.926027397	0	6.500024	6.561529	5.979617	7.723187	6.377661	5.387646	4.140898	3.582644	3.559695	0.347101101	low
TCGA-AA-3862	2.504109589	0	7.479832	6.349334	6.738916	8.53505	8.160203	5.813495	5.153683	3.241815	2.693249	0.347303659	low
TCGA-A6-3810	3.043835616	0	3.061037	6.363377	5.40642	8.389075	5.740951	5.536319	4.910983	3.428051	3.609863	0.557921579	low
TCGA-AA-3693	0	0	2.474858	6.834235	5.448344	8.833752	4.49418	6.747161	4.700039	3.058476	3.570444	0.395371134	low
TCGA-AA-3814	0	0	4.400863	7.098705	5.577901	8.692325	6.052894	5.905927	3.761361	3.203647	2.891747	0.542798298	low
TCGA-AA-3679	1.252054795	0	4.184196	6.85115	5.693355	8.867658	5.223867	6.150918	3.94129	3.472162	3.268617	0.470157333	low
TCGA-G4-6303	5.487671233	1	2.434277	6.871503	4.675385	7.548996	5.092306	5.59077	4.333586	3.520316	4.184598	0.525500058	low
TCGA-G4-6586	2.983561644	0	4.987683	6.274588	5.67246	8.638968	8.960913	4.064973	3.975636	3.013894	2.953381	0.619454643	low
TCGA-QG-A5YV	3.564383562	0	4.176896	6.688178	5.332778	8.668822	5.866941	6.406375	4.113604	3.96664	3.173751	0.928425331	low
TCGA-AA-3495	3.087671233	0	3.849569	6.935922	5.714097	8.539139	5.516709	5.176183	4.742186	4.445914	3.158357	0.683978816	low
TCGA-AA-3842	3.084931507	0	6.394299	6.374676	6.306229	8.2402	6.541294	5.978848	4.14882	2.600751	2.941449	0.342102593	low
TCGA-AA-3844	1.243835616	0	4.485625	6.407072	6.354362	8.74697	6.176574	4.444493	4.572464	2.625523	3.215501	0.230580049	low
TCGA-AA-3982	2.252054795	0	5.110059	6.944783	5.909576	8.700483	5.901749	5.487192	4.785388	4.007772	3.069159	0.462728314	low
TCGA-A6-6140	2.010958904	0	4.445386	6.889633	5.522261	8.426275	4.546688	4.7939	5.019558	3.568482	3.965993	0.208262847	low
TCGA-AA-3509	5.246575342	0	5.887325	6.717401	5.406878	7.548409	6.595789	5.143233	4.702005	3.655202	3.15417	0.614793771	low

TCGA-QL-A97D	1.824657534	0	3.783887	6.500437	5.661103	8.545325	7.911473	5.237768	4.581687	3.140044	3.092865	0.611504808	low
TCGA-F4-6461	0.926027397	1	3.171119	6.491505	5.116242	8.43991	6.684984	5.824419	3.33149	3.520196	3.815496	0.727530797	low
TCGA-AZ-6600	1.008219178	1	5.22763	5.975858	5.764561	8.088695	6.557621	6.375317	4.012071	3.001906	2.896493	0.938399972	low
TCGA-AA-3520	2.002739726	0	3.615887	7.151297	6.094989	8.286181	8.877021	6.214306	3.950193	3.880315	2.98551	0.746757417	low
TCGA-AA-3854	3.002739726	0	3.032696	6.719473	5.123043	8.741294	5.338172	5.982326	3.853475	3.311471	2.985288	0.951292962	low
TCGA-G4-6588	2.180821918	0	4.477418	6.384715	5.702685	8.053113	8.08935	5.301222	3.951216	2.569325	3.052088	0.573643251	low
TCGA-CM-5348	1.915068493	0	6.345457	7.123529	4.999567	8.624328	6.346828	6.699297	3.778002	4.909547	3.484003	0.868089548	low
TCGA-AA-3875	1.504109589	0	4.763433	6.728359	5.589172	8.766303	6.286736	5.247891	4.881946	4.160346	3.044776	0.68601734	low
TCGA-AA-3510	5.331506849	0	3.978052	6.10165	6.127285	8.248656	5.653262	5.225843	3.615132	3.123445	2.774673	0.856892434	low
TCGA-AA-3555	2.495890411	0	2.515602	7.173713	5.468853	8.315169	7.44832	5.249048	4.484251	2.823902	3.266391	0.408935866	low
TCGA-A6-6781	1.638356164	0	3.82443	6.366456	5.066241	7.86311	6.661286	6.723748	4.287549	2.688768	3.401474	0.749138679	low
TCGA-A6-6141	0.698630137	0	5.031529	6.837716	5.797335	8.683815	8.299587	5.286637	4.159795	3.525357	3.276642	0.435313027	low
TCGA-A6-6138	1.876712329	0	4.495408	7.119933	5.222663	8.47703	7.152445	5.565856	3.680416	2.540348	3.767696	0.236234017	low
TCGA-DM-A1D7	1.109589041	1	4.439254	6.665269	5.992251	8.740837	8.230095	5.9127	4.198584	3.318199	3.307698	0.44445154	low
TCGA-D5-6927	0.78630137	0	3.33008	6.689715	5.403303	7.839211	7.01034	6.06811	3.591301	2.89418	3.234597	0.748370251	low
TCGA-G4-6306	3.723287671	0	6.10511	7.052194	5.296199	8.655121	8.642701	4.742388	4.129729	3.378899	3.380797	0.346973386	low
TCGA-AA-3556	1.917808219	0	4.687404	6.86466	5.274643	8.76842	7.694546	4.118238	4.382016	3.034267	3.433565	0.316183245	low
TCGA-AA-A01G	1	0	3.869847	6.835556	5.064349	8.390895	8.189951	4.526012	3.874018	3.043691	3.353174	0.546794941	low
TCGA-AA-A010	2.915068493	0	5.670021	6.087753	5.400387	8.014754	6.657897	5.229415	4.686104	3.438333	2.894065	0.964672804	low
TCGA-5M-AATE	3.287671233	0	2.400546	6.23223	5.158113	8.478893	5.731212	5.555048	3.530911	3.387868	3.632842	0.889964772	low
TCGA-CM-6679	0.838356164	0	4.019007	6.773407	5.248175	8.334319	6.769812	6.756368	3.848915	4.446449	4.084381	0.680288075	low
TCGA-AA-3532	2.416438356	0	5.426865	7.257498	6.303087	8.539667	6.779806	5.650269	3.908685	4.056192	2.784226	0.502805779	low
TCGA-AA-3531	2.835616438	0	4.723245	5.969506	5.439122	7.955018	4.906751	4.38507	3.48344	4.37729	4.108023	0.662583085	low
TCGA-DM-A28G	5.065753425	1	4.341398	6.79963	5.296446	8.459878	5.239337	5.091353	4.161038	4.831818	3.407829	0.912130259	low
TCGA-AA-3956	2.835616438	0	4.601042	6.600257	6.037089	8.449953	5.692901	5.785857	4.28661	5.065311	3.285594	0.940797124	low
TCGA-A6-3807	2.887671233	0	3.596247	6.45097	6.046543	8.617926	5.792134	5.730229	4.515765	3.274233	2.641962	0.784240574	low
TCGA-AA-3688	1.583561644	0	3.544995	6.657762	6.104051	8.568272	5.085082	4.92257	3.704256	4.599138	3.409997	0.677367354	low
TCGA-CM-6168	1.082191781	0	3.866281	6.779335	5.22657	8.156958	6.960514	7.004339	4.032596	3.367422	3.134773	0.964194748	low
TCGA-DM-A28F	2.997260274	1	3.79489	6.610089	5.40172	8.473402	7.545528	5.10212	4.22762	3.746736	3.141401	0.868060493	low
TCGA-DM-A28C	6.780821918	1	2.221387	6.386088	5.712095	8.314808	3.933533	4.4078	3.094298	3.647668	3.674316	0.599475852	low
TCGA-DM-A1DB	3.693150685	1	7.046592	6.510387	4.606984	7.573241	5.169133	4.810324	3.56638	3.67248	3.629687	0.636659613	low
TCGA-CK-4951	5.846575342	1	4.397077	6.583314	6.148439	8.266469	9.517861	6.639145	4.246155	2.70841	2.635494	0.733704839	low
TCGA-AA-3979	2	0	4.287547	6.54949	6.208365	9.027254	9.175336	3.877421	4.365125	3.630414	2.283149	0.894958978	low
TCGA-A6-5665	1.838356164	0	5.440765	6.55853	5.010612	7.911688	7.794661	4.920055	5.266639	2.827275	3.043683	0.564275809	low
TCGA-AD-6965	2.205479452	0	3.90594	7.112907	4.982762	7.844243	6.039768	5.452325	4.046805	3.350127	3.297866	0.63174588	low
TCGA-AA-3680	0.917808219	1	4.062091	7.146783	5.379628	8.769394	6.908133	4.875592	4.296848	4.560178	3.264368	0.712826381	low
TCGA-AZ-4615	2.745205479	0	3.389926	6.746588	5.735279	8.315854	7.83732	5.422708	4.061712	2.568132	2.322506	0.902012669	low
TCGA-AZ-5403	5.232876712	1	4.138013	7.677666	5.759604	8.407895	5.719019	6.697888	4.091109	5.08507	3.269945	0.716536507	low
TCGA-AZ-4315	4.865753425	0	4.276006	7.241504	6.209151	8.997825	6.634649	5.889632	4.011533	2.532008	2.581194	0.321796966	low
TCGA-AA-3972	4.249315068	0	2.756335	6.559085	5.713124	8.828527	4.575445	4.698962	3.683449	3.244086	3.492353	0.421585084	low
TCGA-AA-3517	3.249315068	0	5.23699	5.95354	5.025531	7.216787	5.311418	5.326716	3.913225	3.962139	4.202886	0.723282961	low
TCGA-AA-3877	2.583561644	0	7.071958	6.732385	6.064968	8.406829	8.4286	5.874217	3.933798	2.977019	3.058781	0.338070579	low
TCGA-NH-A50T	1.515068493	0	3.219362	6.209965	5.252786	8.342343	4.025092	4.899567	4.125517	3.529956	3.197484	0.924952863	low
TCGA-F4-6809	1.104109589	1	3.912285	6.406846	5.532435	8.388649	7.219637	6.782588	3.564975	3.480622	3.353165	0.923275856	low
TCGA-G4-6309	7.123287671	0	5.776317	6.841699	5.408449	8.603723	6.542532	5.208496	3.95175	2.699094	2.295553	0.684825664	low
TCGA-CK-5915	0	0	2.174978	6.400285	4.668074	8.346215	6.547668	3.637302	4.337182	2.685494	4.162865	0.353518798	low
TCGA-AD-6889	6.936986301	1	3.489577	7.289902	5.568897	8.22331	5.363713	5.137798	4.666464	2.690183	2.928446	0.342556762	low
TCGA-AA-3968	1.832876712	0	3.620761	6.57234	5.507717	8.341827	5.564314	5.967376	4.16551	3.41948	2.925322	0.91623667	low
TCGA-CM-6675	1.087671233	0	6.048898	6.708227	4.908284	8.631323	8.853747	5.429879	5.275479	3.469177	3.236861	0.565883698	low
TCGA-A6-A565	1.353424658	1	4.711475	6.721189	5.603201	8.46389	6.202804	5.921443	4.475076	3.780422	2.852276	0.832179588	low
TCGA-CM-6165	1.336986301	0	4.511281	6.816415	5.825528	8.551316	5.826461	5.694934	4.438247	3.015793	3.697724	0.230286624	low

TCGA-AY-4071	0.079452055	1	5.255681	6.808506	5.683475	8.951848	6.641816	5.618544	4.400037	3.420098	3.439151	0.326029924	low
TCGA-CM-6163	1.169863014	0	4.226325	6.839729	5.880893	8.115835	5.914967	5.206471	4.2984	3.88214	3.16688	0.577114922	low
TCGA-A6-2677	2.02739726	1	3.175903	6.226532	4.943604	8.369976	4.147037	4.373579	5.583718	3.803216	3.114363	0.96542832	low
TCGA-AA-3712	0	0	3.946688	6.623576	4.986139	8.204637	5.960549	6.022764	3.773313	3.282772	3.150465	0.963648312	low
TCGA-G4-6628	6.64109589	0	4.578579	6.32763	5.123196	7.779916	9.638384	4.835093	3.530688	2.778778	3.260504	0.845755	low
TCGA-D5-6898	0.62739726	0	3.917178	6.834237	5.119364	8.688283	5.848475	6.748322	4.845251	4.464019	4.092175	0.530701799	low
TCGA-AA-A00F	2.835616438	0	5.753495	6.972183	5.306516	8.239683	5.800856	5.679289	3.782211	3.444657	2.953293	0.62383446	low
TCGA-CK-4950	7.120547945	0	5.54123	6.660615	5.57812	8.428559	6.595788	5.330507	3.92939	3.096718	2.575287	0.738202386	low
TCGA-F4-6806	3.452054795	0	5.767048	6.280888	5.199248	8.693794	5.839483	5.673913	4.042297	3.81362	3.295636	0.784630461	low
TCGA-F4-6856	2.942465753	0	3.282718	6.920777	5.620896	7.938782	8.522043	5.667455	3.991884	3.272801	3.029283	0.795027239	low
TCGA-AA-A00Q	3.501369863	0	3.291619	6.996373	5.34176	8.329351	5.993265	5.787044	4.149203	4.006873	3.563898	0.629554682	low
TCGA-D5-6922	0.843835616	0	5.440201	6.741876	4.512932	8.590548	6.777853	7.051956	4.171623	3.856677	3.830521	0.714467759	low
TCGA-D5-5537	3.783561644	1	3.923527	6.439521	4.472823	8.621099	7.557721	6.629192	4.507425	3.673855	4.279628	0.62845841	low
TCGA-DM-A1D4	7.728767123	1	7.655115	6.067027	6.050307	8.149543	6.347752	5.290524	4.644023	3.81439	2.722966	0.726357799	low
TCGA-AA-3950	2	0	3.963018	6.986866	5.772375	8.452667	8.208516	5.977826	3.501194	2.159874	2.431659	0.619704038	low
TCGA-D5-6532	1.520547945	0	3.400433	6.405115	5.758709	8.427289	5.778529	4.39811	3.70651	3.150068	3.269088	0.531880712	low
TCGA-AA-3831	1.498630137	0	5.737049	6.742769	5.752332	8.818347	6.386758	4.766172	4.393944	3.390311	2.925209	0.423449548	low
TCGA-CM-6171	1.169863014	0	3.813862	6.630712	5.202174	7.989069	5.041644	4.91637	4.186155	3.064828	5.20839	0.112758298	low
TCGA-AA-3970	3.002739726	0	5.623543	6.795445	6.150307	8.781194	6.319903	4.712944	4.304807	4.38479	2.916061	0.565417297	low
TCGA-DM-A1D9	11.69863014	0	2.661915	6.808238	5.742275	8.949369	7.802933	7.206409	3.897487	3.877255	3.432472	0.806756643	low
TCGA-G4-6315	5.15890411	0	4.575639	6.768602	5.456966	8.642207	3.880494	5.430626	4.866847	3.217352	3.938494	0.201384109	low
TCGA-4N-A93T	0.4	0	3.136764	6.200653	6.006702	8.232286	4.357989	5.842002	4.478497	4.633179	2.91958	1.550913758	high
TCGA-AA-A00K	1.504109589	0	5.81044	5.906307	5.251455	8.393687	7.504459	6.232399	3.983442	3.384575	2.551565	1.833955787	high
TCGA-G4-6307	4.58630137	0	2.73999	6.784413	4.889939	8.954077	4.013309	5.239777	4.820699	5.696549	3.463887	1.567820355	high
TCGA-A6-2683	1.380821918	1	2.335644	5.643416	5.468729	6.836463	8.196102	4.550862	3.137581	4.982004	3.360398	4.53625522	high
TCGA-DM-A285	0.490410959	1	3.218298	6.441055	5.35586	8.269564	4.99106	6.409225	3.8748	4.397611	2.515416	2.801974311	high
TCGA-A6-5666	2.726027397	0	2.148141	5.755802	4.889059	8.668041	5.004625	4.422045	3.652065	3.459772	2.987483	1.958466826	high
TCGA-AA-3662	0.504109589	0	5.339059	6.302806	5.192076	8.393221	6.948591	5.813101	3.584113	6.038413	3.215062	3.233890049	high
TCGA-CK-5913	4.276712329	0	3.231435	6.395712	5.214635	8.514497	8.70592	5.388733	4.498847	2.967455	2.326831	1.668391232	high
TCGA-AA-3492	0.002739726	1	4.349853	6.362867	6.054393	7.745553	8.335676	5.491317	3.93044	2.521069	2.279604	1.030800177	high
TCGA-A6-2686	3.084931507	1	3.997919	6.504197	5.753114	8.285376	8.533015	5.603187	3.739942	3.121377	2.406571	1.31442132	high
TCGA-A6-6654	1.989041096	0	5.56957	6.653986	4.835057	8.086389	7.8166	6.834627	2.889894	3.238409	3.070384	1.251199652	high
TCGA-CM-5863	1.252054795	0	4.678897	6.352756	4.71421	8.329753	6.334897	6.35303	4.030191	4.508273	2.842097	2.754243486	high
TCGA-AA-3506	4.835616438	0	7.852078	6.276646	5.08656	7.747133	7.609558	6.125269	3.953465	3.826711	3.152988	1.025164398	high
TCGA-NH-A50U	0.915068493	1	3.352617	5.202293	5.211527	8.082547	9.013206	6.445007	4.201862	3.92987	2.435069	6.633785844	high
TCGA-A6-2681	3.8	0	3.874116	6.499785	5.715852	7.665367	7.42691	6.563373	3.596872	3.947805	3.453595	1.048399621	high
TCGA-AU-3779	1.208219178	0	3.424983	6.301377	5.442422	8.498386	6.398956	5.44303	4.647891	4.049732	2.893633	1.400131242	high
TCGA-NH-A6GC	1.065753425	0	2.760672	6.202016	5.301097	8.431368	5.925121	6.75953	4.663788	4.119035	3.682362	1.087103809	high
TCGA-A6-2676	3.575342466	1	6.361661	6.240306	5.850238	8.507141	9.14711	5.06388	3.99688	2.599054	1.94438	1.077548483	high
TCGA-D5-6534	3.605479452	0	3.922042	6.686709	5.120243	8.120612	8.890758	6.716422	3.220104	3.311159	2.533082	2.130888521	high
TCGA-F4-6807	3.58630137	0	3.724297	6.796941	5.554721	7.92883	8.538067	6.623761	3.853412	4.384236	3.221642	1.41491845	high
TCGA-G4-6310	5.301369863	0	2.207415	6.881252	5.329554	8.218829	5.038573	8.902947	3.837505	3.755199	3.31312	1.312309807	high
TCGA-CM-6167	1.249315068	0	3.839644	6.414709	5.190755	8.248876	6.975422	8.661658	3.863563	4.266186	2.974373	2.724910188	high
TCGA-CM-6676	0.923287671	0	4.285174	6.494014	4.528571	8.546584	5.305302	6.186816	5.028986	6.023659	3.647985	2.324736849	high
TCGA-A6-3809	2.728767123	0	3.385414	5.968247	4.803047	8.41726	7.163108	4.104753	4.620506	2.253035	2.246039	1.5269342	high
TCGA-AA-3845	0	1	4.497355	6.705162	5.150775	8.554959	8.120823	6.191259	3.919363	3.815775	2.400216	1.998580476	high
TCGA-AD-A5EK	1.369863014	0	2.562268	5.951359	4.868171	7.89809	6.298355	5.441685	3.533531	3.822003	4.04081	1.198358208	high
TCGA-AD-6899	0.482191781	1	3.293704	6.396086	5.654098	7.85286	8.769625	6.463158	4.631269	5.327459	2.62558	4.212662049	high
TCGA-CA-5256	1.038356164	0	4.619748	6.64404	5.978662	8.376171	5.23314	4.913451	5.464677	5.92895	2.613414	1.829485365	high
TCGA-AA-3554	1.495890411	0	4.481072	6.153708	5.772745	8.398769	6.478191	6.197339	4.021473	2.97961	2.250939	1.439270732	high
TCGA-AA-3667	1.167123288	0	3.601869	5.287524	4.678611	7.911799	5.98271	5.846954	3.444507	4.073645	3.91298	2.256494144	high

TCGA-AZ-6601	8.334246575	1	5.440128	6.191172	5.178673	8.42141	7.135743	5.803508	4.289325	3.153235	2.323945	1.592944229	high
TCGA-AA-A02E	0.246575342	1	3.514254	6.484666	4.915698	8.271315	8.764871	6.701572	3.617371	4.104657	2.730016	3.161941153	high
TCGA-AA-3692	3	1	5.533788	6.676362	5.245926	8.742934	5.63663	5.780653	4.045975	5.245832	3.42957	1.106389358	high
TCGA-CM-5341	2.421917808	0	5.29137	7.524417	6.736081	8.32307	6.323789	5.674671	4.45686	6.270456	2.173474	1.606507013	high
TCGA-AD-6548	1.780821918	0	4.178186	6.500042	5.258789	8.43611	8.592981	5.787111	3.885516	3.401168	2.455616	1.756732475	high
TCGA-AA-3930	0.167123288	1	2.941902	7.132188	6.19655	8.03845	6.544272	6.150867	4.364147	3.547311	2.028713	1.277751075	high
TCGA-A6-6782	1.690410959	0	2.969364	6.141732	5.417424	8.52512	6.776194	5.890229	3.842194	4.168388	3.545075	1.254025826	high
TCGA-AM-5821	0.076712329	0	2.98261	5.849299	4.055206	8.41887	9.463436	6.129165	3.246072	2.439287	1.912663	6.754669242	high
TCGA-D5-6926	0.753424658	0	2.999672	6.662047	4.527953	7.790415	6.653603	7.221721	3.068273	4.689751	3.965767	1.996578865	high
TCGA-AA-3860	2.589041096	0	4.085487	6.262926	4.74459	8.575795	6.630068	5.649847	4.532075	3.648667	3.064763	1.377856983	high
TCGA-AA-A00R	0.082191781	0	5.480468	6.287307	5.493488	8.639182	9.597249	5.397216	4.542367	3.038979	1.917525	1.69890356	high
TCGA-AA-A00N	0.334246575	1	3.357794	6.500943	5.027973	8.389013	6.945463	6.624637	3.987904	4.117762	2.49374	3.045844091	high
TCGA-AA-3715	1.58630137	1	3.281245	5.56955	4.328188	7.896787	9.408893	5.415304	2.995804	3.251577	2.224656	8.09556977	high
TCGA-A6-6649	2.01369863	0	3.663097	6.724402	5.593584	8.036144	7.557113	6.547721	3.712381	4.687795	3.666705	1.095137498	high
TCGA-F4-6805	2.868493151	0	4.259782	6.03848	5.453205	8.20786	7.935232	7.460807	3.622878	3.4355	3.008405	1.748901279	high
TCGA-A6-2674	3.646575342	0	3.038428	6.126447	5.097127	7.860172	5.55923	5.95342	4.262439	3.23513	2.707468	1.898084937	high
TCGA-AA-3552	1.084931507	1	4.30932	6.618225	5.559696	8.589913	8.391403	5.976609	3.929805	4.71699	3.127572	1.454213797	high
TCGA-CM-5861	1.252054795	0	3.095407	6.565304	5.139721	8.276419	7.816517	6.46343	3.292714	3.945331	2.961935	2.106024605	high
TCGA-AA-A017	1.252054795	0	4.856007	6.409516	5.304029	8.47031	6.203088	5.709819	4.179906	5.302442	3.12517	1.947269776	high
TCGA-CM-5349	2.506849315	0	4.747027	6.172351	5.235788	8.968921	7.367172	7.453122	3.542755	4.549587	3.615964	1.455072475	high
TCGA-D5-5538	4.550684932	1	3.387682	5.95757	5.478189	8.50751	8.600007	5.935814	4.12606	2.907409	2.730038	1.472451668	high
TCGA-CK-6747	2.246575342	0	4.177032	6.519418	5.303594	8.473248	8.006419	5.196603	4.797337	3.493129	2.4929	1.316202621	high
TCGA-D5-6530	1.701369863	0	4.164994	6.703278	5.279798	8.221656	8.616984	4.369314	4.000403	3.769393	2.367241	1.695255212	high
TCGA-A6-5664	1.84109589	0	6.558297	6.671827	4.636494	8.31674	6.334015	6.617475	4.035135	3.779547	3.120759	1.050368987	high
TCGA-AA-3872	0	0	3.244579	6.929864	5.432989	7.732918	6.790793	6.918527	3.280206	4.41987	2.444799	3.116474261	high
TCGA-AD-6895	2.090410959	0	2.509229	5.920903	4.615902	8.215938	9.390607	6.209121	3.524941	2.898981	2.365705	4.471603128	high
TCGA-AU-6004	2.257534247	0	4.645862	6.653866	5.126627	8.050608	9.004067	5.948577	3.854731	3.027616	2.518603	1.440950258	high
TCGA-AA-3684	0	0	6.34528	5.633769	5.465583	7.609327	7.05111	6.070067	3.220734	4.2914	2.75374	3.022622083	high
TCGA-AA-3812	2.920547945	0	7.01477	5.654519	5.308428	7.82734	6.301406	5.930223	3.200406	3.842428	3.152483	1.530734893	high
TCGA-A6-2671	3.646575342	1	2.623911	6.42374	5.230094	7.418813	8.824994	6.877616	3.33506	3.945889	3.315285	2.30032511	high
TCGA-D5-6531	1.479452055	0	2.709374	6.179742	4.489398	7.940211	8.525048	5.156362	3.88709	3.021891	3.186366	1.748320798	high
TCGA-CK-6748	0.167123288	0	2.619175	6.591293	4.525378	8.105665	5.988824	6.699394	3.504612	5.587789	2.975166	6.134644957	high
TCGA-AA-3496	0.084931507	0	4.274424	6.058746	4.983528	7.970924	6.537511	5.820556	4.07162	5.13747	3.67176	2.080655387	high
TCGA-A6-6142	2.090410959	0	2.445094	7.122566	4.566564	8.351016	6.479753	6.509324	3.413484	4.740745	2.983675	2.836302961	high
TCGA-CM-4747	2.084931507	0	3.756105	6.085186	4.776801	7.471174	6.304265	5.934353	3.587994	4.97326	3.115548	4.311582048	high
TCGA-A6-5661	2.794520548	0	5.119606	5.818528	4.241525	7.023074	4.330718	4.747127	4.776995	2.425361	3.084827	1.128224486	high
TCGA-AD-6901	1.868493151	1	2.827441	6.631483	5.177487	7.961715	7.876688	6.777966	3.307122	4.60532	3.819458	1.536613618	high
TCGA-DM-A28A	2.205479452	1	2.234964	6.166984	5.378695	7.890145	6.614537	6.407663	3.357719	3.441535	3.609765	1.210355511	high
TCGA-G4-6304	4.468493151	0	2.556777	6.619676	5.689652	8.240727	8.606169	4.800787	3.83267	4.019181	2.585427	1.843547749	high
TCGA-AA-3870	2.498630137	0	3.492427	6.682722	5.174517	8.259986	8.220351	6.593397	4.13015	3.200879	2.564929	1.61086849	high
TCGA-AA-3710	2.249315068	0	3.996888	6.320733	5.868029	8.628255	8.821772	4.981603	3.777207	2.95339	2.300925	1.214920058	high
TCGA-CM-6677	0.923287671	0	2.796659	6.564666	5.762208	8.516711	6.114463	5.582373	4.315147	3.413059	1.969411	1.864282546	high
TCGA-AA-3966	0.167123288	0	3.964556	5.663695	5.746895	8.557076	6.576105	5.841731	3.235147	4.683351	3.558297	1.706900718	high
TCGA-G4-6299	6.21369863	0	3.837972	6.300413	5.381278	8.17714	7.731028	6.276663	3.392038	4.914271	3.393208	2.15839168	high
TCGA-AA-3856	0.082191781	0	5.460329	7.088982	5.321018	8.559596	7.043253	5.50274	4.096273	4.863942	2.939728	1.128048151	high
TCGA-DM-A0XD	2.035616438	1	3.237916	5.926311	5.008157	8.619116	8.406945	5.954317	3.407666	3.277215	2.468094	3.1497381	high
TCGA-AZ-6607	0.265753425	1	3.750041	6.614805	4.138176	7.695659	8.133929	6.496966	3.620049	5.721126	2.051548	17.06780936	high
TCGA-AD-6890	2.043835616	0	6.26701	6.576045	5.126576	8.316444	6.908982	6.009905	4.43701	3.780201	2.169452	1.804043133	high
TCGA-AA-3489	0.58630137	1	2.515702	6.289473	5.007163	7.620011	7.197334	6.371109	3.669385	3.538515	2.821214	2.726890827	high
TCGA-3L-AA1B	1.301369863	0	3.524078	6.165577	4.929179	8.187618	6.330141	6.22541	4.048898	3.912438	3.640393	1.255012051	high
TCGA-AA-3527	0	0	6.221888	5.594085	4.570038	6.881225	7.874743	5.390563	2.764796	4.489348	2.641479	7.140225603	high

TCGA-D5-6924	1.191780822	0	4.056105	6.255872	4.607406	7.836028	6.796504	6.851438	4.105394	4.5491	3.61045	2.14648063	high
TCGA-AA-A01C	1.252054795	0	2.98538	6.592068	4.983001	8.315692	6.753387	6.433515	3.857481	3.695698	3.004766	1.646069972	high
TCGA-AA-3852	0	1	3.050263	6.562051	4.89077	8.486575	6.443593	5.787122	3.80381	3.356482	2.486882	2.006574048	high
TCGA-AZ-4313	6.328767123	0	2.810299	6.518053	5.357251	8.503075	5.071143	5.482265	3.952218	3.792078	2.398852	1.946069073	high
TCGA-NH-A5IV	0	0	3.780369	6.35578	4.950288	8.161487	6.235788	5.470029	4.451981	4.340218	2.398769	3.164282757	high
TCGA-CM-6164	2.419178082	0	2.903277	6.62504	4.880151	8.54974	6.326115	6.840993	4.01565	3.74408	3.520455	1.089139664	high
TCGA-CK-6751	1.419178082	0	3.226911	6.14714	5.515077	8.451775	7.983762	5.749182	4.288142	2.697402	2.453069	1.392778301	high
TCGA-AA-A01R	2.917808219	0	5.560868	5.222667	4.745626	8.14049	9.016863	5.670229	3.663354	3.863651	1.928777	8.85485914	high
TCGA-CM-6680	1.002739726	0	4.08922	6.371462	5.054173	8.532016	7.839676	5.791615	4.339349	4.362704	3.194939	1.592922788	high
TCGA-D5-6928	0.969863014	0	8.088269	5.903416	4.949594	7.636764	9.051782	5.990364	3.29166	3.882357	3.159232	1.658523126	high
TCGA-5M-AAT4	0.134246575	1	2.350116	6.275081	5.074668	8.078203	5.272307	6.148025	4.561513	3.717446	3.101433	1.605755528	high
TCGA-AM-5820	0.038356164	0	5.019888	5.962754	4.511883	8.65332	5.523393	7.257214	5.066961	2.575316	2.413606	1.744316597	high
TCGA-F4-6459	0.717808219	1	4.820174	6.633555	4.712757	8.349548	5.678788	7.38363	3.802809	5.918025	2.945963	4.720516762	high
TCGA-AA-A00A	3.169863014	0	3.517713	5.8873	5.257585	8.438713	8.45394	5.281382	4.368719	3.021713	2.769824	1.529352462	high
TCGA-AA-3655	5.084931507	0	3.24777	6.915127	4.662431	8.397025	8.745401	5.485839	4.353958	3.502316	2.945279	1.311658857	high
TCGA-CM-6166	1.832876712	0	2.720032	6.014569	5.107481	8.572196	7.711434	6.149362	3.701816	3.18365	3.305573	1.367756241	high
TCGA-DM-A1D8	1.049315068	1	2.471857	5.85882	5.930262	8.322491	8.240443	5.393374	3.730951	3.785776	3.425471	1.226852166	high
TCGA-F4-6570	0.515068493	1	6.982329	6.071009	4.489758	8.154026	8.670699	6.032932	3.835841	2.853578	3.020755	1.204317864	high
TCGA-AA-A01X	2.167123288	0	3.877141	6.694697	4.328547	7.568645	7.556419	6.486228	3.585402	5.096903	3.649667	2.80044753	high
TCGA-AD-6963	2.284931507	0	2.808904	6.772402	4.754259	8.797906	6.28435	4.679192	3.748359	3.842597	2.906894	1.413850701	high
TCGA-AA-3811	0.838356164	1	4.689186	6.685935	5.789446	8.29473	6.992186	5.142651	3.414011	4.079825	2.485174	1.429278819	high
TCGA-A6-5662	1.967123288	0	2.499816	7.078777	5.292539	7.9076	5.014011	6.327269	3.821828	4.888886	3.164105	1.715250824	high
TCGA-DM-A0XF	3.183561644	1	5.180337	6.93476	5.441057	8.09652	7.532436	6.585741	4.223232	4.588003	3.144389	1.13657082	high
TCGA-A6-4105	1.210958904	1	3.248293	6.436282	5.110301	8.281359	6.905137	5.999524	3.90688	3.239382	2.772525	1.519982873	high
TCGA-D5-6535	1.260273973	0	3.057847	6.82387	5.287387	7.980191	7.148288	4.806578	4.746375	3.484402	2.812957	0.988212725	high
TCGA-A6-A56B	4.687671233	1	3.218581	5.897623	4.642568	7.509583	5.199223	6.370981	3.921226	4.461885	3.87194	2.143899666	high
TCGA-D5-6536	1.487671233	0	2.121489	5.667147	4.404613	7.674625	6.068506	6.258729	3.480202	3.662095	3.043566	4.713677321	high
TCGA-D5-6529	1.682191781	0	4.376323	6.408847	4.714296	8.361005	8.381479	5.729129	4.52928	3.205794	2.882051	1.347424968	high
TCGA-D5-6931	1	0	5.256498	6.546847	4.937471	8.244281	7.69471	5.419884	4.063562	3.128249	2.582624	1.246351273	high
TCGA-DM-A1HA	7.123287671	0	2.214634	6.251711	4.047578	8.132535	8.231515	5.172398	3.571279	2.971325	2.50872	3.835164435	high
TCGA-AA-3837	3.249315068	0	4.02244	6.291756	5.011792	8.248612	7.18235	6.552627	4.323008	3.408324	3.086903	1.340253623	high
TCGA-G4-6314	2.994520548	0	2.982045	6.716538	4.714466	7.998578	6.966393	7.829556	4.200852	3.872439	3.784274	1.206711096	high
TCGA-D5-5540	4.673972603	0	2.203071	6.682935	5.581804	8.032148	5.392188	6.660566	4.101655	3.247486	2.856852	1.137893841	high
TCGA-DM-A288	1.169863014	1	3.233412	5.687531	5.695254	7.79359	3.731782	4.759214	4.087232	3.270199	2.865358	1.291172508	high
TCGA-AD-5900	1.01369863	0	2.982619	5.790937	4.868985	8.275861	8.978335	6.285022	3.629081	2.751675	2.674876	2.753130857	high
TCGA-G4-6317	3	0	3.302737	6.687608	5.638654	8.660918	4.660916	5.563719	4.671877	5.518181	3.397269	1.212634256	high
TCGA-CA-5255	1.030136986	0	3.962269	6.183728	5.524949	8.226135	7.84152	4.5408	4.087255	3.279161	2.581771	1.342918495	high
TCGA-G4-6627	5.95890411	0	4.903921	6.557117	5.089672	8.241017	8.716157	7.191692	4.742818	3.219088	2.875786	1.171290962	high
TCGA-AA-A00O	2.252054795	0	3.117312	6.132526	5.231004	7.875252	7.942948	6.304903	2.854908	3.51241	3.579618	1.451748986	high
TCGA-AZ-4323	0.117808219	1	4.707593	6.506938	4.876215	7.983113	7.625951	6.532721	2.924562	3.6407	3.011819	1.861137559	high
TCGA-AA-3542	1.082191781	0	6.615124	6.214605	5.508036	8.817996	4.799782	6.063709	3.963629	6.104417	2.701241	3.070122304	high
TCGA-AA-A022	0	0	4.419777	5.862968	4.819563	8.6212	8.786203	5.660299	3.180405	3.926972	2.083759	5.917367422	high
TCGA-DM-A0X9	9.975342466	0	3.140193	5.920368	5.085322	8.358837	6.514331	4.832465	3.544837	3.250939	3.392553	1.124038603	high
TCGA-AA-3994	2.252054795	0	3.054675	6.7197	5.194889	8.634331	6.172695	5.334907	3.801889	3.459719	2.603425	1.346939217	high
TCGA-D5-6923	1.035616438	0	3.822791	6.440574	4.799314	7.966491	5.857296	6.179823	3.940181	4.1053	3.587917	1.287082016	high
TCGA-AD-6888	1.293150685	1	3.954127	6.205977	4.76318	8.28884	7.301422	5.182727	4.095011	4.409438	3.225673	2.02955762	high
TCGA-D5-7000	0.854794521	0	2.504916	6.326674	5.136275	8.32012	8.109294	5.762343	4.233867	3.128958	2.887983	1.488584044	high
TCGA-CK-4948	12.33424658	0	4.92325	6.43834	4.58653	8.420432	6.145769	6.876878	4.227281	4.671006	2.531406	3.806087522	high
TCGA-CM-4746	3.084931507	0	3.598849	6.228719	5.926764	8.614049	7.354345	4.885188	4.221983	4.350013	2.45496	1.934559698	high
TCGA-AZ-6608	0.161643836	1	2.011919	6.559507	5.423734	8.773304	3.674536	6.039537	4.1258	4.858179	3.22281	1.571890203	high
TCGA-AA-A02J	0.419178082	1	4.202392	6.287327	4.769236	8.351369	4.114174	5.463038	4.194185	5.009861	3.881866	1.209978263	high

TCGA-AA-3516	1.084931507	1	3.142157	6.50014	5.171621	8.493084	7.166759	4.346827	3.744509	2.663352	2.106417	1.534106087	high
TCGA-A6-6651	1.81369863	0	5.208372	6.033998	4.796483	8.04856	7.22677	7.987387	3.462681	3.640005	3.29883	2.016777636	high
TCGA-DM-A1DA	0.624657534	1	2.771392	5.549991	5.760286	8.345017	8.781856	4.939944	4.022358	2.924468	2.226614	2.565878117	high
TCGA-CK-5916	1.761643836	1	2.720455	6.035744	5.041166	8.146184	7.140891	5.349986	4.29792	2.367505	2.397233	1.736585527	high
TCGA-AZ-6599	0.564383562	1	2.434439	6.306733	4.984149	8.569853	5.771588	4.062996	4.320336	4.167969	3.332005	1.235729122	high
TCGA-CA-5254	1.057534247	0	5.459606	7.043082	4.548272	7.907046	7.961296	6.041683	4.486553	3.354556	2.661804	1.247557357	high
TCGA-DM-A280	0.646575342	1	4.535067	6.159556	5.102257	8.712525	4.91505	5.874789	4.135372	2.564152	2.093289	1.496962947	high
TCGA-QG-A5YW	2.454794521	0	3.189274	6.266181	5.686554	8.439386	5.737733	5.237583	4.453154	3.817048	2.094291	2.242653228	high
TCGA-AA-A029	4.331506849	0	3.144826	5.744606	4.848623	8.197698	5.795698	4.193832	3.705744	2.960544	2.217621	2.913592795	high
TCGA-AA-3984	0	0	3.606831	6.881864	5.035958	8.829968	6.928107	5.403369	4.233871	3.52768	2.756276	1.047894042	high
TCGA-AA-A00U	1.419178082	0	2.526926	6.214225	5.141935	8.770466	8.45771	6.316589	4.245788	3.911239	3.350033	1.567814118	high
TCGA-AA-A02H	0.167123288	1	4.120497	6.214451	4.738319	8.201578	5.630875	6.777037	4.106178	4.287492	3.239414	2.115632115	high
TCGA-D5-5539	1.632876712	0	3.509647	6.564628	5.235001	8.349357	5.835889	5.621797	3.454011	3.850672	2.662651	1.795437972	high
TCGA-AA-3562	1.665753425	0	5.587448	7.240896	5.966634	8.319943	5.591259	5.708356	3.718664	4.942736	2.556018	1.113831646	high
TCGA-CM-4744	1.668493151	0	4.466183	6.584106	4.889266	8.913795	8.864663	6.760165	4.134392	2.588791	2.149263	1.681541876	high
TCGA-NH-A8F8	1.4	1	3.282137	6.138004	4.957625	7.88772	7.086975	6.730968	4.172053	4.240546	3.636298	1.776308006	high
TCGA-CA-6719	1.191780822	0	2.58275	6.567966	4.963883	8.108541	5.937648	6.508557	3.39832	4.281622	3.380741	1.876628868	high
TCGA-CA-6718	0.838356164	1	4.58796	6.33005	4.921775	8.168076	8.942682	6.11946	3.07205	2.86251	2.950731	1.424047747	high
TCGA-AA-A01K	2.583561644	0	5.663151	6.09372	5.081343	7.949689	6.904126	6.169028	4.002178	3.967778	2.955585	1.799469076	high
TCGA-DM-A28K	8.18630137	0	3.267123	6.424657	5.618494	8.617294	4.312484	5.228736	3.726004	3.598526	2.513299	1.32254462	high
TCGA-F4-6855	3.950684932	0	2.978448	6.214491	4.695354	7.563374	6.772596	6.997867	3.873859	3.178989	3.64774	1.358600372	high
TCGA-D5-6541	1.298630137	0	3.713865	6.478213	5.862572	8.484452	10.098702	6.178562	3.790221	3.67602	2.813274	1.369980903	high
TCGA-AA-3841	3.079452055	0	5.720801	5.637022	5.169015	7.67163	6.947391	5.059991	3.490449	4.202786	3.417396	1.720171618	high
TCGA-4T-AA8H	1.054794521	0	4.111511	6.155526	5.477168	8.606215	3.846782	4.946979	4.245523	4.650432	3.072721	1.341800531	high
TCGA-AA-3696	0.419178082	1	4.046401	5.959753	5.306893	8.340762	5.994537	7.35375	3.771475	5.128409	3.165458	3.405337119	high
TCGA-AA-3549	1.750684932	0	2.516362	6.276564	5.663608	7.719962	6.535107	5.173455	3.789997	3.29604	3.071599	1.133158071	high
TCGA-A6-2685	3.104109589	0	6.154674	6.327691	5.996654	7.151953	7.079175	8.175149	3.901413	4.219738	2.915414	1.697803255	high
TCGA-AA-A02O	0.076712329	0	4.134789	6.020912	4.661456	8.092253	8.542569	5.025883	3.453942	4.246628	3.377019	2.308283039	high
TCGA-D5-6540	1.345205479	0	3.605992	6.013711	4.821445	8.182397	9.022601	5.902997	3.738278	3.086975	3.730102	1.048539773	high
TCGA-CA-6715	1.049315068	0	3.025776	6.111213	4.965449	8.589696	4.502835	5.905949	4.933563	3.917556	3.552596	1.012989362	high
TCGA-DM-A1D6	1.561643836	1	2.135229	5.472397	5.387244	8.113103	3.947318	4.676952	4.247247	3.675271	3.375587	1.443183935	high
TCGA-AA-A02R	1.835616438	1	2.924154	5.864594	4.731575	8.331885	9.943774	5.106557	3.568301	2.944814	2.403197	3.549654182	high
TCGA-AY-A8YK	1.569863014	0	4.262824	6.552997	5.35806	8.593028	4.657164	4.800821	4.517009	5.385118	3.347068	1.223557937	high
TCGA-AA-3848	0.838356164	1	3.301636	5.256693	5.372428	7.374502	7.195657	4.685344	3.014774	3.967556	3.594059	2.328966654	high
TCGA-F4-6463	2.978082192	0	4.142554	6.815318	4.566664	8.534271	6.379222	6.453037	3.92951	4.098562	3.103253	1.567370938	high
TCGA-AA-3867	2.002739726	0	5.228946	6.953487	4.965142	8.119675	5.69348	6.529801	3.670766	6.064507	3.318236	2.447122137	high
TCGA-AA-A01S	0.084931507	0	2.319618	6.286349	5.944885	8.530112	4.001646	4.010998	4.420335	5.495346	3.252937	1.434073431	high
TCGA-AA-3514	0.084931507	0	7.943146	5.288441	4.387643	7.330855	6.332654	6.278837	3.110036	5.063881	2.633203	8.781184972	high
TCGA-AY-6196	0.016438356	0	4.492549	6.063023	4.792391	7.770517	7.770537	7.038077	3.098063	4.907395	3.4442	3.68217537	high
TCGA-A6-6652	2.057534247	0	1.883151	6.647272	4.130499	7.967469	5.022154	5.372724	3.489051	5.1998	3.587011	3.252166571	high
TCGA-A6-3808	2.778082192	0	4.132649	6.556008	5.604782	8.297674	7.861776	7.330286	3.794146	3.019203	2.441277	1.479564616	high
TCGA-AD-6964	0.906849315	1	4.503282	6.426279	5.03367	8.225014	7.710573	6.247269	3.521731	4.018506	2.763471	2.296122174	high
TCGA-AA-3833	1.328767123	0	4.391603	7.04544	5.340568	8.522223	7.126713	6.087727	3.975286	3.021259	2.366371	0.976075678	high
TCGA-5M-AAT6	0.794520548	1	3.288062	6.082137	5.244311	8.229383	9.983084	6.155675	3.176734	3.70769	2.313613	4.48322365	high
TCGA-F4-6460	2.663013699	1	5.636566	7.288941	5.298139	7.749627	6.127936	7.694015	3.595987	4.818483	3.372974	1.084307075	high
TCGA-AZ-4616	0.42739726	1	3.137182	6.19905	4.555191	8.489984	9.092502	6.057657	4.0389	3.182586	2.27611	3.699545522	high
TCGA-AZ-4614	0.471232877	1	3.631255	6.132045	5.406365	8.197602	7.90934	6.4237	3.745783	3.440332	2.392861	2.654806286	high
TCGA-AA-3673	4.169863014	0	4.010562	6.985525	5.445323	8.452929	7.011906	5.343107	4.202019	4.87946	2.769634	1.570649338	high
TCGA-AA-3973	1.087671233	0	2.49842	6.162046	5.152236	7.974592	4.63933	5.833408	3.928131	4.50038	3.434359	1.888016324	high
TCGA-AY-4070	1.35890411	1	4.953428	6.766645	4.802835	8.419857	6.028514	6.138052	3.663401	5.389899	2.818537	3.083108898	high
TCGA-AA-3952	0.167123288	1	2.458114	6.339848	5.814861	8.330434	8.644711	6.537982	4.431863	3.757149	2.416908	2.352595189	high

TCGA-AA-3529	0	1	6.04673	6.787453	4.972364	8.030889	4.51771	5.782337	3.823002	5.456372	2.628515	2.704206519	high
TCGA-NH-A50V	1.610958904	0	2.924139	6.63567	5.654965	8.349412	8.679622	6.04363	3.871188	4.322144	3.426558	1.157313686	high
TCGA-AA-3502	2.917808219	0	3.420943	6.767352	4.951655	8.307347	5.081153	4.634181	3.748043	4.341817	3.035869	1.391100173	high
TCGA-SS-A7HO	5.010958904	0	4.379112	6.271706	5.151251	8.142867	5.748149	5.300048	3.345076	4.713384	3.564768	1.437901053	high
TCGA-AA-A01Q	0.084931507	0	3.24723	6.381224	5.324297	8.502525	6.790204	4.165728	3.9707	3.600582	2.848936	1.172127496	high
TCGA-AA-3553	2	0	5.435578	6.786688	5.389942	8.274756	6.065136	5.567876	4.626533	5.038027	2.792428	1.500561119	high
TCGA-CK-6746	0	0	2.24662	6.342895	5.670163	8.774801	10.161661	3.847347	3.644665	2.844875	2.529211	1.214269025	high
TCGA-CA-6717	1.063013699	0	3.622739	6.662661	5.225745	8.109136	6.169522	7.105671	3.765119	2.659999	2.477072	1.348652616	high
TCGA-G4-6298	0	1	3.407306	6.603603	4.424847	7.908564	4.658149	6.750823	4.313421	5.098233	3.163563	3.289509035	high
TCGA-D5-6930	1.112328767	0	3.056707	6.640228	5.397801	8.002551	7.905653	6.293257	3.86786	2.821591	2.561097	1.363472071	high
TCGA-AA-3660	6.506849315	0	5.896854	6.314159	5.439706	8.316554	7.700088	6.759729	3.703573	3.937633	3.203386	1.12320955	high
TCGA-AA-3672	0	0	7.886804	5.600581	4.918691	7.549276	9.02756	4.706855	3.090701	3.319983	3.140835	1.432611783	high
TCGA-AA-A03F	0	0	5.701554	5.997467	5.502975	8.123191	5.480664	5.056765	3.831976	3.831314	2.585627	1.560850822	high
TCGA-AA-3851	2.756164384	0	5.983782	7.314318	5.707243	8.411691	8.620767	5.500742	4.157465	5.100963	2.544559	1.317721426	high
TCGA-AZ-4308	9.106849315	0	6.665275	5.785469	5.672094	8.624025	7.634531	6.517856	3.055778	4.313776	3.32289	1.36408503	high
TCGA-AA-A02F	3.331506849	0	2.626633	5.833048	5.333751	8.712295	4.230264	7.600943	3.465494	2.774918	3.430416	1.02311313	high
TCGA-G4-6311	3.284931507	0	4.119182	6.567655	5.398455	8.187378	6.206941	6.075237	3.767805	4.722895	2.96467	1.916323624	high
TCGA-A6-2682	1.161643836	1	4.952859	6.452774	5.487233	8.348371	6.420245	6.869466	4.115069	5.517748	2.53383	3.798616169	high
TCGA-NH-A6GA	0.82739726	1	6.018363	6.386672	4.464272	7.435819	7.269615	5.869519	4.160716	3.999931	2.330247	3.555475573	high
TCGA-AY-A69D	1.487671233	0	2.987927	6.126907	5.408303	8.338705	4.595827	5.135357	4.066947	3.555326	3.154737	1.049229958	high
TCGA-AA-3975	2.838356164	0	5.189819	6.407737	5.143863	8.080668	5.278089	5.54449	3.715414	5.149678	3.486419	1.503741484	high
TCGA-A6-A5ZU	0.802739726	0	4.094779	6.943898	4.768969	7.964914	6.307642	5.608987	3.447634	3.978326	3.496824	0.972935894	high
TCGA-WS-AB45	5.835616438	0	3.360777	6.164626	5.484394	7.647195	6.542816	7.692237	3.117913	2.73853	2.605163	2.094572896	high
TCGA-CM-5862	0.419178082	1	4.091578	6.510704	5.780317	8.238292	5.01515	5.439216	3.484059	5.296348	2.74599	2.338886784	high
TCGA-D5-6932	0.947945205	0	2.24829	6.126961	5.516308	8.417605	8.62045	6.112282	3.549849	3.189834	3.438799	1.105811114	high
TCGA-F4-6569	2.978082192	0	3.594534	6.328532	5.38124	8.078117	7.132699	6.601549	3.398881	2.525242	2.565113	1.413545388	high
TCGA-A6-2679	3.742465753	0	6.141651	5.610558	6.263116	9.172552	7.904465	5.836758	3.270255	4.822585	2.73059	2.051910392	high
TCGA-A6-5657	2.635616438	0	3.333899	6.519863	5.052867	8.287354	7.603699	7.223567	3.884299	3.312473	2.641445	2.074647433	high
TCGA-CM-6169	1.084931507	0	4.71281	6.504572	4.847741	8.02782	8.160989	6.610071	4.18449	3.102637	3.222706	1.018893111	high
TCGA-AA-3980	0.663013699	0	4.619617	6.54325	5.931798	8.688995	5.379637	4.625695	4.17926	4.332423	2.349862	1.289169087	high
TCGA-DM-A28H	9.756164384	0	5.587654	6.083018	5.934063	7.618442	4.062705	4.976993	4.267151	4.34807	2.834432	1.195633065	high
TCGA-G4-6302	0	0	4.223967	5.960011	4.988342	7.93935	8.247216	9.002833	3.319809	2.93798	2.256998	4.724964008	high
TCGA-F4-6703	3.989041096	0	2.675101	5.961776	4.801063	7.88918	8.552989	6.751726	2.709979	3.021992	2.490057	4.661671523	high
TCGA-AA-A004	1.161643836	0	8.309287	5.401545	4.914754	9.036264	7.601362	6.113662	3.736086	5.08025	2.790588	3.539531951	high
TCGA-AA-A01P	3.17260274	1	5.510343	5.583477	5.348457	8.223379	9.523786	6.780377	3.524817	3.394583	2.909727	2.214852308	high
TCGA-D5-6537	0.4	1	2.59684	6.588441	4.189897	8.633928	7.645446	4.408275	4.051463	2.844236	2.650416	1.684785552	high
TCGA-A6-A566	2.076712329	1	5.775427	6.205304	5.46297	8.331004	8.300321	7.348803	4.017864	3.332148	2.440135	1.800539106	high
TCGA-AA-A02K	1.167123288	1	5.42689	6.152688	5.179442	8.669616	9.416874	5.049786	4.546182	3.716483	3.074968	1.073756792	high
TCGA-CM-4751	2.252054795	0	4.112541	6.662036	5.576594	8.785793	9.094139	7.090773	4.14909	3.550986	2.193774	1.992494424	high
TCGA-F4-6704	0.128767123	0	5.881889	6.536912	4.805841	8.156578	7.845106	7.792164	3.940079	3.06544	2.691024	1.549202139	high
