# Supplementary material for: Single-Cell RNA Sequencing Reveals the Role of Epithelial Cell Marker Genes in Predicting the Prognosis of Colorectal Cancer Patients
Source: Dis Markers. 2022 Aug 1;2022:8347125. doi: 10.1155/2022/8347125 (PMC9372514; doi:10.1155/2022/8347125)
Supplement: Supplementary 2 — Table S1: clinical characteristics of the four CRC patients. Table S2: CNV scores of epithelial cell subclusters. Table S3: MEC subclusters of marker genes. Table S4: MECRG signature using the TCGA-CRC training cohort. Table S5: MECRG signature using the GSE17538 validation cohort. [file 8347125.f2.zip › Table S5.pdf]

TableS5 MECRGs signature in GEO testing set

id	survival time	state	LGALS2	GDE1	MPC1	GNG5	BST2	TPM2	PRELID2	CAPS	CACNA1D	riskScore	risk
GSM437095	4.704522425	0	4.45486	6.876871	4.805726	7.929478	4.269167	4.599491	4.699502	3.866809	4.074829	0.353905036	low
GSM437096	5.128767123	0	4.008833	6.500478	5.882049	8.777589	5.901271	6.037256	4.166055	4.120559	3.428792	0.653419191	low
GSM437097	4.064102082	0	6.786328	6.714067	5.280452	8.101568	7.421539	4.669563	4.269687	3.250427	2.825468	0.593640607	low
GSM437100	4.788290485	0	5.860948	5.788474	5.058547	7.943113	6.709874	5.518356	3.601096	3.739505	4.06251	0.733996803	low
GSM437101	4.282979921	0	5.197281	6.519879	5.237958	8.737384	7.319907	4.866792	4.520249	4.739782	3.442556	0.894105959	low
GSM437103	5.639482079	0	3.688265	6.56203	5.106876	8.752104	6.638157	5.569045	4.46188	3.517092	3.570676	0.600277794	low
GSM437107	0.297241509	1	4.99287	6.511826	5.710559	8.434342	6.085623	6.049061	3.838767	4.470126	3.515653	0.780752545	low
GSM437108	2.534659411	1	5.9919	6.433047	5.605605	8.147327	6.82274	5.996143	4.864475	4.179732	3.016988	0.920196911	low
GSM437112	2.380273973	1	2.664496	6.201459	5.614906	8.342679	6.399147	4.69045	4.237963	3.282535	3.569516	0.593322137	low
GSM437117	0.961643836	1	3.40413	6.608393	5.597043	8.415961	5.98494	6.318916	3.484265	3.784251	3.576526	0.72144333	low
GSM437120	4.315068493	1	6.238767	6.703269	5.034562	7.770738	5.958601	6.279043	4.033817	3.486817	3.399606	0.635839652	low
GSM437122	2.772328767	1	4.701357	6.801773	5.294393	8.161962	7.001948	5.942591	4.139573	3.53989	3.493362	0.566675948	low
GSM437123	3.066575342	1	4.866477	6.47645	5.495888	7.949724	6.002935	4.939189	4.374584	2.945645	3.743423	0.309680694	low
GSM437125	3.066575342	1	3.734211	6.117588	5.58072	8.023495	6.230086	5.563584	3.699251	2.958534	3.131199	0.877118516	low
GSM437128	1.993972603	1	4.239266	6.694736	5.70411	8.481076	5.421944	4.9872	4.47174	3.003678	2.957457	0.455345085	low
GSM437129	0.966575342	1	3.5445	6.783705	5.826435	8.360326	8.143171	5.290468	3.197947	3.105712	3.55171	0.437026298	low
GSM437131	2.26109589	1	5.196453	6.591878	5.59127	8.584435	5.42325	5.82005	4.855246	4.449908	3.722715	0.489234099	low
GSM437132	3.774246575	1	3.153849	7.035664	5.378628	8.737384	5.356825	5.236242	4.117478	3.69187	4.248752	0.246718073	low
GSM437136	6.295890411	1	3.106452	6.737748	5.012698	8.499978	5.045067	5.727271	4.281801	4.321204	3.805477	0.752203631	low
GSM437146	3.182465753	1	5.779764	6.221237	5.622429	8.552574	8.852945	4.801685	4.464455	3.528003	2.967485	0.766556309	low
GSM437151	0.348493151	1	3.866885	6.74311	5.030752	8.155145	5.503687	5.062988	5.296538	3.315348	3.263236	0.55303169	low
GSM437154	1.448219178	1	2.896115	6.730249	5.455876	8.5108	5.831112	6.499987	4.284669	3.483848	3.155148	0.82555257	low
GSM437164	4.744931507	1	3.040809	6.768617	5.058946	8.584435	4.810222	5.520655	3.568745	3.273434	3.554205	0.574164522	low
GSM437165	1.182739726	1	2.709443	6.489269	5.100514	8.791487	5.048697	3.978448	3.788136	2.4559	2.580787	0.814455546	low
GSM437168	2.72630137	1	2.947657	7.106	5.832704	8.832902	7.344796	4.932994	4.400354	4.029446	3.619821	0.390021626	low
GSM437169	4.153150685	1	3.038722	7.297602	5.209455	8.108986	4.454765	6.060099	3.383802	4.413046	3.380879	0.929069559	low
GSM437170	0.470136986	1	5.091195	7.074291	5.423914	8.664193	5.137537	6.27385	4.854401	5.474314	3.508128	0.813289178	low
GSM437177	1.631506849	1	2.85323	6.67177	4.996853	8.184098	7.318558	5.095062	4.442752	3.392533	4.053437	0.459391521	low
GSM437186	0.643122537	1	3.583836	6.223904	5.571472	7.981672	5.983885	6.297094	4.081	3.490414	3.352246	0.930752026	low
GSM437191	2.95890411	1	4.727301	6.599394	5.47164	8.889591	5.817105	6.090648	4.00495	3.751751	3.325526	0.627058317	low
GSM437193	1.451079002	1	3.985177	5.821094	6.282599	8.263527	6.464512	4.819403	4.711867	3.371607	2.900489	0.787562054	low
GSM437199	11.71643836	0	2.645765	6.500478	4.961668	8.323686	6.34044	7.171048	3.733022	3.444711	4.109299	0.706604356	low
GSM437205	6.914794521	0	5.750886	6.10952	5.328853	8.803722	6.268841	6.220657	4.864475	3.83694	3.599349	0.601366524	low
GSM437211	4.531232877	0	6.939284	6.156707	5.384563	8.246462	8.232822	5.47002	4.181667	3.254854	2.797918	0.908724322	low
GSM437214	3.599178082	0	5.299533	6.56415	5.304686	8.531232	5.799496	6.11446	4.631568	3.285935	3.511959	0.426603053	low
GSM437216	0.299178082	0	5.960063	6.50657	5.898375	7.594701	5.040177	4.869057	4.627304	3.406712	4.043272	0.202144895	low
GSM437217	3.00739726	0	5.998151	6.482604	4.92308	7.764954	4.970755	4.235327	4.96182	2.66794	2.606619	0.647217459	low
GSM437227	2.180547945	0	4.976824	8.125047	5.106876	8.386378	6.282274	5.239186	3.499484	4.176259	2.614876	0.710405941	low
GSM437229	4.596164384	0	3.883256	6.65053	5.129065	8.791487	7.296151	4.843405	4.315796	2.955705	2.713007	0.822567736	low
GSM437230	5.806849315	0	3.849967	6.619875	5.296514	7.856574	5.307313	5.851491	3.899524	4.09728	3.702045	0.778241176	low
GSM437233	6.903287671	0	2.98066	6.187285	5.061318	8.360326	5.535836	5.492676	4.379591	3.701459	3.720032	0.8381205	low
GSM437237	5.198630137	0	3.936327	6.783705	5.462769	8.305216	7.976091	5.573182	4.424572	3.288262	2.802094	0.862255864	low
GSM437238	2.801917808	0	3.383673	6.49745	5.265963	8.712513	7.749137	5.584179	4.043925	3.423786	3.590338	0.651510958	low
GSM437244	3.78	0	3.326612	6.945743	5.579314	9.141261	6.174259	6.193049	4.202519	4.344156	3.344994	0.726310628	low
GSM437251	3.696164384	0	4.110424	6.716576	5.273544	8.161962	6.800549	6.38661	4.304622	3.365334	4.037905	0.387211741	low
GSM437253	2.191232877	0	5.707217	6.373963	5.984363	8.386378	5.756606	5.569675	4.204588	4.026017	3.087523	0.680781335	low
GSM437256	1.82630137	0	3.744349	6.676412	5.854072	9.066312	6.082753	4.335727	3.70682	3.565373	2.645228	0.750373107	low
GSM437257	2.728767123	0	2.296798	6.110274	5.602995	7.943113	7.797682	4.896269	4.346397	2.315417	3.673727	0.449632321	low
GSM437258	3.261369863	0	5.852539	6.932157	5.41551	8.406197	7.179849	4.515717	4.255359	4.663504	3.208001	0.725035818	low

GSM437261	1.236986301	0	2.704721	6.96959	4.806124	8.415961	6.244509	5.459248	4.359975	3.871093	3.461997	0.85019074	low
GSM437264	8.644109589	0	2.736657	6.110274	5.924669	8.699975	5.175553	5.24153	4.104318	3.903584	3.51449	0.718548986	low
GSM437266	9.23260274	0	5.526094	6.209288	4.926639	8.605898	7.907793	4.842214	3.58664	2.724313	3.025302	0.793641154	low
GSM437267	8.54630137	0	2.61611	6.815642	5.55129	8.415961	6.95284	5.533778	4.082678	3.332009	3.038611	0.788599999	low
GSM437268	8.17890411	0	3.468847	6.170748	5.201694	8.777589	8.758629	4.540396	4.169748	3.224414	3.470857	0.727499122	low
GSM437269	7.911780822	0	3.062506	7.109261	5.689834	8.279663	4.337154	4.596734	4.410242	3.482863	5.230454	0.073802896	low
GSM437270	7.036438356	0	4.005209	6.616198	4.886891	8.406197	6.291096	6.747811	3.492612	3.092713	3.358616	0.868399533	low
GSM437271	6.482465753	0	4.800305	7.576782	6.096234	8.791487	6.558241	4.487554	4.649741	3.116917	2.428583	0.304358507	low
GSM437273	6.525205479	0	4.744562	6.238026	5.557976	8.434342	6.121679	6.03227	4.621926	2.883538	3.093179	0.59532394	low
GSM437274	6.087945205	0	6.93545	7.115748	6.683669	8.552574	4.513711	5.950944	4.801825	3.645144	2.890866	0.217746396	low
GSM437275	5.822465753	0	8.365491	6.892471	5.266519	8.874588	4.147096	4.480523	4.792165	3.077737	3.596119	0.132286235	low
GSM437277	5.582465753	0	4.410787	7.763005	5.666644	8.57444	3.763553	5.320471	4.870455	3.731766	3.836684	0.140671909	low
GSM437279	4.877260274	0	5.767621	7.012775	5.516693	8.396519	5.118764	5.737706	3.860157	2.88599	3.022847	0.364665109	low
GSM437281	4.344657534	0	5.607065	6.891038	5.957264	8.377914	5.597689	6.367349	4.535972	3.560365	3.283725	0.359579758	low
GSM437282	4.598630137	0	2.875947	6.783705	6.455172	7.922491	8.030629	4.283763	5.192005	2.559183	2.880105	0.323542156	low
GSM437283	4.657808219	0	8.392265	7.49007	7.197294	8.481076	4.65303	5.862398	5.106849	3.661124	2.934771	0.102883149	low
GSM437284	4.144931507	0	2.789373	6.67177	5.78279	7.981672	7.69949	6.191836	3.607528	2.552805	3.030522	0.69377346	low
GSM437285	4.036438356	0	4.741558	7.70225	5.262388	8.818084	3.728048	5.307277	5.13046	4.770992	3.424464	0.362652816	low
GSM437286	4.074246575	0	4.640895	6.609574	6.292849	8.079486	4.725329	5.005964	3.569693	2.827403	3.158935	0.319583846	low
GSM437287	4.149863014	0	3.458422	6.333191	5.738742	8.406197	3.931467	3.813178	3.733022	3.595655	4.090295	0.290157755	low
GSM437288	5.182191781	0	5.358666	6.391965	6.104206	8.434342	6.99134	5.994281	4.006018	2.632442	3.73821	0.226680655	low
GSM437289	0.075616438	0	3.92432	6.766055	5.398545	8.874588	6.976472	5.048038	4.193362	4.599554	4.277125	0.403878461	low
GSM437290	2.577534247	0	3.492887	6.639539	5.562972	8.453363	4.485778	6.792863	4.039662	4.023936	3.235523	0.940935456	low
GSM437291	3.312328767	0	4.085767	6.609574	6.242698	8.079486	6.356766	5.497541	4.80438	3.700418	3.301686	0.455753929	low
GSM437292	9.586849315	0	3.983461	6.597215	6.624769	7.943113	4.308832	3.726643	4.994082	2.919087	4.6738	0.058866295	low
GSM437293	2.623561644	0	6.13162	6.724126	5.407316	8.140315	5.752292	6.213133	3.947824	2.813469	2.912697	0.529266459	low
GSM437294	1.642191781	0	4.032608	6.818102	5.993205	8.57444	8.984062	5.225358	4.078955	2.506793	2.632134	0.512280748	low
GSM437295	2.023561644	0	3.957286	6.750776	5.804246	9.240194	6.201833	6.377944	4.170633	2.987236	3.898295	0.220350283	low
GSM437296	10.74657534	0	4.270834	7.210759	5.915678	9.101667	4.394818	6.07343	4.462913	4.292245	2.529745	0.764964739	low
GSM437297	7.813972603	0	3.939308	6.724126	6.626326	8.752104	8.388256	4.232098	3.660973	2.340797	3.020963	0.234882181	low
GSM437298	4.993150685	0	3.405224	6.436209	5.645179	8.605898	4.906428	4.597814	4.813079	3.223051	3.476541	0.376414555	low
GSM437299	2.617808219	0	4.644161	6.801773	6.079511	8.351597	6.486255	4.021468	4.305905	3.057927	2.787149	0.392779508	low
GSM437300	3.474246575	0	5.828971	7.2687	5.943646	8.542934	6.650009	5.059013	3.970309	3.09755	3.488309	0.178620908	low
GSM437301	2.685205479	0	3.873898	6.576346	5.068572	7.794967	8.316524	5.826597	3.515209	2.77401	3.150815	0.948354712	low
GSM437302	1.836986301	0	4.776166	6.791358	5.777993	8.651554	5.211445	5.257329	4.067694	3.245049	4.098298	0.175462809	low
GSM437303	5.253068118	0	2.777604	7.178748	6.049719	8.471333	6.160795	4.289802	4.095982	2.626728	2.620389	0.391331376	low
GSM437304	4.934209044	0	7.627709	7.590835	6.443664	8.640396	5.59259	6.507682	4.457625	3.118705	2.89174	0.15349618	low
GSM437305	4.934209044	0	3.984542	6.975692	4.475429	8.065029	5.581237	4.899094	3.948119	3.286937	3.368294	0.704269289	low
GSM437306	4.977444173	0	5.652586	6.836901	5.893703	8.499978	6.796437	6.236678	3.736572	2.70255	2.51961	0.558750619	low
GSM437308	4.591030211	0	3.217567	6.966485	5.859674	8.542934	7.945973	4.894453	3.890055	2.807538	3.336039	0.336325887	low
GSM437309	5.331431789	0	6.791828	6.865279	5.679272	8.453363	6.654902	5.398484	4.334836	2.928361	2.78914	0.378938701	low
GSM437310	4.499155564	0	3.417262	5.516053	5.228975	8.664193	3.748744	5.424186	4.880372	3.396232	4.245882	0.469925352	low
GSM437311	4.912591479	0	4.182835	5.38687	5.760621	8.207024	6.924447	5.867327	3.694923	3.516779	3.742927	0.963316891	low
GSM437312	4.480240195	0	4.967311	7.106	5.22967	8.520747	3.857092	4.248769	4.727643	3.032838	2.749662	0.388303706	low
GSM437316	6.106961907	0	4.365514	6.720343	6.803385	10.167164	2.729161	5.431649	4.718443	1.870303	3.235288	0.072060452	low
GSM437317	3.81820229	0	4.147808	6.74026	5.581418	8.396519	7.978279	5.706782	3.722188	2.483465	2.540859	0.766308072	low
GSM437319	5.685419403	0	5.799796	6.879817	5.978073	8.552574	7.255316	5.348541	3.7622	3.164801	2.703714	0.494794259	low
GSM437320	3.861437419	0	6.118649	6.623454	6.197951	8.424816	6.23519	4.861771	4.656299	2.671378	2.75895	0.292643893	low
GSM437321	3.342615877	0	4.853823	6.543502	5.739802	8.491169	5.693692	3.635189	4.056423	2.789922	2.904617	0.387626527	low
GSM437322	3.348020266	0	4.148222	6.902277	6.003711	8.161962	6.097816	4.298888	4.613072	3.326851	2.616939	0.540603468	low
GSM437323	5.79080503	0	4.873634	6.57169	6.061232	8.765399	5.03416	4.830023	4.703782	3.925119	3.525038	0.31899438	low

GSM437324	3.404766373	0	3.723078	6.903756	6.323459	8.542934	5.055296	4.643721	4.313602	3.110214	2.960942	0.315588094	low
GSM437093	4.442409458	0	3.782633	6.669422	5.247045	9.018432	7.457681	5.212128	3.866421	4.328645	2.813837	1.51613166	low
GSM437094	4.763970726	0	3.70752	6.461631	4.797271	8.640396	5.42985	5.80617	4.42764	3.766997	3.14858	1.1840693	low
GSM437098	5.274685682	0	4.493259	6.572811	5.383529	7.949724	6.351093	5.344456	4.053091	3.429189	2.715392	1.104092632	low
GSM437099	4.126252581	0	3.277619	6.512855	5.577597	8.079486	6.053	4.242876	4.691319	3.636902	2.716535	1.013399465	low
GSM437102	9.163145055	0	3.522128	6.047127	4.813775	7.838462	6.555054	6.570434	3.342309	3.152574	2.625933	2.959473584	low
GSM437104	4.377556764	0	2.852168	6.324856	6.214969	7.862322	9.156118	4.773472	3.910236	3.941611	3.037338	1.179909598	low
GSM437105	4.453218241	0	5.565316	6.123161	6.172138	7.819155	8.261018	5.591853	3.856006	3.843891	2.518395	1.493810723	low
GSM437106	3.966823044	1	7.158567	6.070185	5.129756	8.140315	8.174833	6.487417	4.268181	3.946288	2.339338	2.484488166	low
GSM437109	3.36963783	1	5.755645	5.906066	4.507527	7.740072	8.735653	6.017121	4.085271	3.846622	2.364616	4.587739207	low
GSM437110	0.670144492	1	3.91392	6.604951	5.474079	8.499978	8.208193	5.888562	4.094094	3.642159	2.613069	1.429748109	low
GSM437111	0.42964909	1	8.019364	6.30433	4.376645	8.552574	8.391102	5.059601	3.882567	3.909929	2.823516	1.502802969	low
GSM437113	5.655616438	1	4.03607	6.669422	4.39471	8.57444	5.192998	4.713493	4.249252	4.134176	3.36265	1.089070074	low
GSM437114	5.414794521	1	3.290135	6.707977	4.882632	8.453363	5.3823	5.049182	4.752826	3.241697	2.693906	1.044667364	low
GSM437115	1.35369863	1	3.664537	6.614045	5.009281	8.803722	5.285309	5.590664	4.707375	5.22435	3.225161	1.657672408	low
GSM437116	11.08438356	1	4.05583	6.397632	4.476498	8.491169	7.989813	5.299058	3.889769	2.772097	3.219764	0.974692097	low
GSM437118	2.36630137	1	2.314011	5.99926	5.406232	8.5108	10.052501	5.212128	3.309127	3.363993	2.157322	3.933665961	high
GSM437119	2.204383562	1	4.341442	6.291564	4.7257	7.770738	5.387743	7.254711	3.521811	3.945283	2.797918	3.053484886	high
GSM437121	1.975068493	1	2.965847	6.260189	4.796532	8.396519	8.408726	5.378422	4.076657	3.527343	2.982546	1.88933314	high
GSM437124	2.552876712	1	3.389647	5.946238	5.255066	7.161943	6.541923	6.146629	4.764505	3.685934	3.104134	1.916749653	high
GSM437126	1.809863014	1	3.650975	5.885663	5.753176	8.214794	9.257297	4.908003	4.26873	3.046087	2.091994	2.230476833	high
GSM437127	2.231506849	1	4.996006	5.997732	5.058946	7.988906	6.382433	5.474033	4.106978	3.32252	2.457556	2.02937281	high
GSM437130	5.790410959	1	2.400396	6.557663	4.792279	8.314888	8.550061	5.934485	3.935273	3.167154	2.406647	2.617817457	high
GSM437133	4.441643836	1	2.902607	6.098017	5.24421	8.132842	10.071748	6.091883	3.146438	3.858184	2.371343	4.901513097	high
GSM437134	0.502191781	1	4.036529	5.866362	5.469215	8.101568	5.886106	5.794641	4.276135	4.000871	2.855162	1.960499468	high
GSM437135	1.68	1	2.704997	6.070978	4.841152	8.108986	7.409292	6.74425	3.395511	3.503682	2.80132	3.312420499	high
GSM437137	0.823561644	1	3.380558	6.091083	4.707344	8.542934	8.367434	5.999404	3.78108	3.674247	3.648857	1.399241605	high
GSM437138	3.406849315	1	3.162841	6.044006	5.751705	8.214794	6.382857	6.161433	3.865662	4.089993	3.129703	1.611102448	high
GSM437139	1.828767123	1	3.235079	6.683322	4.931813	8.434342	8.484045	5.902084	3.217527	4.452338	3.292482	1.956907055	high
GSM437140	4.855068493	1	3.415979	6.107162	5.520011	8.462447	5.026478	5.132157	4.024215	4.59698	3.550084	1.134960993	high
GSM437141	3.928767123	1	3.819215	6.773758	5.148438	8.444041	8.399759	6.017445	4.693305	3.778626	2.818615	1.299844358	high
GSM437142	0.466849315	1	2.647191	6.144359	4.484225	8.664193	7.711389	6.872433	3.210812	3.93446	2.347782	6.542770012	high
GSM437143	4.172054795	1	7.365993	6.222143	5.608088	8.198961	9.182996	4.649561	4.019764	3.413404	2.469679	1.032844474	high
GSM437144	5.574246575	1	3.695157	6.796411	5.440157	8.542934	6.114633	6.845141	3.770899	4.410578	3.011169	1.459120474	high
GSM437145	2.842191781	1	4.268886	6.351415	5.5555	8.491169	8.788428	6.112608	3.79415	3.819504	2.603953	1.877924905	high
GSM437147	4.512328767	1	3.766312	6.061632	5.643779	8.191262	8.221293	5.58586	3.951359	6.112599	3.007048	4.682317068	high
GSM437148	1.564109589	1	3.18683	6.172386	5.962061	8.125491	8.304614	5.289292	4.463339	3.821141	2.562855	1.734287017	high
GSM437149	2.158356164	1	6.847254	6.219575	5.228638	8.03086	8.031798	6.05045	4.396636	3.101335	2.633863	1.095291735	high
GSM437150	2.634246575	1	2.700498	6.388067	5.488698	8.254869	4.605211	5.173943	3.197114	3.809927	2.828255	1.573808017	high
GSM437152	4.047123288	1	4.787564	6.553402	4.834755	8.002707	7.282583	6.404055	3.580402	3.69552	2.61278	2.259823542	high
GSM437153	1.523835616	1	4.133404	5.912395	4.388701	8.184098	4.71284	5.728911	3.574951	3.960036	3.013608	2.830454583	high
GSM437155	0.437260274	1	3.366731	6.730249	5.511507	8.594947	7.178601	6.402699	4.135287	3.158217	2.373339	1.329980367	high
GSM437156	0.193972603	1	3.8934	5.977304	5.640719	8.05829	9.029342	6.148544	4.234014	3.302412	2.408325	2.255564004	high
GSM437157	0.470136986	1	3.585303	5.672357	5.504204	8.147327	7.191553	5.539637	3.273365	3.432569	2.518698	2.826735032	high
GSM437158	1.255890411	1	2.96868	6.332182	4.697488	8.155145	6.077647	5.196508	3.604957	4.760065	3.393612	2.385217318	high
GSM437159	0.407671233	1	3.990261	6.884126	5.381796	8.305216	6.238712	5.563584	3.897339	4.446673	3.032898	1.199648312	high
GSM437160	2.374520548	1	3.046857	5.939718	5.052278	7.419746	8.818478	4.20422	3.587333	3.472731	2.600457	3.130108567	high
GSM437161	0.267123288	1	4.59407	5.873252	5.13217	8.263527	8.219786	5.779937	3.830727	4.699542	3.138906	2.806162961	high
GSM437162	0.075616438	1	5.029112	6.447428	5.107884	8.222796	7.462273	5.93513	4.072413	3.825392	3.178249	1.130407579	high
GSM437163	1.542739726	1	5.626347	6.463465	5.314929	8.640396	6.633855	6.593614	3.80139	3.646415	2.792379	1.17747684	high
GSM437166	0.420821918	1	4.623746	6.301603	4.860943	8.542934	5.764495	4.944293	4.615372	2.867876	2.477593	1.141626806	high

GSM437167	0.410136986	1	7.619587	5.91903	4.942668	8.207024	8.60288	4.997547	4.200405	2.901485	2.73827	1.018799321	high
GSM437171	0.659178082	1	4.156541	6.47645	5.152556	8.453363	9.046333	6.155645	3.710239	3.401652	2.848057	1.496075547	high
GSM437172	2.796164384	1	2.898204	6.672983	5.421491	8.351597	6.354473	5.675306	3.89625	3.371607	2.892005	1.047627415	high
GSM437173	0.118356164	1	3.05774	7.032645	5.503525	8.238509	6.295608	6.792238	3.38843	4.578903	2.706849	2.129924685	high
GSM437174	3.036986301	1	2.612371	7.106	5.232824	8.675906	9.156118	5.948555	4.515904	2.829691	2.439723	1.055914094	high
GSM437175	3.077260274	1	3.614423	6.314335	4.713449	8.369369	7.40495	6.337374	3.220709	4.099618	3.134992	2.513570894	high
GSM437176	1.896164384	1	3.759559	5.966072	4.625353	7.710733	6.194961	5.866107	4.29401	3.475173	3.567241	1.376093841	high
GSM437178	0.707671233	1	4.014453	6.008809	5.542465	7.936409	8.477641	6.556464	3.779311	2.995564	2.362954	2.310741113	high
GSM437179	1.253424658	1	2.080302	6.142852	4.784396	7.974867	5.567179	5.318188	3.718863	5.064591	3.472433	3.156440134	high
GSM437180	1.196712329	1	3.121067	6.010237	4.772101	8.132842	5.188063	6.230882	4.17994	3.259302	3.334375	1.376835875	high
GSM437181	0.818630137	1	5.964478	6.381455	5.221171	8.333047	6.961531	6.52558	3.661692	3.79963	2.72744	1.550764262	high
GSM437182	0.734794521	1	2.380425	6.767375	4.877733	8.093756	6.321315	5.618437	4.601823	3.712442	2.836969	1.596343908	high
GSM437183	0.170136986	1	2.757441	6.719139	5.1415	8.481076	5.487905	5.123245	3.87811	4.336008	3.409558	1.070045938	high
GSM437184	1.872328767	1	3.528775	6.097233	5.298225	8.360326	8.855043	5.04633	4.278985	2.735379	2.508407	1.460475065	high
GSM437185	3.804691312	1	3.995103	7.014366	4.692104	8.207024	5.51257	5.087554	3.962432	4.421625	3.250882	1.169133845	high
GSM437187	1.92396322	1	6.211111	6.397632	5.206628	8.699975	5.743077	5.496939	3.820221	4.686214	2.516026	2.030904735	high
GSM437188	1.778044661	1	3.241247	6.294333	4.909773	8.406197	6.660623	6.011549	3.961862	5.608801	2.870286	5.098391937	high
GSM437189	2.721110903	1	4.454973	6.127872	4.749923	8.552574	6.704406	5.900891	3.675308	5.479412	3.380879	3.185391618	high
GSM437190	1.13762432	1	2.936068	6.3533	5.171318	8.169015	5.962635	6.030864	3.739658	3.224055	2.821252	1.545116831	high
GSM437192	0.12700319	1	4.267071	6.57169	4.935996	8.453363	5.664225	6.160768	3.256051	4.861771	3.483193	1.649642338	high
GSM437194	1.040345281	1	5.157043	6.744336	4.863077	8.499978	6.120457	6.355848	3.99233	4.915102	3.112042	1.762511504	high
GSM437195	0.567461062	1	3.529179	6.473458	5.560856	7.819155	5.981755	5.913236	4.053371	4.251368	2.905696	1.685879213	high
GSM437196	2.672471383	1	3.28876	5.810198	5.078751	8.386378	6.721985	5.951563	4.219774	3.360279	2.093102	3.853871513	high
GSM437197	1.326778007	1	3.325288	6.588492	5.527198	7.943113	6.112449	6.521069	3.809847	3.75769	2.706525	1.68967763	high
GSM437198	0.035128542	1	4.055612	7.355001	4.706275	7.764954	7.303689	6.971128	3.816766	3.770255	3.123495	1.199763813	high
GSM437200	10.08657534	0	3.700425	6.444297	5.406544	8.396519	6.310822	6.682185	3.642305	3.166104	2.714212	1.341264873	high
GSM437201	9.798082192	0	2.600696	6.461631	4.953521	7.782855	8.511687	7.33941	3.711898	4.949593	2.489914	7.659131726	high
GSM437202	4.892876712	0	4.7624	6.499442	5.196564	7.895712	9.346509	5.925791	3.930128	5.889064	2.714212	5.321650141	high
GSM437203	6.763561644	0	5.889549	6.397632	5.189285	7.75863	8.546883	5.721156	3.939761	3.773699	2.093429	2.805183485	high
GSM437204	9.108493151	0	3.070801	6.319045	5.348999	7.198584	6.571948	10.49466	2.970506	3.284482	3.057972	3.055667962	high
GSM437206	7.376712329	0	2.556415	6.215151	4.907601	8.520747	5.755039	5.759347	4.340554	3.844599	3.680024	1.065986741	high
GSM437207	6.252328767	0	3.017527	6.724126	5.612757	8.594947	8.744981	6.246297	3.387336	3.954062	3.248747	1.139441919	high
GSM437208	5.574246575	0	4.029186	6.292546	5.082266	8.169015	8.65854	6.478186	3.823765	3.970321	3.383259	1.57406456	high
GSM437209	4.29369863	0	3.338878	5.755102	4.939757	8.023495	9.365842	6.233389	3.33998	3.396969	2.304127	5.42466832	high
GSM437210	5.268493151	0	6.943789	5.899987	5.054027	8.101568	8.157933	6.57646	3.874792	3.600354	3.126159	1.398873642	high
GSM437212	4.749863014	0	3.629063	5.719045	5.285987	7.962567	9.740196	5.76495	3.622494	3.256929	2.419418	3.535709991	high
GSM437213	4.117808219	0	3.361972	5.713418	4.881551	7.429735	9.041584	5.004327	4.655949	3.706156	2.599866	3.937918314	high
GSM437215	2.487945205	0	4.722985	6.199789	5.534877	8.169015	6.056552	5.657428	4.111645	3.980138	2.69702	1.603533729	high
GSM437218	1.645479452	0	5.010536	6.204946	5.72772	8.238509	8.095696	5.895846	4.291204	4.640567	3.274004	1.31287468	high
GSM437219	9.74630137	0	4.609245	6.733801	4.871464	8.044305	4.897066	4.440045	4.474597	4.167644	3.013307	1.062481345	high
GSM437220	4.547671233	0	4.7661	5.951986	4.671034	7.902248	8.039669	5.081717	3.61292	2.989612	2.555825	2.356737535	high
GSM437221	10.46219178	0	4.306096	6.175639	5.772196	8.584435	5.838866	5.668531	3.568997	4.432644	3.449883	1.008227953	high
GSM437222	9.913972603	0	7.019559	6.124725	5.091999	8.263527	7.033187	5.769644	3.990382	6.49028	2.380464	7.253808338	high
GSM437223	8.789589041	0	2.747063	6.698399	4.683716	8.214794	8.044051	5.995545	3.489075	3.424497	2.542923	2.633412549	high
GSM437224	7.314246575	0	3.961309	6.3645	4.685881	8.444041	8.774444	6.335535	3.916674	6.340924	3.268962	5.93479973	high
GSM437225	8.419726027	0	5.554109	6.501468	4.775583	8.737384	8.744981	6.392946	3.787049	3.438892	2.854132	1.404905723	high
GSM437226	7.009315068	0	6.129187	6.062422	4.812757	7.813036	8.204318	5.748802	3.35986	4.484649	2.760077	3.474541668	high
GSM437228	7.103835616	0	2.950737	6.736382	5.177443	8.752104	6.357002	5.890972	3.656423	6.348836	3.185675	3.702158322	high
GSM437231	6.249863014	0	4.766364	6.656459	4.992124	7.64625	5.941441	6.104133	4.616183	3.18985	2.768205	1.091993213	high
GSM437232	6.503835616	0	3.793467	6.507694	5.544322	9.050307	5.731478	4.951779	4.708627	4.590827	3.019421	1.044567533	high
GSM437234	6.595890411	0	3.423809	6.578438	4.999236	8.406197	6.019355	5.709823	3.788136	3.541222	2.569445	1.799769789	high

GSM437235	3.336986301	0	3.866885	6.461631	5.081158	8.155145	7.000242	5.803572	3.90006	4.131545	3.325748	1.363629049	high
GSM437236	7.838630137	0	2.229155	5.827098	5.423914	7.838462	9.1027	5.290468	3.393105	3.475867	2.848057	2.808423599	high
GSM437239	4.285479452	0	1.817149	6.309822	5.287775	8.27126	5.841868	4.998733	3.572704	3.187437	2.188323	2.551063034	high
GSM437240	5.427945205	0	4.06814	6.301603	4.662838	8.377914	5.194963	6.394329	3.410824	5.047285	3.457379	2.542152782	high
GSM437241	4.928219178	0	3.821357	6.251158	5.650957	8.791487	5.846913	6.69452	3.621778	3.978501	2.887253	1.523819633	high
GSM437242	3.93369863	0	2.629135	5.700341	4.568901	8.254869	5.987314	6.159722	4.417172	3.360279	3.325748	2.021391516	high
GSM437243	4.804109589	0	2.922223	6.606046	5.133581	8.712513	8.583665	6.797767	4.531962	3.938806	2.801043	1.952550038	high
GSM437245	4.966027397	0	2.774341	6.230985	5.488367	8.406197	7.576126	6.433931	3.707081	3.766652	2.710764	2.293782074	high
GSM437246	3.096164384	0	3.294622	6.488167	5.291933	7.483079	6.162642	4.955159	3.573589	4.142129	3.598221	1.082862851	high
GSM437247	1.291232877	0	3.78366	5.994844	5.52646	8.57444	8.21211	5.950255	3.904931	3.802932	2.965279	1.69479825	high
GSM437248	3.226027397	0	3.805577	6.6211	5.103365	8.214794	8.447988	5.333363	4.363302	3.921645	2.688044	1.777104433	high
GSM437249	4.09890411	0	3.143011	6.111921	5.222663	8.27126	5.010757	6.145462	4.471039	4.647398	2.728614	3.056243573	high
GSM437250	2.074520548	0	2.736132	5.712839	4.796215	7.881614	6.921193	6.507682	3.652532	3.579329	3.720714	1.90573392	high
GSM437252	2.950684932	0	4.734907	6.573897	5.333358	8.360326	6.05904	5.487947	4.248594	4.754289	2.997568	1.467075151	high
GSM437254	3.023013699	0	5.683175	6.306037	5.603396	7.902248	6.979859	6.220657	3.377085	3.781627	2.787149	1.450538334	high
GSM437255	3.41260274	0	4.590733	6.420783	5.196147	8.314888	6.098934	5.104129	4.872233	4.393535	3.273512	1.01562919	high
GSM437259	2.104931507	0	4.640408	6.37105	5.881358	8.491169	5.387743	6.731601	3.777561	5.458639	3.305659	1.658378902	high
GSM437260	6.903287671	0	4.257979	5.951321	4.991139	7.929478	6.809353	6.032885	3.643283	3.322205	2.975813	1.859341807	high
GSM437262	0.688767123	0	2.865101	6.376802	4.608856	8.499978	7.513555	6.066438	3.886263	4.912032	3.814946	2.005006663	high
GSM437263	1.494246575	0	2.63625	6.174026	5.208034	8.314888	6.661104	5.51894	4.275793	2.843607	2.913205	1.172052785	high
GSM437265	7.813972603	0	3.419602	6.511826	4.912212	7.981672	8.429493	6.336766	4.12408	3.879158	3.112521	1.873639214	high
GSM437272	6.125753425	0	3.520817	6.892471	5.233842	8.491169	5.202928	4.784103	3.381839	3.942291	2.564645	1.420314911	high
GSM437276	4.158082192	0	4.307931	6.586365	5.601623	8.874588	5.994009	7.200494	3.101616	4.496551	3.568634	0.998279282	high
GSM437278	4.804109589	0	4.130158	7.002588	5.284975	8.5108	5.499004	7.001477	3.320814	3.930415	2.961733	1.15610794	high
GSM437280	4.482739726	0	3.566257	7.309876	5.298922	8.846624	5.968635	7.224309	2.967522	5.221753	3.188956	1.665334212	high
GSM437307	4.691011447	0	2.629135	6.023596	5.488017	8.377914	9.04893	4.503308	3.042384	2.816787	2.331007	2.188572414	high
GSM437313	5.593544756	0	4.134561	6.755775	5.629875	8.462447	8.880513	5.179754	3.522988	2.627864	2.100719	1.155003479	high
GSM437314	4.120848189	0	2.747063	6.66006	4.974531	8.207024	8.812388	6.335535	4.009038	2.601473	2.372328	1.808316402	high
GSM437315	5.823231375	0	4.191821	6.637214	5.350408	8.396519	7.675315	8.25352	3.250143	2.66794	2.43882	1.600609297	high
GSM437318	6.309626573	0	3.293248	6.875385	5.867179	8.279663	8.001472	6.020345	3.722943	2.832016	2.335151	1.03451669	high
